# Cu-Catalyzed Enantioselective Protoboration of 2,3-Disubstituted 1,3-Dienes

**DOI:** 10.1021/acs.orglett.3c02627

**Published:** 2023-09-11

**Authors:** Sensheng Liu, Yangbin Liu, Arthur Flaget, Cheng Zhang, Clément Mazet

**Affiliations:** Department of Organic Chemistry, University of Geneva, 30 quai Ernest Ansermet, 1211 Geneva, Switzerland

## Abstract

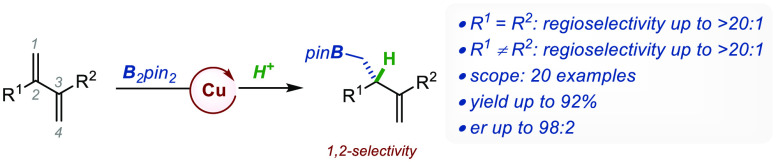

A Cu-catalyzed regio- and enantioselective protoboration of 2,3-disubstituted 1,3-dienes is described. The protocol operates under mild conditions and is applicable to symmetrically and unsymmetrically substituted dienes, providing access to homoallylic boronates in consistently high yield, regioselectivity, and enantiomeric ratio. Preliminary investigations point to a complex mechanism.

The growing interest for Cu-catalyzed selective borofunctionalizations of 1,3-dienes witnessed in recent years stems from the diversity of polyfunctional (chiral) alkenyl boronates that can be generated and the net increase in molecular complexity enabled by these approaches.^1^ At their most sophisticated, the development of these methods requires overcoming challenges associated with chemo-, regio-, and enantioselectivity (Figure 1A). The substitution pattern of the 1,3-dienes has a profound influence on these aspects and affects product distribution. Therefore, much of the work in developing enantioselective borofunctionalization reactions hinges on the design or identification of a chiral ligand capable of exerting high levels of chemo-, regio-, and stereocontrol irrespective of the innate biases imposed by the substrate. Among notable achievements, typical electrophiles include acyl silanes, aldehydes, ketones, imines, aryl halides, and cyanamides.^1^ In contrast, examples of enantioselective protoborations remain scarce (*E*^+^ = H^+^, Figure 1A).^2^ Even though little is known regarding their exact mechanism, it can be surmised that protonation of the putative Cu–allyl intermediate is likely to be enantio-determining in certain cases and, thereby, adds a layer of complexity to the development of these processes, in particular for acyclic substrates.^3^ In 2010, Ito and co-workers reported the preparation of highly enantioenriched homoallylic boronates by Cu-catalyzed protoboration of *cyclic* 1,3-dienes using **L1** (Figure 1B).^2a^ While 1,2-regioselectivity was predominant at low temperatures, the product of 1,4-addition was generated preferentially at ambient temperature. Of note, 3,4-protoboration of isoprene led to an achiral homoallylic boronate, and a modest *er* was obtained with a linear diene. Capitalizing on the modularity of a chiral monophosphane ligand (**L2**),^4^ the Mazet group disclosed a 1,2-regioselective Cu-catalyzed enantioselective protoboration of 2-(hetero)aryl *branched* 1,3-dienes (Figure 1C).^2b^ After *in situ* oxidation, acyclic homoallylic alcohols featuring a benzylic tertiary stereocenter were obtained with high levels of regio- and enantiocontrol. For alkyl substituted dienes, selectivity was enhanced with increasing steric demand, suggesting that for large substituents formation of a 1,4-σ-allyl copper intermediate is favored and subsequent S_E_2′ protonation is enantio-determining. Reduced performances were disclosed for all other substitution patterns surveyed. In 2022, Zhang and co-workers reported the 1,4-selective Cu-catalyzed enantioselective protoboration of *linear* dienes using **L3** (Figure 1D).^2c^ Mechanistic investigations pointed to an enantio-determining borocupration followed by a stereoretentive S_E_2′-type protonation. In the same year, the Diver group developed a Cu-catalyzed enantioselective protoboration of *acyclic* 2,4-disubstituted 1,3-dienes accessed by ene-yne metathesis (Figure 1E).^2d^ Using Duphos-type ligands (**L1**), excellent 3,4-regioselectivity was achieved, and *er* values ranging from 82:18 to 92.5:7.5 were measured. Mechanistic investigations were consistent with borocupration being both rate- and enantio-determining and with protonation occurring via an S_E_2-type mechanism. Of note, a handful of Cu-catalyzed enantioselective protoborations using electronically biased conjugated dienes has also been reported (i.e., 1,2-dihydropyridines, [*n*]dendralenes, trifluoromethylated linear 1,3-dienes).^5^

**Figure 1:**
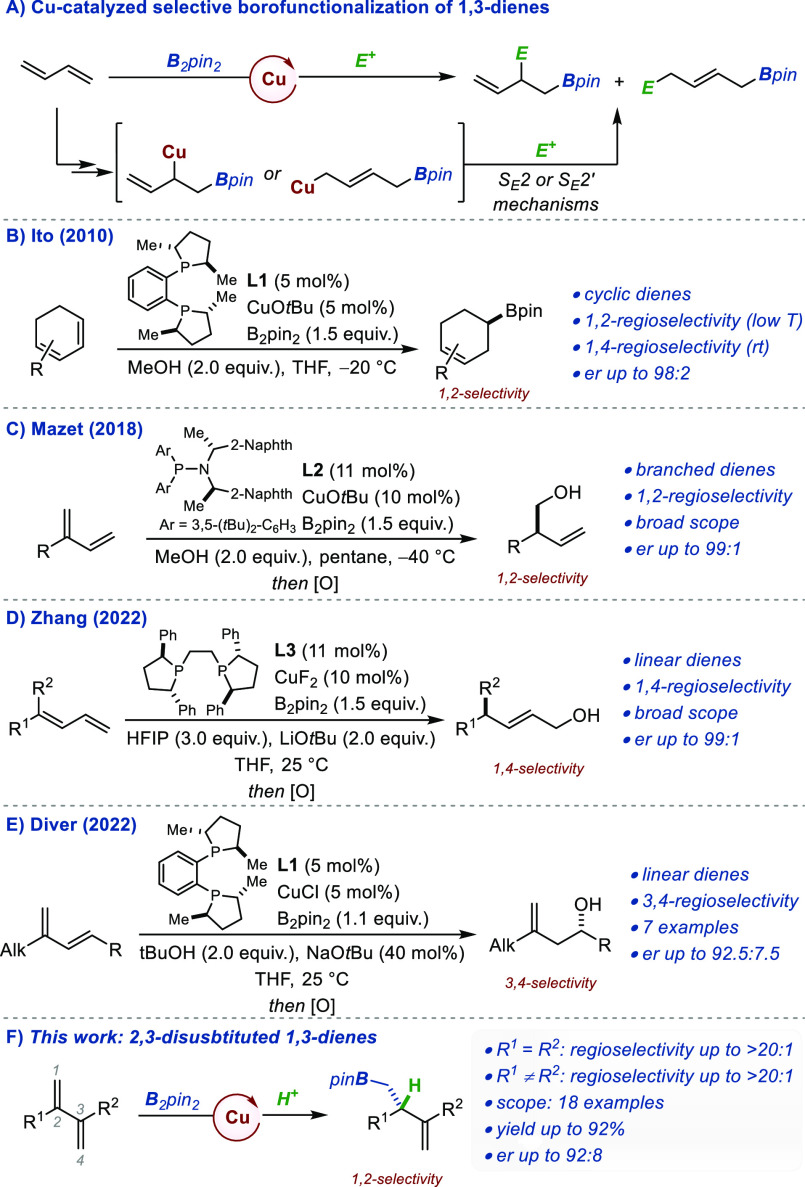
(A) Cu-catalyzed selective borofunctionalization of 1,3-dienes. (B–E) Cu-catalyzed enantioselective protoboration of: (B) cyclic 1,3-dienes; (C) branched 1,3-dienes; (D) linear 1,3-dienes; (E) 2,4-disubstituted 1,3-dienes. (F) This work: Cu-catalyzed enantioselective protoboration of 2,3-disubstituted 1,3-dienes.

To expand the repertoire of borofunctionalizations of nonactivated precursors, we became particularly attracted to the challenge of developing a Cu-catalyzed enantioselective protoboration of acyclic 2,3-disubstituted 1,3-dienes that would be applicable to both symmetrical and unsymmetrical substrates (Figure 1F). We recognized that the successful development of this reaction would provide access to enantioenriched homoallylic boronates that would be difficult to prepare using traditional approaches. We report the results of our progress in this direction, along with preliminary mechanistic insights. We began our study with the survey of standard reaction parameters and the evaluation of several chiral ligands using 2,3-diphenyl-1,3-butadiene **1a** as model substrate.^6^ An overview of these results is presented in Table 1. Additional details are available in the Supporting Information (SI). After an initial round of optimizations, we found that QuinoxP* (**L6**) afforded the homoallylic boronate **2a** in 71% conv. with an excellent regioselectivity (>20:1) and high enantiomeric ratio (*er* 89:11) using B_2_pin_2_ and MeOH as the proton source (entry 1). Ligands **L1**–**L3** which were used by Ito, Mazet, and Zhang for the protoboration of other 1,3-dienes did not provide significant improvements, even though regioselectivity was high in all cases (entries 2–4). Noticeably, this was not the case with Binap (**L4**, 4:1 *rr*, entry 5). Several other privileged bisphosphine ligands (including **L5**, entry 6) were surveyed, but none could compare with **L6**. Introduction of large silyl substituents on the 5 and 8 positions of the quinoxaline core had only marginal impact on the reaction outcome (**L7**, entry 7).^7^ Much reduced catalytic activity and enantiomeric ratio were obtained with **L8**, a *C*_1_-symmetric analogue of **L6** (entry 8). Representative results of the influence of the solvent, the temperature, the copper precursor, and the proton source are disclosed in entries 9–14. Finally, using BenzP* (**L9**),^8^ we found that both the reactivity and *er* could be slightly improved without impairing the regioselectivity (entry 15).

**Table 1:**
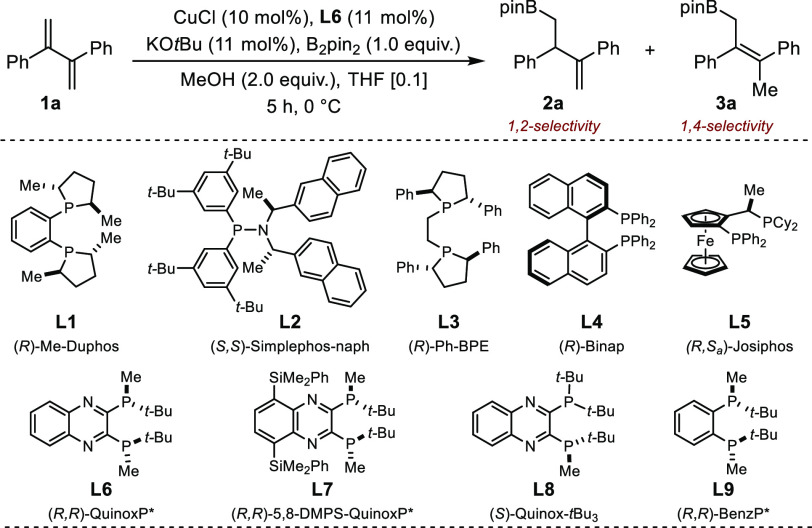
Reaction Optimization^a^

Entry	Variation from optimized conditions	Conv. (%)^b^	***rr*_2a/3a_**	*er***2a**^c^
1	none	71	>20:1	89:11
2^d^^,^^e^	**L1** instead of **L6**	75	>20:1	68:32
3	**L2** instead of **L6**	<5	*nd*	*nd*
4^d^^,^^e^	**L3** instead of **L6**	68	>20:1	77:23
5^d^^,^^e^	**L4** instead of **L6**	39	4:1	73:27
6^d^	**L5** instead of **L6**	54	>20:1	82:18
7	**L7** instead of **L6**	83	>20:1	88:12
8	**L8** instead of **L6**	40	>20:1	64:36
9	Pentane instead of THF	36	>20:1	59:41
10	Toluene instead of THF	89	>20:1	72:28
11^f^	25 °C instead of 0 °C	71	>20:1	88:12
12^g^	–40 °C instead of 0 °C	47	>20:1	80:20
13	CuOAc instead of CuCl	18	>20:1	90:10
14	*i*PrOH instead of MeOH	64	>20:1	90:10
15	**L9** instead of **L6**	83	>20:1	93:7

a Reaction conditions: **1a** (0.2 mmol), B_2_pin_2_ (0.2 mmol).

b Determined by ^1^H NMR using an internal standard.

c Determined by HPLC after oxidation to **2′a**.

d Using CuO*t*Bu (5 mol %) instead of CuCl/KO*t*Bu.

e B_2_pin_2_ (2.0 equiv).

f 1 h.

g 48 h.

This last set of conditions was employed to delineate the scope of symmetrically 2,3-disubstituted 1,3-dienes. For ease of purification and analysis, the boronic esters obtained were converted to the corresponding alcohols by oxidation (Figure 2). Variations of the electronic nature of the aryl groups using ether, alkyl, various halides, or trifluoromethyl substituents did not affect the catalytic performances in terms of activity, regiocontrol, or enantiocontrol (**2′a**–**2′i**). A diminished yield was obtained for ortho-substituted derivatives (**2′h**). While a primary alkyl substituent was found to be compatible, no reaction was observed for a cyclohexyl-derived substrate (**2′j**–**2′k**).

**Figure 2:**
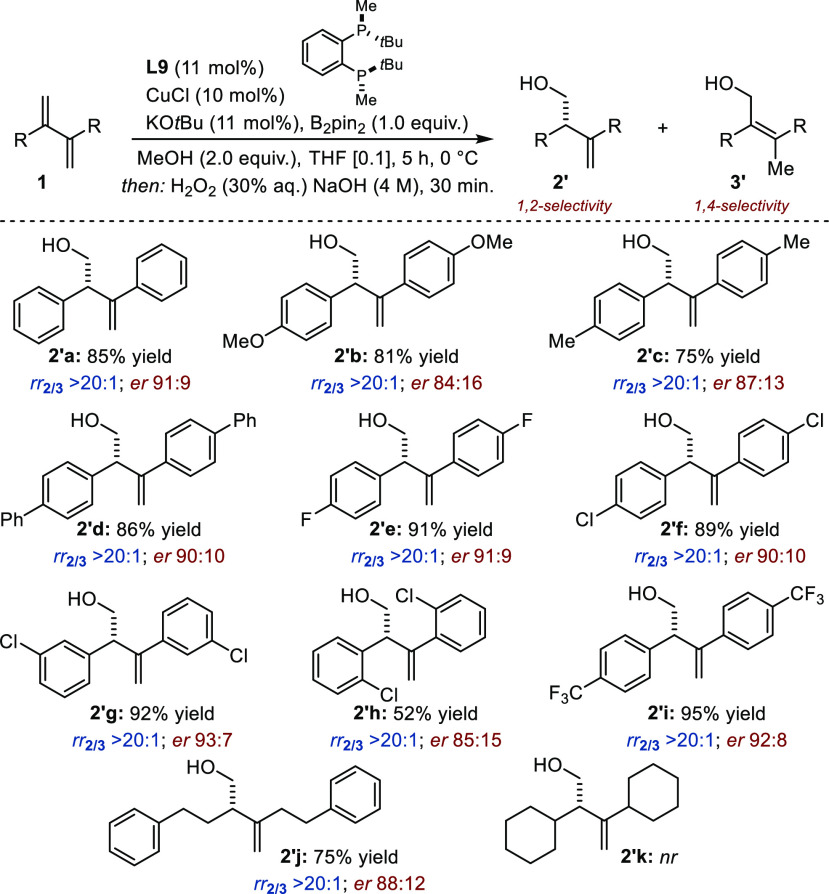
Sequential protoboration/oxidation of symmetrical 2,3-disubstituted 1,3-dienes. Reaction conditions: **1a**–**k** (0.5 mmol), B_2_pin_2_ (0.5 mmol). Regioselectivity assessed by ^1^H NMR before oxidation. Yield after oxidation to **2′**. *Er* determined after oxidation by HPLC. Absolute configuration determined by analogy with a known compound.^9^

We next evaluated unsymmetrical 2,3-disubstituted 1,3-dienes (Figure 3). With **1l**, a compound characterized by two aryl groups with markedly different electronic properties (*p*-CF_3_ vs *p*-OMe), the product of formal 1,2-protoboration **2l** was formed preferentially in 89:11 *er*. While no traces of 4,3-protoboration were observed (*rr*_2/4_ > 20:1), a significant amount of **3l** was detected (*rr*_2/3_ 1.5:1). Noticeably, this regioisomer was not observed for all the other substrates and reaction occurred systematically at the most electron-deficient C=C bond of the diene. Using **1m**, homoallylic alcohol **2′m** was obtained in 92% yield with *rr*_2/4_ 13:1 and a similar *er* (92:8). The extent of regiocontrol decreased for a substrate with two electron-rich aryl groups (**1n**: *p*-Me, *p*-OMe; *rr*_2/4_ 1.5:1). The two inseparable regioisomers were both obtained with high levels of enantiocontrol (*er*_2_ 88:12; *er*_4_ 91:9). The experimental protocol was found to be applicable to substrates combining an aryl substituent and a primary alkyl substituent (**1o**–**1r**). The yield and regioselectivity were high in all cases, and the enantiomeric ratio ranged from 74:26 to 88:12. The yield was impaired by the presence of a secondary alkyl residue (R^2^ = cyclohexyl), but the regioselectivity and the enantiomeric ratio remained high (**2′s**: *er*_2_ 91:9). No reaction occurred with the adamantyl derivative **1t**.

**Figure 3:**
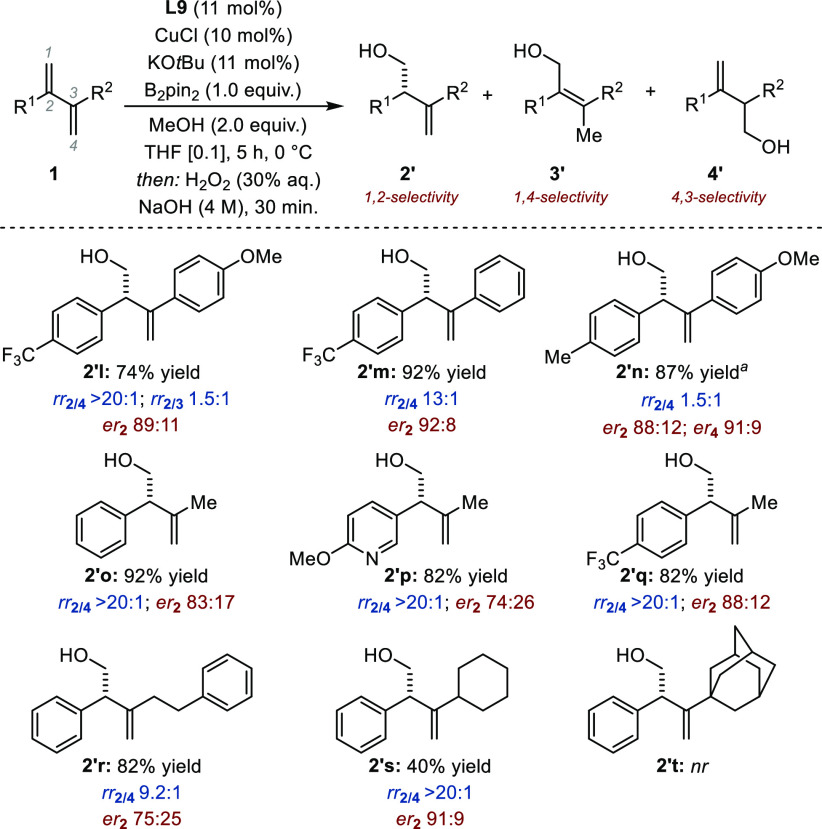
Sequential protoboration/oxidation of unsymmetrical 2,3-disubstituted 1,3-dienes. Reaction conditions: **1l**–**t** (0.5 mmol), B_2_pin_2_ (0.5 mmol). Regioselectivity assessed by ^1^H NMR before oxidation. Yield after oxidation to **2′**. *Er* was determined after oxidation by HPLC. Absolute configuration was determined by analogy with a known compound.^9^^*a*^Combined yield.

When the robustness of the reaction was evaluated by performing the Cu-catalyzed protoboration using 1.10 g of diene **1d**, homoallylic boronate **2d** was isolated as the sole regioisomer in 91% yield (1.36 g) and 92:8 *er*. We showed that the enantiopurity of the protoboration product could be further increased to >99:1 *er* by a single recrystallization using a 10:1 MeOH/CHCl_3_ solvent mixture (Figure 4).

**Figure 4:**
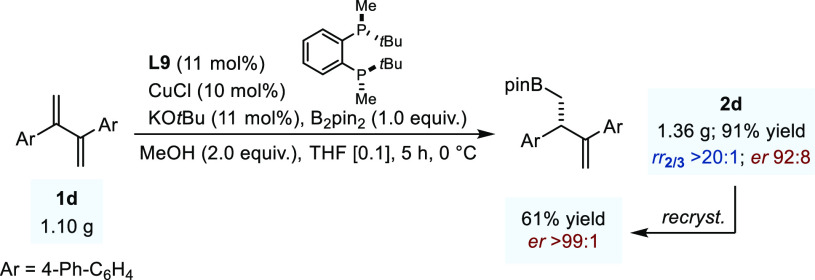
Large scale experiment using **1d**. *Er* was measured by HPLC after oxidation. Recrystallization was performed using MeOH/CHCl_3_ (10:1).

A series of complementary experiments was conducted to gather preliminary insights into the mechanism of the catalytic reaction (Figure 5). We first measured the initial rates of protium and deuterium incorporation in parallel experiments run in separate vessels using MeOH and MeOD (Figure 5A).^10^

**Figure 5:**
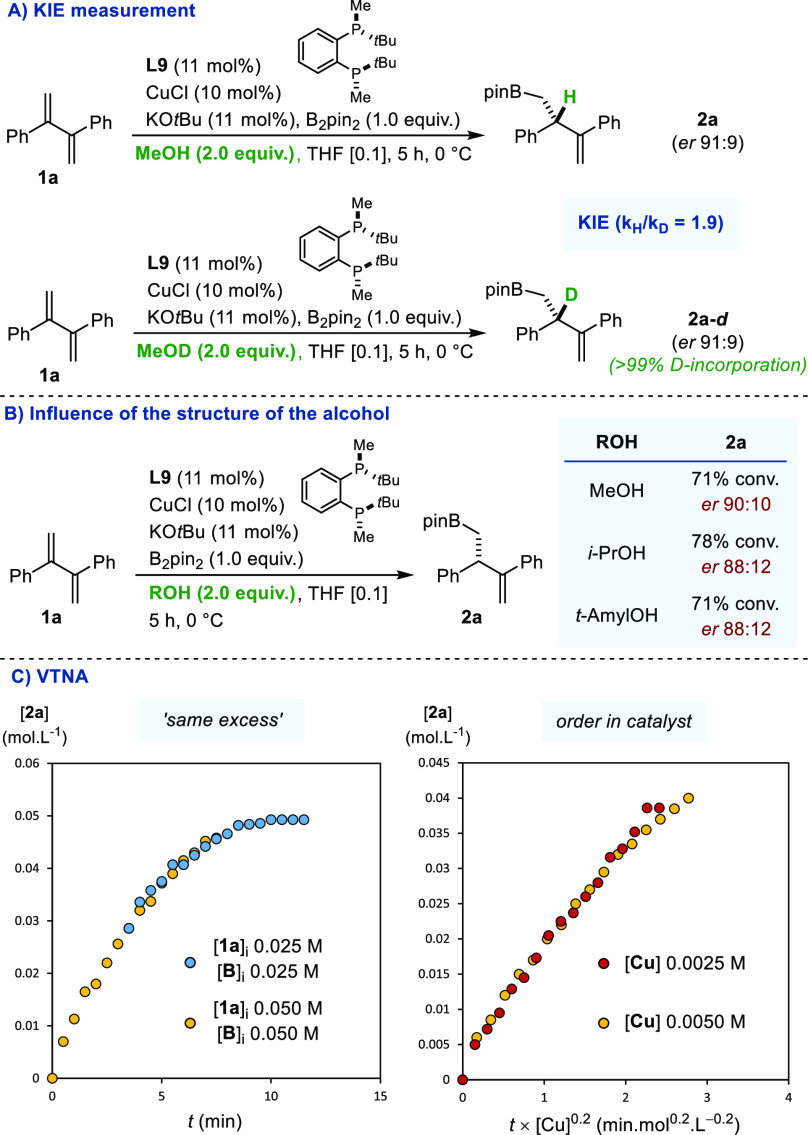
(A) Kinetic isotope effect. (B) Influence of the structure of the protonation agent. (C) Variable time normalization analyses. All data are the average of at least two experiments. *Er* was measured by HPLC.

Under the standard reaction conditions, a primary kinetic isotope effect was measured (KIE = 1.9), a result consistent with protonation being the rate-determining step (RDS). Quite noticeably, the *er* values were identical in both experiments. In line with this observation, we did not observe any significant variation in *er* using sterically more demanding alcohols (Figure 5B). Taken together, these data suggest that borocupration rather than protonation is likely to be the enantio-determining step. The model reaction was conducted using scalemic mixtures with different proportions of the two enantiomers of ligand **L9**.^11^ The homogeneity of the reaction was checked to avoid a false positive.^11e^ The plot of the *ee* of product **2a** as a function of the *ee* of chiral ligand **L9** showed both positive and negative nonlinear effects (NLE), as observed in some rare occasions for other Cu-catalyzed processes (see SI).^11,12^ Using the method developed by the Burés group, variable time normalization analyses (VTNA) were performed by monitoring formation of **2a** by ^1^H NMR (Figure 5D).^13^ The graphical overlay obtained for the “same excess” experiment indicates that there is neither catalyst deactivation nor product inhibition over the course of the reaction. An unusual partial order of 0.2 in [Cu] was probed by means of a “different excess” experiment. Coupled with the observation of NLE, this finding reveals catalyst speciation and that oligomeric copper complexes probably directly participate in the prevailing productive catalytic cycle. These observations are reminiscent of those reported by Blackmond and co-workers in the context of Pd-catalyzed enantioselective C(sp^3^)–H bond arylations.^14^

A tentative catalytic cycle consistent with the results of our mechanistic investigations is disclosed in Figure 6. Activation of B_2_pin_2_ by a copper-alkoxo complex followed by diene coordination (**I** → **II** → **III**) is well documented and is expected to proceed smoothly.^1,7,15^ Collectively, our experimental data are in agreement with the subsequent borocupration (**III** → **IV**) being the enantio-determining step. The tertiary Cu-alkyl intermediate generated undergoes rate-determining S_E_2 protonation to liberate the product and regenerate the starting copper-alkoxo complex (**IV** → **I**). The tertiary allylcopper complex (**IV**) is potentially in equilibrium with the primary allylcopper (**V**) via a σ–π–σ mechanism. Because protonation is not enantio-determining, formation of **2** from **V** is unlikely to occur. The product of formal 1,4-protoboration (**3**) could be accessed either by S_E_2′ protonation of **IV** or by S_E_2 protonation of **V**. The high levels of regiocontrol observed under the optimized conditions suggest either that protonation is slow or that **V** is kinetically not accessible. Finally, the results of the NLE study and our kinetic measurements indicate that a composite catalytic system arising from catalyst speciation is likely operating. Even though the exact nature of the copper complex remains unclear, formation of dimeric μ-boryl-bridged copper complexes from a mononuclear intermediate similar to **II** is thermodynamically favorable (using **L6** instead of **L9**).^7a,16^ Overall, the exact composition of the mixture of catalytically active intermediates is unclear and whether these numerous species lie on or off-cycle is difficult to assess.^11,14^ Finally, our data also point to the fact that product coordination is not inhibitory, and displacement by binding of an incoming molecule of substrate to regenerate **III** should be favorable.

**Figure 6:**
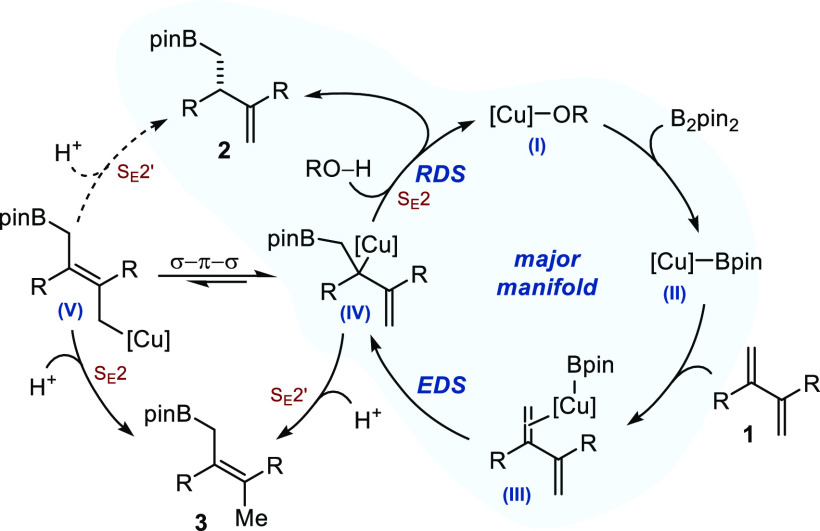
Proposed catalytic cycle.

In conclusion, we have developed a regio- and enantioselective Cu-catalyzed protoboration of 2,3-disubstituted 1,3-dienes, which provides access to chiral homoallylic alcohols with high levels of selectivity. The method operates under mild conditions and is applicable to symmetrical and unsymmetrical derivatives. Preliminary mechanistic investigations have shed light on unusual features indicative of catalyst speciation and the formation of catalytically competent oligomeric copper species. Further studies are underway in our laboratories to understand the intricacies of this mechanism, which may well extend to other Cu-catalyzed selective borofunctionalization reactions.

## Data Availability

The data underlying this study are available in the published article and its Supporting Information.

## Supporting Information Available

Experimental procedures, characterization of all new compounds, and NMR spectra. (PDF)

## References

[ref1] aSemba K.; Fujihara T.; Terao J.; Tsuji Y. Copper-Catalyzed Borylative Transformations of Non-Polar Carbon–Carbon Unsaturated Compounds Employing Borylcopper as an Active Catalyst Species. Tetrahedron 2015, 71, 2183–2197. 10.1016/j.tet.2015.02.027.

[ref2] aSasaki Y.; Zhong C.; Sawamura M.; Ito H. Copper (I)-Catalyzed Asymmetric Monoborylation of 1,3-Dienes: Synthesis of Enantioenriched Cyclic Homoallyl-and Allylboronates. J. Am. Chem. Soc. 2010, 132, 1226–1227. 10.1021/ja909640b. 20063883

[ref3] aFehr C. Enantioselective Protonation of Enolates and Enols. Angew. Chem., Int. Ed. 1996, 35, 2566–2587. 10.1002/anie.199625661.

[ref4] aPalais L.; Mikhel I. S.; Bournaud C.; Micouin L.; Falciola C. A.; Augustin M. V.; Rosset S.; Bernardinelli G.; Alexakis A. SimplePhos Monodentate Ligands: Synthesis and Application in Copper-Catalyzed Reactions. Angew. Chem., Int. Ed. 2007, 119, 7606–7609. 10.1002/ange.200702186. 17702075

[ref5] aJarava-Barrera C.; Parra A.; López A.; Cruz-Acosta F.; Collado-Sanz D.; Cárdenas D. J.; Tortosa M. Copper-Catalyzed Borylative Aromatization of *p*-Quinone Methides: Enantioselective Synthesis of Dibenzylic Boronates. ACS Catal. 2016, 6, 442–446. 10.1021/acscatal.5b02742. 27088045 PMC4831668

[ref6] See Supporting Information for substrates syntheses.

[ref7] aIwamoto H.; Ozawa Y.; Takenouchi Y.; Imamoto T.; Ito H. Backbone-Modified *C*_2_-Symmetrical Chiral Bisphosphine TMS-QuinoxP*: Asymmetric Borylation of Racemic Allyl Electrophiles. J. Am. Chem. Soc. 2021, 143, 6413–6422. 10.1021/jacs.0c08899. 33891398

[ref8] Imamoto T. Searching for Practically Useful P-Chirogenic Phosphine Ligands. Chem. Rec. 2016, 16, 2659–2673. 10.1002/tcr.201600098. 27524808

[ref9] Chen Y.-L.; Hoppe D. Copper-Catalyzed Asymmetric Conjugate Addition of Grignard Reagents to 1-(*N*,*N*-Diisopropylcarbamoyloxy)-1-tosyl-1-alkenes. Tetrahedron: Asymmetry 2009, 20, 1561–1567. 10.1016/j.tetasy.2009.06.001.

[ref10] aAnslyn E. V.; Dougherty D. A. In Modern Physical Organic Chemistry; University Science Books: Sausalito, CA, 2006.

[ref11] aKagan H. B. In Comprehensive Asymmetric Catalysis; Jacobsen E. N., Pfaltz A., Yamamoto H., Eds.; Springer: Berlin, 1999, Chapter 4.

[ref12] aTanaka K.; Matsui J.; Kawabata Y.; Suzuki H.; Watanabe A. Chiral Amplification in the Synthesis of (*R*)-Muscone by Conjugate Addition of Chiral Alkoxydimethylcuprate to (*E*)-Cyclopentadec-2-enone. J. Chem. Soc. Chem. Commun. 1991, 1632–1634. 10.1039/c39910001632.

[ref13] aBurés J. A Simple Graphical Method to Determine the Order in Catalyst. Angew. Chem., Int. Ed. 2016, 55, 2028–2031. 10.1002/anie.201508983. PMC4797368 26749539

[ref14] Hill D. E.; Pei Q.-I.; Zhang E.-X.; Gage J. R.; Yu J.-Q.; Blackmond D. G. A General Protocol for Addressing Speciation of the Active Catalyst Applied to Ligand-Accelerated Enantioselective C(sp^3^)–H Bond Arylation. ACS Catal. 2018, 8, 1528–1531. 10.1021/acscatal.8b00103.

[ref15] aLaitar D. S.; Tsui E. Y.; Sadighi J. P. Copper(I) β-Boroalkyls from Alkene Insertion: Isolation and Rearrangement. Organometallics 2006, 25, 2405–2408. 10.1021/om060131u.

[ref16] aBorner C.; Anders L.; Brandhorst K.; Kleeberg C. Elusive Phosphine Copper (I) Boryl Complexes: Synthesis, Structures, and Reactivity. Organometallics 2017, 36, 4687–4690. 10.1021/acs.organomet.7b00775.

