# Supplementary material for: Cu-Catalyzed Enantioselective Protoboration of 2,3-Disubstituted 1,3-Dienes
Source: Org Lett. 2023 Sep 11;25(37):6897–901. doi: 10.1021/acs.orglett.3c02627 (PMC10521025; doi:10.1021/acs.orglett.3c02627)

Cu-Catalyzed Enantioselective Protoboration of 2,3-Disubstituted 1,3-Dienes

Sensheng Liu, Yangbin Liu, Arthur Flaget, Cheng Zhang and Clément Mazet*

Department of Organic Chemistry, University of Geneva,
30 quai Ernest Ansermet, 1211 Geneva
Switzerland
clement.mazet@unige.ch

Table of Contents

1. General information 2

2. Reaction optimization 3

3. General procedure for the synthesis of unsymmetrical 2,3-disubstituted 1,3-dienes 9

4. General procedure for the protoboration/oxidation sequence 13

5. Large scale experiment 50

6. Mechanistic study 51

6.1 Kinetic Isotope Effect 51

6.2 Non-Linear Effect 53

6.3 Variable Time Normalization Analyses 54

7. References 56

8. NMR Spectra 57

9. ^{1}H NMR spectra of known compounds 127

1. General information

Unless otherwise noted, all reactions were carried out under nitrogen using either two-manifold vacuum/inert gas lines or a M.Braun glove box. Solvents were dried over activated alumina columns and further degassed by three successive "freeze-pump-thaw" cycles.

NMR spectrometers. NMR spectra were acquired at the University of Geneva NMR platform (https://www.unige.ch/sciences/chiorg/nmr/) using a 500 MHz Avance III Bruker NMR spectrometer equipped with a helium-cooled cryogenic 5-mm DCH ^{13}C-^{1}H/D Bruker probe, a 400 MHz Avance III HD NanoBay spectrometer equipped with a N_2 prodigy cryogenic 5 mm CPP BB(F)-H-D probe or a 300 MHz Avance III, HD NanoBay spectrometer, equipped with a 5 mm PA BBO, BB(F)-H-D probe. All ^{1}H and $^{13}C\{^{1}H\}$ experiments were internally referenced with respect to $CDCl_3$ solvent signals and acquired at 298 K. $^{31}P\{^{1}H\}$ and $^{19}F\{^{1}H\}$ NMR chemical shifts are reported in ppm with reference to the lock.

Infrared spectra were obtained on a Perkin−Elmer 1650 FT-IR spectrometer using neat samples on a diamond ATR Golden Gate sampler. The mass spectrometric data were obtained at the mass spectrometry facility of the University of Geneva (http://www.unige.ch/sciences/sms/). GC-MS analyses were performed on GC–HP 6890, column Agilent–HP1 (30 m–ID 0.32 mm, Film 0.25 µm) coupled with MS–HP 5973.

The enantiomeric ratios (*er*) were determined by HPLC analyses. HPLC analyses were performed on a *Shimadzu* CTO-20AA equipped with DAICEL columns (chiral phases: OD-H, OJ-H, AD-H, OZ-H and IC). Retention times (t) are given in minutes.

Thin layer chromatography (TLC) was performed on plates of silica pre-coated with 0.25 mm Kieselgel 60 F_{254} from *Merck*. Flash chromatography was performed using silica gel SiliaFlash® P60 (230-400 mesh) from *Silicycle*. Commercial reagents, precatalysts and ligands were purchased from Fluka, Acros or Strem and used without purification unless otherwise noted. Liquid reagents were transferred with stainless steel syringes or cannula. Copper precatalysts, as well as ligands were stored and weighted inside a M.Braun glove box.

Symmetrical 2,3-disubstituted 1,3-dienes were prepared following reports from the literature.[1]

2. Reaction optimization

Table S1: Ligand survey

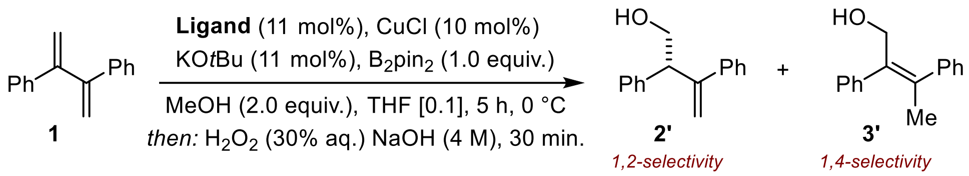

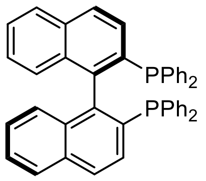

(*R*)-BINAP
39% conv.
rr$_{2a/3a}$ 4:1; *er* 73:27

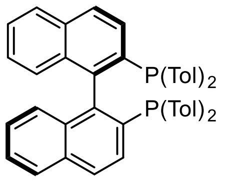

(*R*)-tol-BINAP
20% conv.
rr$_{2a/3a}$ >20:1; *er* 78:22

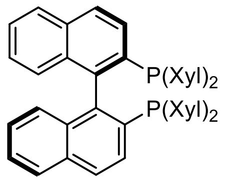

(*R*)-xyl-BINAP
34% conv.
rr$_{2a/3a}$ >20:1;*er* 90:10

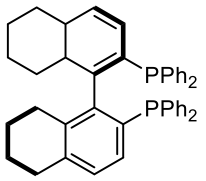

(*R*)-H$_8$-BINAP
<5% conv.
nd

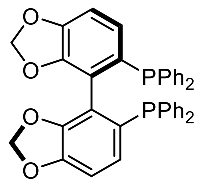

(*R*)-Segphos
39% conv.
rr$_{\mathbf{2a/3a}}$ >20:1; *er* 73:27

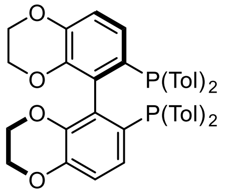

(*R*)-tol-Synphos
<5% conv.
nd

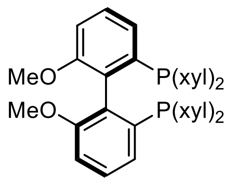

(*R*)-xyl-Biphep
15% conv.
rr$_{2a/3a}$ >20:1; *er* 90:10

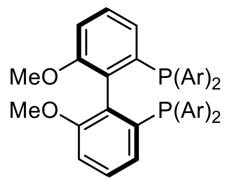

(*S*)-DTBM-Biphep
<5% conv.
nd

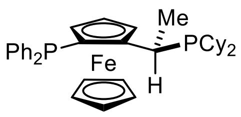

(*R*)-(*Sp*)-Josiphos
54% conv.
rr$_{\mathbf{2a/3a}}$ >20:1; *er* 82:18

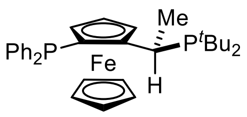

(*R*)-Josi-1
<5% conv.
nd

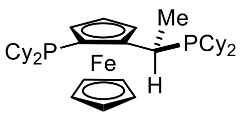

(*R*)-Josi-2
<5% conv.
nd

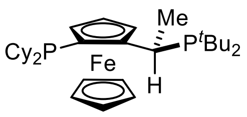

(*R*)-Josi-3
<5% conv.
nd

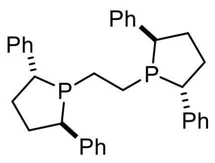

(*R,R*)-Ph-BEPs
68% conv.
rr$_{2a/3a}$ >20:1; *er* 77:23

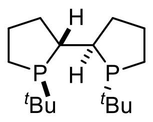

(*S,S,R,R*)-Tangphos
<5% conv.
nd

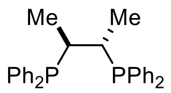

(*S,S*)-Chiraphos
<5% conv.
nd

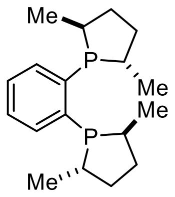

(R)-Me-Duphos
75% conv.
rr$_{2a/3a}$ >20:1; *er* 68:32

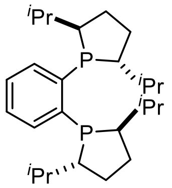

(*R*)-*i*Pr-Duphos
23% conv.
rr$_{2a/3a}$ >20:1; er 76:24

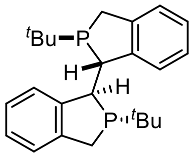

(*R*)-Duanphos
41% conv.
rr$_{2a/3a}$ >20:1; er 81:19

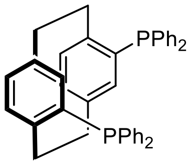

(*S*)-Phanephos
<5% conv.
nd; nd

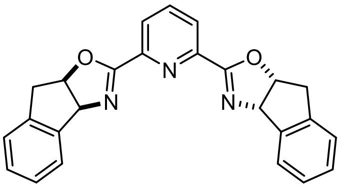

Indenyl-Pybox
<5% conv.
nd; nd

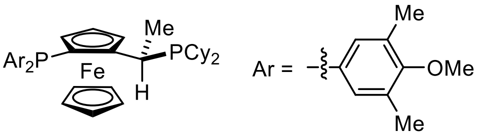

(*R*)-Josi-4
81% conv.
rr$_{2a/3a}$ 10:1; er 83:17

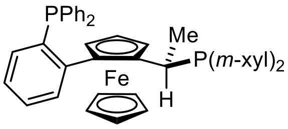

(*R*)-(*Rp*)-Walphos
<5% conv.
nd; nd

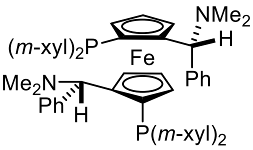

(*R*)-(*Sp*)-Mandyphos
81% conv.
rr$_{2a/3a}$ 10:1; er 90:10

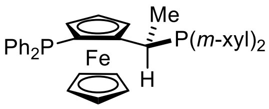

(*R*)-Josi-5
81% conv.
rr$_{2a/3a}$ 10:1; er 71:29

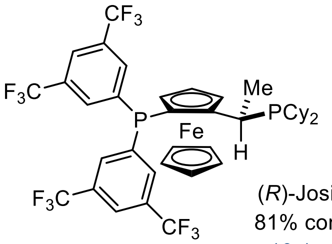

(*R*)-Josi-6
81% conv.
rr$_{2a/3a}$ 10:1; er 74:26

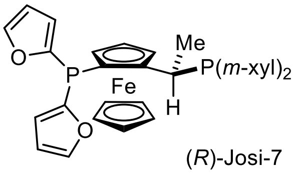

(*R*)-Josi-7
<5% conv.
nd; nd

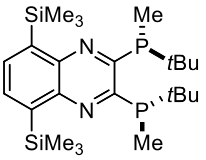

(*S*,*S*)-5,8-TMS-QuinoxP*
81% conv.
rr$_{2a/3a}$ >20:1; *er* 90:10

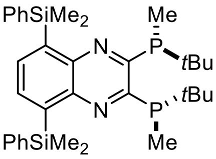

(*S*,*S*)-5,8-DMPS-QuinoxP*
83% conv.
rr$_{2a/3a}$ >20:1; *er* 88:12

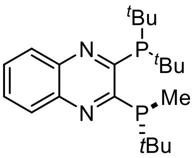

(*S*)-Quinox-*t*Bu$_3$
40% conv.
rr$_{2a/3a}$ >20:1; *er* 64:36

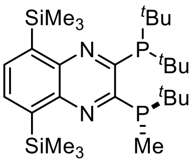

(*S*)-5,8-TMS-3H
40% conv.
rr$_{2a/3a}$ >20:1; *er* 64:36

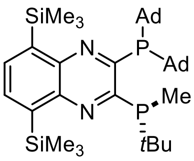

(*S*)-5,8-TMS-Ad-QuinoxP*
nr

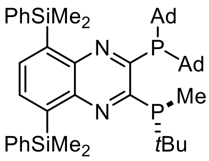

(*S*)-5,8-DMPS-Ad-QuinoxP*
nr

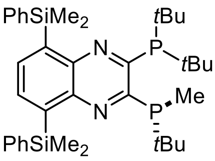

(*S*)-5,8-DMPS-3H-QuinoxP*
32% conv.
rr$_{2a/3a}$ >20:1; *er* 70:30

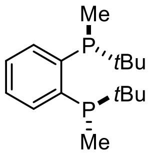

(*R,R*)-BenzP*
83% conv.
rr$_{2a/3a}$ >20:1; *er* 93:7

[a] Reaction conditions: Unless otherwise noted, all reactions were performed with diene (0.2 mmol), ligand/CuCl (1.1:1, 10 mol%), B_2pin_2 (0.1 mmol), KO*t*Bu (40 mol%), MeOH (0.2 mmol) in THF (2 mL) at 0 °C. Conversion and regioselectivity are from the boronic ester detected by ^{1}H NMR. Enantioselectivity determined by HPLC analysis on a chiral stationary phase after oxidation to the alcohol.

Table S2: Solvent survey using **L1**[a]

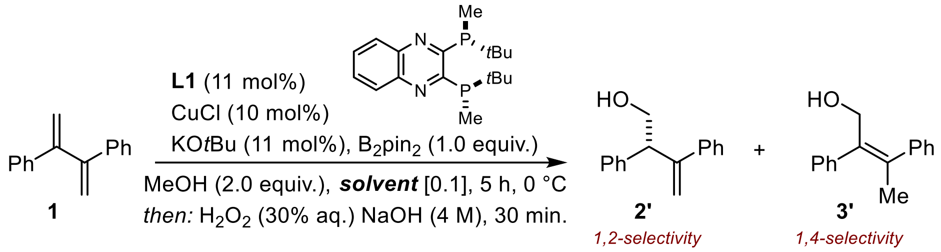

Entry	Solvent	conv. **2a** (%)	*er* (**2a**)
1	2-Me-THF	89	89:11
2	Et_2O	86	85:15
3	MTBE	87	87:13
4[b]	1,4-Dioxane	87	90:10
5	Pentane	36	59:41
6	Toluene	89	72:28

[a]Buta-1,3-diene-2,3-diyldibenzene (0.2 mmol), CuCl (10 mol%), (*R*,*R*)-QuinoxP* (11 mol%), KO*t*Bu (10 mol%, 1 M in THF), B_2pin_2 (0.2 mmol, 1.0 equiv.), MeOH (0.4 mmol, 2.0 equiv.), Solvent (2 mL), at 0 °C for 5 h. Conversion and regioselectivity are from the boronic ester detected by ^{1}H NMR. Enantioselectivity determined by HPLC analysis on a chiral stationary phase after oxidation to the alcohol. [b]At room temperature.

Table S3: Base variations using **L1**[a]

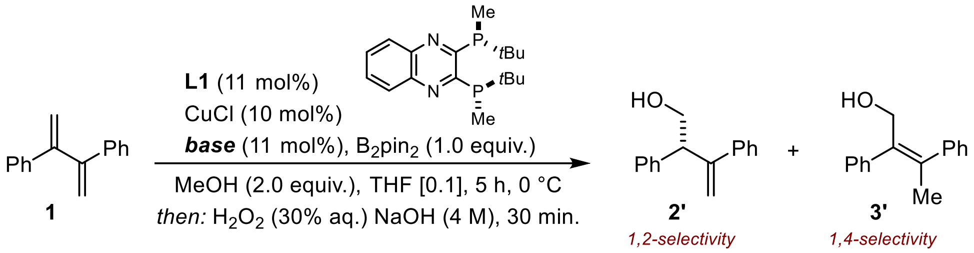

Entry	Base	conv. **2a** (%)	*er* (**2a**)
1	LiO*t*Bu	<5	*nd*
2	NaO*t*Bu	58	90:10
3	KO*t*Bu	71	89:11

[a]Buta-1,3-diene-2,3-diyldibenzene (0.2 mmol), CuCl (10 mol%), (*R*,*R*)-QuinoxP* (11 mol%), base (10 mol%, 1 M in THF), B_2pin_2 (0.2 mmol, 1.0 equiv.), MeOH (0.4 mmol, 2.0 equiv.), THF (2 mL), at 0 °C for 5 h. Conversion and regioselectivity are from the boronic ester detected by ^{1}H NMR. Enantioselectivity determined by HPLC analysis on a chiral stationary phase after oxidation to the alcohol.

Table S4: Influence of the structure of the proton source using **L1**[a]

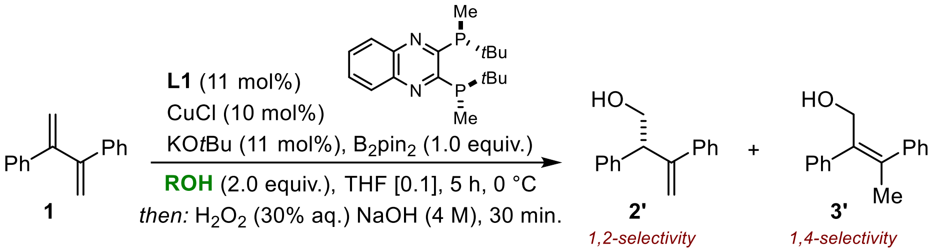

Entry	Alcohol	conv. **2a** (%)	*er* (**2a**)
1	TFE	<5	*nd*
2	*i*-PrOH	64	90:10
3	*t*-AmylOH	38	90:10
4	Morpholinium.TFA	<5	*nd*

[a]Buta-1,3-diene-2,3-diyldibenzene (0.2 mmol), CuCl (10 mol%), (*R*,*R*)-QuinoxP* (11 mol%), KO*t*Bu (10 mol%, 1 M in THF), B_2pin_2 (0.2 mmol, 1.0 equiv.), ROH (0.4 mmol, 2.0 equiv.), THF(2 mL), at 0 ˚C for 5 h. Conversion and regioselectivity are from the boronic ester detected by ^{1}H NMR. Enantioselectivity determined by HPLC analysis on a chiral stationary phase after oxidation to the alcohol.

Table S5: Copper source optimization using **L1**[a]

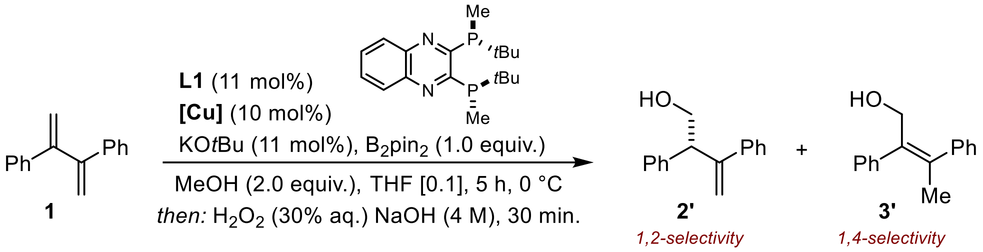

Entry	[Cu]	conv. **2a** (%)	*er* (**2a**)
1	CuBr	60	90:10
2	CuI	<5	*nd*
3	CuOAc	18	89:11
4	CuCl/NaBAr$_F$	<5	*nd*
5	[Cu(MeCN)$_4$]PF$_6$	61	90:10

[a]Buta-1,3-diene-2,3-diyldibenzene (0.2 mmol), [Cu] (10 mol%), (*R*,*R*)-QuinoxP* (11 mol%), KO*t*Bu (10 mol%, 1 M in THF), B_2pin_2 (0.2 mmol, 1.0 equiv.), MeOH (0.4 mmol, 2.0 equiv.), THF (2 mL), at 0 ˚C for 5 h. Conversion and regioselectivity are from the boronic ester detected by ^{1}H NMR. Enantioselectivity determined by HPLC analysis on a chiral stationary phase after oxidation to the alcohol.

Table S6: Effect of the temperature using (*R*,*R*)-QuinoxP* (**L6**) or (*R*,*R*)-BenzP* (**L9**)[a]

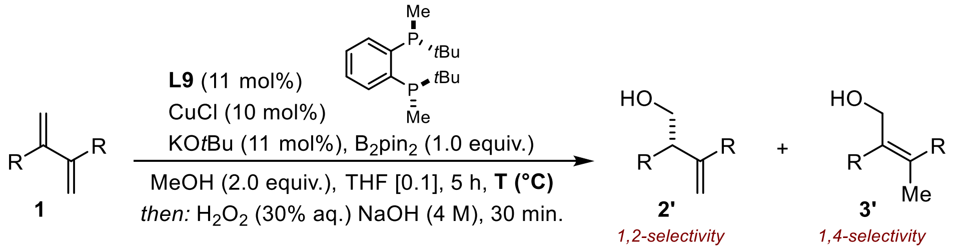

Entry	Ligand	Temp (˚C)	conv. **2a** (%)	*er* (**2a**)
1	**L9**	25	75	90:10
2	**L6**	25	71	87:13
3	**L6**	–10	64	90:10
4	**L6**	–20	61	90:10
5	**L6**	–40	15	80:20
6	**L9**	–40	<5	*nd*
7	**L6**	–60	<5	*nd*

[a]Buta-1,3-diene-2,3-diyldibenzene (0.2 mmol), CuCl (10 mol%), Ligand (11 mol%), KO*t*Bu (10 mol%, 1 M in THF), B_2pin_2 (0.2 mmol, 1.0 equiv.), MeOH (0.4 mmol, 2.0 equiv.), THF (2 mL), at indicated temperature for 5 h. Conversion and regioselectivity are from the boronic ester detected by ^1H NMR. Enantioselectivity determined by HPLC analysis on a chiral stationary phase after oxidation to the alcohol.

Table S7: Influence of the structure of the alcohol using (*R*,*R*)-BenzP* (**L9**)[a]

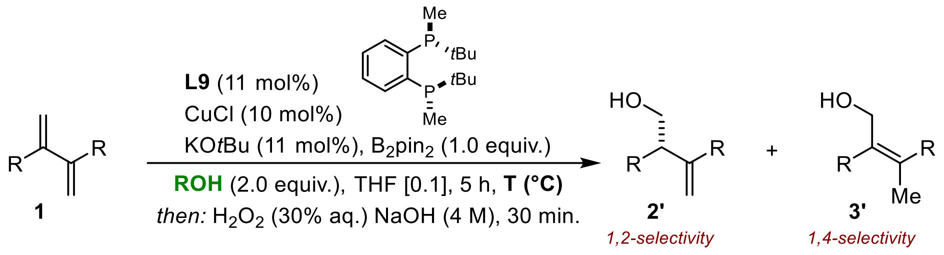

Entry	Alcohol	conv. **2a** (%)	*er* (**2a**)
1	EtOH	71	90:10
2	*i*-PrOH	78	88:12
3	*t*-AmylOH	71	88:12

[a]Buta-1,3-diene-2,3-diyldibenzene (0.2 mmol), CuCl (10 mol%), (*R*,*R*)-BenzP* (11 mol%), KO*t*Bu (10 mol%, 1 M in THF), B_2pin_2 (0.2 mmol, 1.0 equiv.), ROH (0.4 mmol, 2.0 equiv.), THF(2 mL), at 0 ˚C for 5 h. Conversion and regioselectivity are from the boronic ester detected by ^{1}H NMR. Enantioselectivity determined by HPLC analysis on a chiral stationary phase after oxidation to the alcohol.

3. General procedure for the synthesis of unsymmetrical 2,3-disubstituted 1,3-dienes[1b]

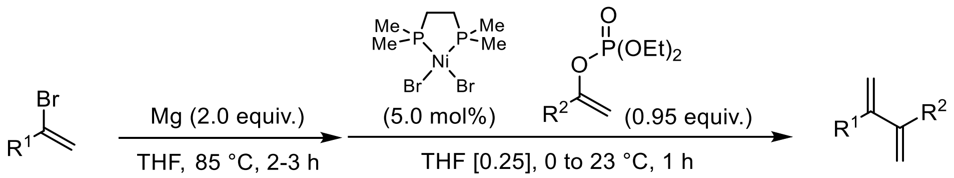

To a 3 mL suspension of Mg (2.0 equiv.) in THF was added the appropriate α-vinyl bromide derivative (1.0 equiv.). The suspension was stirred at 85 °C for 2 to 3 h and then cooled to room temperature. This suspension was added dropwise at 0 °C to a 10 mL Schlenk tube containing [(dmpe)$NiBr_2$] (5 mol%) and the appropriate enol phosphate (0.95 equiv.) dissolved in 5 mL of THF. The mixture was warmed to room temperature once the addition was complete and stirred for one hour later. The reaction was quenched with saturate NH_4Cl (5 mL). The organic layer was separated, and the aqueous phase extracted with ethyl acetate (3×10 mL). The combined organic layers were washed with brine and dried over Na_2SO_4. After removal of the volatiles, the residue was purified by flash chromatography on silica gel (pentane to pentane/Et_2O 98/2) to yield the desired product.

(3,4-dimethylenehexane-1,6-diyl)dibenzene (**1j**)

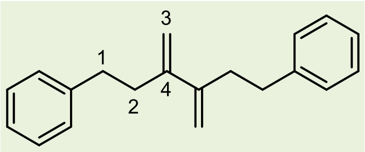

(3-phenylpropyl)magnesium bromide was prepared according to a literature procedure,[1a] using magnesium turnings (365 mg, 15 mmol, 3.0 equiv.), (3-bromopropyl)benzene (2.28 mL, 15 mmol, 3.0 equiv.) in THF, CuI (286 mg, 1.5 mmol, 30 mol%) and tetraethyl hex-3-yne-1,6-diylbis(phosphonate) (1.63 g, 5 mmol, 1.0 equiv.). The reaction mixture was purified by column chromatography to afford the diene as white solid (1.15 g, 88%).

TLC: Rf = 0.5 (pentane).

^{1}H NMR (400 MHz, $CDCl_3$) δ (ppm) 7.30 (dd, $^3J_{HH}$ = 8.2, 6.9 Hz, 4H), 7.21 (d, $^3J_{HH}$ = 7.4 Hz, 6H), 5.18 (s, 2H), 5.01 (s, 2H), 2.85 – 2.73 (m, 4H), 2.64 – 2.53 (m, 4H).

^{13}C{^{1}H} NMR (101 MHz, $CDCl_3$) δ (ppm) = 146.9, 142.4, 128.5, 128.5, 126.0, 112.4, 36.3, 35.3.

LRMS (ESI +): calculated for $C_{20}H_{22}$ $[M+H]^+$: 263.2; found: 263.5.

IR (neat) ν (cm^{-1}): 3100, 3027, 2901, 2857, 1597, 1427, 1073, 895, 743, 696, 640, 608.

m.p.: 35.6 °C

1-methoxy-4-(3-(4-(trifluoromethyl)phenyl)buta-1,3-dien-2-yl)benzene (**1l**)

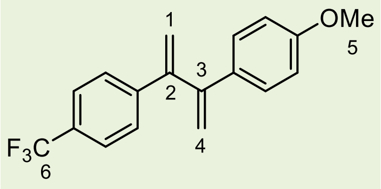

Following the general procedure using 1-(1-bromovinyl)-4-methoxybenzene (270 mg, 1.27 mmol, 1.0 equiv.), Mg (61.6 mg, 2.53 mmol, 2.0 equiv.), diethyl (1-(4-(trifluoromethyl)phenyl)vinyl) phosphate (390 mg, 1.2 mmol, 0.95 equiv.), [(dmpe)$NiBr_2$] (23.2 mg, 5 mol%). The reaction mixture was purified by column chromatography to afford the diene as white solid (230 mg, 60% yield).

TLC: Rf = 0.4 (pentane).

^{1}H NMR (400 MHz, $CDCl_3$) δ (ppm) 7.55 – 7.42 (m, 4H), 7.33 – 7.20 (m, 2H), 6.83 – 6.75 (m, 2H), 5.59 (d, $^3J_{HH}$ = 1.5 Hz, 1H), 5.50 (d, $^3J_{HH}$ = 1.6 Hz, 1H), 5.42 (d, $^3J_{HH}$ = 1.5 Hz, 1H), 5.23 (d, $^3J_{HH}$ = 1.6 Hz, 1H), 3.75 (s, 3H).

^{13}C{^{1}H} NMR (75 MHz, $CDCl_3$) δ (ppm) = 159.4, 149.1, 148.7, 143.9, 132.2, 128.7, 127.8, 125.3, 117.9, 115.4, 113.8, 55.4.

^{19}F{^{1}H} NMR ($CDCl_3$, 282 MHz) δ (ppm) = –62.5 (s).

HRMS (ESI +): calculated for $C_{18}H_{15}F_3O$ $[M+H]^+$: 305.1148; found: 305.1128.

IR (neat) ν (cm^{-1}): 3045, 1926, 1607, 1510, 1288, 1168, 1081, 919, 835, 747, 693, 681, 622.

M.P: 33.8-35.1 °C

1-(3-phenylbuta-1,3-dien-2-yl)-4-(trifluoromethyl)benzene (1m)

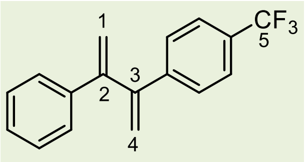

Following the general procedure using (1-bromovinyl)benzene (485 mg, 2.5 mmol, 1.0 equiv.), Mg (122 mg, 5.0 mmol, 2.0 equiv.), diethyl (1-(4-(trifluoromethyl)phenyl)vinyl) phosphate (770 mg, 238 mmol, 0.95 equiv.), [(dmpe)NiBr$_2$] (32 mg, 5 mol%). The reaction mixture was purified by column chromatography to afford the diene as colorless oil (300 mg, 44% yield).

TLC: Rf = 0.56 (pentane/Et_2O = 50:1).

^{1}H NMR (400 MHz, $CDCl_3$) δ (ppm) 7.54 – 7.46 (m, 4H), 7.39 – 7.34 (m, 2H), 7.31 – 7.22 (m, 3H), 5.60 (d, $^3J_{HH}$ = 1.4 Hz, 1H), 5.57 (d, $^3J_{HH}$ = 1.6 Hz, 1H), 5.42 (d, $^3J_{HH}$ = 1.4 Hz, 1H), 5.31 (d, $^3J_{HH}$ = 1.5 Hz, 1H).

^{13}C{^{1}H} NMR (101 MHz, $CDCl_3$) δ (ppm) = 149.4, 148.9, 143.9, 139.8, 128.5, 127.92, 127.90, 127.6, 125.3, 118.3, 117.1.

^{19}F{^{1}H} NMR ($CDCl_3$, 282 MHz) δ (ppm) = –62.5 (s).

IR (neat) ν (cm^{-1}): 3057, 1445, 1321, 1163, 1028, 1015, 909, 847, 778, 750, 699, 632, 597.

1-methoxy-4-(3-(*p*-tolyl)buta-1,3-dien-2-yl)benzene (1n)

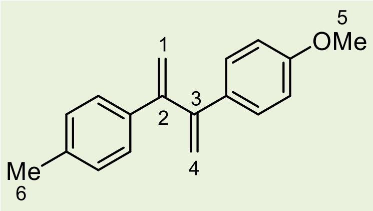

Following the general procedure using 1-(1-bromovinyl)-4-methoxybenzene (300 mg, 1.41 mmol, 1.0 equiv.), Mg (68.4 mg, 2.82 mmol, 2.0 equiv.), diethyl (1-(*p*-tolyl)vinyl) phosphate (361 mg, 1.34 mmol, 0.95 equiv.), [(dmpe)NiBr$_2$] (25.9 mg, 5 mol%). The reaction mixture was purified by column chromatography to afford the diene as colorless oil (200 mg, 57% yield).

TLC: Rf = 0.6 (pentane/Et_2O = 50:1).

^{1}H NMR (300 MHz, $CDCl_3$) δ (ppm) 7.33 – 7.18 (m, 4H), 7.07 – 6.98 (m, 2H), 6.78 – 6.73 (m, 2H), 5.46 (dd, $^3J_{HH}$ = 13.1, 1.8 Hz, 2H), 5.22 (dd, $^3J_{HH}$ = 13.0, 1.8 Hz, 2H), 3.73 (s, 3H), 2.26 (s, 3H).

^{13}C{^{1}H} NMR (75 MHz, $CDCl_3$) δ (ppm) = 159.2, 150.0, 149.4, 137.41, 137.35, 132.8, 129.0, 128.7, 127.4, 115.3, 114.6, 113.7, 55.3, 21.3.

HRMS (ESI +): calculated for $C_{18}H_{18}O$ $[M+H]^+$: 251.1431; found: 251.1432.

IR (neat) ν (cm^{-1}): 3657, 2995, 2836, 1915, 1506, 1439, 1293, 836, 827, 730, 668, 638, 627.

2-methoxy-5-(3-methylbuta-1,3-dien-2-yl)pyridine (1p)

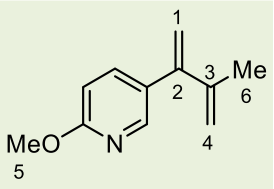

Following the general procedure using prop-1-en-2-ylmagnesium bromide (10.3 mL, 2.06 mmol, 1.0 equiv., 0.2 M in THF), diethyl (1-(6-methoxypyridin-3-yl)vinyl) phosphate (564 mg, 1.96 mmol, 0.95

equiv.), [(dmpe)$NiBr_2$] (37.7 mg, 5 mol%). The reaction mixture was purified by column chromatography to afford the diene as colorless oil (300 mg, 44% yield).

TLC: Rf = 0.42 (pentane/Et_2O = 10:1).

^{1}H NMR (300 MHz, $CDCl_3$) δ (ppm) 8.08 (dd, $^3J_{HH}$ = 2.5, 0.8 Hz, 1H), 7.48 (dd, $^3J_{HH}$ = 8.5, 2.4 Hz, 1H), 6.70 (dd, $^3J_{HH}$ = 8.5, 0.8 Hz, 1H), 5.32 – 5.30 (m, 1H), 5.12 – 5.11 (m, 2H), 4.85 – 4.82 (m, 1H), 3.95 (s, 3H), 2.00 (s, 3H).

^{13}C{^{1}H} NMR (75 MHz, $CDCl_3$) δ (ppm) = 163.6, 147.5, 146.3, 143.5, 139.3, 130.5, 117.2, 114.7, 110.0, 53.6, 21.2.

HRMS (ESI +): calculated for $C_{11}H_{13}NO$ $[M+H]^+$: 176.1070; found: 176.1059.

IR (neat) ν (cm^{-1}): 3094, 1563, 1368, 1341, 1281, 1175, 1097, 1024, 832, 810, 765, 685, 596, 579.

1-(3-methylbuta-1,3-dien-2-yl)-4-(trifluoromethyl)benzene (**1q**)

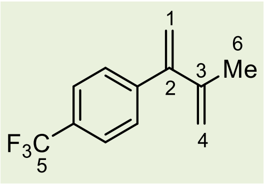

Following the general procedure using prop-1-en-2-ylmagnesium bromide (8.12 mL, 1.62 mmol, 1.0 equiv., 0.2 M in THF), diethyl (1-(4-(trifluoromethyl)phenyl)vinyl)phosphonate (500 mg, 1.54 mmol, 0.95 equiv.), [(dmpe)$NiBr_2$] (30 mg, 5 mol%). The reaction mixture was purified by column chromatography to afford the diene as colorless oil (230 mg, 67% yield).

TLC: Rf = 0.42 (pentane/Et_2O = 10:1).

^{1}H NMR (400 MHz, $CDCl_3$) δ (ppm) 7.58 (d, $^3J_{HH}$ = 8.1 Hz, 2H), 7.42 – 7.36 (m, 2H), 5.38 (s, 1H), 5.16 (s, 1H), 5.14 – 5.11 (m, 1H), 4.80 (s, 1H), 2.01 (s, 3H).

^{13}C{^{1}H} NMR (75 MHz, $CDCl_3$) δ (ppm) = 150.0, 145.46, 145.45, 143.2, 129.3, 125.1, 125.0, 123.1, 117.6, 115.4, 21.2.

^{19}F{^{1}H} NMR ($CDCl_3$, 282 MHz) δ (ppm) = –62.5 (s).

IR (neat) ν (cm^{-1}): 3096, 1593, 1321, 1163, 1108, 1092, 955, 899, 854, 839, 775, 710, 618, 593.

(3-methylenepent-4-ene-1,4-diyl)dibenzene (**1r**)

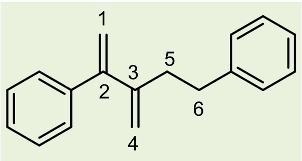

Following the general procedure using (3-bromobut-3-en-1-yl)benzene (317 mg, 1.50 mmol, 1.0 equiv.), Mg (72.9 mg, 3.0 mmol, 2.0 equiv.), diethyl (1-phenylvinyl) phosphate (365 mg, 1.42 mmol, 0.95 equiv.), [(dmpe)$NiBr_2$] (27.6 mg, 5 mol%). The reaction mixture was purified by column chromatography to afford the diene as colorless oil (125 mg, 36% yield).

TLC: Rf = 0.6 (pentane).

^{1}H NMR (400 MHz, $CDCl_3$) δ (ppm) 7.31 – 7.18 (m, 7H), 7.16 – 7.09 (m, 3H), 5.28 (d, *J* = 1.5 Hz, 1H), 5.18 – 5.15 (m, 1H), 5.05 – 5.01 (m, 1H), 4.93 (d, $^{3}J_{HH}$ = 1.8 Hz, 1H), 2.77 – 2.69 (m, 2H), 2.56 – 2.47 (m, 2H).

^{13}C{^{1}H} NMR (101 MHz, $CDCl_3$) δ (ppm) = 150.6, 148.4, 142.2, 141.3, 128.6, 128.4, 128.3, 128.2, 127.5, 126.0, 116.1, 113.9, 36.5, 34.8.

IR (neat) *ν* (cm^{-1}): 3084, 2936, 1808, 1493, 1444, 1392, 1218, 1156, 1027, 897, 777, 747, 621.

(3-cyclohexylbuta-1,3-dien-2-yl)benzene (**1s**)

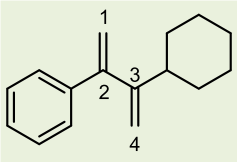

Following the general procedure using (1-bromovinyl)benzene (300 mg, 1.64 mmol, 1.0 equiv.), Mg (79.7 mg, 3.28 mmol, 2.0 equiv.), 1-cyclohexylvinyl diethyl phosphate (408 mg, 1.56+ mmol, 0.95 equiv.), [(dmpe)$NiBr_2$] (30.2 mg, 5 mol%). The reaction mixture was purified by column chromatography to afford the diene as colorless oil (139 mg, 40%).

TLC: Rf = 0.6 (pentane).

^{1}H NMR (300 MHz, $CDCl_3$) δ (ppm) 7.32 – 7.19 (m, 5H), 5.20 – 5.14 (m, 2H), 5.01 – 4.92 (m, 2H), 1.85 – 1.63 (m, 5H), 1.23 – 1.06 (m, 6H).

^{13}C{^{1}H} NMR (75 MHz, $CDCl_3$) δ (ppm) = 155.4, 151.5, 141.1, 128.1, 127.7, 127.3, 113.0, 112.3, 41.1, 32.5, 26.7, 26.4.

IR (neat) *ν* (cm^{-1}): 2923, 1590, 1492, 1297, 1262, 1170, 1027, 898, 776, 763, 736, 610.

(3*r*,5*r*,7*r*)-1-(3-phenylbuta-1,3-dien-2-yl)adamantane (**1t**)

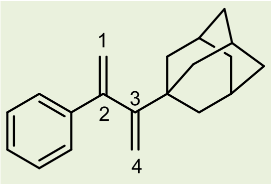

Following the general procedure using α-bromostyrene (800 mg, 4.37 mmol, 1.0 equiv.), Mg (212 mg, 8.74 mmol, 2.0 equiv.), diethyl (1-((3*r*,5*r*,7*r*)-adamantan-1-yl)vinyl)phosphonate (1.31 g, 4.15 mmol, 0.95 equiv.), [(dmpe)$NiBr_2$] (40 mg, 2.5 mol%). The reaction mixture was purified by column chromatography to afford the diene as colorless oil (735 mg, 64% yield).

TLC: Rf = 0.55 (pentane/Et_2O = 10:1).

^{1}H NMR (400 MHz, $CDCl_3$) δ (ppm) 7.47 – 7.40 (m, 2H), 7.33 – 7.22 (m, 3H), 5.40 (d, $^{3}J_{HH}$ = 2.1 Hz, 1H), 5.19 (d, $^{3}J_{HH}$ = 2.0 Hz, 1H), 4.97 (d, $^{3}J_{HH}$ = 2.1 Hz, 1H), 4.92 (d, $^{3}J_{HH}$ = 2.1 Hz, 1H), 1.90 (s, 3H), 1.67 – 1.51 (m, 12H).

^{13}C{^{1}H} NMR (101 MHz, $CDCl_3$) δ (ppm) = 159.4, 150.7, 141.5, 128.2, 127.4, 126.6, 114.9, 112.3, 42.1, 37.8, 36.9, 28.8.

IR (neat) *ν* (cm^{-1}): 2900, 1624, 1445, 1262, 1103, 1074, 906, 819, 777, 756, 672, 643, 560.

4. General procedure for the protoboration/oxidation sequence

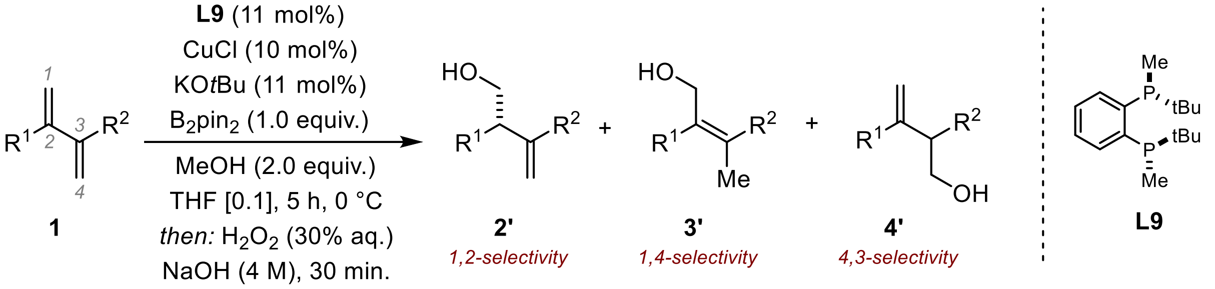

Protoboration: In a glove box, CuCl (0.05 mmol, 10 mol%), (*R*,*R*)-BenzP* (0.055 mmol, 11 mol%) were introduced into a Schlenk tube and dissolved in THF (2.0 mL). After stirring at room temperature for 10 min., B_2pin_2 (0.5 mmol, 1.0 equiv.) and a solution of KO*t*Bu (50 μL, 0.05 mmol, 10 mol%, 1.0 M in THF) were added. The reaction mixture was cooled to 0 °C. The appropriate diene (0.5 mmol, 1.0 equiv.) and MeOH (1.0 mmol, 2.0 equiv.) were added next. After stirring at 0 °C for 5 h, the reaction was filtered through a short pad of Celite®, washed with diethyl ether (3×10 mL) and the solvent was removed under vacuum. The conversion and isomeric ratio of boronic ester were determined by ^{1}H NMR analysis of the crude reaction mixture using an internal standard.

Oxidation: To a THF solution of boronic ester (3.0 mL) was added 30% H_2O_2 (1.5 mL) and 4 M NaOH (1.5 mL). The reaction mixture was stirred vigorously for 30 min. at room temperature. The mixture was diluted with water and extracted with diethyl ether (3×10 mL). The combined organic layers were washed with brine (10 mL), dried over Na_2SO_4, and concentrated. The residue was purified by column chromatography (pentane/ethyl acetate 6:1 to 3:1) to afford the corresponding alcohol. The yields reported in the manuscript are calculated over the 2 steps of the protoboration/oxidation sequence. The alcohol was used to determine the enantiomeric ratio by HPLC.

Note: all racemates were prepared according to the general procedure using Drewphos as ligand.[3]

(*S*)-2,3-diphenylbut-3-en-1-ol (**2'a**)

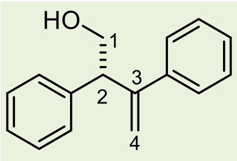

Following the general procedure using buta-1,3-diene-2,3-diyldibenzene (103.5 mg, 0.5 mmol, 1.0 equiv.), CuCl (4.95 mg, 0.05 mmol, 10 mol%), KO*t*Bu (50 µL, 0.05 mmol, 10 mol%, 1.0 M in THF), (*R*,*R*)-BenzP* (15.5 mg, 0.055 mmol, 11 mol%), B_2pin_2 (127 mg, 0.5 mmol, 1.0 equiv.) and MeOH (40.5 µL, 1.0 mmol, 2.0 equiv.). The reaction mixture was then oxidized under basic conditions following the general procedure and purified by column chromatography to afford the desired alcohol as a colorless oil (91.8 mg, 85% yield, 91:9 *er*)

TLC: Rf = 0.4 (Et_2O/pentane 1:1).

^{1}H NMR (400 MHz, $CDCl_3$) δ (ppm) 7.35 – 7.29 (m, 6H), 7.28 – 7.21 (m, 4H), 5.57 (d, $^3J_{HH}$ = 0.7 Hz, 1H), 5.24 (t, $^3J_{HH}$ = 1.0 Hz, 1H), 4.12 (dd, $^3J_{HH}$ = 6.9, 1.1 Hz, 1H), 4.09 – 3.91 (m, 2H), 1.59 (s, 1H).

^{13}C{^{1}H} NMR (101 MHz, $CDCl_3$) δ (ppm) = 148.5, 142.0, 140.1, 128.9, 128.6, 128.4, 127.7, 127.2, 126.7, 114.2, 65.9, 52.9.

HRMS: calculated for $C_{16}H_{16}O$ $[M+Na]^+$: 247.1094; found: 247.1076

IR (neat) ν (cm^{-1}): 3359, 2800, 1625, 1443, 1384, 1181, 1057, 902, 778, 751, 598, 566.

HPLC: 91:9 *er*, chiral stationary column: AD-H, mobile phase: hexane/*i*PrOH = 99/1, 1.0 mL/min, 254 nm, 30 °C, t(minor) = 42.5 min, t(major) = 47.8 min.

[α]$^{20}_{D}$ = –50.8 (c 0.95, $CHCl_3$).

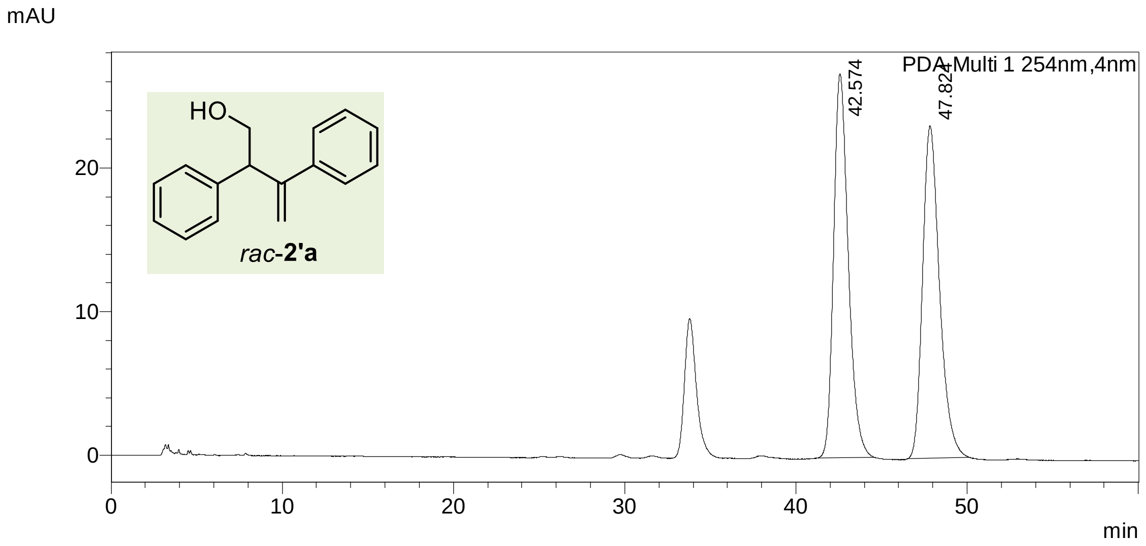

<Peak Table>

PDA Ch1 254nm

Peak#	Ret. Time	Area%
1	42.574	50.000
2	47.824	50.000
Total		100.000

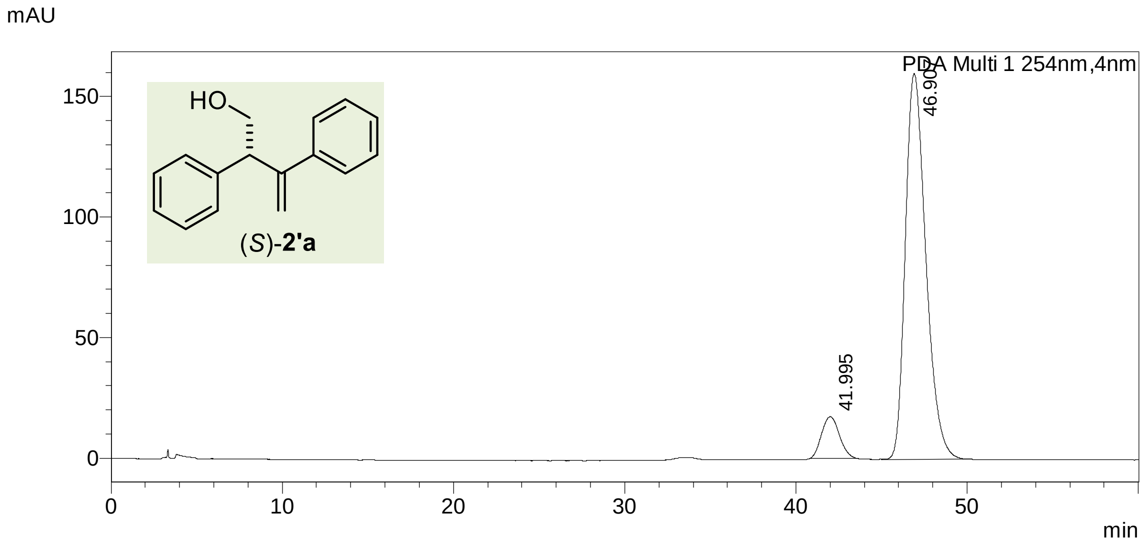

<Peak Table>

PDA Ch1 254nm

Peak#	Ret. Time	Area%
1	41.995	9.008
2	46.907	90.992
Total		100.000

(*S*)-2,3-bis(4-methoxyphenyl)but-3-en-1-ol (**2'b**)

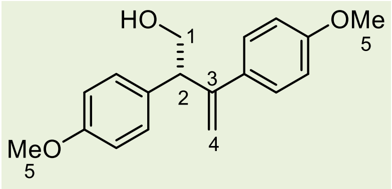

Following the general procedure using 4,4'-(buta-1,3-diene-2,3-diyl)bis(methoxybenzene) (133.5 mg, 0.5 mmol, 1.0 equiv.), CuCl (4.95 mg, 0.05 mmol, 10 mol%), KO*t*Bu (50 μL, 0.05 mmol, 10 mol%, 1.0 M in THF), (*R*,*R*)-BenzP* (15.5 mg, 0.055 mmol, 11 mol%), B_2pin_2 (127 mg, 0.5 mmol, 1.0 equiv.) and MeOH (40.5 μL, 1.0 mmol, 2.0 equiv.). The reaction mixture was then oxidized under basic conditions following the general procedure and purified by column chromatography to afford the desired alcohol as a colorless oil (115.0 mg, 81% yield, 84:16 *er*)

TLC: Rf = 0.4 (Et_2O/pentane 2:1).

^{1}H NMR (300 MHz, $CDCl_3$) δ (ppm) 7.22 – 7.10 (m, 4H), 6.73 (dd, $^3J_{HH}$ = 17.1, 8.8 Hz, 4H), 5.41 (d, $^3J_{HH}$ = 0.8 Hz, 1H), 5.06 (d, $^3J_{HH}$ = 1.0 Hz, 1H), 4.00 – 3.87 (m, 2H), 3.82 (dd, $^3J_{HH}$ = 7.9, 5.8 Hz, 1H), 3.69 (s, 3H), 3.69 (s, 3H), 1.54 (s, 1H).

^{13}C{^{1}H} NMR (75 MHz, $CDCl_3$) δ (ppm) = 159.2, 158.7, 147.9, 134.3, 132.1, 129.5, 127.8, 114.3, 113.7, 112.3, 66.0, 55.4, 52.0.

HRMS: calculated for $C_{18}H_{20}O_3$ $[M+H]^+$: 285.1475, found: 285.1491.

IR (neat) ν (cm^{-1}): 3415, 2929, 1607, 1463, 1441, 1243, 906, 811, 729, 618, 647.

HPLC: 84:16 *er*, chiral stationary column: OD-H, mobile phase: hexane/*i*PrOH = 95/5, 1.0 mL/min, 254 nm, 30 °C, t(major) = 23.48 min, t(minor) = 26.87 min.

[α]$^{20}_{D}$ = –20.3 (c 1.7, $CHCl_3$).

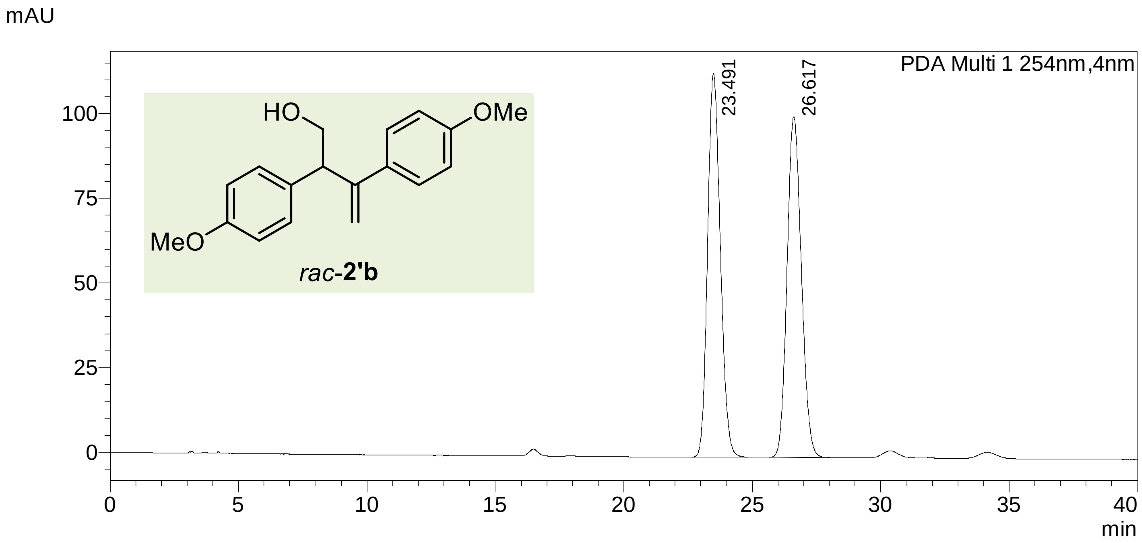

<Peak Table>

PDA Ch1 254nm

Peak#	Ret. Time	Area%
1	23.491	49.997
2	26.617	50.003
Total		100.000

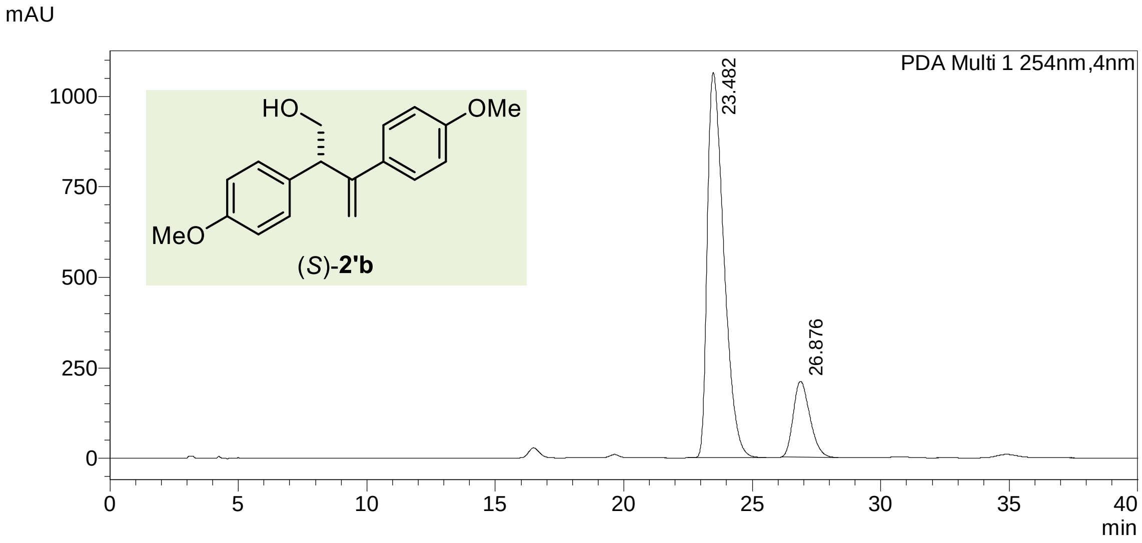

<Peak Table>

PDA Ch1 254nm

Peak#	Ret. Time	Area%
1	23.482	83.618
2	26.876	16.382
Total		100.000

(*S*)-2,3-di-*p*-tolylbut-3-en-1-ol (2'c)

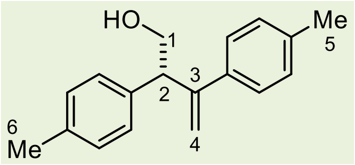

Following the general procedure using 4,4'-(buta-1,3-diene-2,3-diyl)bis(methylbenzene) (117.0 mg, 0.5 mmol, 1.0 equiv.), CuCl (4.95 mg, 0.05 mmol, 10 mol%), KO*t*Bu (50 μL, 0.05 mmol, 10 mol%, 1.0 M in THF), (*R*,*R*)-BenzP* (15.5 mg, 0.055 mmol, 11 mol%), B_2pin_2 (127 mg, 0.5 mmol, 1.0 equiv.) and MeOH (40.5 μL, 1.0 mmol, 2.0 equiv.). The reaction mixture was then oxidized under basic conditions following the general procedure and purified by column chromatography to afford the desired alcohol as a colorless oil (95.8 mg, 75% yield, 87:13 *er*)

TLC: Rf = 0.44 (Et_2O/pentane 1:1).

^{1}H NMR (400 MHz, $CDCl_3$) δ (ppm) 7.44 – 7.26 (m, 4H), 7.20 – 7.14 (m, 4H), 5.62 (s, 1H), 5.26 (s, 1H), 4.15 (q, $^3J_{HH}$ = 6.6 Hz, 1H), 4.11 – 3.95 (m, 2H), 2.39 (s, 3H), 2.38 (s, 3H), 1.64 (s, 1H).

^{13}C{^{1}H} NMR (75 MHz, $CDCl_3$) δ (ppm) = 148.3, 139.0, 137.39, 137.1, 136.7, 129.6, 129.1, 128.4, 126.5, 113.2, 66.0, 52.4, 21.19, 21.17.

HRMS: calculated for $C_{18}H_{20}O$ $[M+Na]^+$: 275,1407; found: 275.1417.

IR (neat) ν (cm^{-1}): 3415, 2248, 1583, 1298, 1243, 1060, 906, 831, 811, 831, 729, 618, 647.

HPLC: 88:12 *er*, chiral stationary column: OD-H, mobile phase: hexane/*i*PrOH = 99/1, 1.0 mL/min, 254 nm, 30 °C, t(minor) = 28.96 min, t(major) = 33.15 min.

[α]$^{20}_{D}$ = –20.3 (c 1.7, $CHCl_3$).

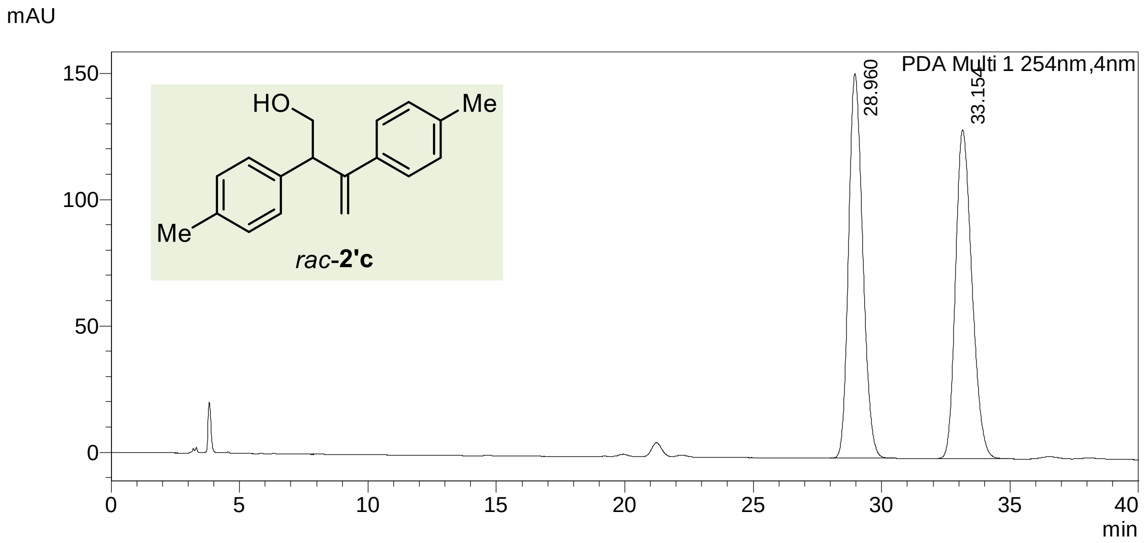

<Peak Table>

PDA Ch1 254nm

Peak#	Ret. Time	Area%
1	28.960	50.191
2	33.154	49.809
Total		100.000

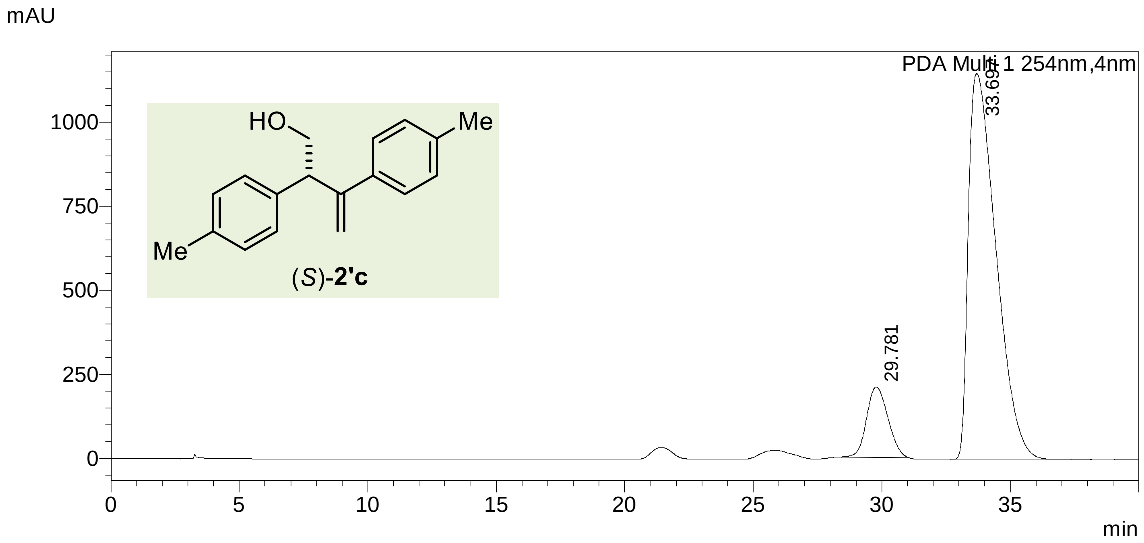

<Peak Table>

PDA Ch1 254nm

Peak#	Ret. Time	Area%
1	29.781	12.569
2	33.697	87.431
Total		100.000

(*S*)-2,3-di([1,1'-biphenyl]-4-yl)but-3-en-1-ol (**2'd**)

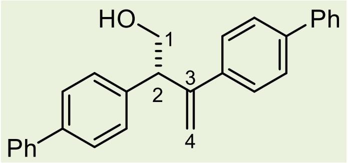

Following the general procedure using 4,4''-(buta-1,3-diene-2,3-diyl)di-1,1'-biphenyl (179.1 mg, 0.5 mmol, 1.0 equiv.), CuCl (4.95 mg, 0.05 mmol, 10 mol%), KO*t*Bu (50 μL, 0.05 mmol, 10 mol%, 1.0 M in THF), (*R*,*R*)-BenzP* (15.5 mg, 0.055 mmol, 11 mol%), B_2pin_2 (127 mg, 0.5 mmol, 1.0 equiv.) and MeOH (40.5 μL, 1.0 mmol, 2.0 equiv.). The reaction mixture was then oxidized under basic conditions following the general procedure and purified by column chromatography to afford the desired alcohol as a white solid (154.14 mg, 82% yield, 90:10 *er*)

TLC: Rf = 0.35 (Et_2O/pentane 2:1).

M.P.: 127.9-128.7 ˚C.

^{1}H NMR (400 MHz, $CDCl_3$) δ (ppm) 7.61 – 7.54 (m, 6H), 7.54 – 7.37 (m, 10H), 7.37 – 7.30 (m, 2H), 5.68 (s, 1H), 5.31 (s, 1H), 4.21 (t, $^3J_{HH}$ = 6.9 Hz, 1H), 4.13 (dd, $^3J_{HH}$ = 11.0, 6.8 Hz, 1H), 4.02 (dd, $^3J_{HH}$ = 11.0, 7.1 Hz, 1H), 1.65 (s, 1H).

^{13}C{^{1}H} NMR (101 MHz, $CDCl_3$) δ (ppm) = 147.9, 140.9, 140.72, 140.70, 140.5, 140.1, 139.2, 129.0, 128.9, 127.6, 127.5, 127.4, 127.2, 127.1, 127.09, 127.08, 114.2, 65.9, 52.4.

HRMS: calculated for $C_{28}H_{24}O$ $[M+H]^+$: 377.1901; found: 377.1911.

IR (neat) ν (cm^{-1}): 3305, 1623, 1599, 1147, 1402, 1207, 1057, 904, 839, 761, 734, 692.

HPLC: 90:10 *er*, chiral stationary column: OZ-H, mobile phase: hexane/*i*PrOH = 95/5, 1.0 mL/min, 254 nm, 30 °C, t(major) = 15.62 min, t(minor) = 19.29 min.

[α]$^{20}_{D}$ = –30.15 (c 0.60, $CHCl_3$).

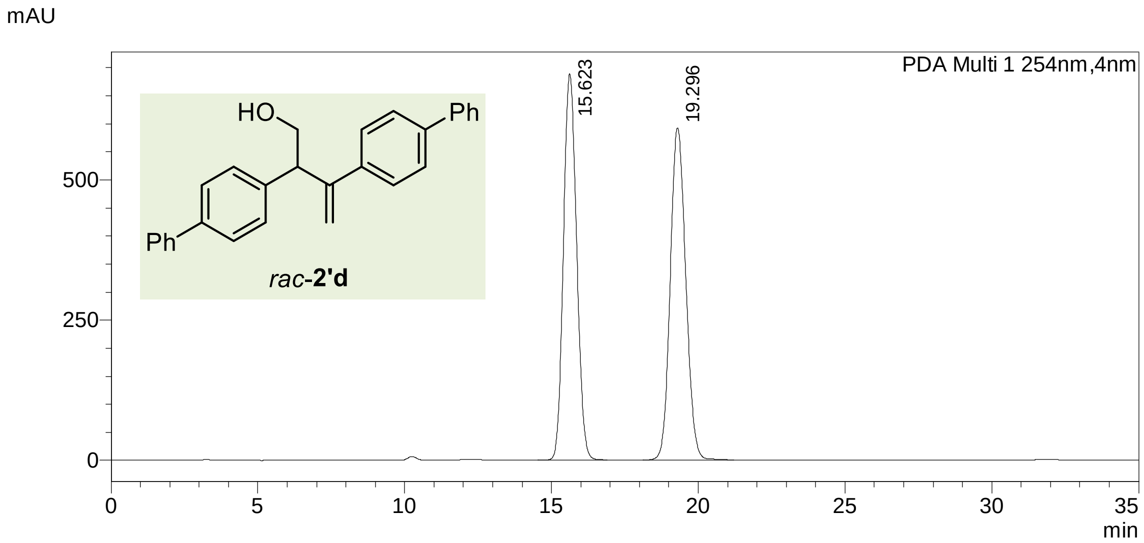

<Peak Table>

PDA Ch1 254nm

Peak#	Ret. Time	Area%
1	15.623	49.661
2	19.296	50.339
Total		100.000

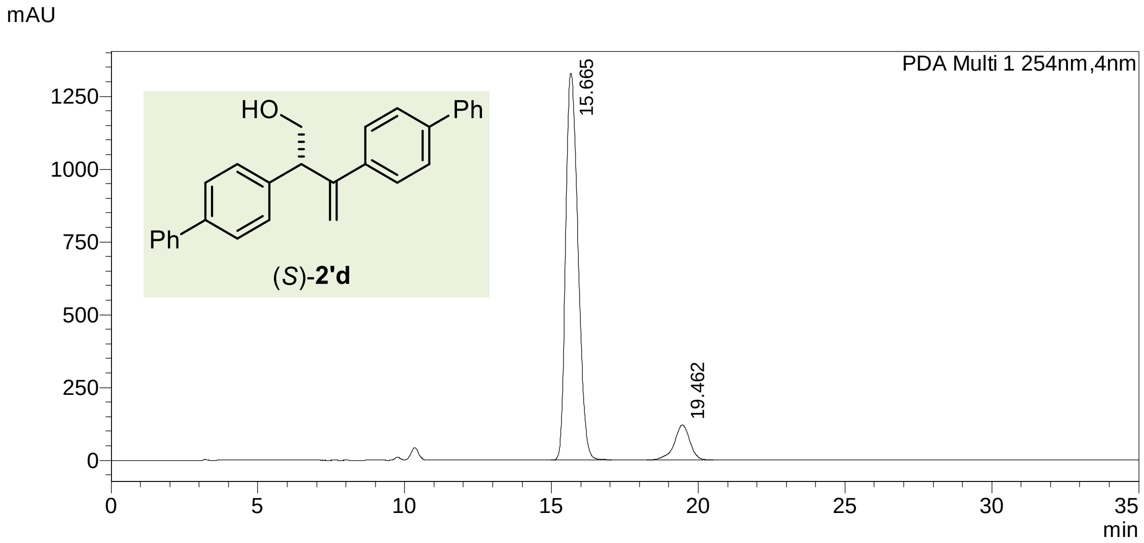

<Peak Table>

PDA Ch1 254nm

Peak#	Ret. Time	Area%
1	15.665	89.800
2	19.462	10.200
Total		100.000

(*S*)-2,3-bis(4-fluorophenyl)but-3-en-1-ol (**2'e**)

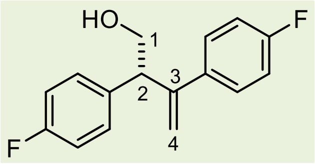

Following the general procedure using 4,4'-(buta-1,3-diene-2,3-diyl)bis(fluorobenzene) (121.0 mg, 0.5 mmol, 1.0 equiv.), CuCl (4.95 mg, 0.05 mmol, 10 mol%), KO*t*Bu (50 μL, 0.05 mmol, 10 mol%, 1.0 M in THF), (*R*,*R*)-BenzP* (15.5 mg, 0.055 mmol, 11 mol%), B_2pin_2 (127 mg, 0.5 mmol, 1.0 equiv.) and MeOH (40.5 μL, 1.0 mmol, 2.0 equiv.). The reaction mixture was then oxidized under basic conditions following the general procedure and purified by column chromatography to afford the desired alcohol as a colorless oil (118.3 mg, 91% yield, 91:9 *er*)

TLC: Rf = 0.20 (Et_2O/pentane 2:1).

^{1}H NMR (400 MHz, $CDCl_3$) δ (ppm) 7.29 – 7.20 (m, 4H), 7.01 – 6.91 (m, 4H), 5.50 (s, 1H), 5.21 (s, 1H), 4.07 – 3.99 (m, 2H), 3.94 – 3.86 (m, 1H), 1.70 (s, 1H).

^{13}C{^{1}H} NMR (100 MHz, $CDCl_3$) δ (ppm) = 163.5, 161.0, 147.5, 137.8, 135.5, 130.0, 128.3, 115.8, 115.3, 114.1, 65.73, 52.2.

^{19}F{^{1}H} NMR ($CDCl_3$, 282 MHz) δ (ppm) = –114.73, –115.65.

HRMS: calculated for $C_{16}H_{14}F_2O$ $[M+NH_4]^+$: 278.1352; found: 278.1330.

IR (neat) ν (cm^{-1}): 3308, 1627, 1602, 1506, 1325, 1313, 907, 833, 799, 738, 614, 583, 563.

HPLC: 91:9 *er*, chiral stationary column: OZ-H, mobile phase: hexane/*i*PrOH = 95/5, 1.0 mL/min, 254 nm, 30 °C, t(major) = 8.42 min, t(minor) = 9.87 min.

[α]$^{20}_{D}$ = –7.54 (c 0.87, $CHCl_3$).

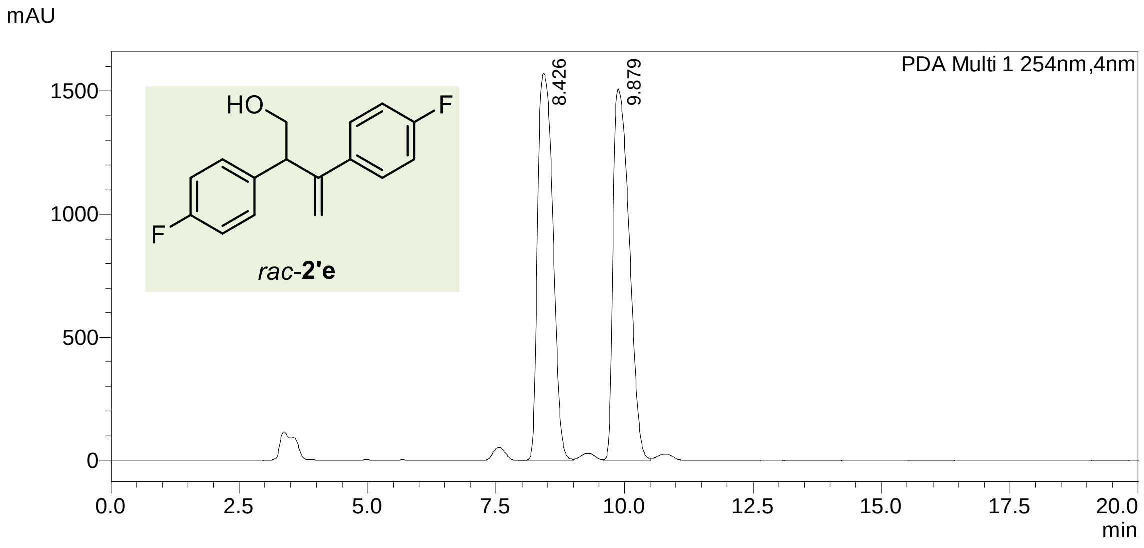

<Peak Table>

PDA Ch1 254nm

Peak#	Ret. Time	Area%
1	8.426	49.603
2	9.879	50.397
Total		100.000

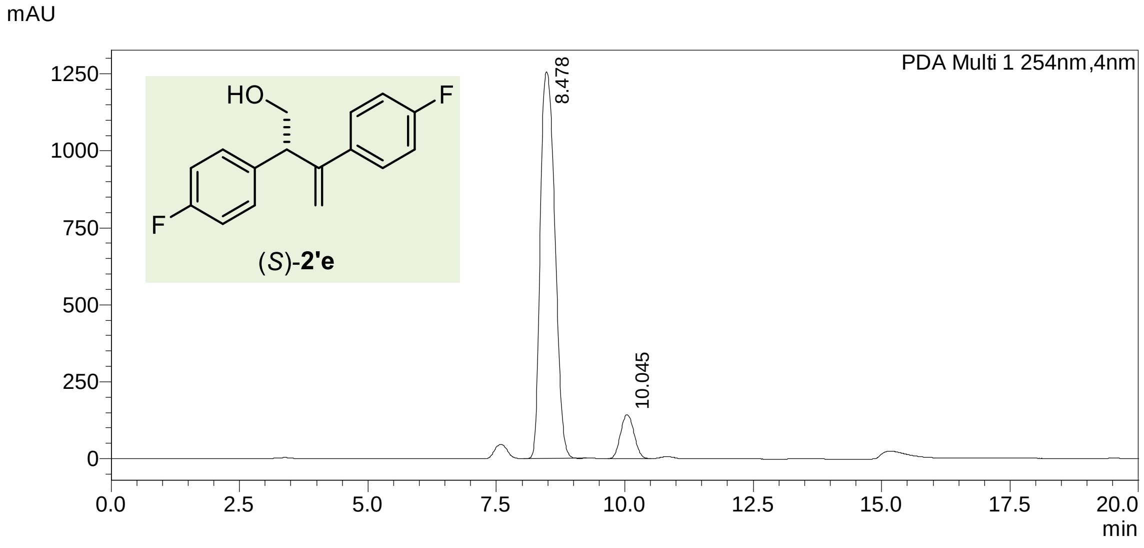

<Peak Table>

PDA Ch1 254nm

Peak#	Ret. Time	Area%
1	8.478	90.720
2	10.045	9.280
Total		100.000

(*S*)-2,3-bis(4-Cholorophenyl)but-3-en-1-ol (**2'f**)

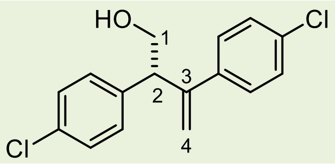

Following the general procedure using 4,4'-(buta-1,3-diene-2,3-diyl)bis(4-chlorobenzene) (146.0 mg, 0.5 mmol, 1.0 equiv.), CuCl (4.95 mg, 0.05 mmol, 10 mol%), KO*t*Bu (50 µL, 0.05 mmol, 10 mol%, 1.0 M in THF), (*R*,*R*)-BenzP* (15.5 mg, 0.055 mmol, 11 mol%), B_2pin_2 (127 mg, 0.5 mmol, 1.0 equiv.) and MeOH (40.5 µL, 1.0 mmol, 2.0 equiv.). The reaction mixture was then oxidized under basic conditions following the general procedure and purified by column chromatography to afford the desired alcohol as a colorless oil (103.4 mg, 89% yield, 90:10 *er*)

TLC: Rf = 0.20 (Et_2O/pentane 2:1).

^{1}H NMR (400 MHz, $CDCl_3$) δ (ppm) 7.35 – 7.28 (m, 4H), 7.28 – 7.20 (m, 4H), 5.58 (s, 1H), 5.29 (d, $^3J_{HH}$ = 1.0 Hz, 1H), 4.13 – 4.03 (m, 2H), 4.02 – 3.88 (m, 1H), 2.05 (s, 1H).

^{13}C{^{1}H} NMR (100 MHz, $CDCl_3$) δ (ppm) = 147.1, 140.0, 138.3, 133.7, 133.1, 129.9, 129.1, 128.7, 128.0, 114.8, 65.6, 52.1.

HRMS: calculated for $C_{16}H_{14}Cl_2O$ $[M+Na]^+$: 315.0315; found: 315.0325.

IR (neat) ν (cm^{-1}): 3343, 1625, 1593, 1261, 1057, 960, 907, 851, 831, 767, 658, 634, 565.

HPLC: 90:10 *er*, chiral stationary column: OZ-H, mobile phase: hexane/*i*PrOH = 95/5, 1.0 mL/min, 254 nm, 30 °C, t(major) = 8.64 min, t(minor) = 9.95 min.

$[\alpha]^{20}_D$ = –27.10 (c 0.38, $CHCl_3$).

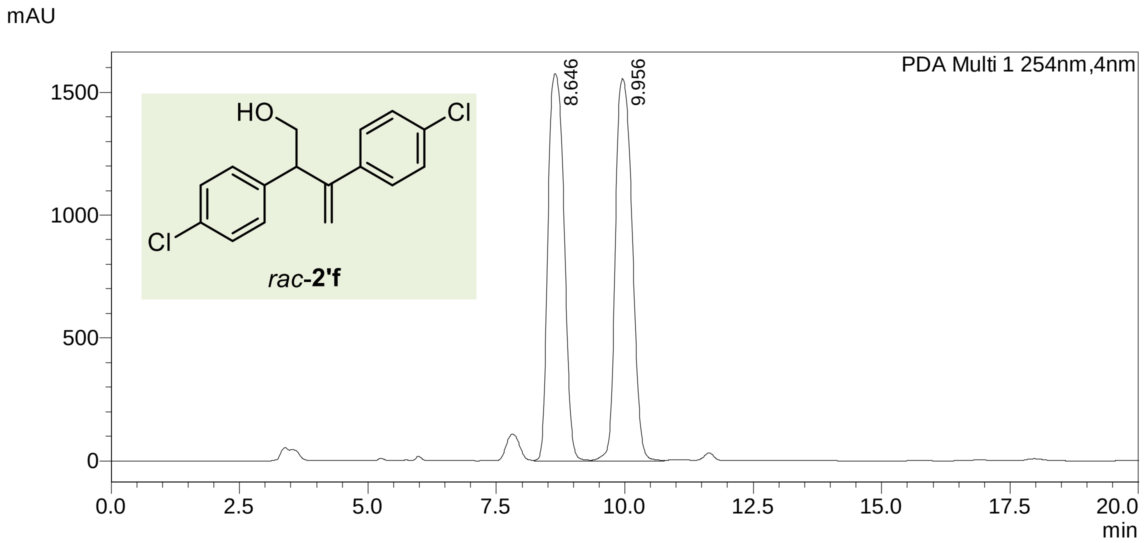

<Peak Table>

PDA Ch1 254nm

Peak#	Ret. Time	Area%
1	8.646	49.156
2	9.956	50.844
Total		100.000

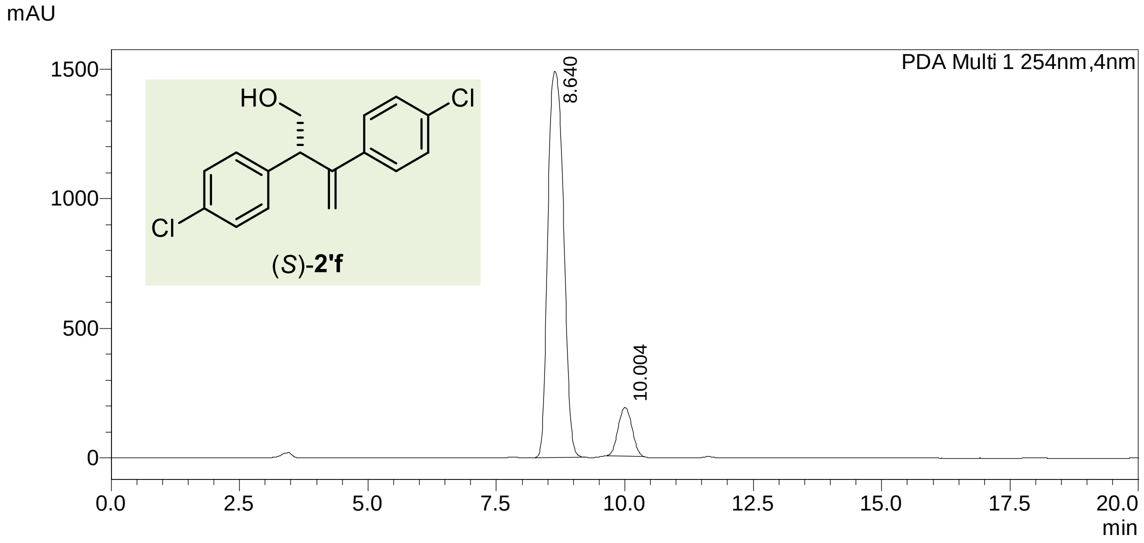

<Peak Table>

PDA Ch1 254nm

Peak#	Ret. Time	Area%
1	8.640	90.000
2	10.004	10.000
Total		100.000

(*S*)-2,3-bis(3-chlorophenyl)but-3-en-1-ol (**2'g**)

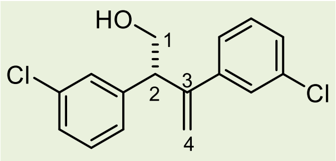

Following the general procedure using 3,3'-(buta-1,3-diene-2,3-diyl)bis(chlorobenzene) (146.0 mg, 0.5 mmol, 1.0 equiv.), CuCl (4.95 mg, 0.05 mmol, 10 mol%), KO*t*Bu (50 µL, 0.05 mmol, 10 mol%, 1.0 M in THF), (*R*,*R*)-BenzP* (15.5 mg, 0.055 mmol, 11 mol%), B_2pin_2 (127 mg, 0.5 mmol, 1.0 equiv.) and MeOH (40.5 µL, 1.0 mmol, 2.0 equiv.). The reaction mixture was then oxidized under basic conditions following the general procedure and purified by column chromatography to afford the desired alcohol as a colorless oil (134.3 mg, 92% yield, 93:7 *er*)

TLC: Rf = 0.40 (Et_2O/pentane 1:1).

^{1}H NMR (400 MHz, $CDCl_3$) δ (ppm) 7.31 – 7.13 (m, 8H), 5.58 (s, 1H), 5.27 (d, $^3J_{HH}$ = 1.0 Hz, 1H), 4.07 – 3.99 (m, 2H), 3.95 – 3.91 (m, 1H), 1.55 (s, 1H).

^{13}C{^{1}H} NMR (100 MHz, $CDCl_3$) δ (ppm) = 146.7, 143.32, 141.8, 134.7, 134.3, 130.0, 129.6, 128.4, 127.8, 127.4, 126.8, 126.7, 124.7, 115.5, 65.4, 52.3.

HRMS: calculated for $C_{16}H_{14}Cl_2O$ $[M+Na]^+$: 315.0315; found: 315.0289.

IR (neat) ν (cm^{-1}): 3351, 2971, 1940, 1593, 1189, 1065, 1027, 999, 909, 880, 728, 606, 565.

HPLC: 93:7 *er*, chiral stationary column: AD-H, mobile phase: hexane/*i*PrOH = 95/5, 1.0 mL/min, 254 nm, 30 °C, t(minor) = 11.59 min, t(major) = 13.22 min.

[α]$^{20}_D$ = –29.14 (c 0.66, $CHCl_3$).

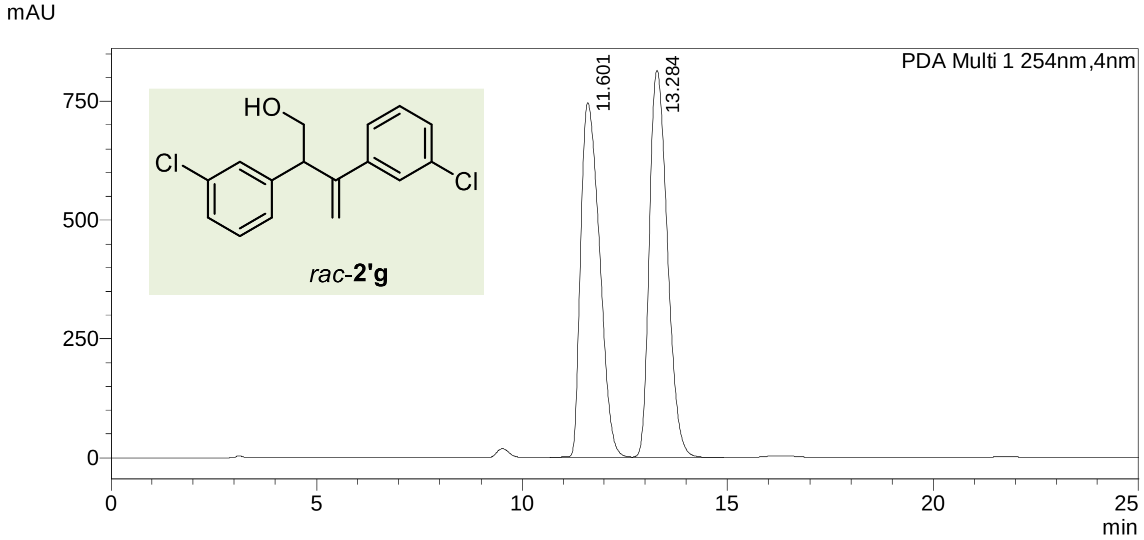

<Peak Table>

PDA Ch1 254nm

Peak#	Ret. Time	Area%
1	11.601	49.061
2	13.284	50.939
Total		100.000

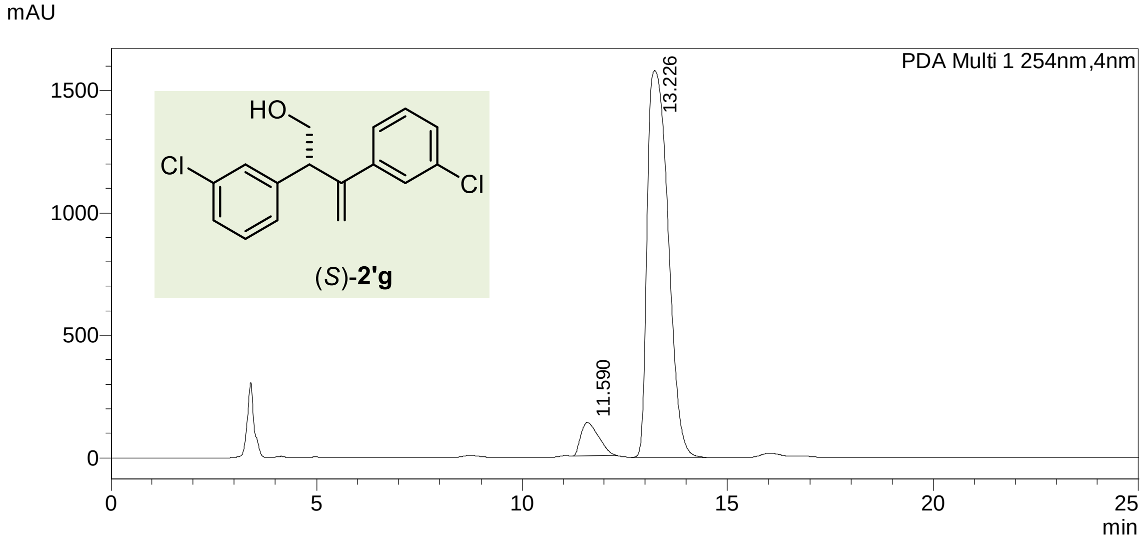

<Peak Table>

PDA Ch1 254nm

Peak#	Ret. Time	Area%
1	11.590	6.814
2	13.226	93.186
Total		100.000

(*R*)-2,3-bis(2-chlorophenyl)but-3-en-1-ol (2'h)

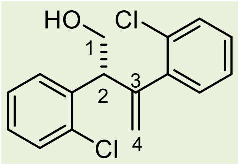

Following the general procedure using 2,2'-(buta-1,3-diene-2,3-diyl)bis(chlorobenzene) (146.0 mg, 0.5 mmol, 1.0 equiv.), CuCl (4.95 mg, 0.05 mmol, 10 mol%), KO*t*Bu (50 μL, 0.05 mmol, 10 mol%, 1.0 M in THF), (*R*,*R*)-BenzP* (15.5 mg, 0.055 mmol, 11 mol%), B_2pin_2 (127 mg, 0.5 mmol, 1.0 equiv.) and MeOH (40.5 μL, 1.0 mmol, 2.0 equiv.). The reaction mixture was then oxidized under basic conditions following the general procedure and purified by column chromatography to afford the desired alcohol as a colorless oil (75.9 mg, 52% yield, 85:15 *er*)

TLC: Rf = 0.51 (Et_2O/pentane 1:1).

^{1}H NMR (400 MHz, $CDCl_3$) δ (ppm) 7.44 (dd, $^3J_{HH}$ = 7.7, 1.8 Hz, 1H), 7.34 (ddd, $^3J_{HH}$ = 7.9, 4.5, 1.5 Hz, 2H), 7.23 (dd, $^3J_{HH}$ = 7.6, 1.5 Hz, 1H), 7.17 (td, $^3J_{HH}$ = 8.0, 1.9 Hz, 2H), 7.11 (td, $^3J_{HH}$ = 7.5, 1.4 Hz, 1H), 7.03 (dd, $^3J_{HH}$ = 7.5, 1.9 Hz, 1H), 5.37 (s, 1H), 5.30 (s, 1H), 4.61 (t, $^3J_{HH}$ = 6.5 Hz, 1H), 4.08 – 4.02 (m, 1H), 4.0 – 3.95 (m, 1H), 1.74 (s, 1H).

^{13}C{^{1}H} NMR (100 MHz, $CDCl_3$) δ (ppm) = 145.8, 140.8, 137.0, 135.3, 132.4, 130.6, 129.9, 129.7, 129.5, 128.7, 128.4, 127.0, 126.6, 118.4, 63.8, 50.2.

HRMS: calculated for $C_{16}H_{14}Cl_2O$ $[M+Na]^+$: 315.0315; found: 315.0325.

IR (neat) ν (cm^{-1}): 3391, 2247, 1636, 1431, 1126, 909, 850, 753, 734, 689, 646, 591, 557.

HPLC: 85:15 *er*, chiral stationary column: OZ-H, mobile phase: hexane/*i*PrOH = 95/5, 1.0 mL/min, 254 nm, 30 °C, t(minor) = 9.17 min, t(major) = 11.37 min.

$[\alpha]^{20}_D$ = –30.54 (c 0.66, $CHCl_3$).

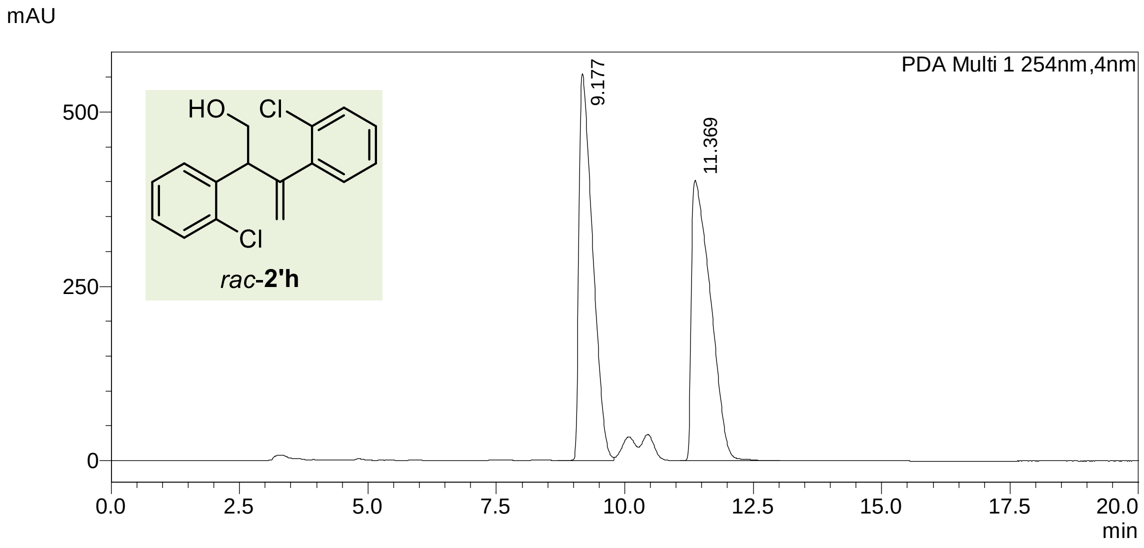

<Peak Table>

PDA Ch1 254nm

Peak#	Ret. Time	Area%
1	9.177	49.844
2	11.369	50.156
Total		100.000

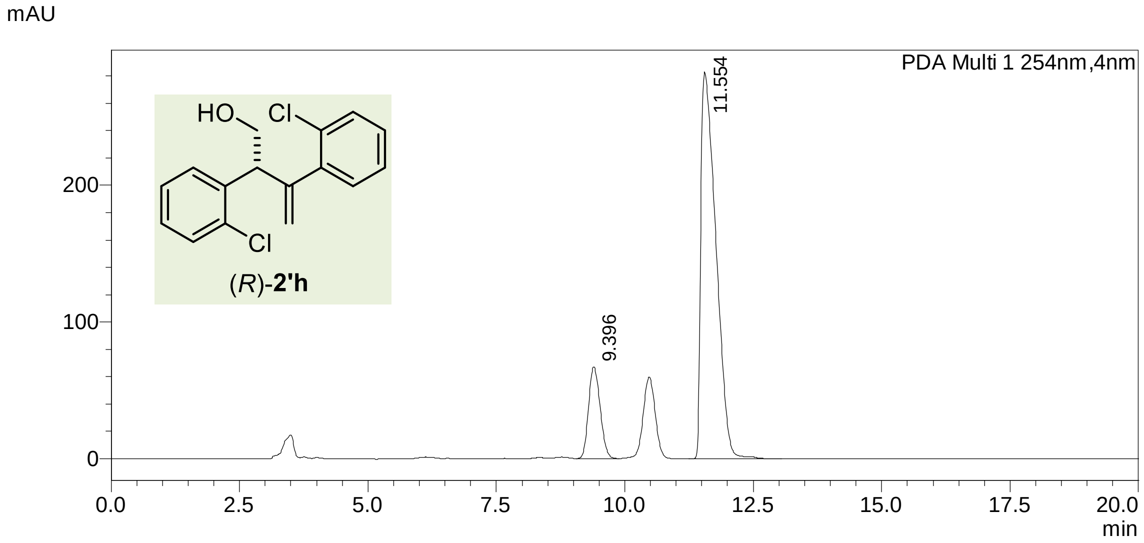

<Peak Table>

PDA Ch1 254nm

Peak#	Ret. Time	Area%
1	9.396	15.490
2	11.554	84.510
Total		100.000

(*S*)-2,3-bis(4-(trifluoromethyl)phenyl)but-3-en-1-ol (**2'i**)

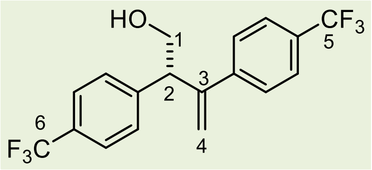

Following the general procedure using 4,4'-(buta-1,3-diene-2,3-diyl)bis((trifluoromethyl)benzene) (171.0 mg, 0.5 mmol, 1.0 equiv.), CuCl (4.95 mg, 0.05 mmol, 10 mol%), KO*t*Bu (50 µL, 0.05 mmol, 10 mol%, 1.0 M in THF), (*R*,*R*)-BenzP* (15.5 mg, 0.055 mmol, 11 mol%), B_2pin_2 (127 mg, 0.5 mmol, 1.0 equiv.) and MeOH (40.5 µL, 1.0 mmol, 2.0 equiv.). The reaction mixture was then oxidized under basic conditions following the general procedure and purified by column chromatography to afford the desired alcohol as a colorless oil (171 mg, 95% yield, 92:8 er).

TLC: Rf = 0.34 (Et_2O/pentane 1:1).

^{1}H NMR (400 MHz, $CDCl_3$) δ (ppm) 7.55 (dd, $^3J_{HH}$ = 19.0, 8.1 Hz, 4H), 7.40 (t, $^3J_{HH}$ = 7.8 Hz, 4H), 5.64 (s, 1H), 5.36 (d, $^3J_{HH}$ = 1.1 Hz, 1H), 4.19 – 4.05 (m, 2H), 4.02 – 3.92 (m, 1H), 1.59 (s, 1H).

^{13}C{^{1}H} NMR (100 MHz, $CDCl_3$) δ (ppm) = 146.9, 145.1, 143.9, 129.7, 128.9, 128.3, 127.0, 125.9, 125.5, 123.0, 122.8, 116.4, 112.4, 65.4, 52.5.

^{19}F{^{1}H} NMR ($CDCl_3$, 282 MHz) δ (ppm) = –62.53, –62.63.

LRMS (ESI+): calculated for $C_{18}H_{14}F_6O$ $[M+NH_4]^+$: 378.3 found: 378.5.

IR (neat) ν (cm^{-1}): 3335, 2940, 2810, 1617, 1419, 1406, 1320, 1162, 1110, 1065, 1014, 957, 916, 881, 844, 734, 661, 607, 560, 570.

HPLC: 92:8 *er*, chiral stationary column: AD-H, mobile phase: hexane/iPrOH = 99/1, 1.0 mL/min, 254 nm, 30 °C, t(minor) = 60.89 min, t(major) = 67.93 min.

[α]$^{20}_D$ = –28.93 (c 1.51, $CHCl_3$).

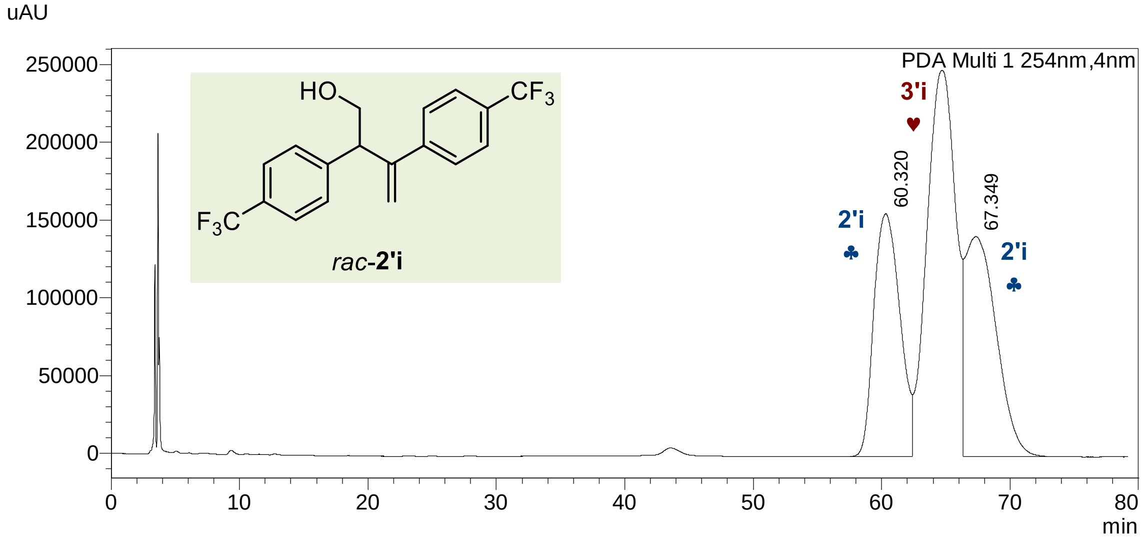

<Peak Table>

PDA Ch1 254nm

Peak#	Ret. Time	Area%
1	60.320	48.139
2	67.349	51.861
Total		100.000

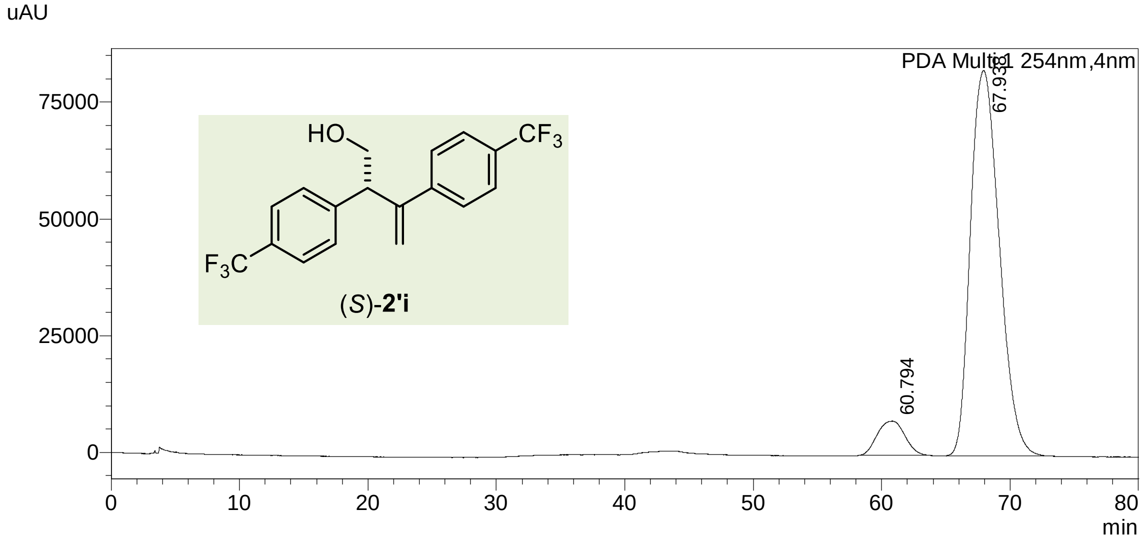

<Peak Table>

PDA Ch2 210nm

Peak#	Ret. Time	Area%
1	60.899	7.936
2	67.937	92.064
Total		100.000

(*S*)-3-methylene-2-phenethyl-5-phenylpentan-1-ol (2'j)

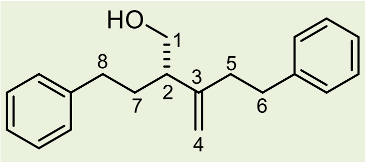

Following the general procedure using (3,4-dimethylenehexane-1,6-diyl)dibenzene (132.18 mg, 0.5 mmol, 1.0 equiv.), CuCl (4.95 mg, 0.05 mmol, 10 mol%), KO*t*Bu (50 μL, 0.05 mmol, 10 mol%, 1.0 M in THF), (*R*,*R*)-BenzP* (15.5 mg, 0.055 mmol, 11 mol%), B_2pin_2 (127 mg, 0.5 mmol, 1.0 equiv.) and MeOH (40.5 μL, 1.0 mmol, 2.0 equiv.). The reaction mixture was then oxidized under basic conditions following the general procedure and purified by column chromatography to afford alcohol as colorless liquid (105.1 mg, 75% yield, 88:12 *er*)

TLC: Rf = 0.52 (Et_2O/pentane 1:1).

^{1}H NMR (400 MHz, $CDCl_3$) δ (ppm) = 7.32 – 7.22 (m, 4H), 7.22 – 7.10 (m, 6H), 5.08 (d, $^3J_{HH}$ = 1.5 Hz, 1H), 4.96 (s, 1H), 3.54 (d, $^3J_{HH}$ = 6.5 Hz, 2H), 2.79 (t, $^3J_{HH}$ = 8.1 Hz, 2H), 2.62 – 2.53 (m, 2H), 2.36 – 2.24 (m, 3H), 1.80 – 1.64 (m, 2H), 1.32 (s, 1H).

^{13}C{^{1}H} NMR (100 MHz, $CDCl_3$) δ (ppm) = 148.7, 142.3, 142.0, 128.6, 128.5, 126.1, 126.0, 112.3, 64.5, 48.9, 35.4, 34.3, 33.6, 31.9.

HRMS: calculated for $C_{20}H_{24}O$ $[M+Na]^+$: 303.1720; found: 303.1729.

IR (neat) ν (cm^{-1}): 3375, 3062, 1641, 1603, 1495, 1453, 1180, 1065, 1029, 892, 743, 696.

HPLC: 88:12 *er*, chiral stationary column: OD-H, mobile phase: hexane/*i*PrOH = 95/5, 1.0 mL/min, 254 nm, 30 °C, t(major) = 22.20 min, t(minor) = 25.41 min.

[α]$^{20}_{D}$ = –22.4 (c 0.46, $CHCl_3$).

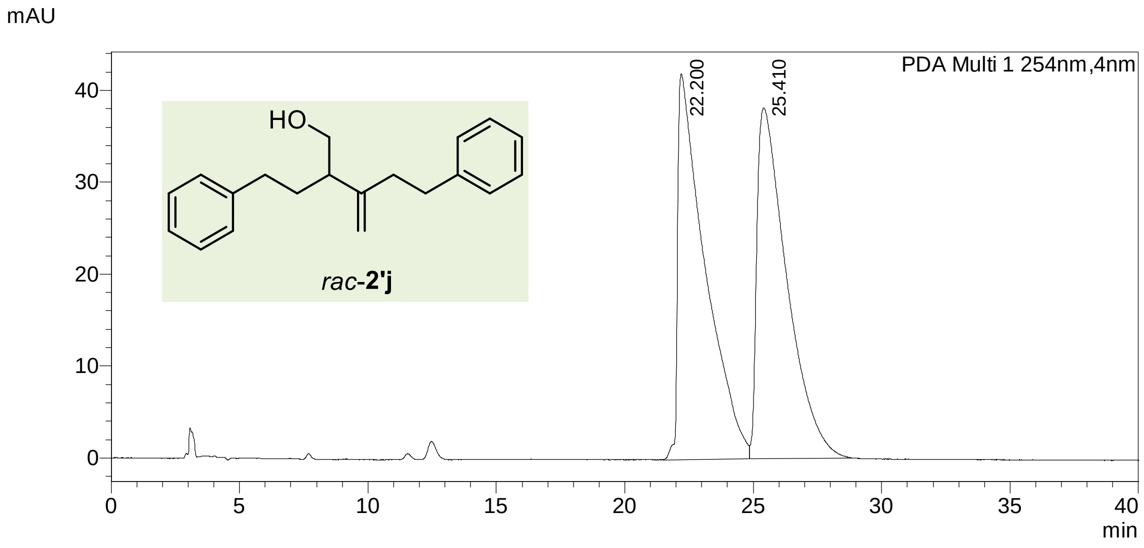

<Peak Table>

PDA Ch1 254nm

Peak#	Ret. Time	Area%
1	22.200	50.008
2	25.410	49.992
Total		100.000

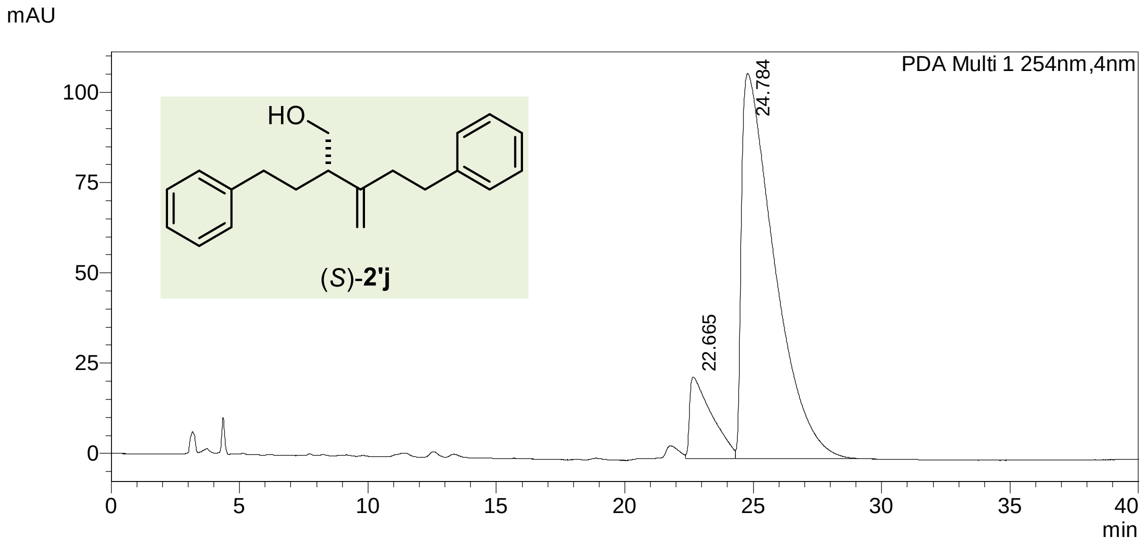

<Peak Table>

PDA Ch1 254nm

Peak#	Ret. Time	Area%
1	22.665	12.440
2	24.784	87.560
Total		100.000

***rac*-2,3-dicyclohexylbut-3-en-1-ol (2'k)**

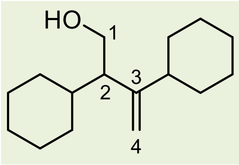

Following the general procedure using buta-1,3-diene-2,3-diyldicyclohexane (109.1 mg, 0.5 mmol, 1.0 equiv.), CuCl (4.95 mg, 0.05 mmol, 10 mol%), KO*t*Bu (50 μL, 0.05 mmol, 10 mol%, 1.0 M in THF), Drewphos (15.5 mg, 0.055 mmol, 11 mol%), B_2pin_2 (127 mg, 0.5 mmol, 1.0 equiv.) and MeOH (40.5 μL, 1.0 mmol, 2.0 equiv.). The reaction mixture was then oxidized under basic conditions following the general procedure and purified by column chromatography to afford alcohol as colorless liquid (80.2 mg, 68% yield)

TLC: Rf = 0.60 (Et_2O/pentane 1:2).

^{1}H NMR (400 MHz, $CDCl_3$) δ (ppm) = 5.00 (s, 1H), 4.80 (d, $^3J_{HH}$ = 1.3 Hz, 1H), 3.63 – 3.59 (m, 2H), 2.02 – 1.97 (m, 1H), 1.83 – 1.65 (m, 10H), 1.45 – 1.36 (m, 2H), 1.31 – 1.05 (m, 9H), 0.96 – 0.84 (m, 2H).

^{13}C{^{1}H} NMR (100 MHz, $CDCl_3$) δ (ppm) = 155.7, 109.0, 62.3, 53.9, 44.9, 38.7, 33.3, 33.0, 32.0, 30.7, 27.1, 26.8, 26.6, 26.5.

HRMS: calculated for $C_{16}H_{28}O$ $[M+Na]^+$: 259.2033; found: 259.2008.

IR (neat) ν (cm^{-1}): 3375, 2920, 1637, 1447, 1194, 1043, 1011, 970, 885, 742, 727, 664.

(*S*)-3-(4-methoxyphenyl)-2-(4- (trifluoromethyl)phenyl)but-3-en-1-ol (2'l)

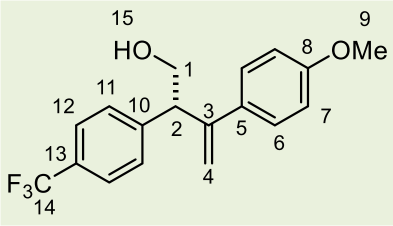

Following the general procedure using 1-methoxy-4-(3-(4-(trifluoromethyl)phenyl)buta-1,3-dien-2-yl)benzene (152.5 mg, 0.5 mmol, 1.0 equiv.), CuCl (4.95 mg, 0.05 mmol, 10 mol%), KO*t*Bu (50 μL, 0.05 mmol, 10 mol%, 1.0 M in THF), (*R*,*R*)-BenzP* (15.5 mg, 0.055 mmol, 11 mol%), B_2pin_2 (127 mg, 0.5 mmol, 1.0 equiv.) and MeOH (40.5 μL, 1.0 mmol, 2.0 equiv.). The reaction mixture was then oxidized under basic conditions following the general procedure and purified by column chromatography to afford the desired alcohol as a colorless oil (119.2 mg, 74% yield, 89:11 er_2, 1.5:1 $rr_{2/3}$).

TLC: Rf = 0.36 (Et_2O/pentane 1:1).

^{1}H NMR (400 MHz, $CDCl_3$) δ (ppm) = 7.55 (d, $^3J_{H\text{-}H}$ = 8.0 Hz, 2H, H12), 7.43 (d, $^3J_{H\text{-}H}$ = 8.1 Hz, 2H, H11), 7.27– 7.23 (m, 2H, H6), 6.86 – 6.74 (m, 2H, H7), 5.55 (s, 1H, H4), 5.15 (s, 1H, H4), 4.16 (t, $^3J_{H\text{-}H}$ = 6.8 Hz, 1H, H2), 4.08 (dd, $J_{H\text{-}H}$ = 11.0, 6.7 Hz, 1H, H1), 3.95 (dd, $J_{H\text{-}H}$ = 11.0, 6.9 Hz, 1H, H1), 3.77 (s, 3H, H9), 1.78 (bs, 1H, H15).

^{13}C{^{1}H} NMR (100 MHz, $CDCl_3$) δ (ppm) = 159.4 (C8), 147.1 (C3), 144.7 (C10), 133.8 (C5), 129.4 (q, $^2J_{C\text{-}F}$ = 32.5 Hz, C13), (128.9 (C11), 127.7 (C6), 125.7 (q, $^3J_{C\text{-}F}$ = 3.8 Hz, C12), 113.9 (C7), 113.3 (C4), 65.6 (C1), 55.4 (C9), 52.5 (C2). C14 not detected.

^{19}F{^{1}H} NMR ($CDCl_3$, 282 MHz) δ (ppm) = –62.5 (s).

HRMS: calculated for $C_{18}H_{17}F_3O_2$: $[M+H]^+$: 323.1254; found: 323.1241.

IR (neat) ν (cm^{-1}): 2860, 1608, 1510, 1322, 1292, 1161, 1117, 1032, 1016, 831, 812, 787, 607, 563.

HPLC: 89:11 er, chiral stationary column: AD-H, mobile phase: hexane/*i*PrOH = 95/5, 1.0 mL/min, 254 nm, 30 °C, t(major) = 9.20 min, t(minor) = 11.41 min.

[α]$^{20}_{D}$ = –20.8 (c 0.75, $CHCl_3$).

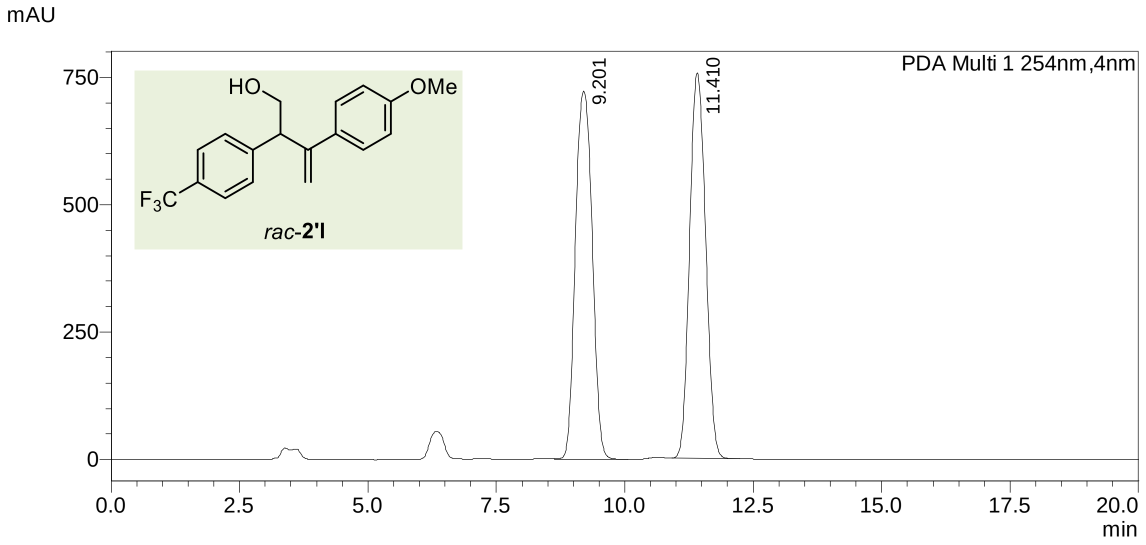

<Peak Table>

PDA Ch1 254nm

Peak#	Ret. Time	Area%
1	9.201	50.170
2	11.410	49.830
Total		100.000

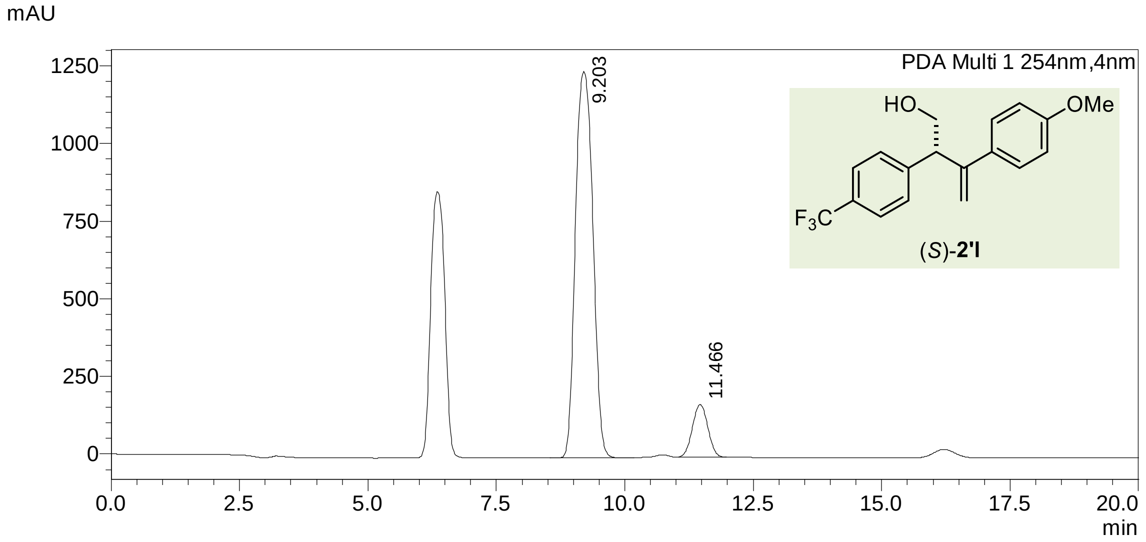

<Peak Table>

PDA Ch1 254nm

Peak#	Ret. Time	Area%
1	9.203	89.264
2	11.466	10.736
Total		100.000

(*S*)-2,3-di(thiophen-2-yl)but-3-en-1-ol (**2'm**)

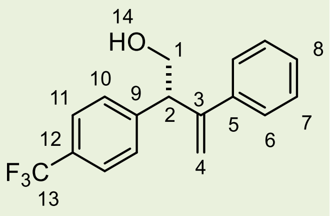

Following the general procedure using 1-(3-phenylbuta-1,3-dien-2-yl)-4-(trifluoromethyl)benzene (137.0 mg, 0.5 mmol, 1.0 equiv.), CuCl (4.95 mg, 0.05 mmol, 10 mol%), KO*t*Bu (50 µL, 0.05 mmol, 10 mol%, 1.0 M in THF), (*R*,*R*)-BenzP* (15.5 mg, 0.055 mmol, 11 mol%), B_2pin_2 (127 mg, 0.5 mmol, 1.0 equiv.) and MeOH (40.5 µL, 1.0 mmol, 2.0 equiv.). The reaction mixture was then oxidized under basic conditions following the general procedure and purified by column chromatography to afford alcohol as colorless liquid (121.2 mg, 92% yield, 92:8 er_2, 13:1 $rr_{2/4}$).

TLC: Rf = 0.26 (Et_2O/pentane 1:1).

^{1}H NMR (400 MHz, $CDCl_3$) δ (ppm) = 7.56 (d, $^3J_{H-H}$ = 8.1 Hz, 2H, H11), 7.44 (d, $^3J_{H-H}$ = 8.1 Hz, 2H, H10), 7.34 – 7.22 (m, 5H, H6, H7 and H8), 5.59 (s, 1H, H4), 5.23 (bs, 1H, H4), 4.19 (t, $^3J_{HH}$ = 6.8 Hz, 1H, H2), 4.09 (dd, J_{H-H} = 11.0, 6.6 Hz, 1H, H1), 3.96 (dd, J_{H-H} = 11.0, 7.1 Hz, 1H, H1), 1.54 (s, 1H, H14).

^{13}C{^{1}H} NMR (100 MHz, $CDCl_3$) δ (ppm) = 147.8 (C3), 144.4 (q, $^5J_{C-F}$ = 1.5 Hz, C9), 141.3 (C5), 129.3 (q, $^2J_{C-F}$ = 32.3 Hz, C12), 128.8 (C10), 128.4 (C6 or C7), 127.8 (C8), 126.9 (C6 or C7), 125.6 (q, $^3J_{C-F}$ = 3.8 Hz, C11), 124.2 (q, $^1J_{C-F}$ = 271.9 Hz, C13), 114.7 (C4), 65.5 (C1), 52.6 (C2).

^{19}F{^{1}H} NMR ($CDCl_3$, 282 MHz) δ (ppm) = –62.47.

LRMS (ESI+): calculated for $C_{17}H_{15}F_3O$ $[M+NH_4]^+$: 310.1; found: 310.7.

IR (neat) ν (cm^{-1}): 3362, 1618, 1418, 1325, 1164, 1068, 1018, 842, 779, 705, 652, 559.

HPLC: 92:8 *er*, chiral stationary column: OZ-H, mobile phase: hexane/iPrOH = 95/5, 1.0 mL/min, 254 nm, 30 °C, t(major) = 6.14 min, t(minor) = 8.78 min.

[α]$^{20}_{D}$ = –31.84 (c 0.24, $CHCl_3$).

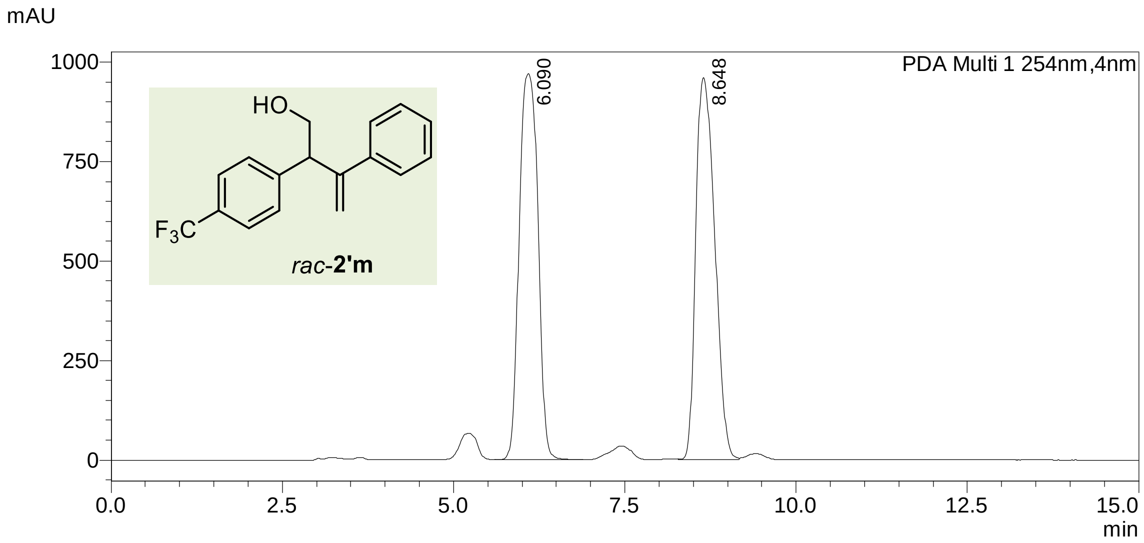

<Peak Table>

PDA Ch1 254nm

Peak#	Ret. Time	Area%
1	6.090	49.909
2	8.648	50.091
Total		100.000

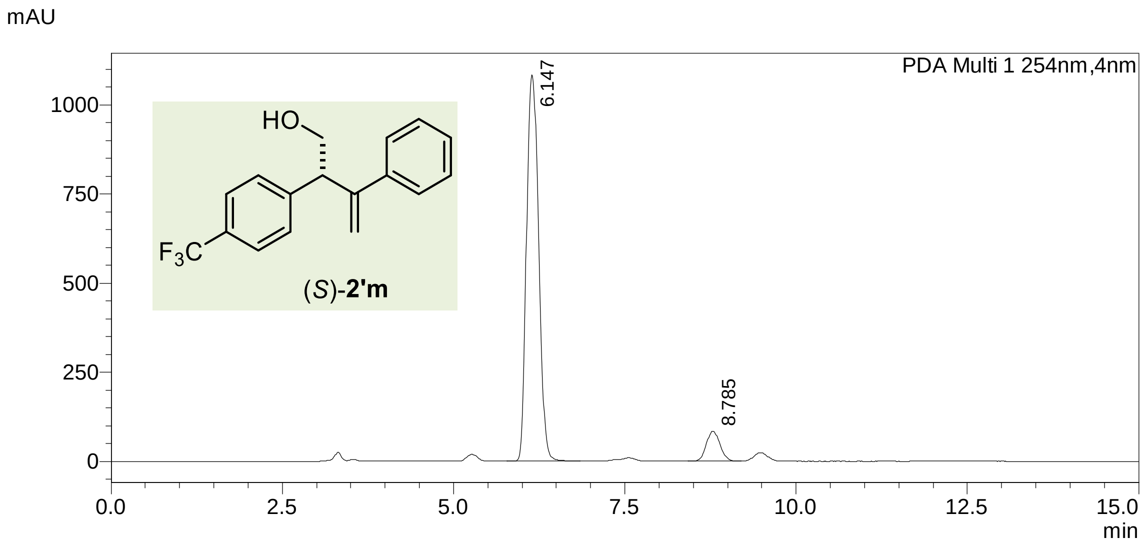

<Peak Table>

PDA Ch1 254nm

Peak#	Ret. Time	Area%
1	6.147	91.905
2	8.785	8.095
Total		100.000

(*S*)-2-(4-methoxyphenyl)-3-(p-tolyl)but-3-en-1-ol (4'n) (minor)

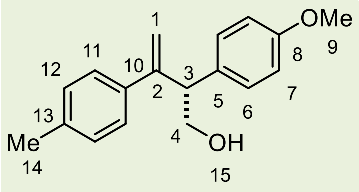

Following the general procedure using 1-methoxy-4-(3-(p-tolyl)buta-1,3-dien-2-yl)benzene (125.0 mg, 0.5 mmol, 1.0 equiv.), CuCl (4.95 mg, 0.05 mmol, 10 mol%), KO*t*Bu (50 μL, 0.05 mmol, 10 mol%, 1.0 M in THF), (*R*,*R*)-BenzP* (15.5 mg, 0.055 mmol, 11 mol%), B_2pin_2 (127 mg, 0.5 mmol, 1.0 equiv.) and MeOH (40.5 μL, 1.0 mmol, 2.0 equiv.). The reaction mixture was then oxidized under basic conditions following the general procedure and purified by column chromatography to afford the desired alcohol as a colorless oil (40.7 mg, 35% yield, 90:10 *er*).

TLC: Rf = 0.36 (Et_2O/pentane 1:1).

^{1}H NMR (400 MHz, $CDCl_3$) δ (ppm) = 7.37 – 7.23 (m, 4H, H7 and H11), 7.12 (d, $J_{H\text{-}H}$ = 8.1 Hz, 2H, H12), 6.93 – 6.88 (m, 2H, H6), 5.58 (s, 1H, H1), 5.22 (s, 1H, H1), 4.18 – 4.02 (m, 2H, H4), 4.01 – 3.91 (m, 1H, H3), 3.76 (s, 3H, H9), 2.29 (s, 3H, H14), 1.70 (s, 1H, H15).

^{13}C{^{1}H} NMR (100 MHz, $CDCl_3$) δ (ppm) = 158.7 (C8), 148.5 (C2), 139.0 (C10), 137.4 (C5 or C13), 132.1 (C5 or C13), 129.5 (C7), 129.1 (C12), 126.5 (C11), 114.2 (C6), 113.1 (C1), 65.93 (C4), 55.3 (C9), 51.9 (C3), 21.18 (C14).

HRMS: calculated for $C_{18}H_{20}O_2$ $[M+Na]^+$: 291.1356; found: 291.1357

IR (neat) ν (cm^{-1}): 2836, 2248, 1608, 1509, 1246, 1178, 1031, 906, 831, 728, 607, 553.

HPLC: 90:10 *er*, chiral stationary column: AD-H, mobile phase: hexane/iPrOH = 95/5, 1.0 mL/min, 254 nm, 30 °C, t(minor) = 21.59 min, t(major) = 22.86 min.

[α]$^{20}_{D}$ = –20.1 (c 0.52, $CHCl_3$).

(*R*)-3-(4-methoxyphenyl)-2-(p-tolyl)but-3-en-1-ol (2'n) (major)

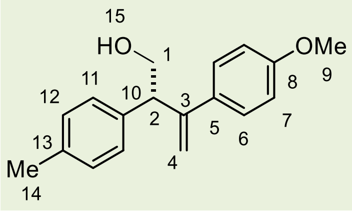

Following the general procedure using 1-methoxy-4-(3-(p-tolyl)buta-1,3-dien-2-yl)benzene (125.0 mg, 0.5 mmol, 1.0 equiv.), CuCl (4.95 mg, 0.05 mmol, 10 mol%), KO*t*Bu (50 μL, 0.05 mmol, 10 mol%, 1.0 M in THF), (*R*,*R*)-BenzP* (15.5 mg, 0.055 mmol, 11 mol%), B_2pin_2 (127 mg, 0.5 mmol, 1.0 equiv.) and MeOH (40.5 μL, 1.0 mmol, 2.0 equiv.). The reaction mixture was then oxidized under basic conditions following the general procedure and purified by column chromatography to afford the desired alcohol as a colorless oil (69.1 mg, 52% yield, 88:12 *er*).

TLC: Rf = 0.36 (Et_2O/pentane 1:1).

^{1}H NMR (400 MHz, $CDCl_3$) δ (ppm) = 7.37 – 7.23 (m, 4H, H7 and H11), 7.17 (dd, $J_{H\text{-}H}$ = 19.6, 8.0 Hz, 2H, H12), 6.87 – 6.82 (m, 2H, H6), 5.57 (s, 1H, H4), 5.21 (s, 1H, H4), 4.18 – 4.02 (m, 2H, H1), 4.01 – 3.91 (m, 1H, H2), 3.75 (s, 3H, H9), 2.30 (s, 3H, H14), 1.70 (s, 1H, H15).

$^{13}C\{^{1}H\}$ NMR (100 MHz, $CDCl_3$) δ (ppm) = 159.2 (C8), 147.7 (C3), 137.1 (C10), 136.7 (C5 or C13), 134.4 (C5 or C13), 129.6 (C12), 128.4 (C11), 127.7 (C7), 113.7 (C6), 112.4 (C4), 65.97 (C1), 55.3 (C9), 52.4 (C2), 21.15 (C14).

HRMS: calculated for $C_{18}H_{20}O_2$ $[M+Na]^+$: 291.1356; found: 291.1357.

IR (neat) ν (cm^{-1}): 2836, 2248, 1608, 1509, 1246, 1178, 1031, 906, 831, 728, 607, 553.

HPLC: 88:12 *er*, chiral stationary column: AD-H, mobile phase: hexane/iPrOH = 95/5, 1.0 mL/min, 254 nm, 30 °C, t(major) = 19.15 min, t(minor) = 20.27 min.

$[\alpha]^{20}_{D}$ = –20.1 (c 0.52, $CHCl_3$).

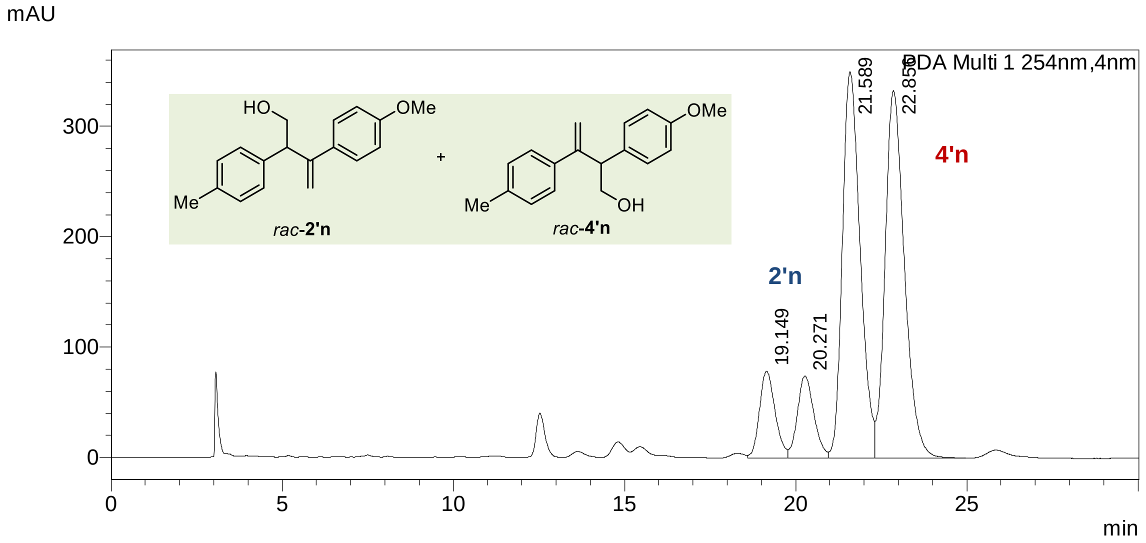

<Peak Table>

PDA Ch1 254nm

Peak#	Ret. Time	Area%	
1	19.149	8.197	**2'n**
2	20.271	8.152	
3	21.589	41.153	**4'n**
4	22.856	42.498	
Total		100.000	

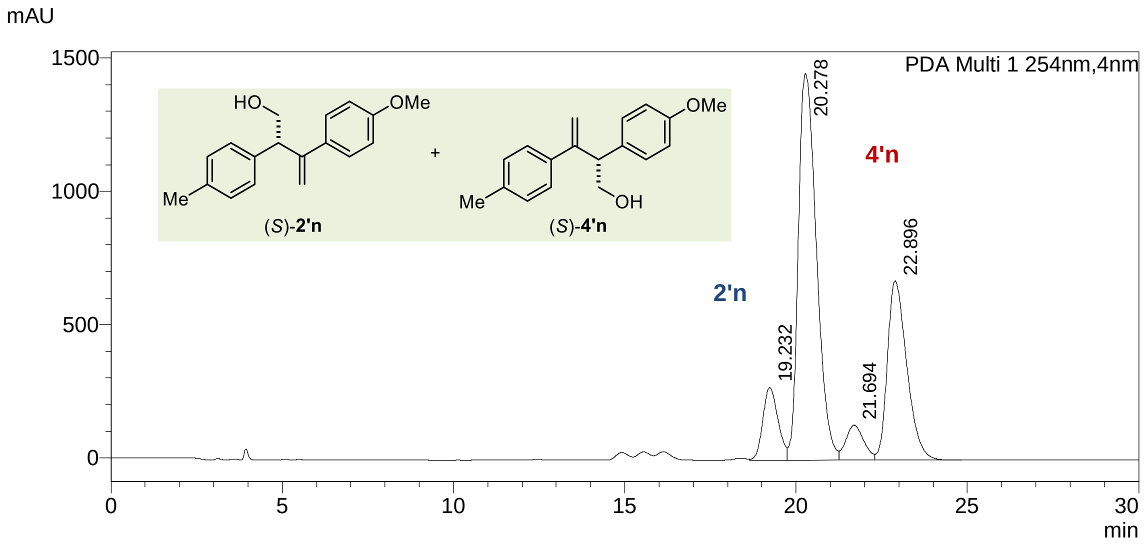

<Peak Table>
PDA Ch1 254nm

Peak#	Ret. Time	Area%
1	19.232	11.898
2	20.278	88.102
Total		100.000

<Peak Table>
PDA Ch1 254nm

Peak#	Ret. Time	Area%
1	21.694	9.964
2	22.896	90.036
Total		100.000

(*R*)-3-methyl-2-phenylbut-3-en-1-ol (2'o)

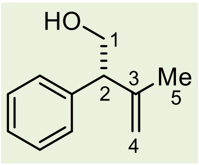

Following the general procedure using (3-methylbuta-1,3-dien-2-yl)benzene (72.0 mg, 0.5 mmol, 1.0 equiv.), CuCl (4.95 mg, 0.05 mmol, 10 mol%), KO*t*Bu (50 µL, 0.05 mmol, 10 mol%, 1.0 M in THF), (*R*,*R*)-BenzP* (15.5 mg, 0.055 mmol, 11 mol%), B_2pin_2 (127 mg, 0.5 mmol, 1.0 equiv.) and MeOH (40.5 µL, 1.0 mmol, 2.0 equiv.). The reaction mixture was then oxidized under basic conditions following the general procedure and purified by column chromatography to afford the desired alcohol as a colorless oil (74.5 mg, 92% yield, 83:17 *er*). The absolute configuration of this compound is known.[2]

TLC: Rf = 0.4 (Et_2O/pentane 1:2).

^{1}H NMR (300 MHz, $CDCl_3$) δ (ppm) 7.39 – 7.30 (m, 2H), 7.29 – 7.22 (m, 2H), 5.04 – 5.02 (m, 1H), 4.97 – 4.95 (m, 1H), 4.01 (dd, $^3J_{HH}$ = 10.9, 7.8 Hz, 1H), 3.88 (dd, $^3J_{HH}$ = 10.9, 6.8 Hz, 1H), 3.49 (t, $^3J_{HH}$ = 7.3 Hz, 1H), 1.67 (s, 3H), 1.53 (s, 1H).

HRMS: calculated for $C_{11}H_{14}O$ $[M+H]^+$: 163.1118; found: 163.1105.

^{13}C{^{1}H} NMR (75 MHz, $CDCl_3$) δ (ppm) = 145.2, 140.2, 128.8, 128.3, 127.1, 111.8, 64.6, 55.2, 22.1.

IR (neat) ν (cm^{-1}): 3342, 2926, 1645, 1492, 1373, 1182, 861, 779, 753, 738, 697, 628.

HPLC: 83:17 *er*, chiral stationary column: AD-H, mobile phase: hexane/*i*PrOH = 99/1, 1.0 mL/min, 220 nm, 30 °C, t(minor) = 18.60 min, t(major) = 21.10 min.

$[\alpha]^{20}_D$ = –18.35 (c 0.3, $CHCl_3$).

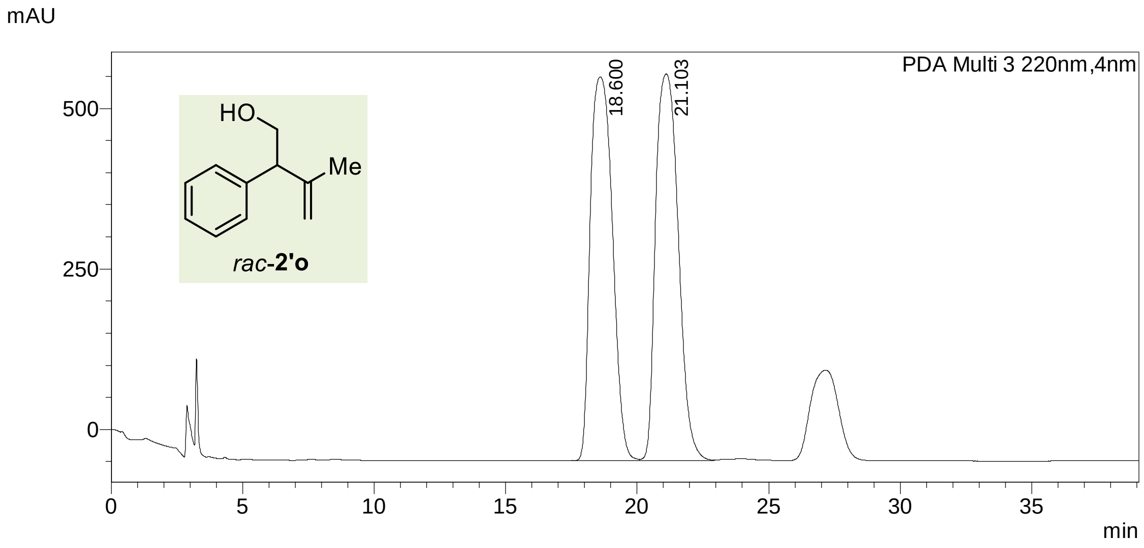

<Peak Table>

PDA Ch3 220nm

Peak#	Ret. Time	Area%
1	18.600	49.127
2	21.103	50.873
Total		100.000

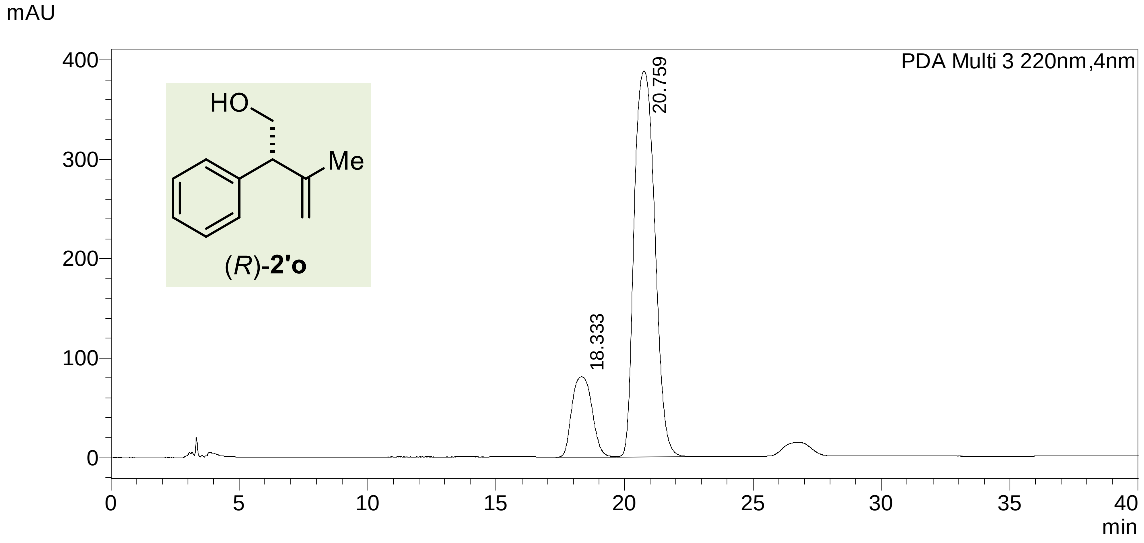

<Peak Table>

PDA Ch3 220nm

Peak#	Ret. Time	Area%
1	18.333	16.916
2	20.759	83.084
Total		100.000

(*R*)-2-(6-methoxypyridin-3-yl)-3-methylbut-3-en-1-ol (**2'p**)

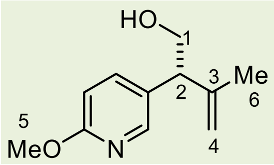

Following the general procedure using 2-methoxy-5-(3-methylbuta-1,3-dien-2-yl)pyridine (88.0 mg, 0.5 mmol, 1.0 equiv.), CuCl (4.95 mg, 0.05 mmol, 10 mol%), KO*t*Bu (50 µL, 0.05 mmol, 10 mol%, 1.0 M in THF), (*R*,*R*)-BenzP* (15.5 mg, 0.055 mmol, 11 mol%), B_2pin_2 (127 mg, 0.5 mmol, 1.0 equiv.) and MeOH (40.5 µL, 1.0 mmol, 2.0 equiv.). The reaction mixture was then oxidized under basic conditions following the general procedure and purified by column chromatography to afford the desired alcohol as a colorless liquid (79.2 mg, 82% yield, 74:26 *er*)

TLC: Rf = 0.32 (Et_2O/pentane 1:1).

^{1}H NMR (300 MHz, $CDCl_3$) δ (ppm) = 8.03 (d, $^3J_{HH}$ = 2.5 Hz, 1H), 7.44 (dd, $^3J_{HH}$ = 8.5, 2.5 Hz, 1H), 6.76 – 6.67 (m, 1H), 5.05 – 5.00 (m, 1H), 4.93 (d, $^3J_{HH}$ = 1.1 Hz, 1H), 4.01 – 3.94 (m, 1H), 3.92 (s, 3H), 3.86 – 3.80 (m, 1H), 3.41 (t, $^3J_{HH}$ = 7.2 Hz, 1H), 1.66 (s, 3H), 1.57 (s, 1H).

^{13}C{^{1}H} NMR (75 MHz, $CDCl_3$) δ (ppm) = 163.5, 146.5, 144.6, 138.4, 112.3, 111.0, 64.4, 53.5, 51.8, 22.0.

HRMS: calculated for $C_{11}H_{15}NO_2$ $[M+H]^+$: 194.1176; found: 194.1179.

IR (neat) ν (cm^{-1}): 3367, 1646, 1465, 1257, 1154, 1110, 948, 884, 768, 670, 609, 550, 522.

HPLC: 74:26 *er*, chiral stationary column: OZ-H, mobile phase: hexane/*i*PrOH = 95/5, 1.0 mL/min, 254 nm, 30 °C, t(minor) = 11.49 min, t(major) = 13.51 min.

[α]$^{20}_{D}$ = –11.17 (c 0.7, $CHCl_3$).

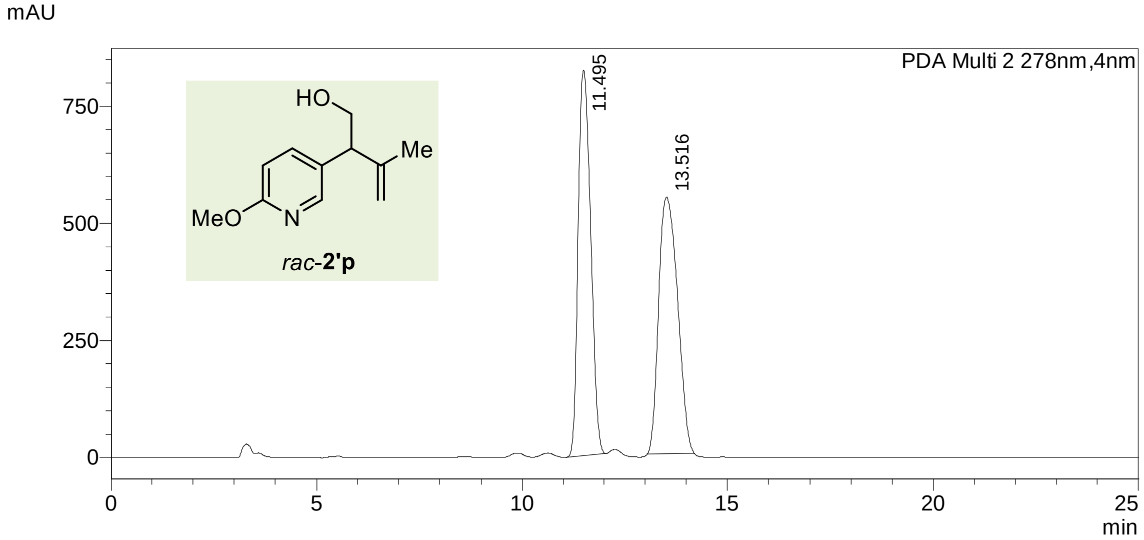

<Peak Table>

PDA Ch2 278nm

Peak#	Ret. Time	Area%
1	11.495	49.910
2	13.516	50.090
Total		100.000

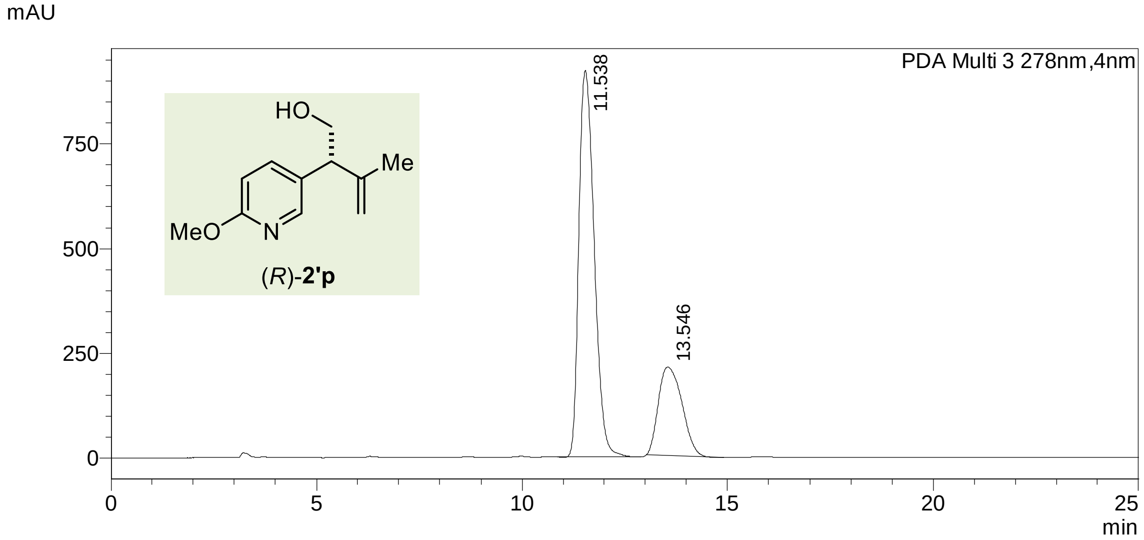

<Peak Table>

PDA Ch3 278nm

Peak#	Ret. Time	Area%
1	11.538	73.938
2	13.546	26.062
Total		100.000

(*R*)-3-methyl-2-(4-(trifluoromethyl)phenyl)but-3-en-1-ol (**2'q**)

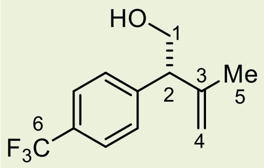

Following the general procedure using 1-(3-methylbuta-1,3-dien-2-yl)-4-(trifluoromethyl)benzene (106 mg, 0.5 mmol, 1.0 equiv.), CuCl (4.95 mg, 0.05 mmol, 10 mol%), KO*t*Bu (50 µL, 0.05 mmol, 10 mol%, 1.0 M in THF), (*R*,*R*)-BenzP* (15.5 mg, 0.055 mmol, 11 mol%), B_2pin_2 (127 mg, 0.5 mmol, 1.0 equiv.) and MeOH (40.5 µL, 1.0 mmol, 2.0 equiv.). The reaction mixture was then oxidized under basic conditions following the general procedure and purified by column chromatography to afford the desired alcohol as a colorless liquid (94.3 mg, 82% yield, 88:12 *er*)

TLC: Rf = 0.50 (Et_2O/pentane 1:1).

^{1}H NMR (400 MHz, $CDCl_3$) δ (ppm) = 7.58 (d, $^3J_{HH}$ = 8.0 Hz, 2H), 7.37 (d, $^3J_{HH}$ = 8.0 Hz, 2H), 5.09 – 5.03 (m, 1H), 4.96 (d, $^3J_{HH}$ = 1.1 Hz, 1H), 4.02 (dt, $^3J_{HH}$ = 7.4, 5.4 Hz, 1H), 3.89 (dt, $^3J_{HH}$ = 10.8, 6.5 Hz, 1H), 3.55 (t, $^3J_{HH}$ = 7.2 Hz, 1H), 1.65 (s, 3H), 1.50 (s, 1H).

^{13}C{^{1}H} NMR (100 MHz, $CDCl_3$) δ (ppm) = 144.5, 144.3, 129.3, 128.6, 125.6, 122.9, 112.7, 64.3, 54.9, 22.1.

^{19}F{^{1}H} NMR ($CDCl_3$, 282 MHz) δ (ppm) = –62.47.

LRMS: calculated for $C_{12}H_{13}F_3O$ $[M+NH_4]^+$: 248.2; found: 248.6.

IR (neat) ν (cm^{-1}): 3348, 1647, 1376, 1066, 954, 897, 864, 796, 770, 702, 604, 539, 479.

HPLC: 88:12 *er*, chiral stationary column: OZ-H, mobile phase: hexane/*i*PrOH = 95/5, 1.0 mL/min, 254 nm, 30 °C, t(major) = 4.98 min, t(minor) = 7.21 min.

[α]$^{20}_{D}$ = –41.55 (c 0.71, $CHCl_3$).

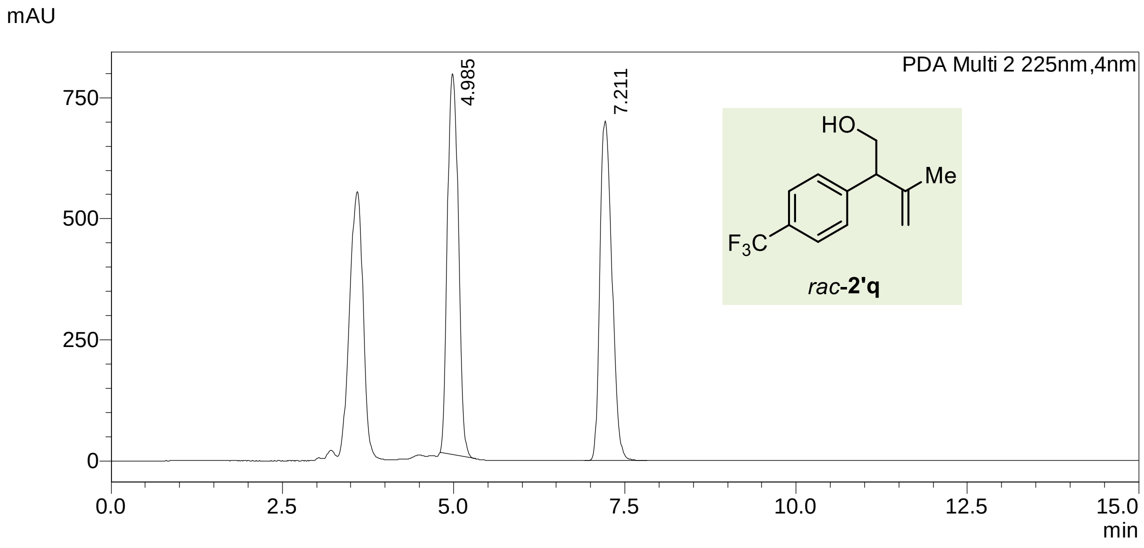

<Peak Table>

PDA Ch2 225nm

Peak#	Ret. Time	Area%
1	4.985	50.555
2	7.211	49.445
Total		100.000

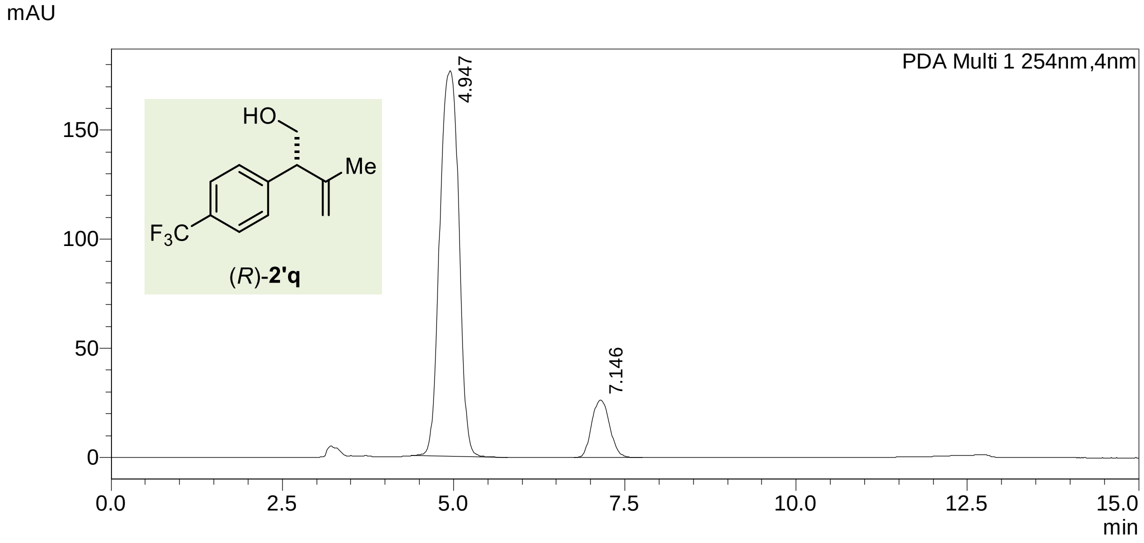

<Peak Table>

PDA Ch1 254nm

Peak#	Ret. Time	Area%
1	4.947	87.769
2	7.146	12.231
Total		100.000

(*R*)-3-methylene-2,5-diphenylpentan-1-ol (2'r)

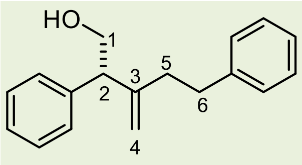

Following the general procedure using (3-methylenepent-4-ene-1,4-diyl)dibenzene (117.7 mg, 0.5 mmol, 1.0 equiv.), CuCl (4.95 mg, 0.05 mmol, 10 mol%), KO*t*Bu (50 μL, 0.05 mmol, 10 mol%, 1.0 M in THF), (*R*,*R*)-BenzP* (15.5 mg, 0.055 mmol, 11 mol%), B_2pin_2 (127 mg, 0.5 mmol, 1.0 equiv.) and MeOH (40.5 μL, 1.0 mmol, 2.0 equiv.). The reaction mixture was then oxidized under basic conditions following the general procedure and purified by column chromatography to afford the desired alcohol as a white solid (93.2 mg, 74% yield, 75:25 er_2, 9.2 :1 $rr_{2/4}$).

TLC: Rf = 0.36 (Et_2O/pentane 1:1).

Mp: 32 –34 °C.

^{1}H NMR (400 MHz, $CDCl_3$) δ (ppm) 7.38 – 7.31 (m, 2H), 7.29 – 7.25 (m, 2H), 7.12 (dd, $^3J_{HH}$ = 7.0, 1.7 Hz, 2H), 5.12 (d, $^3J_{HH}$ = 1.2 Hz, 1H), 5.06 (s, 1H), 4.05 – 3.99 (m, 1H), 3.91 – 3.85 (m, 1H), 3.55 (t, $^3J_{HH}$ = 7.2 Hz, 1H), 2.79 – 2.67 (m, 2H), 2.30 – 2.23 (m, 2H), 1.48 (s, 1H).

^{13}C{^{1}H} NMR (100 MHz, $CDCl_3$) δ (ppm) = 148.5, 141.9, 140.2, 128.8, 128.54, 128.50, 128.45, 127.2, 126.0, 110.8, 65.1, 54.1, 37.5, 34.4.

HRMS: calculated for $C_{18}H_{20}O$ $[M+Na]^+$: 275.1407; found: 275.1417.

IR (neat) ν (cm^{-1}): 3420, 2972, 2901, 1452, 1406, 1394, 1072, 1026, 749, 696, 618, 605.

HPLC: 75:25 *er*, chiral stationary column: AD-H, mobile phase: hexane/*i*PrOH = 99/1, 1.0 mL/min, 210 nm, 30 °C, t(minor) = 31.27 min, t(major) = 33.84 min.

[α]$^{20}_{D}$ = –35.76 (c 0.45, $CHCl_3$).

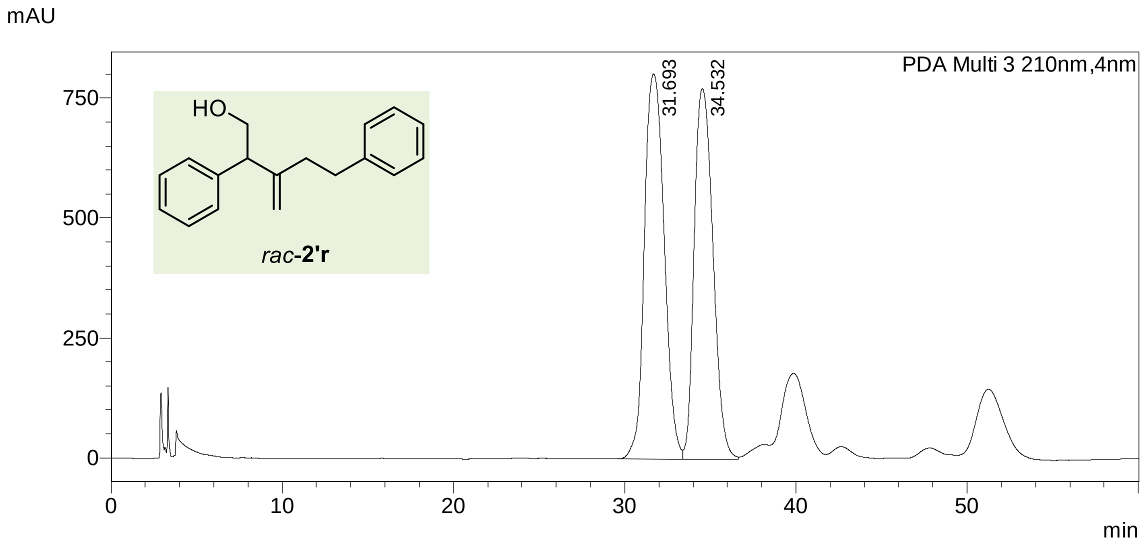

<Peak Table>

PDA Ch3 210nm

Peak#	Ret. Time	Area%
1	31.693	52.279
2	34.532	47.721
Total		100.000

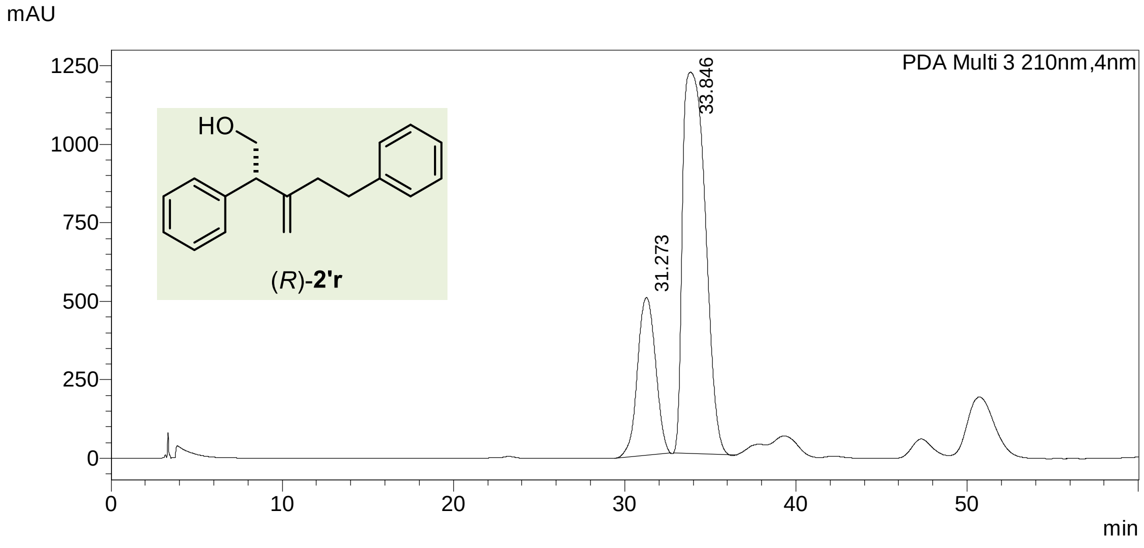

<Peak Table>

PDA Ch3 210nm

Peak#	Ret. Time	Area%
1	31.273	24.837
2	33.846	75.163
Total		100.000

(*R*)-3-cyclohexyl-2-phenylbut-3-en-1-ol (2's)

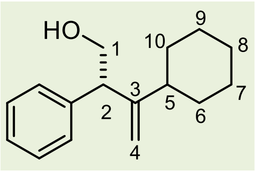

Following the general procedure using (3-cyclohexylbuta-1,3-dien-2-yl)benzene (106.1 mg, 0.5 mmol, 1.0 equiv.), CuCl (4.95 mg, 0.05 mmol, 10 mol%), KO*t*Bu (50 µL, 0.05 mmol, 10 mol%, 1.0 M in THF), (*R*,*R*)-BenzP* (15.5 mg, 0.055 mmol, 11 mol%), B_2pin_2 (127 mg, 0.5 mmol, 1.0 equiv.) and MeOH (40.5 µL, 1.0 mmol, 2.0 equiv.). The reaction mixture was then oxidized under basic conditions following the general procedure and purified by column chromatography to afford the desired alcohol as a colorless liquid (46.2 mg, 40% yield, 91:9 *er*)

TLC: Rf = 0.68 (Et_2O/pentane 1:1).

^{1}H NMR (400 MHz, $CDCl_3$) δ (ppm) 7.41 – 7.11 (m, 5H), 5.10 (s, 1H), 4.96 (s, 1H), 3.95 – 3.89 (m, 1H), 3.83 – 3.77 (m, 1H), 3.58 (t, $^3J_{HH}$ = 7.3 Hz, 1H), 1.81 – 1.39 (m, 8H), 1.33 – 0.95 (m, 4H).

^{13}C{^{1}H} NMR (100 MHz, $CDCl_3$) δ (ppm) = 154.8, 140.7, 128.7, 128.5, 127.0, 108.9, 65.7, 53.2, 44.6, 33.5, 32.6, 27.0, 26.8, 26.4.

HRMS: calculated for $C_{16}H_{22}O$ $[M+Na]^+$: 253.1563; found: 253.1550.

IR (neat) ν (cm^{-1}): 3344, 3062, 3027, 2923, 2851, 1639, 16001492, 1449, 1384, 1261, 1062, 1046, 1018, 886, 755, 698, 671.

HPLC: 91:9 *er*, chiral stationary column: AD-H, mobile phase: hexane/*i*PrOH = 97/3, 1.0 mL/min, 254 nm, 30 °C, t(minor) = 9.55 min, t(major) = 10.59 min.

[α]$^{20}_{D}$ = –32.76 (c 0.25, $CHCl_3$).

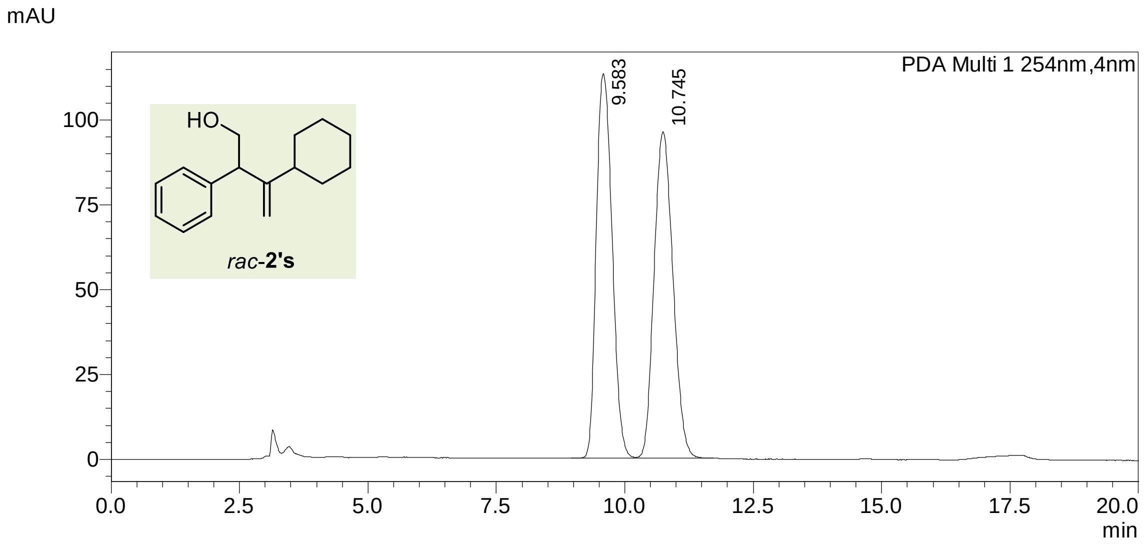

<Peak Table>

PDA Ch1 254nm

Peak#	Ret. Time	Area%
1	9.583	49.648
2	10.745	50.352
Total		100.000

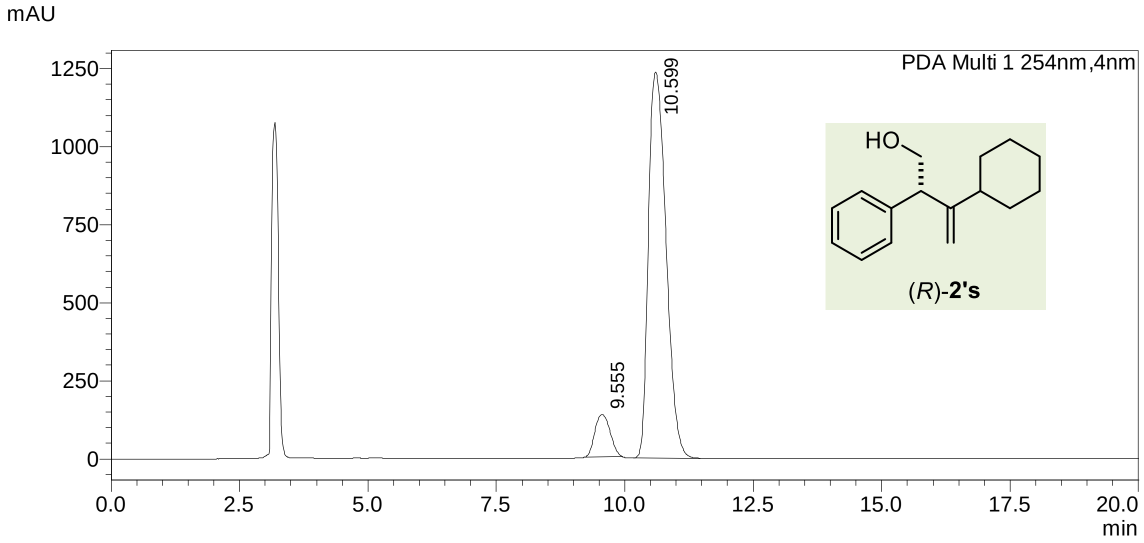

<Peak Table>

PDA Ch1 254nm

Peak#	Ret. Time	Area%
1	9.555	8.715
2	10.599	91.285
Total		100.000

5. Large scale experiment

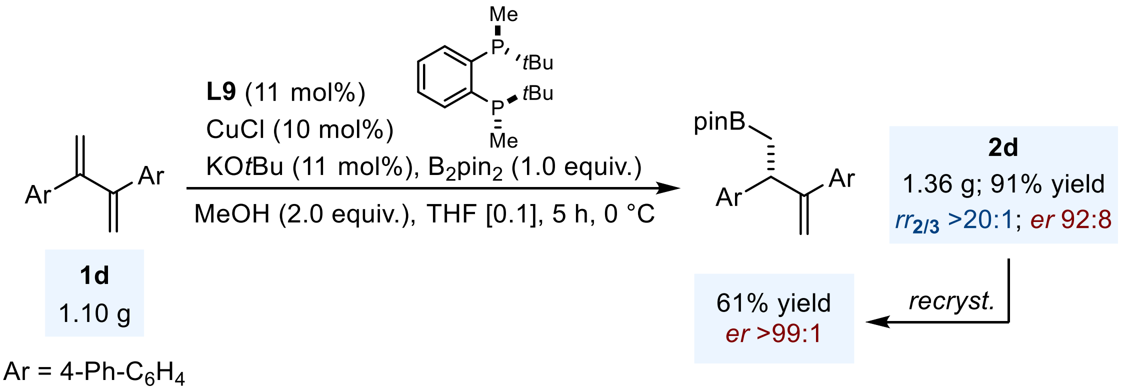

In a glove box, CuCl (30.4 mg, 0.153 mmol, 10 mol%), (*R*,*R*)-BenzP* (95.3 mg, 0.169 mmol, 11 mol%) were introduced into a Schlenk tube and dissolved in THF (10.0 mL). After stirring at room temperature for 10 min., B_2pin_2 (779 mg, 3.07 mmol, 1.0 equiv.) and a THF solution of KO*t*Bu (0.3 mL, 0.05 mmol, 10 mol%, 1.0 M in THF) were added. The reaction mixture was cooled to 0 °C. After stirring for 10 min., the solution was slowly added to a suspension of **1d** (1.1 g, 3.7 mmol) in THF (20 mL) at 0 °C, followed by dropwise addition of MeOH (6.14 mmol, 2.0 equiv.). After stirring at 0 °C for 5 h, the reaction was filtered through a short pad of Celite®, washed with diethyl ether (3×30 mL) and the solvent was removed under vacuum. The residue was purified by column chromatography (pentane/ethyl acetate 30:1) to afford boronic ester **2d** as a light-yellow solid (91% yield, 1.3 g). The enantiomeric excess was measured by HPLC after oxidation to alcohol **2'd**. The enantiopurity of **2d** could be improved by recrystallization from MeOH/$CHCl_3$ (10:1, v/v) (0.8 g, 61% yield, *er* >99:1).

(*S*)-2-(2,3-di([1,1'-biphenyl]-4-yl)but-3-en-1-yl)-4,4,5,5-tetramethyl-1,3,2-dioxaborolane (2d)

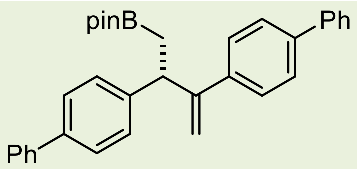

TLC: Rf = 0.6 (Et_2O/pentane 1:10).

M.P.: 127.5-128.5 ˚C.

^{1}H NMR (400 MHz, $CDCl_3$) δ (ppm) 7.59 – 7.54 (m, 4H), 7.51 – 7.47 (m, 4H), 7.45 – 7.30 (m, 10H), 5.45 (s, 1H), 5.32 (s, 1H), 4.26 (t, $^3J_{HH}$ = 8.2 Hz, 1H), 1.53 (dd, $^3J_{HH}$ = 15.4, 8.4 Hz, 1H), 1.41 (dd, $^3J_{HH}$ = 15.4, 8.1 Hz, 1H), 1.17 (d, $^3J_{HH}$ = 6.7 Hz, 12H).

^{13}C{^{1}H} NMR (75 MHz, $CDCl_3$) δ (ppm) = 152.9, 144.5, 141.7, 141.2, 141.0, 140.0, 138.9, 128.83, 128.79, 128.4, 127.5, 127.3, 127.08, 127.07, 126.8, 113.0, 83.3, 45.7, 24,89, 24.86.

^{11}B NMR (128 MHz, $CDCl_3$) δ 32.21.

HRMS: calculated for $C_{34}H_{35}BO_2$ $[M+H]^+$: 487.2809; found: 487.2807.

IR (neat) ν (cm^{-1}): 2974, 1485, 1366, 1325, 1269, 1143, 1077, 901, 844, 764, 737, 694.

$[\alpha]^{20}_D$ = –32.18 (c 0.64, $CHCl_3$).

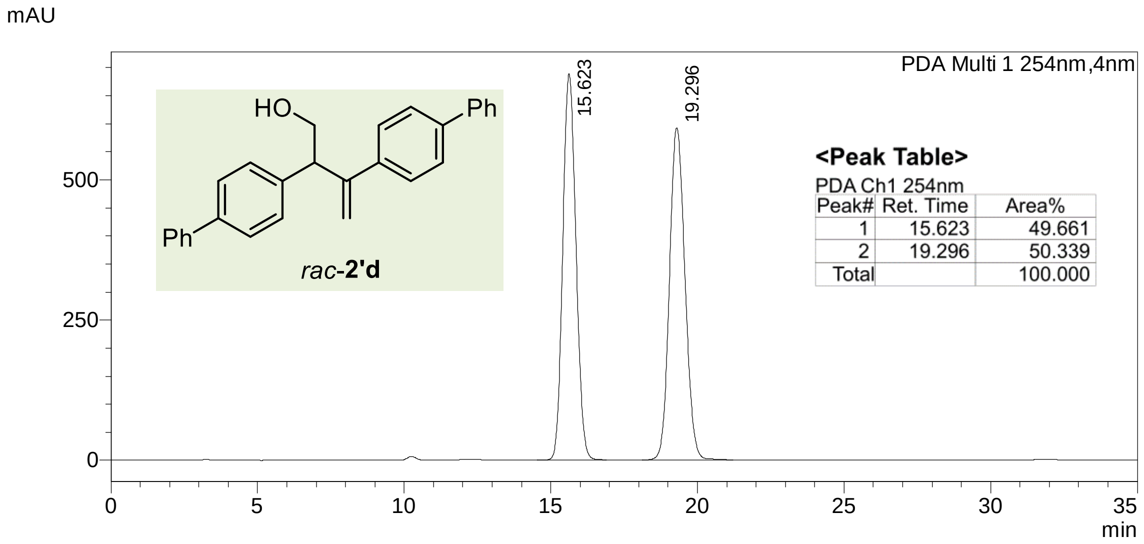

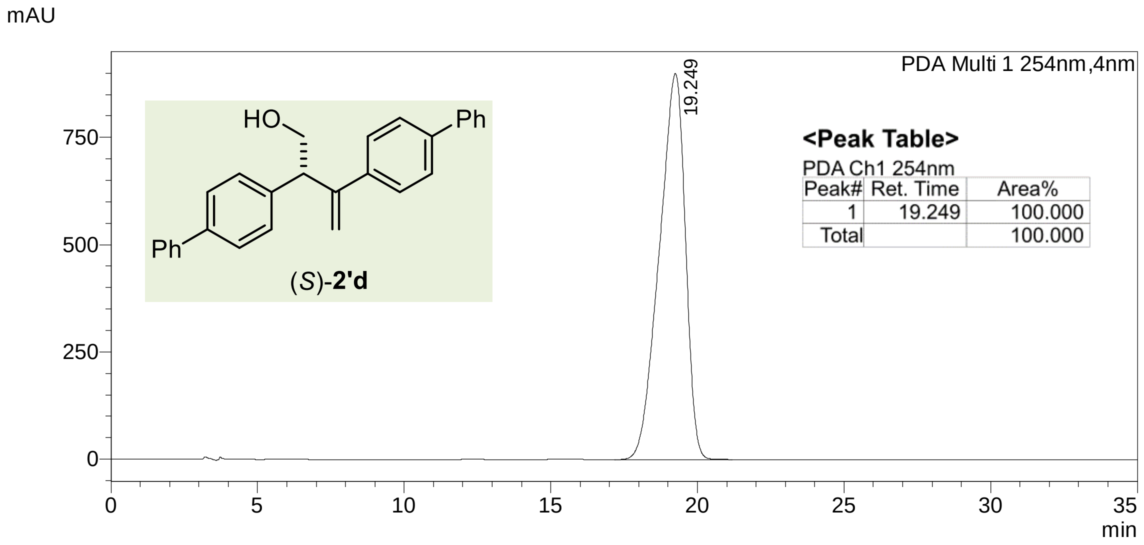

6. Mechanistic study

6.1 Measurement of the kinetic isotope effect

Inside the glove box, a Schlenk tube was charged with CuCl (0.5 mg, 0.001 mmol, 10 mol%, 0.005 M), (*R*,*R*)-BenzP* (1.55 mg, 0.0011 mmol, 11 mol%, 0.0055 M) and THF (2.0 mL). After stirring at room temperature for 10 min., B_2pin_2 (25.4 mg, 0.1 mmol, 1.0 equiv., 0.05 M) and a THF solution of KO*t*Bu (10 μL, 0.005 mmol, 10 mol%, 1.0 M in THF, 0.005 M) were

added. The reaction mixture was then cooled to 0 °C before **1a** (20.6 mg, 0.1 mmol, 1.0 equiv., 0.05 M) and MeOH or CD_3OD (0.2 mmol, 2.0 equiv., 0.1 M) were added. Aliquots (200 μL) were taken at regular intervals and quenched by dilution in $CDCl_3$ (0.4 mL). Product formation was monitored by ^{1}H NMR analysis.

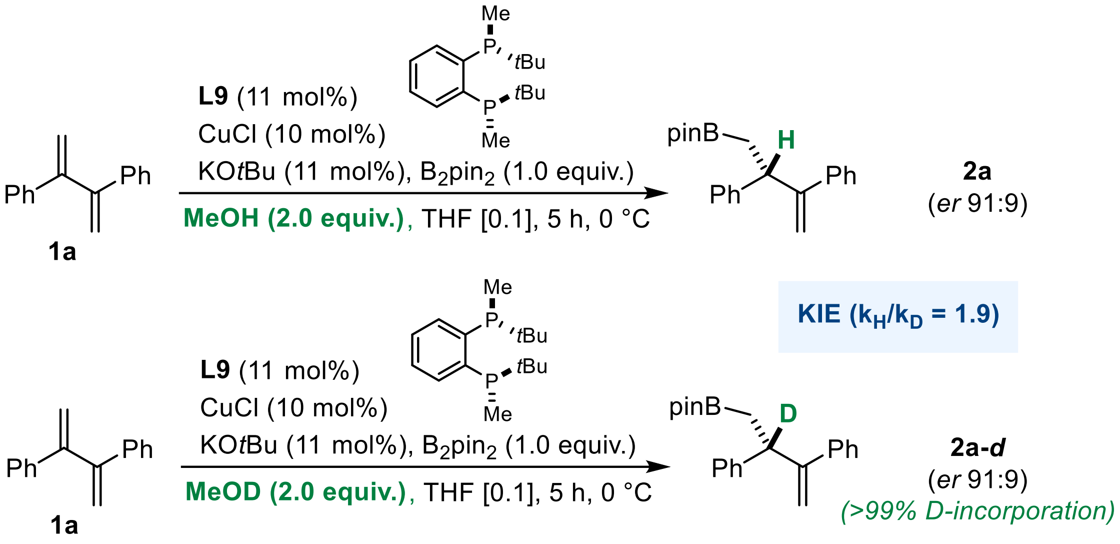

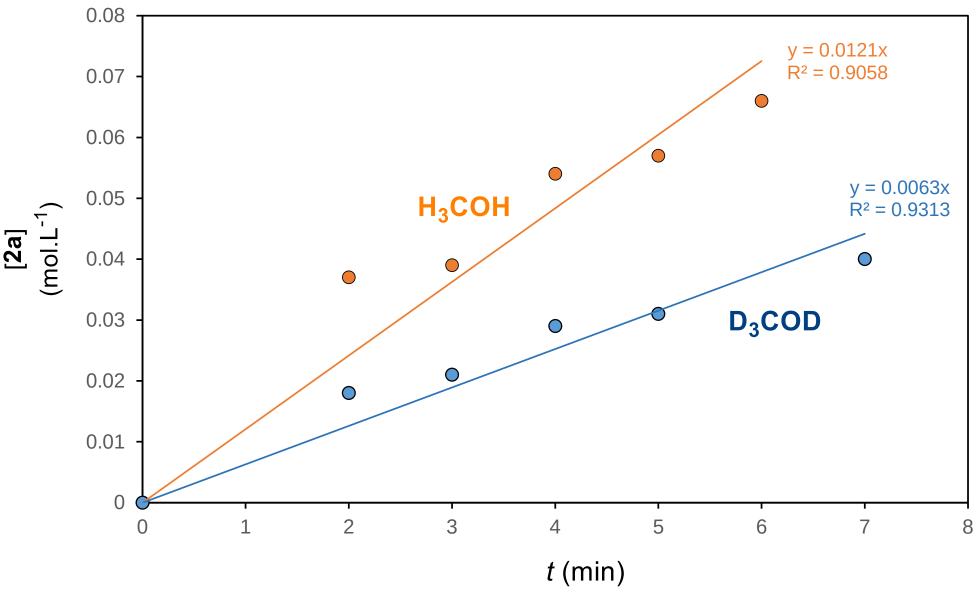

Figure S1. KIE measurement (mol/L/min)

6.2 Non-Linear Effect

Table S8[a]

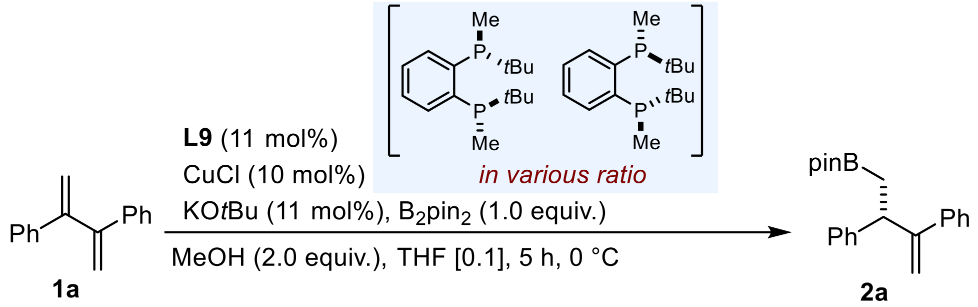

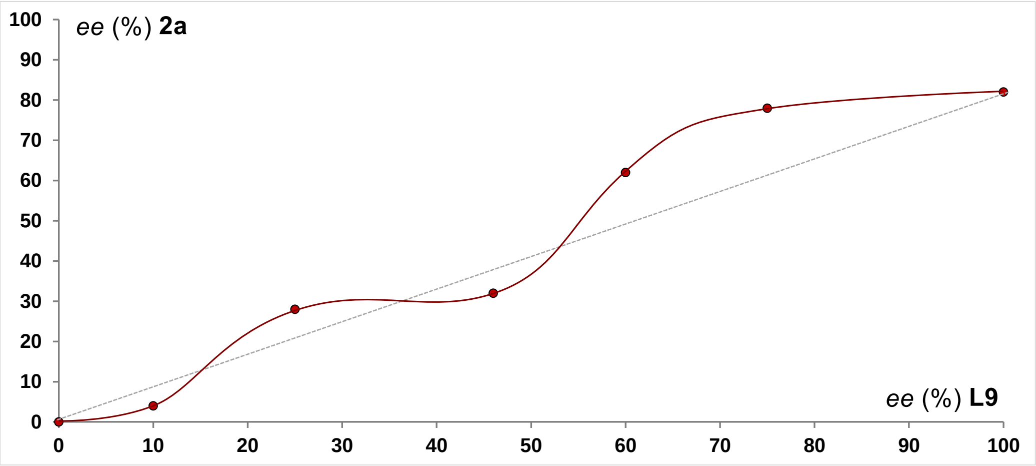

Entry	ee **L9** (%)	conv. **2a** (%)	*er* (**2a**)
1	0	92	50:50
2	10	87	53:47
3	25	87	63:37
4	50	90	67:33
5	60	92	81:19
6	75	89	90:10
7	>99	83	92:8

[a]Reaction conditions: all reactions were performed with **1a** (0.2 mmol), (*R,R*)-**L9**/CuCl (1.1:1, 10 mol%), KO*t*Bu (10 mol%), B_2pin_2 (0.20 mmol), MeOH (0.40 mmol) in THF (2 mL, 0.1 M) at 0 °C for 5 h. Average of at least two experiments.

6.3 Variable Time Normalization Analyses[4]

Reference: In a glove box, a Schlenk was charged with CuCl (0.001 mmol, 10 mol%, 0.005 M), (*R*,*R*)-BenzP* (0.0011 mmol, 11 mol%, 0.0055 M) and THF (2.0 mL). After stirring at room temperature for 10 min., B_2pin_2 (0.1 mmol, 1.0 equiv., 0.05 M) and a solution of KO*t*Bu (10 µL, 0.005 mmol, 10 mol%, 1.0 M in THF, 0.005 M) were added. The reaction mixture was cooled to 0 °C. Diene **1a** (0.1 mmol, 1.0 equiv., 0.05 M) and MeOH (0.2 mmol, 2.0 equiv., 0.1 M) were added next. Aliquots (200 µL) were taken every 30 seconds and quenched by dilution in $CDCl_3$ (0.4 mL). Product formation was monitored by ^1H NMR analysis.

Same excess: This reaction was carried out according to the above procedure, with the following concentrations: [**CuCl**] = 0.005 M; [**(*R*,*R*)-BenzP***] = 0.0055 M; [**KO*t*Bu**] = 0.005 M; [**1a**] = 0.0250 M; [**B_2Pin_2**] = 0.025 M; [**MeOH**] = 0.1 M.

Order in catalyst: This reaction was carried out according to the above procedure, with the following concentrations: [**CuCl**] = 0.0025 M (5.0 mol%); [**(*R*,*R*)-BenzP***] = 0.00275 M (5.5 mol%); [**KO*t*Bu**] = 0.0025 M (5.0 mol%); [**1a**] = 0.05 M; [**B_2Pin_2**] = 0.05 M; [**MeOH**] = 0.1 M.

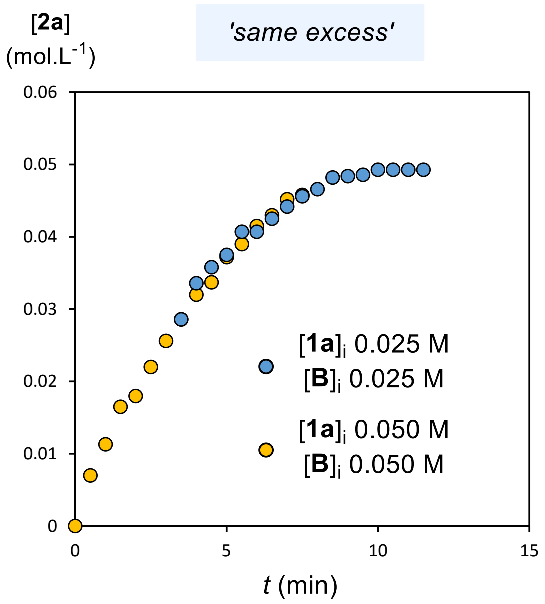

Figure S2. Same excess experiments

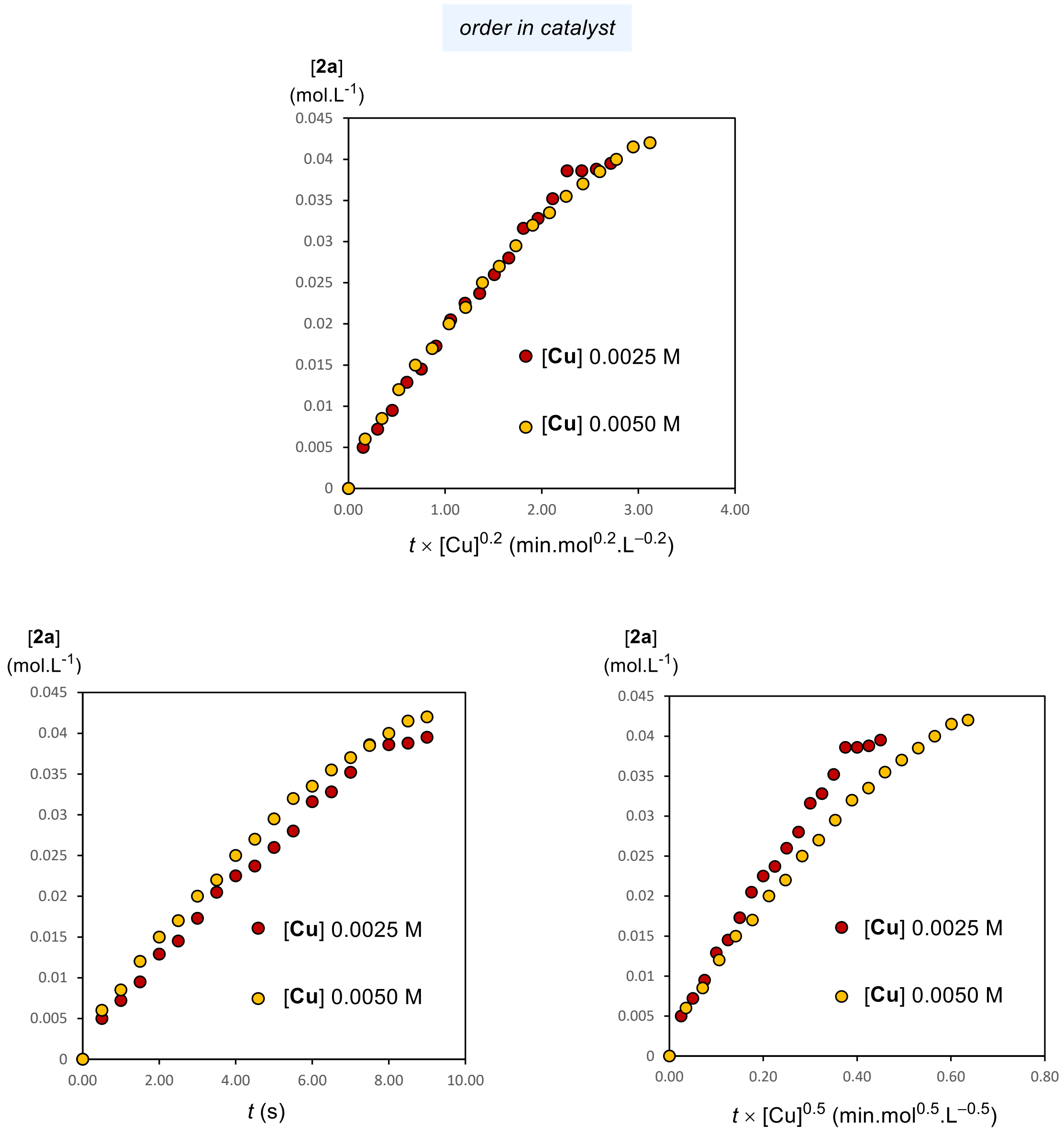

Figure S3. Order in catalyst and alternative overlays (n = 0 and n = 0.5)

7. References

1 (a) Araki, S.; Ohmura, M.; Butsugan, Y. A Facile Synthesis of 2, 3-Dialkyl-1,3-Butadienes. *Synthesis* **1985**, *10*, 963–964. (b) Fiorito, D.; Folliet, S.; Liu, Y. B.; Mazet, C. A General Nickel-Catalyzed Kumada Vinylation for the Preparation of 2-Substituted 1,3-Dienes. *ACS Catal*. **2018**, *8*, 1392 –1398. (c) Guo, H.; Zhang, S.; Li, Y.; Yu, X.; Feng, X.; Yamamoto, Y.; Bao, M. Palladium-Catalyzed Tail-to-Tail Reductive Dimerization of Terminal Alkynes to 2,3-Dibranched Butadienes. *Angew. Chem. Int. Ed*. **2022**, *61*, e202116870. (d) Sakuragi, S.; Akiba, T.; Tanahashi, T.; Fujihara, T. Synthesis of Cyclic Allylborates from 1,3-Dienes and a Diboron Reagent. *Angew. Chem. Int. Ed.* **2022**, *61*, e202202226.

2 Chen, Y.-L.; Hoppe, D. Copper-Catalyzed Asymmetric Conjugate Addition of Grignard Reagents to 1-(*N*,*N*-Diisopropylcarbamoyloxy)-1-tosyl-1-alkenes. *Tetrahedron: Asymmetry* **2009**, *20*, 1561–1567.

3 Cinderella, A. P.; Vulovic, B.; Watson, D. A. Palladium-Catalyzed Cross-Coupling of Silyl Electrophiles with Alkylzinc Halides: A Silyl-Negishi Reaction. *J. Am. Chem. Soc.* **2017**, *139*, 7741– 7744.

4 (a) Burés, J. A Simple Graphical Method to Determine the Order in Catalyst. *Angew. Chem. Int. Ed.* **2016**, *55*, 2028–2031. (b) Burés, J. Variable Time Normalization Analysis: General Graphical Elucidation of Reaction Orders from Concentration Profiles. *Angew. Chem. Int. Ed.* **2016**, *55*, 16084–16321. (c) Nielsen, C. D.-T.; Burés, J. Visual kinetic analysis. *Chem. Sci.* **2019**, *10*, 348–353. (d) Martínez- Carrión, A.; Howlett, M. G.; Alamillo- Ferrer, C.; Clayton, A. D.; Bourne, R. A.; Codina, A.; Vidal- Ferran, A.; Adams, R. W.; Burés, J. Kinetic Treatments for Catalyst Activation and Deactivation Processes based on Variable Time Normalization Analysis. *Angew. Chem. Int. Ed.* **2019**, *58*, 10189–10193. (e) Alamillo-Ferrer, C.; Hutchinson, G.; Burés, J. Mechanistic Interpretation of Orders in Catalyst Greater than One. *Nat. Rev. Chem.* **2022**, *7*, 26–34.

8. NMR Spectra

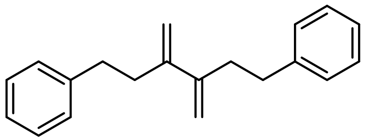

1j

^{1}H NMR (400 MHz, $CDCl_3$)

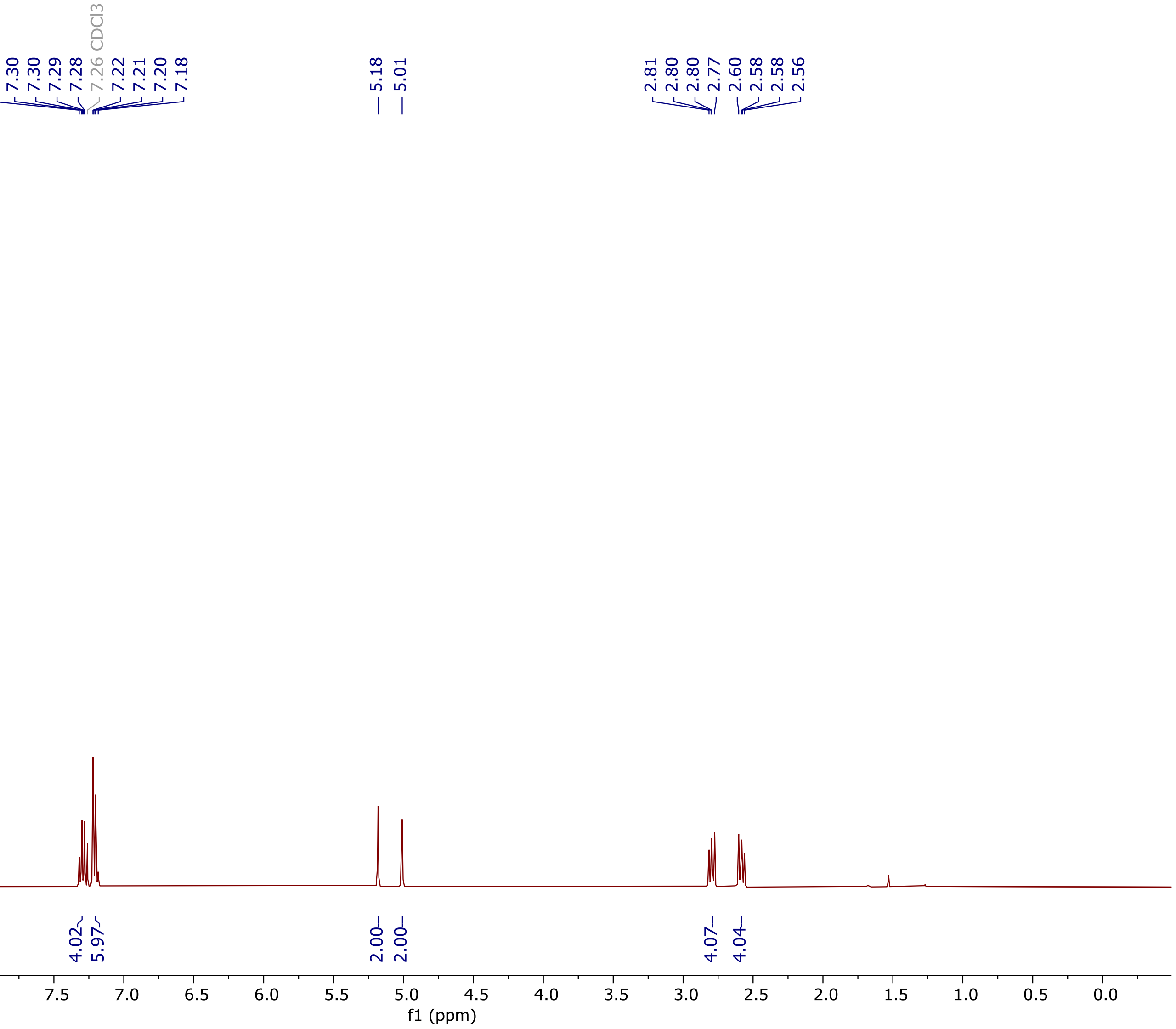

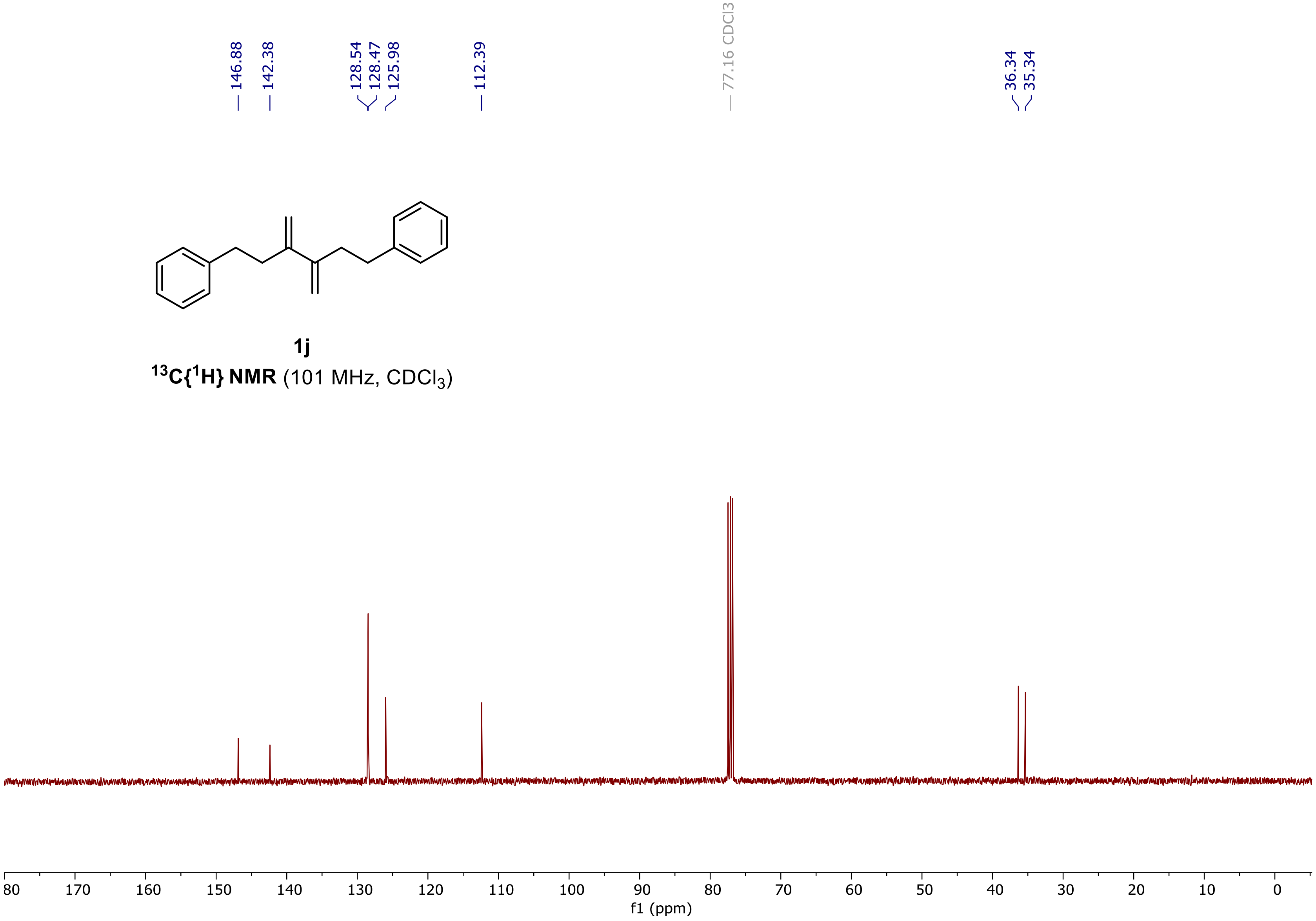

1j

$^{13}C\{^{1}H\}$ NMR (101 MHz, $CDCl_3$)

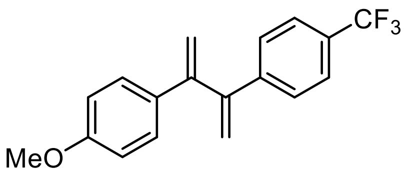

1l

^{1}H NMR (300 MHz, $CDCl_3$)

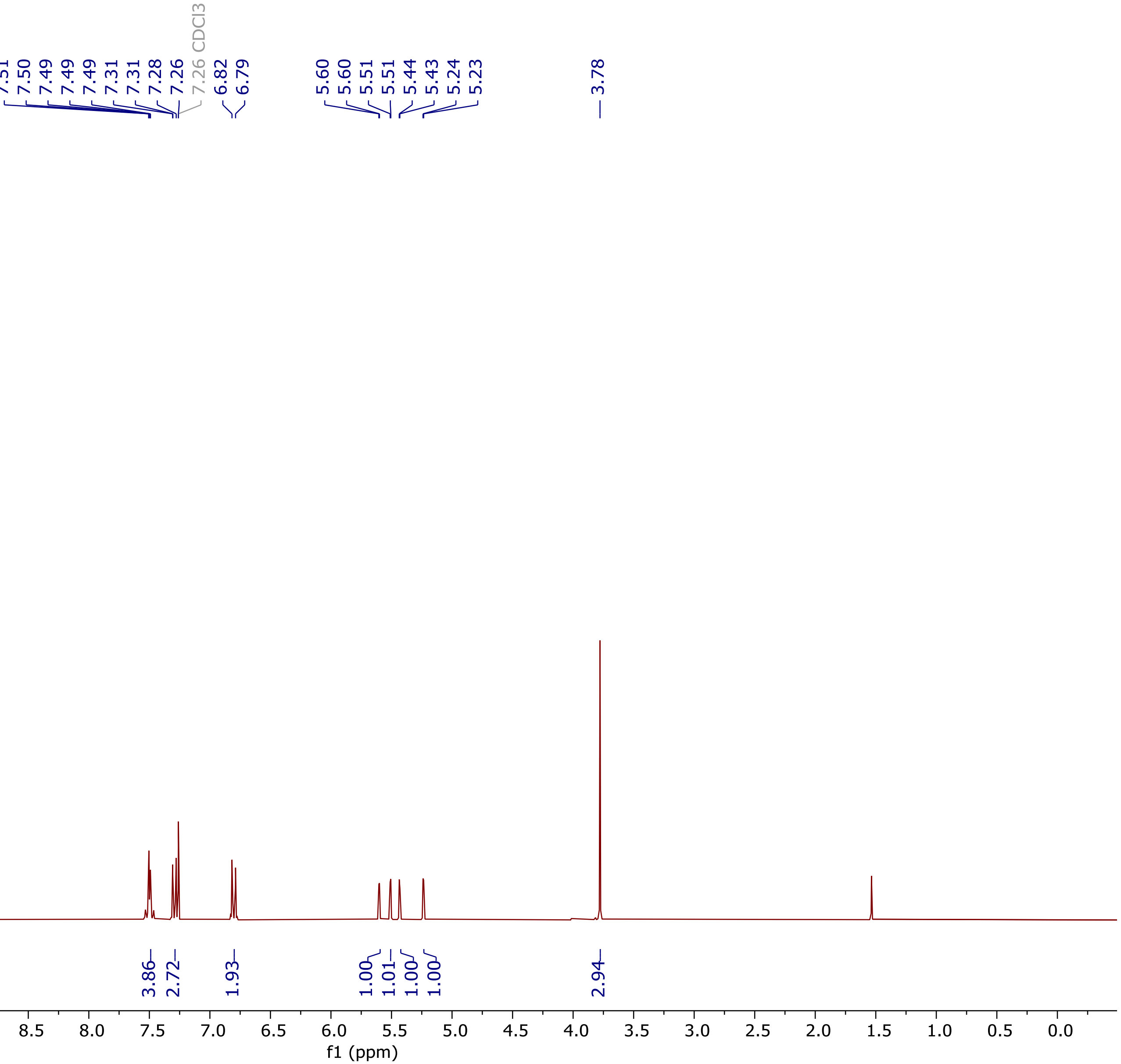

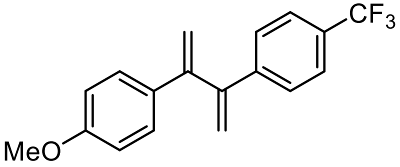

1l

^{19}**F{^{1}H} NMR** (282 MHz, $CDCl_3$)

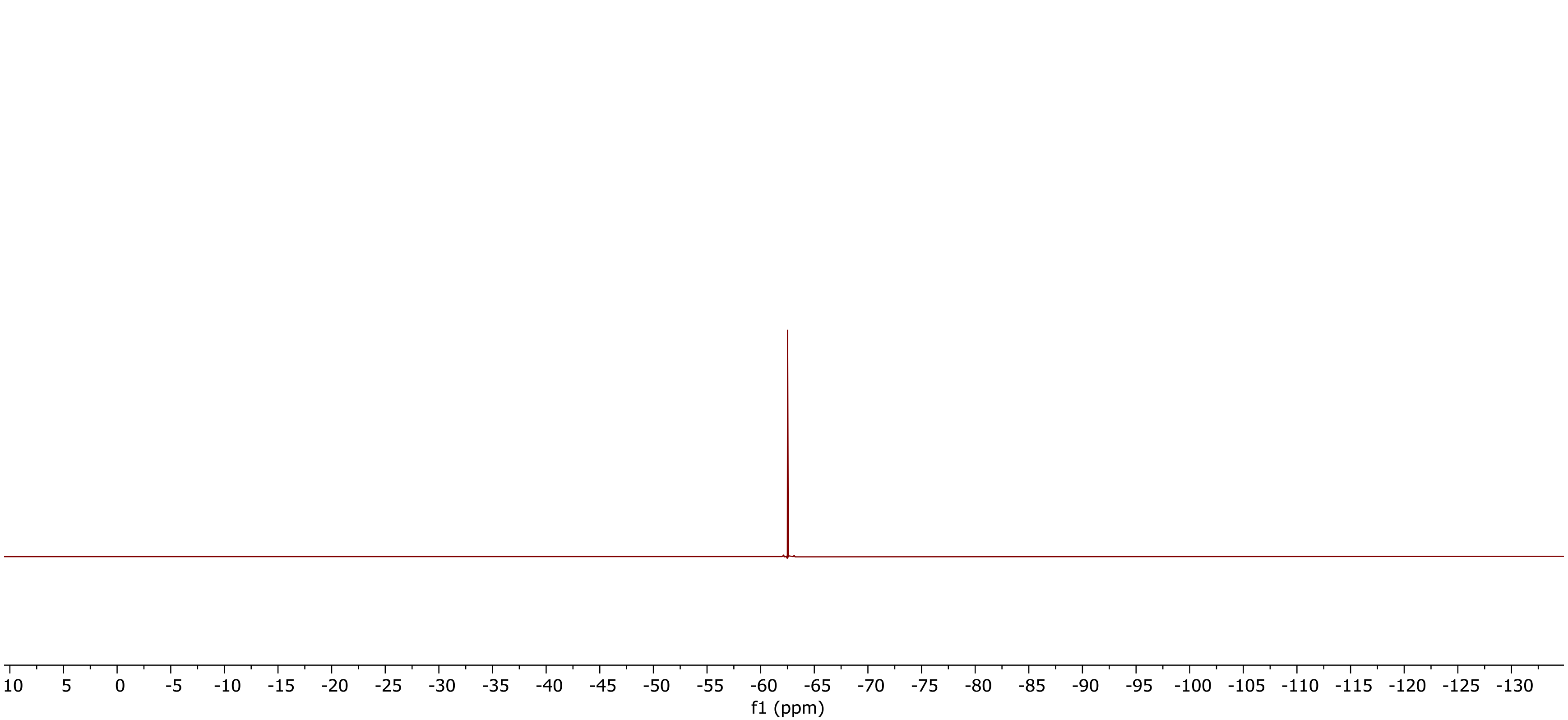

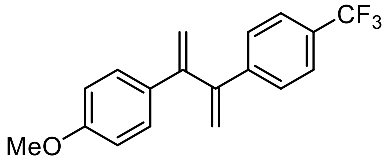

1l

$^{13}C\{^{1}H\}$ NMR (100 MHz, $CDCl_3$)

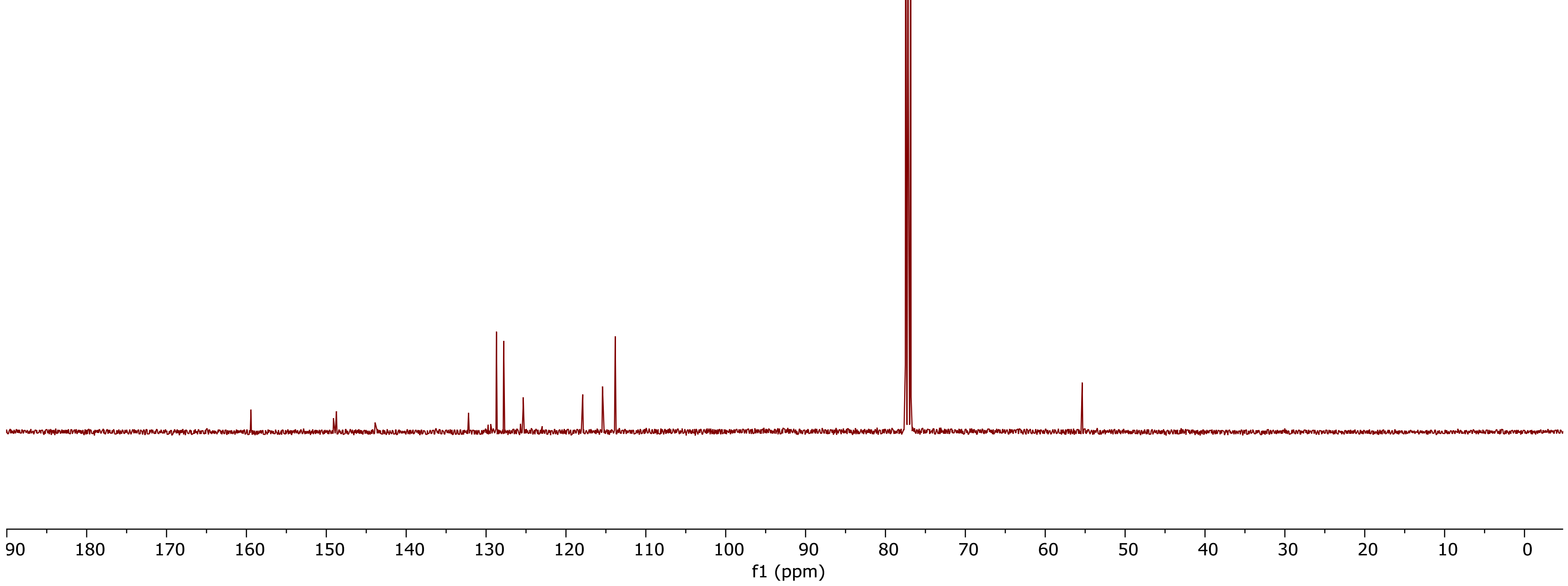

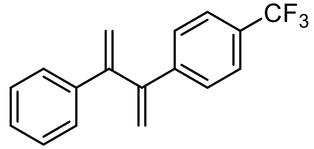

1m

^{1}H NMR (300 MHz, $CDCl_3$)

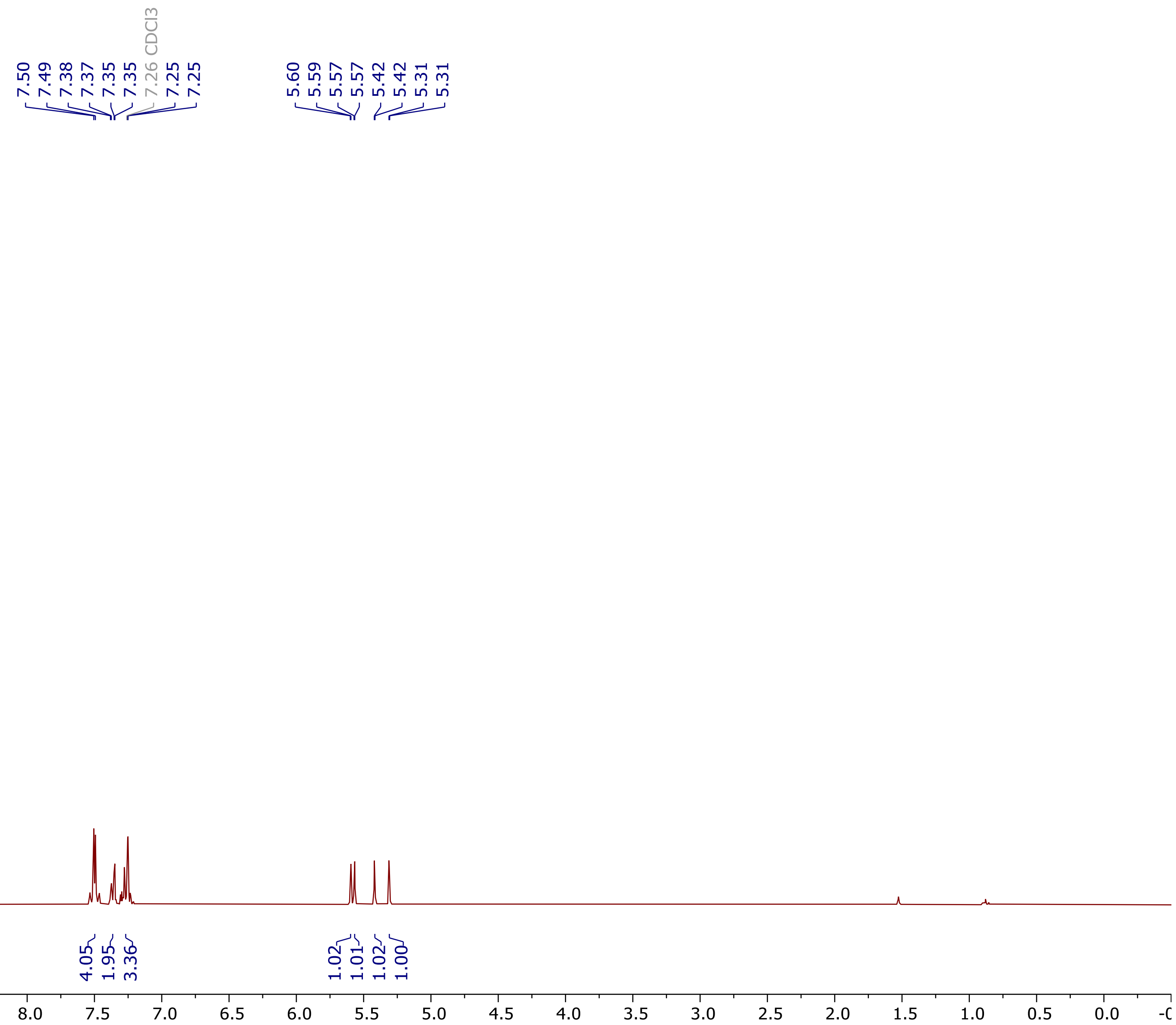

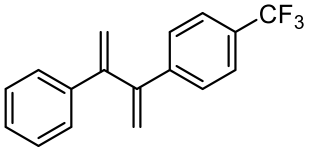

1m

$^{19}F\{^{1}H\}$ NMR (282 MHz, $CDCl_3$)

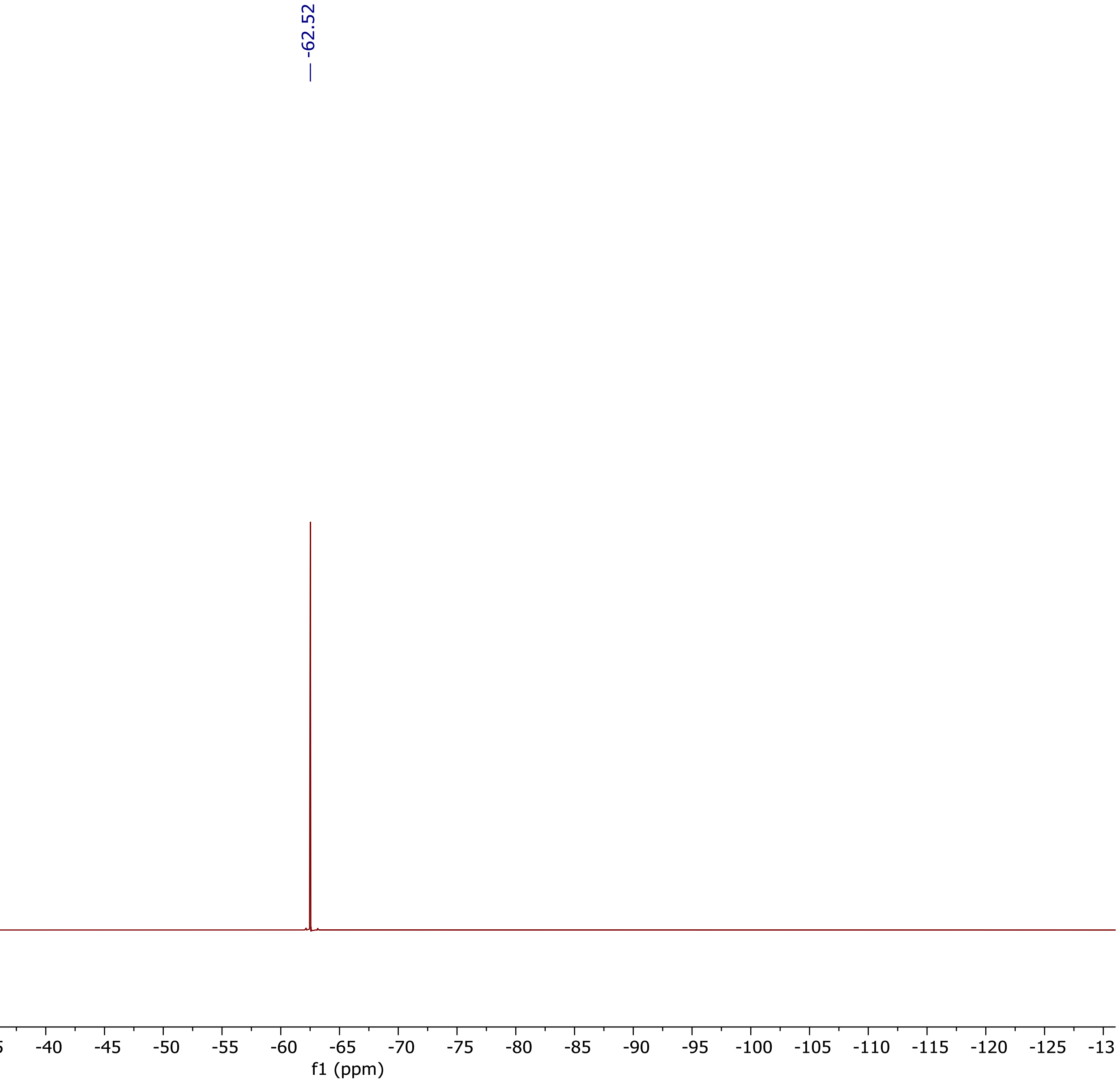

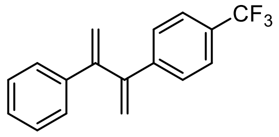

1m

^{13}C{^{1}H} **NMR** (75 MHz, $CDCl_3$)

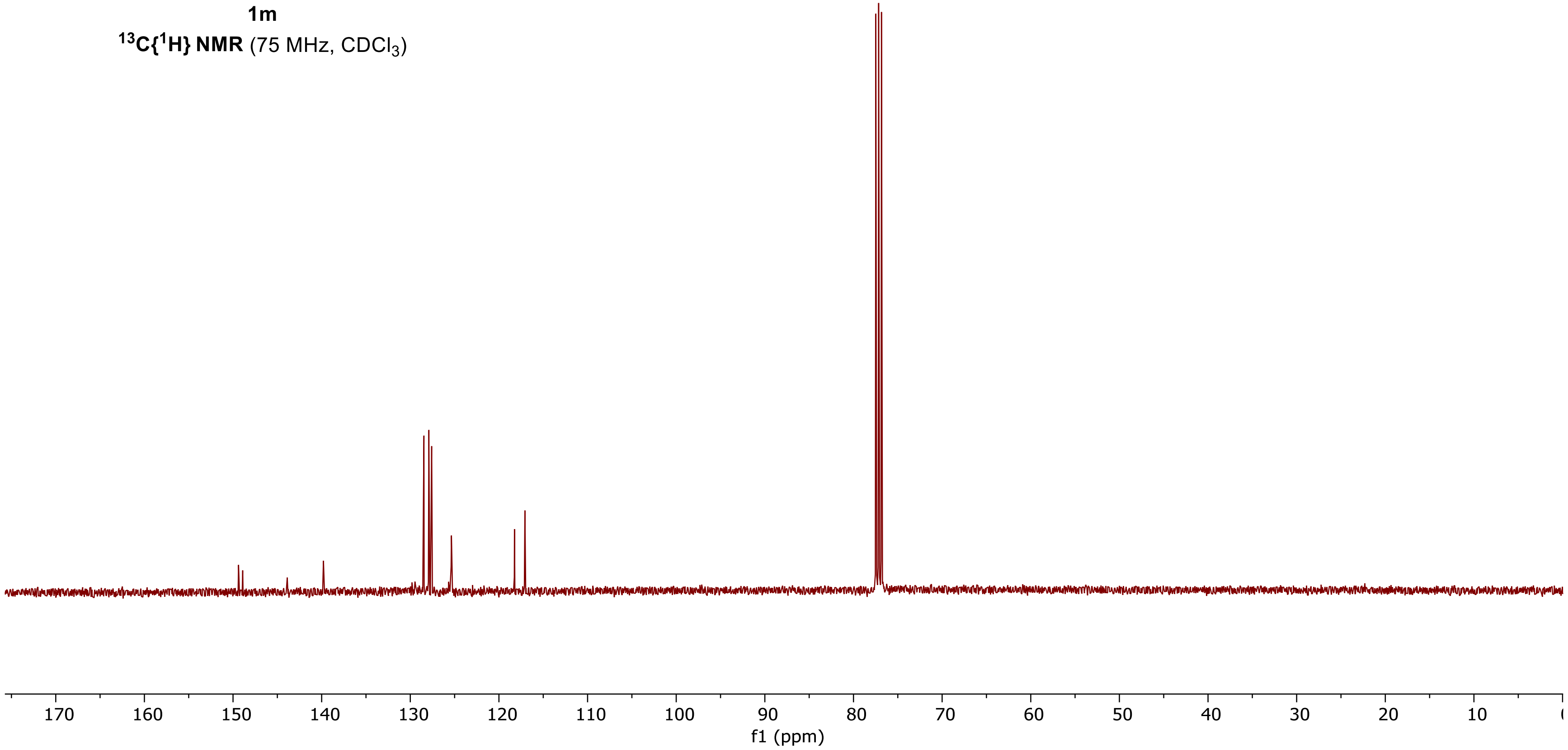

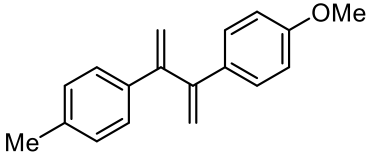

1n

^{1}H NMR (300 MHz, $CDCl_3$)

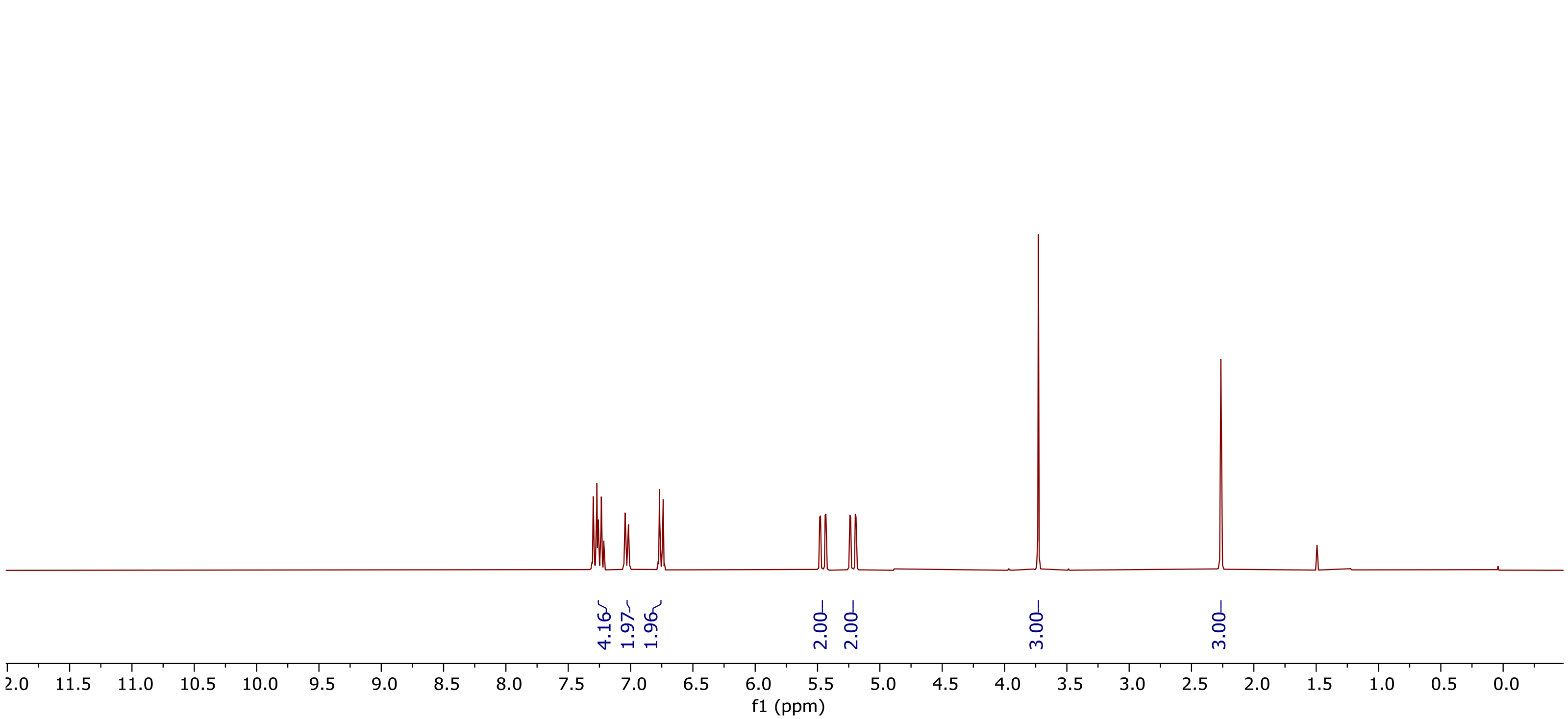

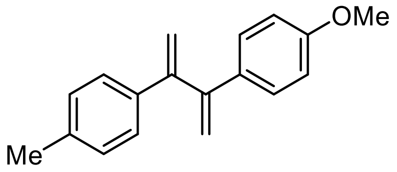

1n

^{13}C{^{1}H} NMR (75 MHz, $CDCl_3$)

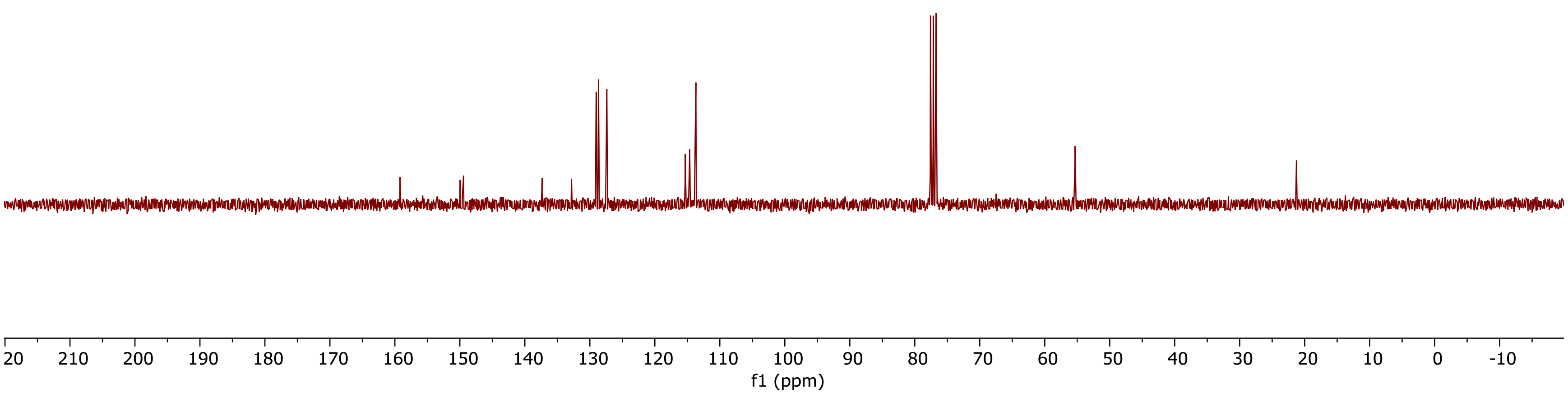

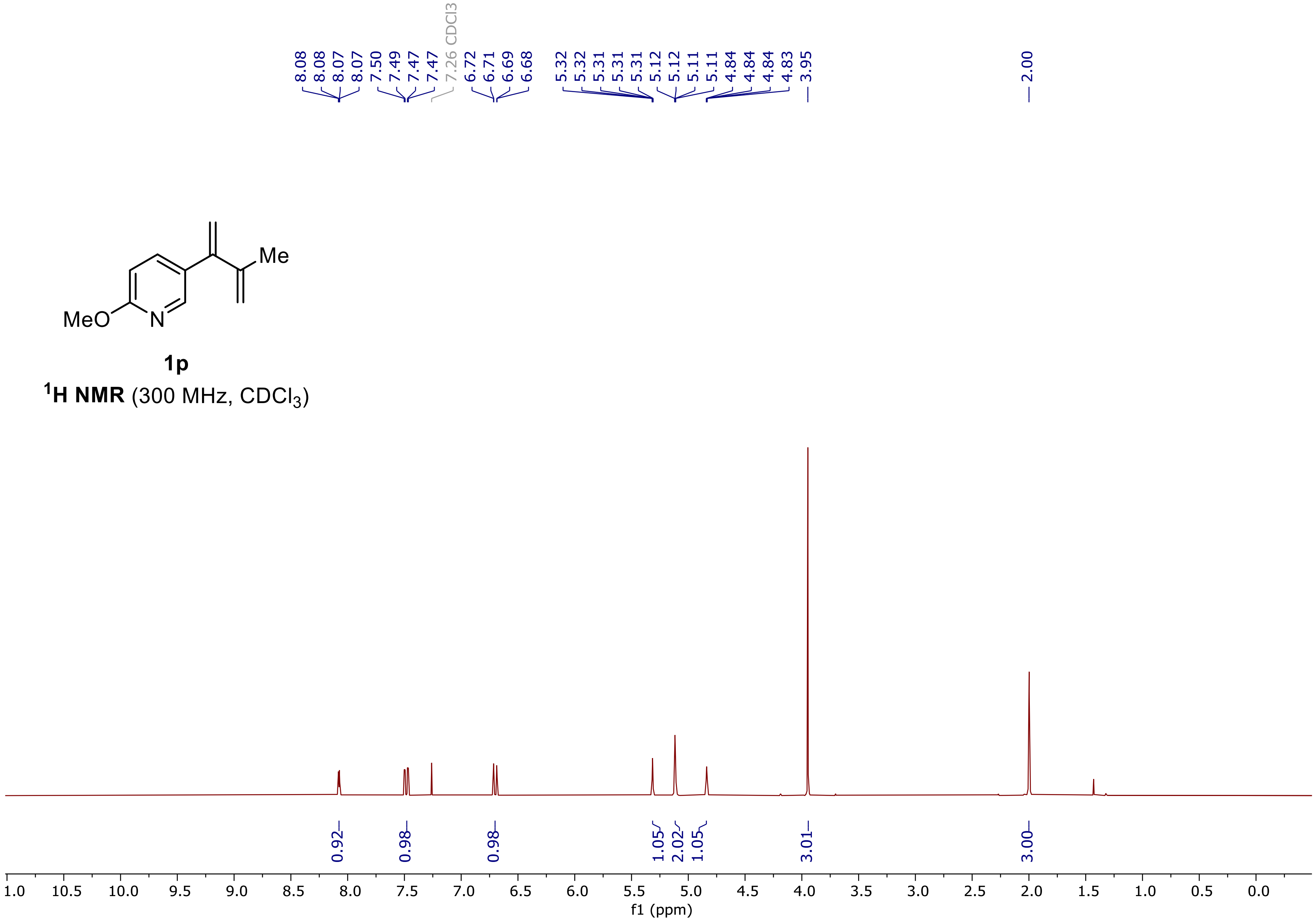

1p

^{1}H NMR (300 MHz, $CDCl_3$)

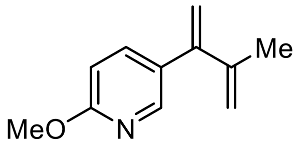

1p

^{13}C{^{1}H} NMR (75 MHz, $CDCl_3$)

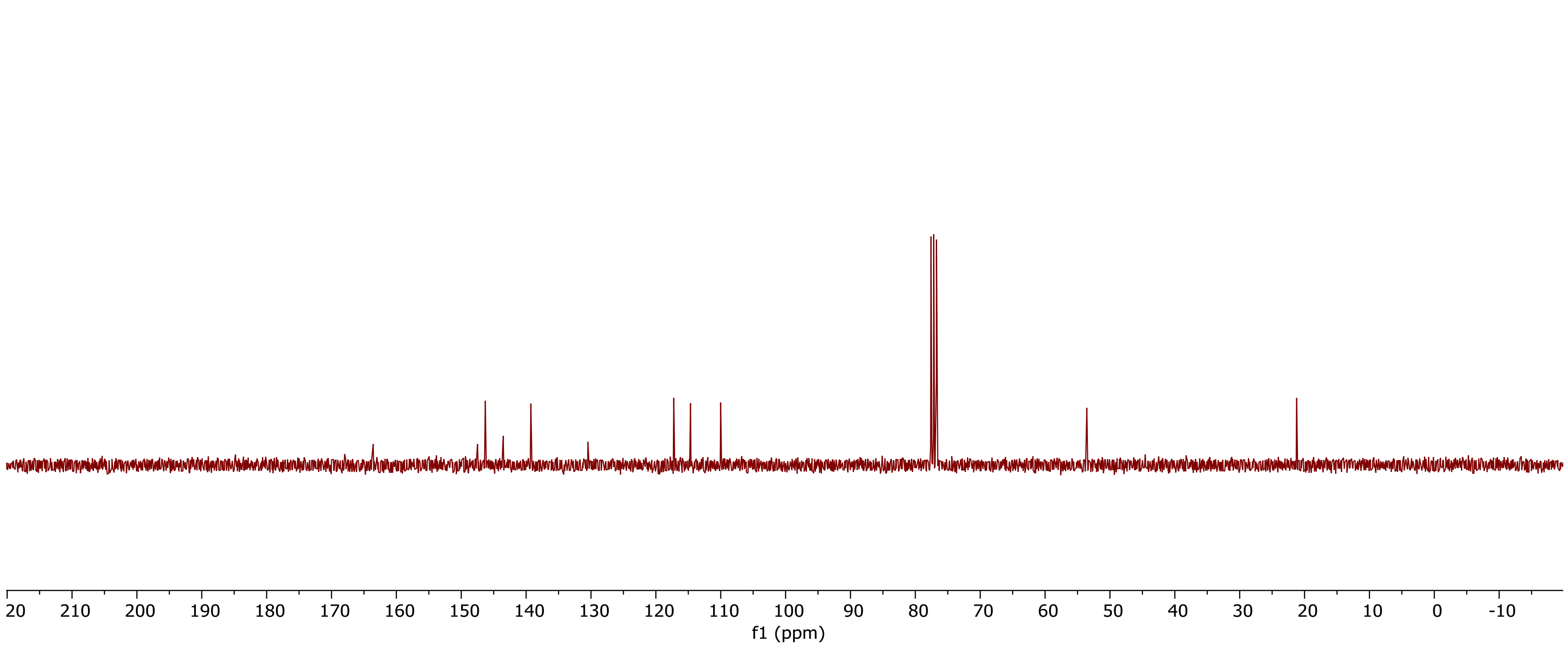

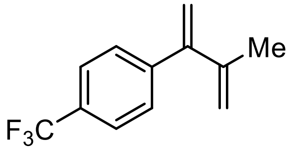

1q

^{1}H NMR (400 MHz, $CDCl_3$)

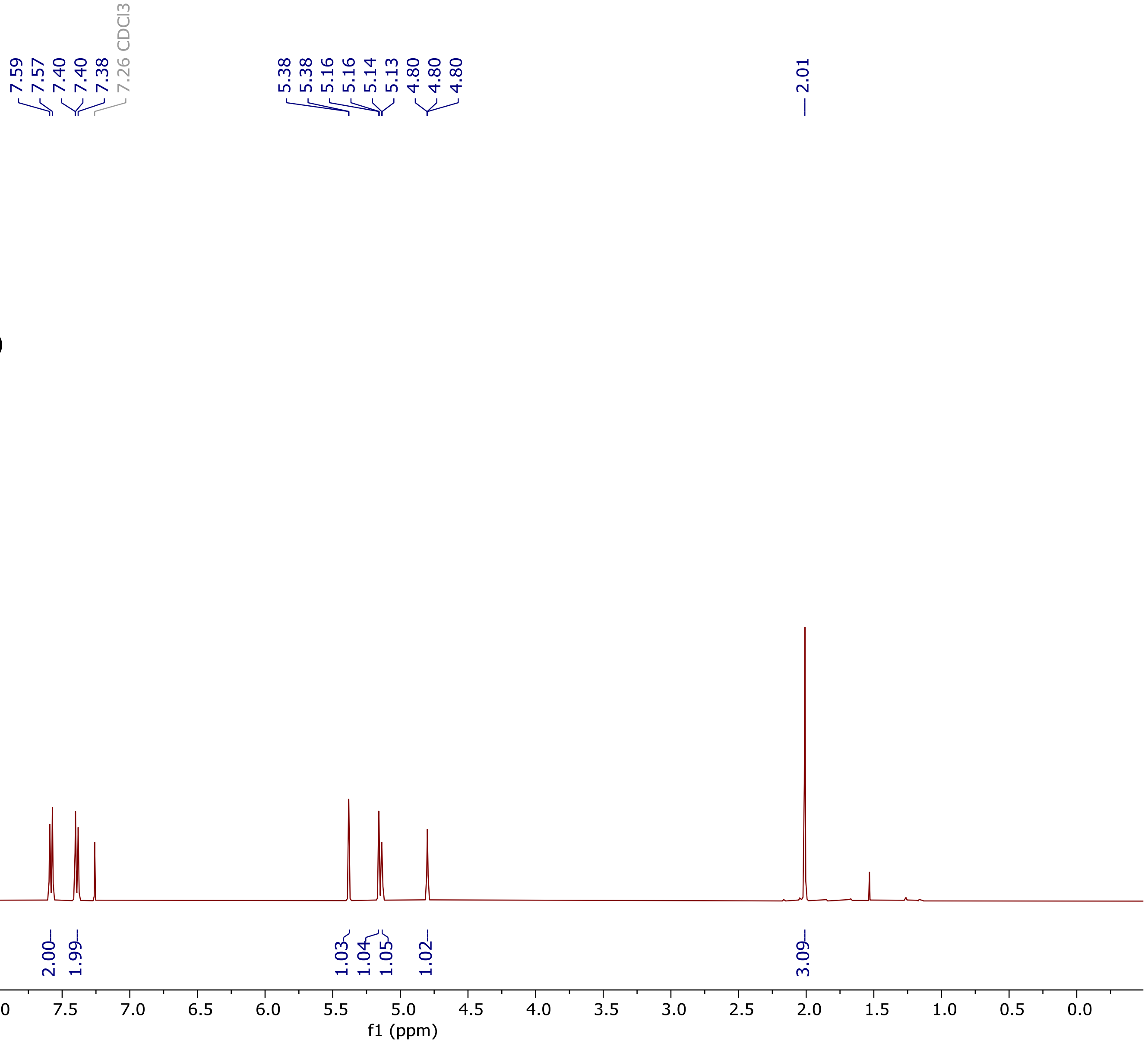

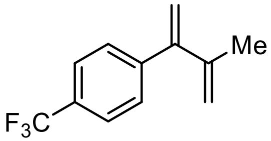

1q

$^{19}F\{^{1}H\}$ NMR (282 MHz, $CDCl_3$)

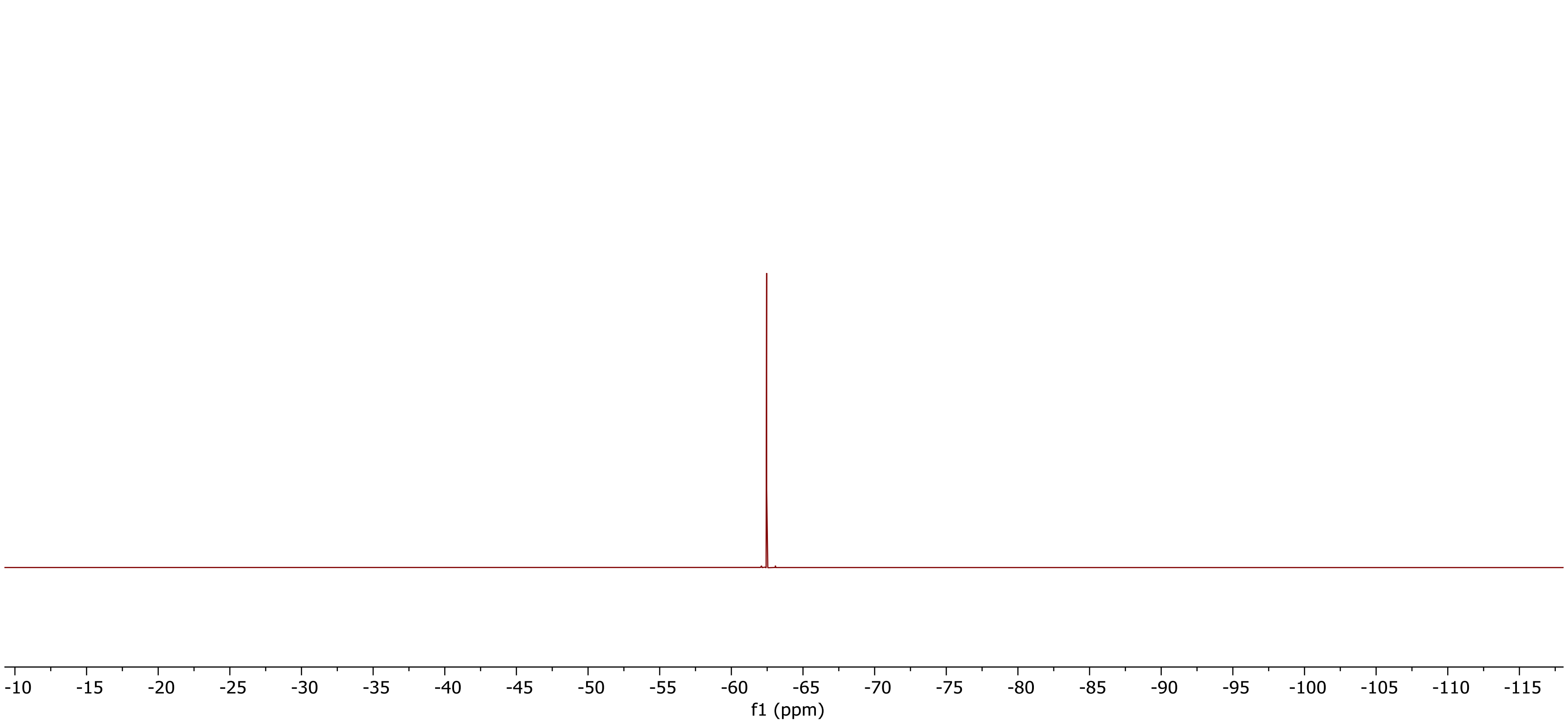

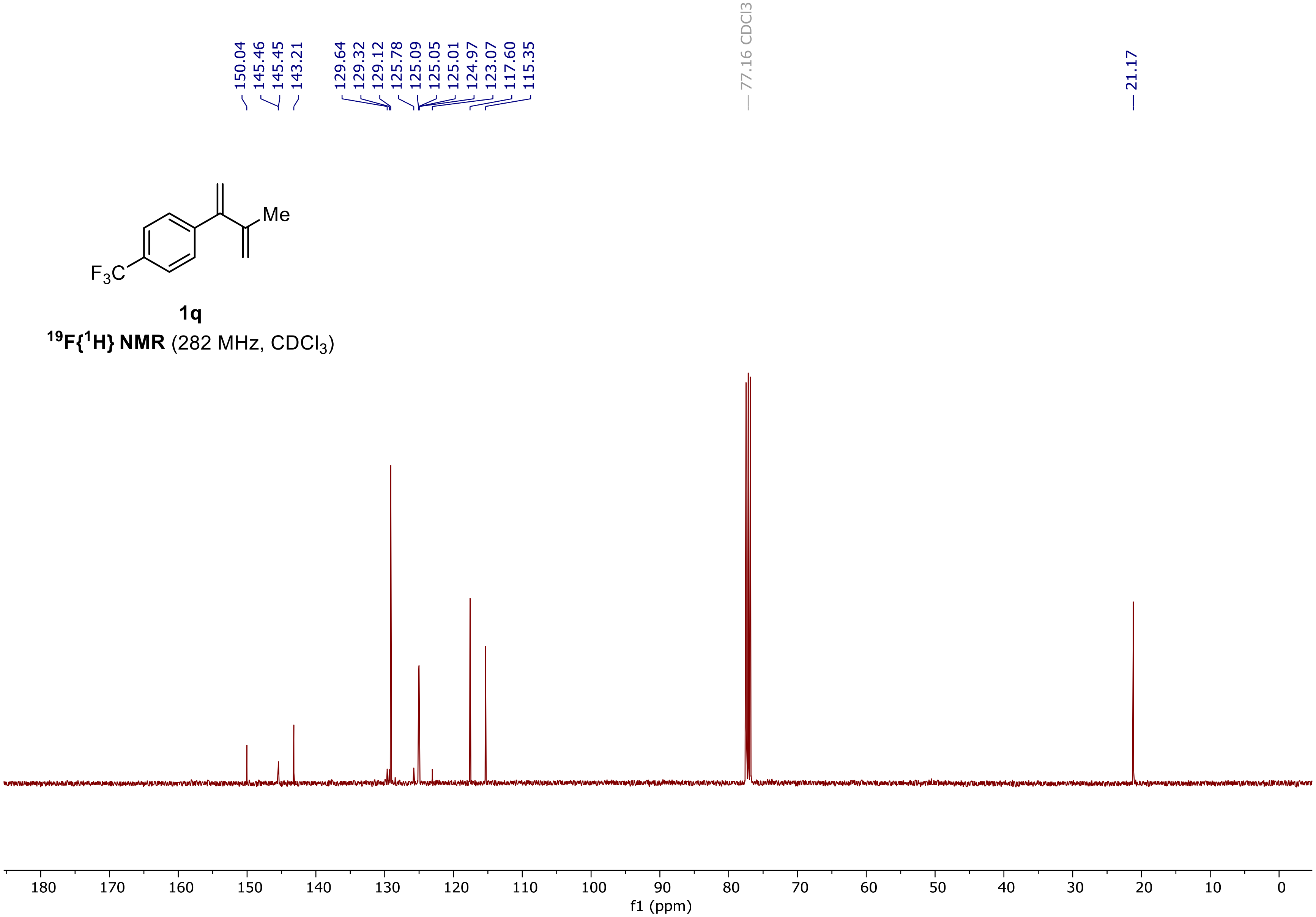

1q

^{19}F{^{1}H} NMR (282 MHz, $CDCl_3$)

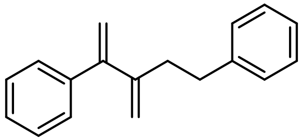

1r

[1]**H NMR** (400 MHz, $CDCl_3$)

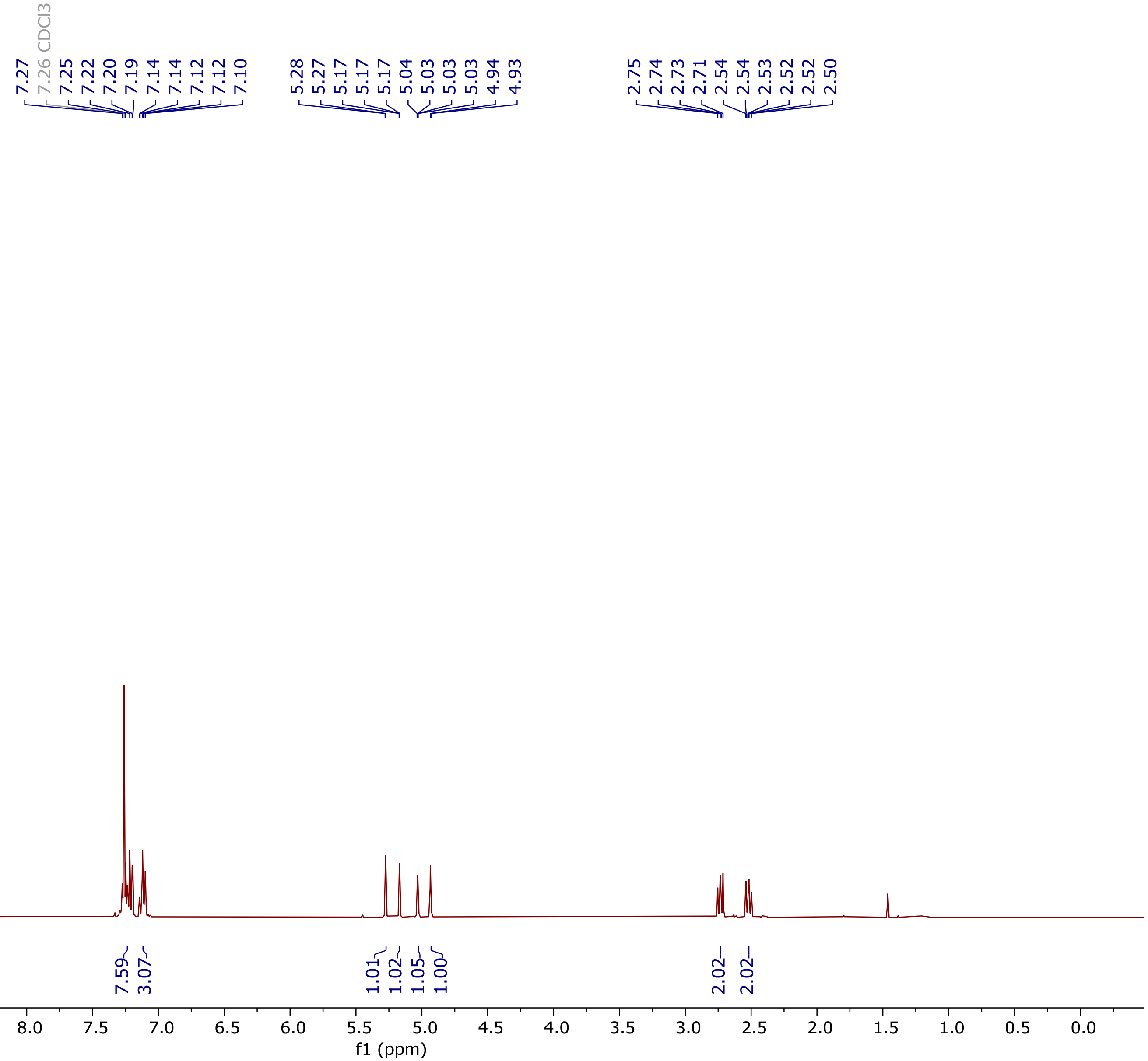

1r

$^{13}C\{^{1}H\}$ NMR (100 MHz, $CDCl_3$)

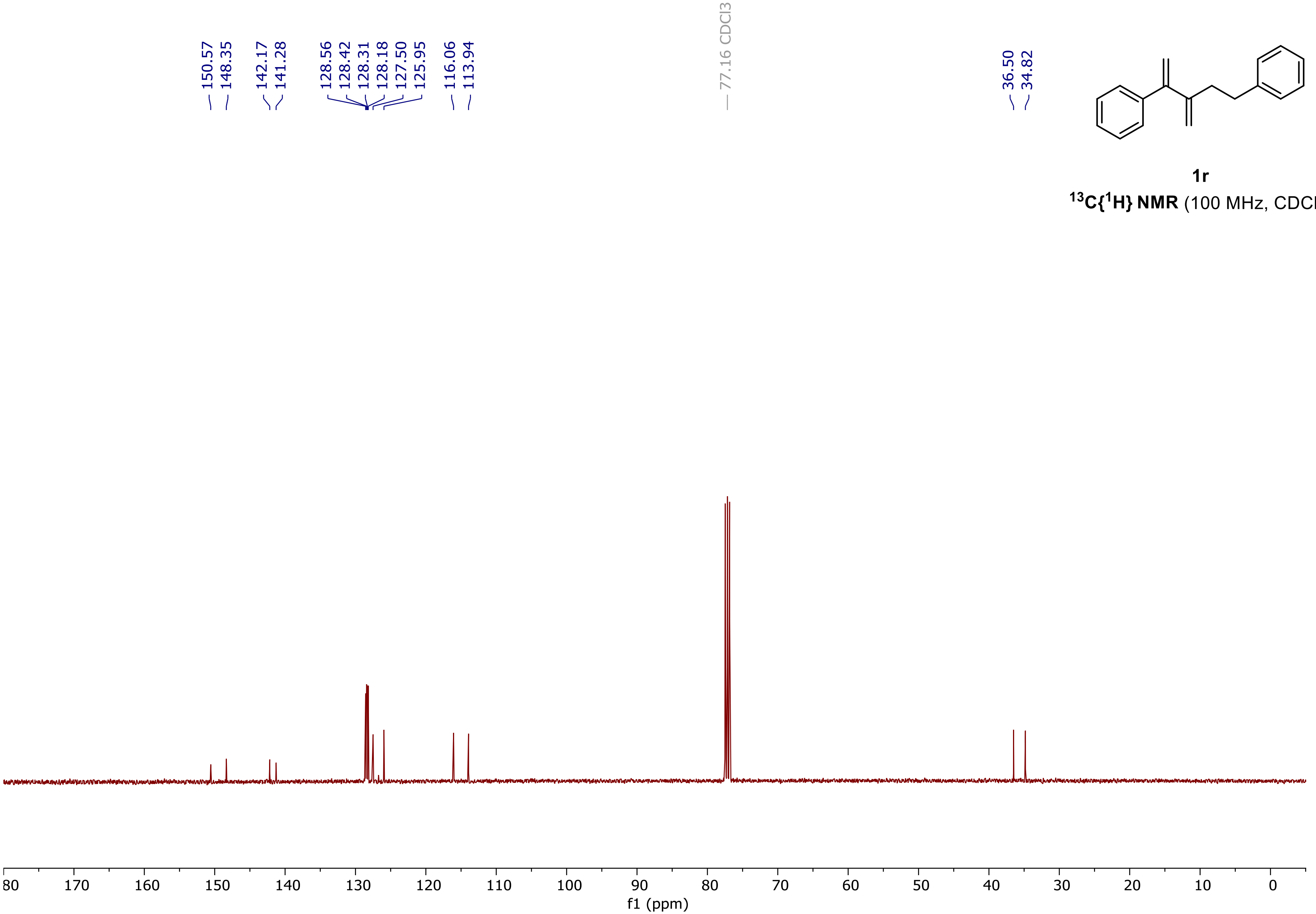

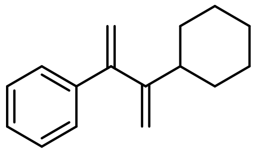

1s

^{1}H NMR (300 MHz, $CDCl_3$)

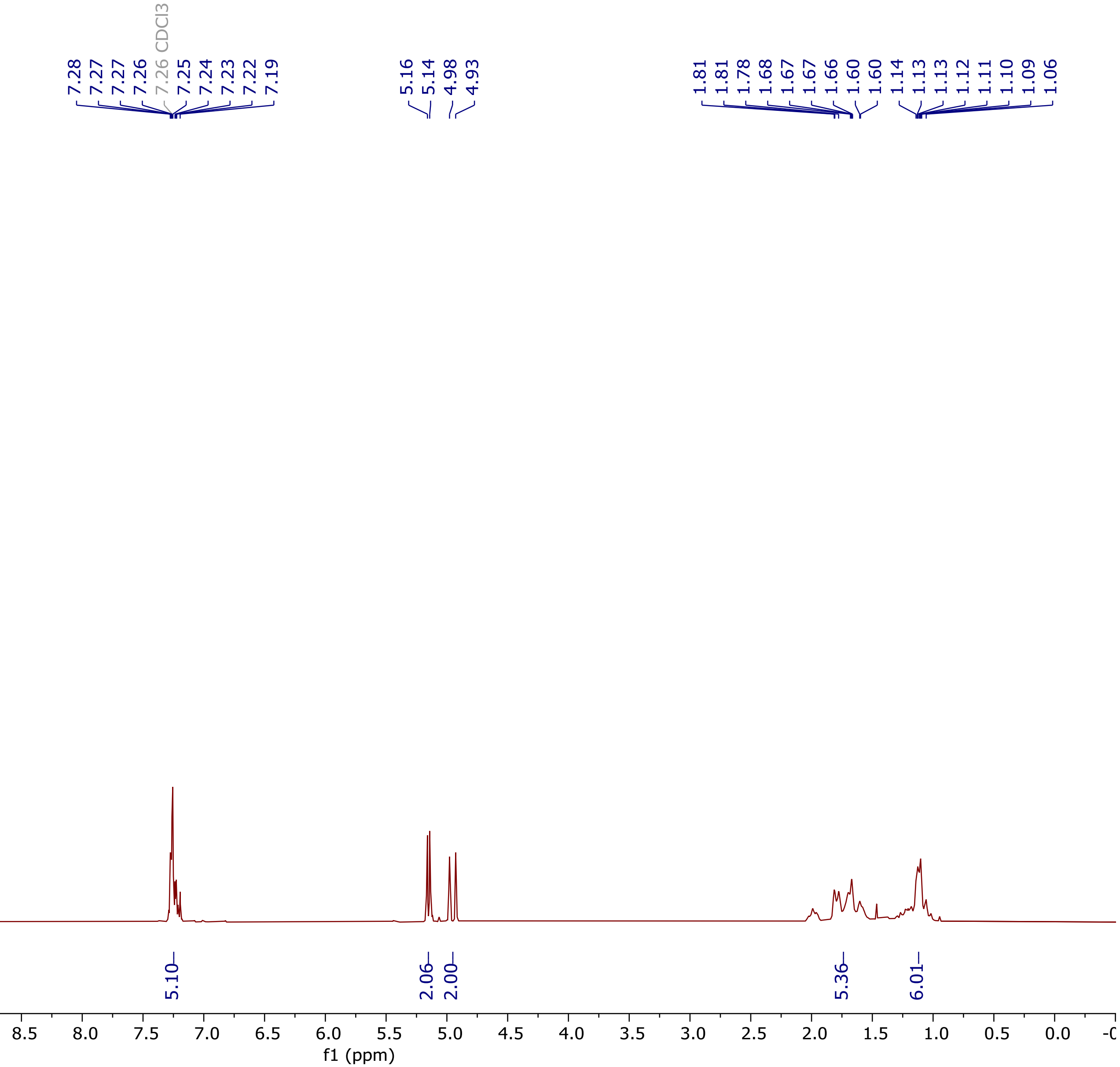

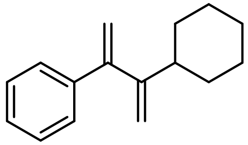

1s

^{13}C{^{1}H} NMR (75 MHz, $CDCl_3$)

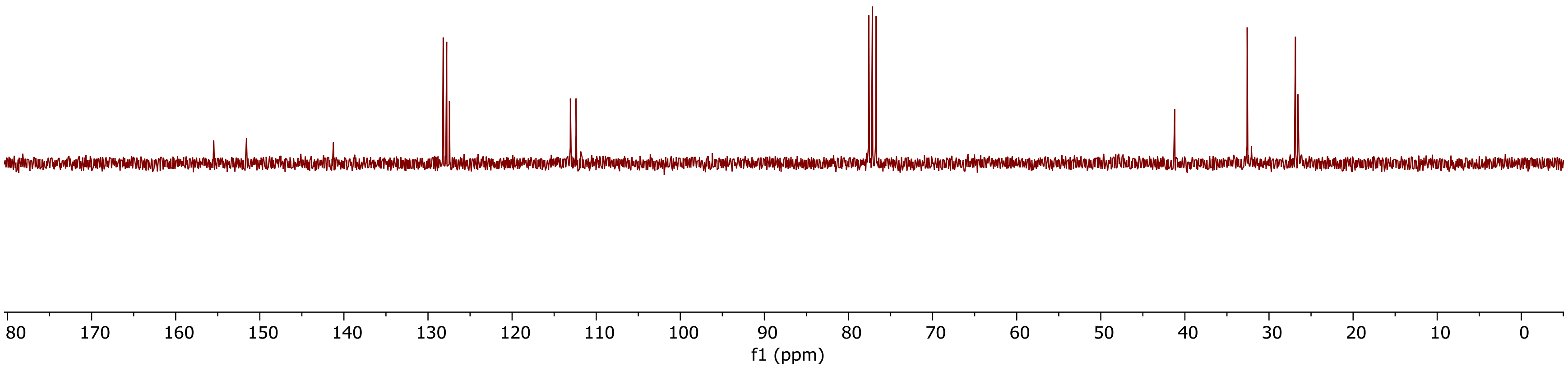

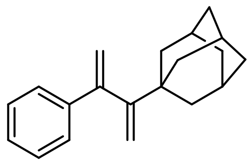

1t

^{1}H NMR (400 MHz, $CDCl_3$)

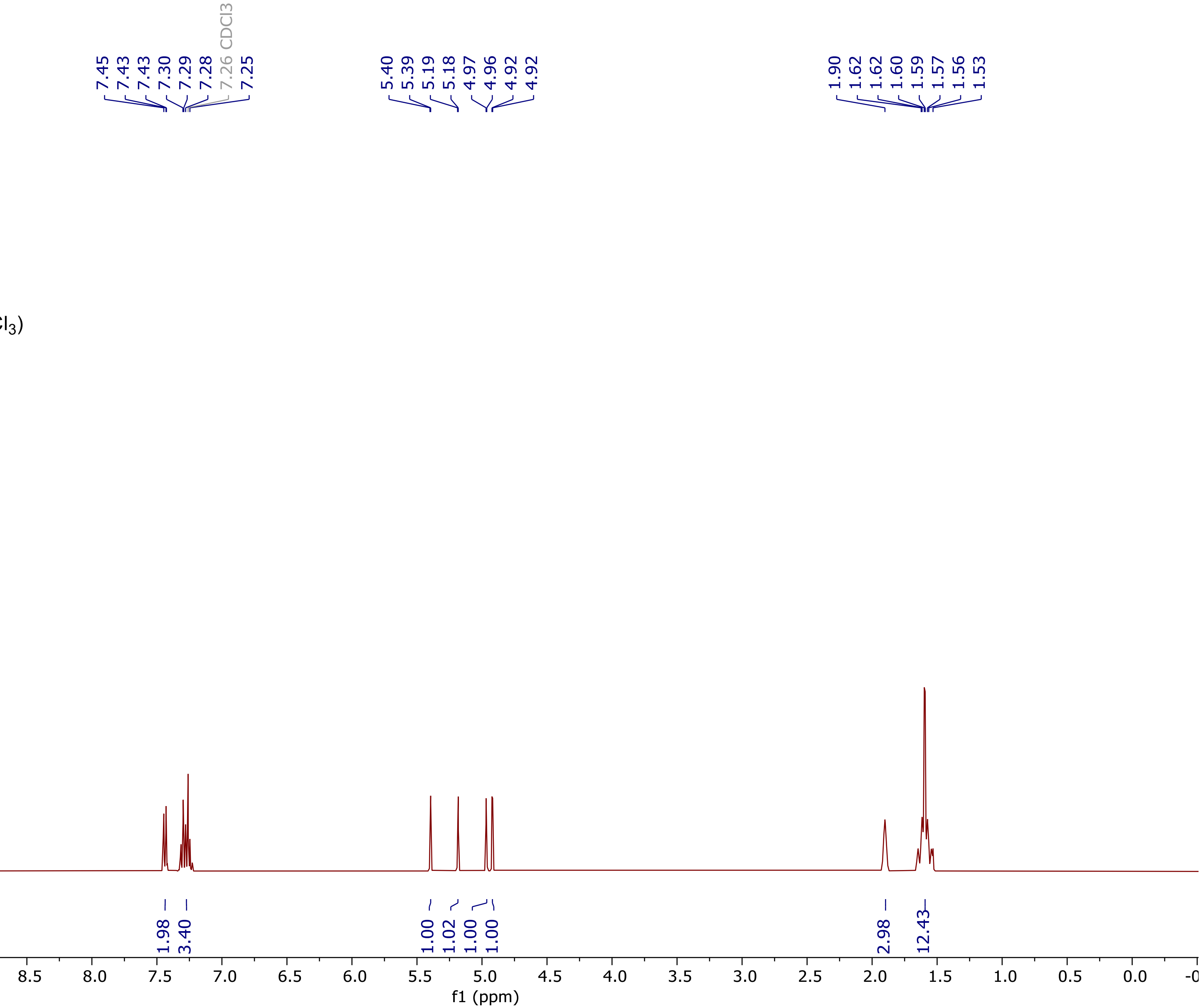

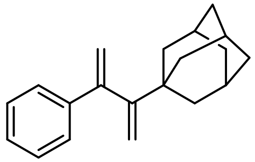

1t

^{13}**C{^{1}H} NMR** (101 MHz, $CDCl_3$)

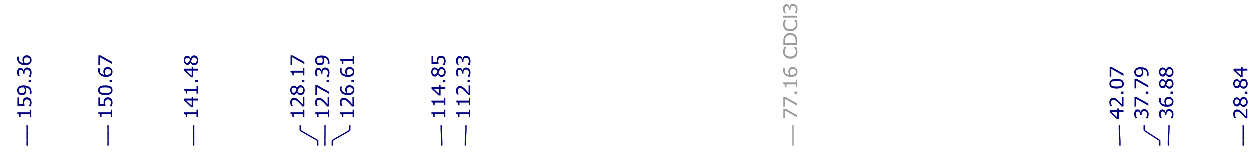

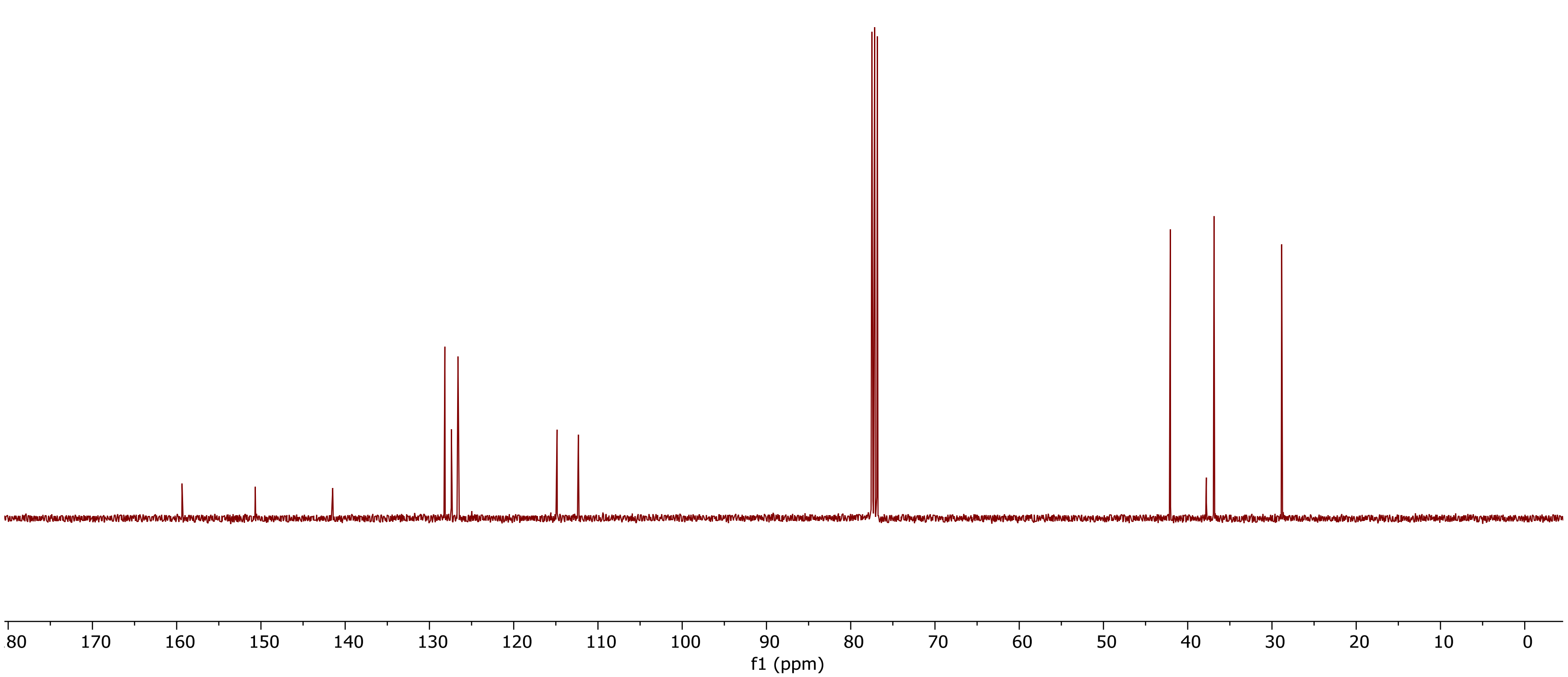

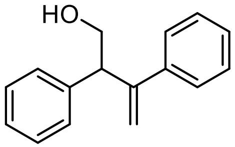

2'a

^{1}H NMR (400 MHz, $CDCl_3$)

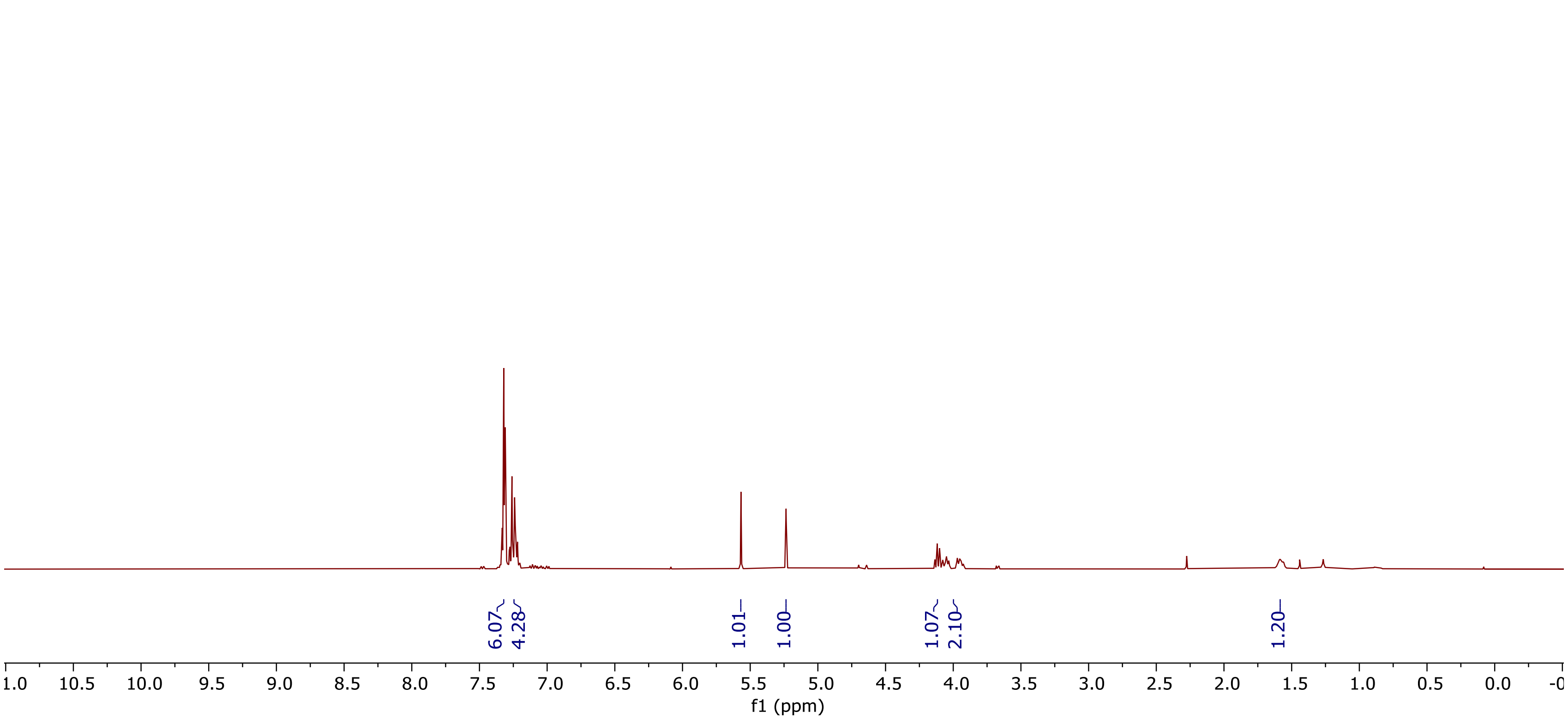

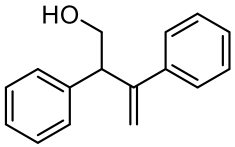

2'a

^{13}**C{^{1}H} NMR** (101 MHz, $CDCl_3$)

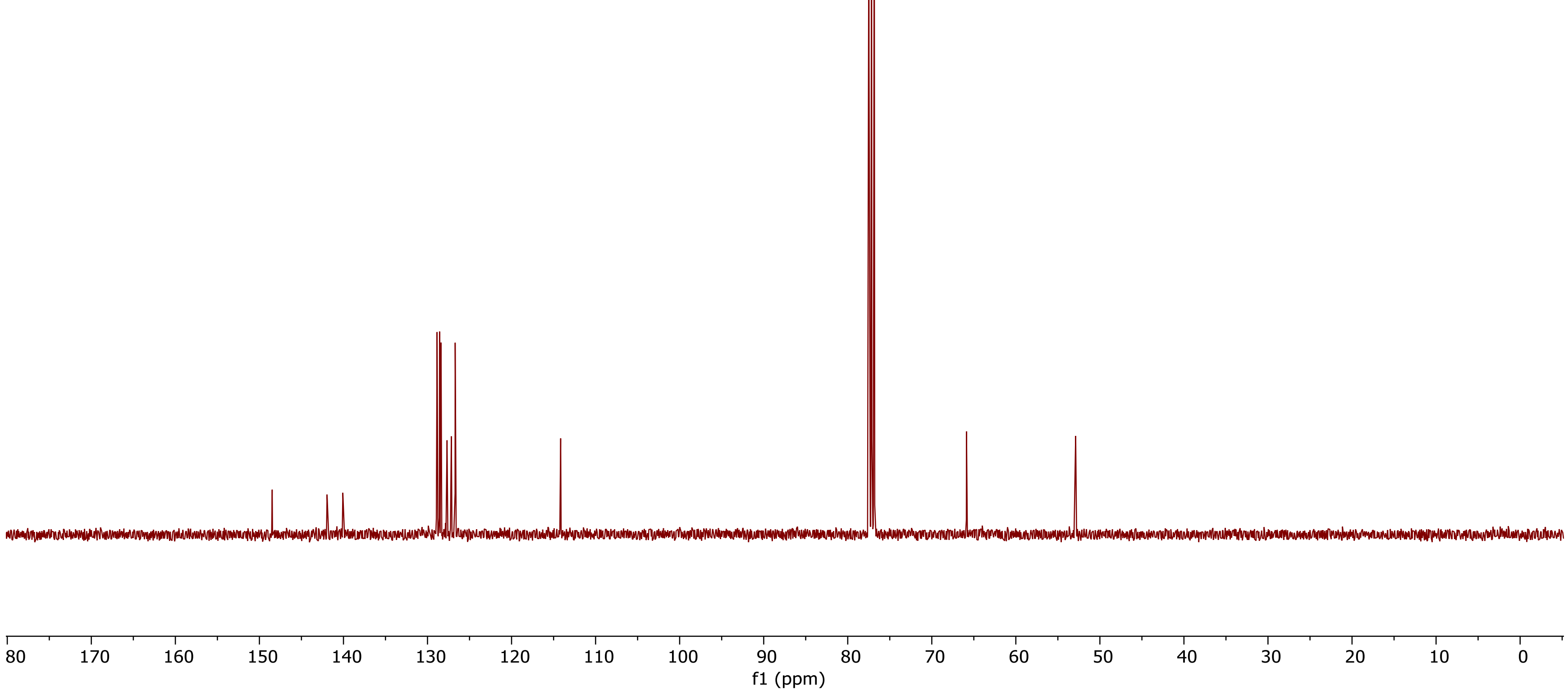

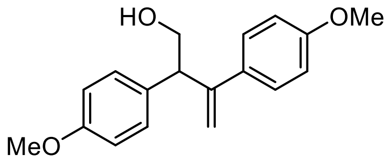

2'b

^{1}H NMR (300 MHz, $CDCl_3$)

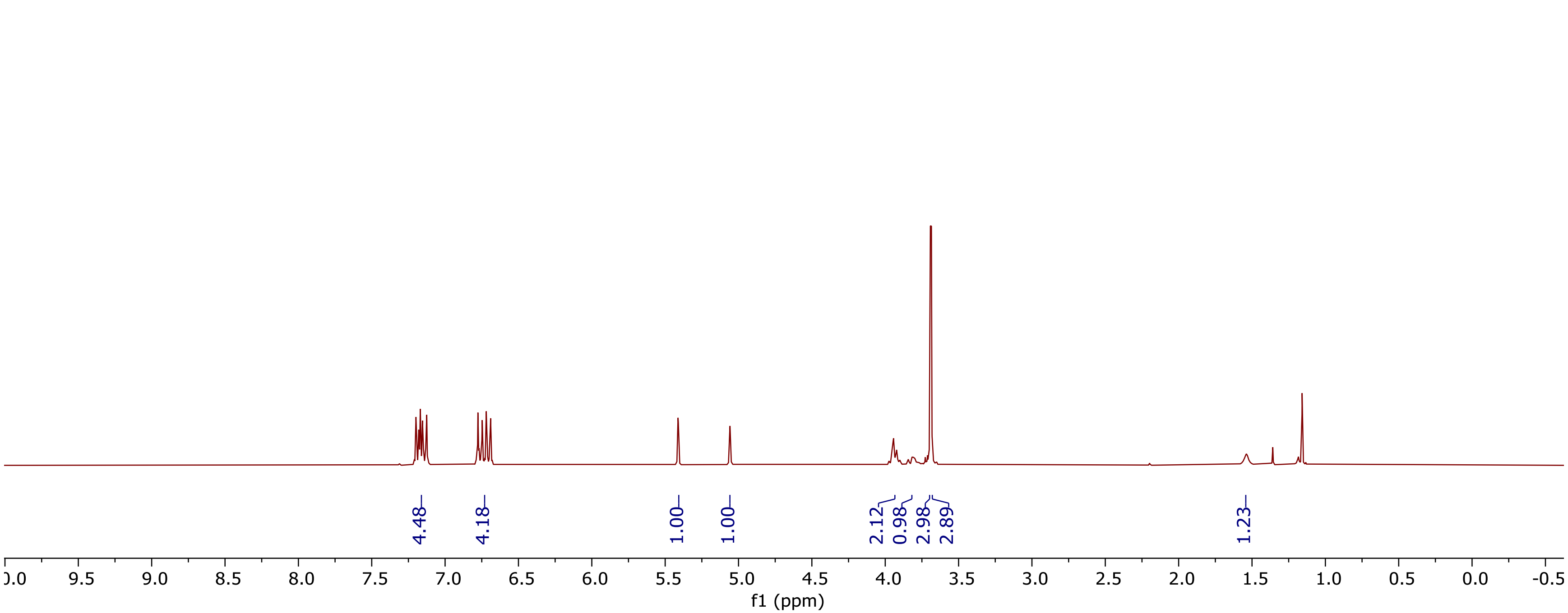

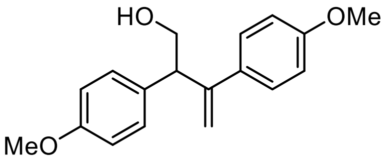

2'b

^{13}C{^{1}H} NMR (75 MHz, $CDCl_3$)

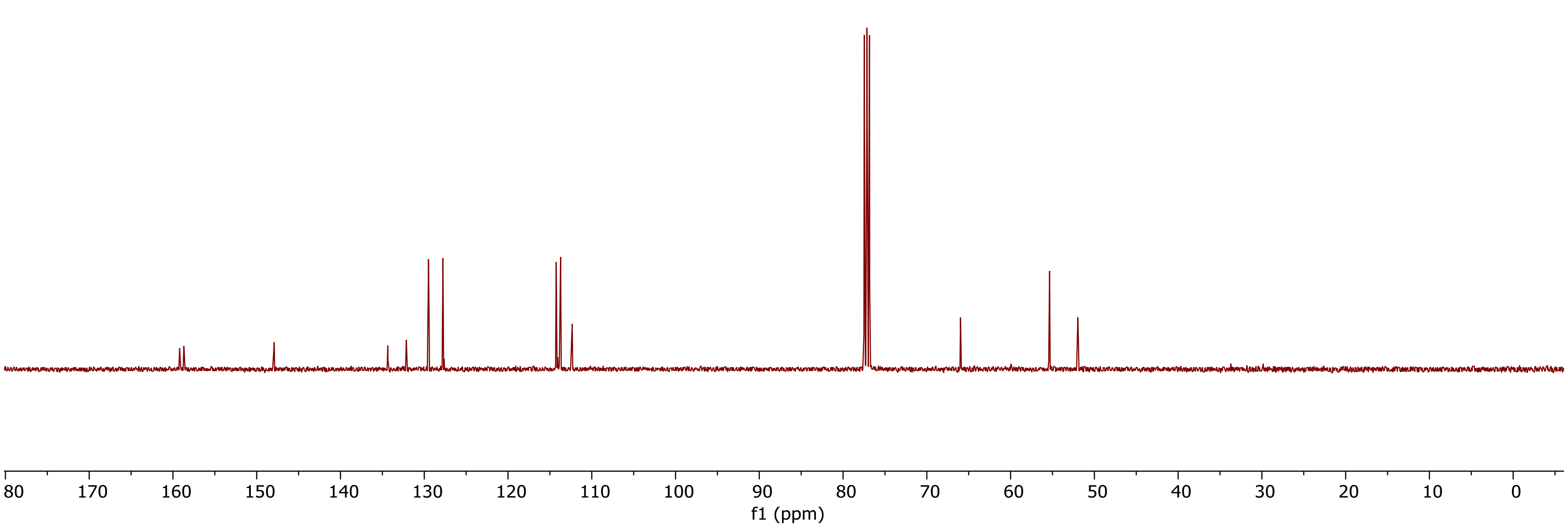

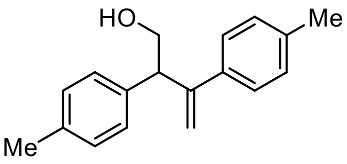

2'c

^{1}H NMR (400 MHz, $CDCl_3$)

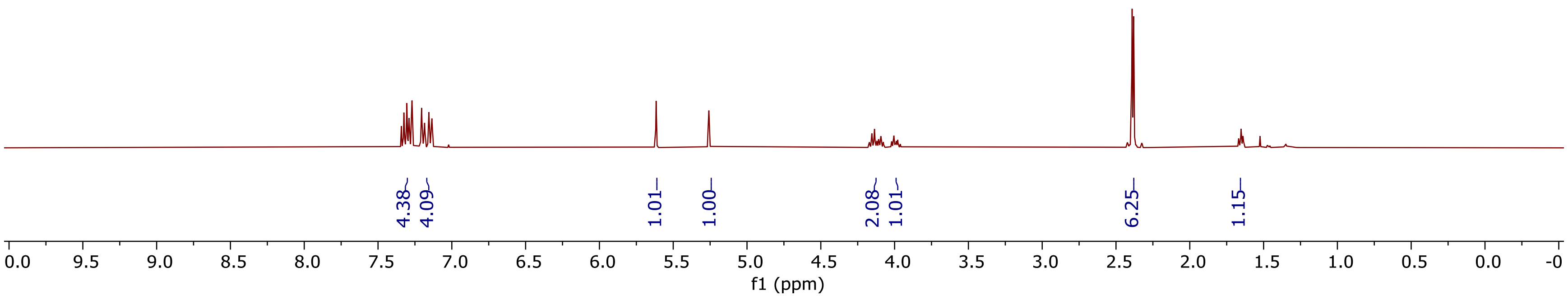

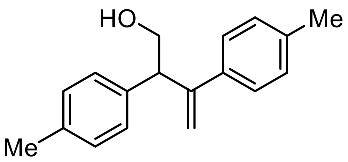

2'c

^{13}C{^{1}H} NMR (100 MHz, $CDCl_3$)

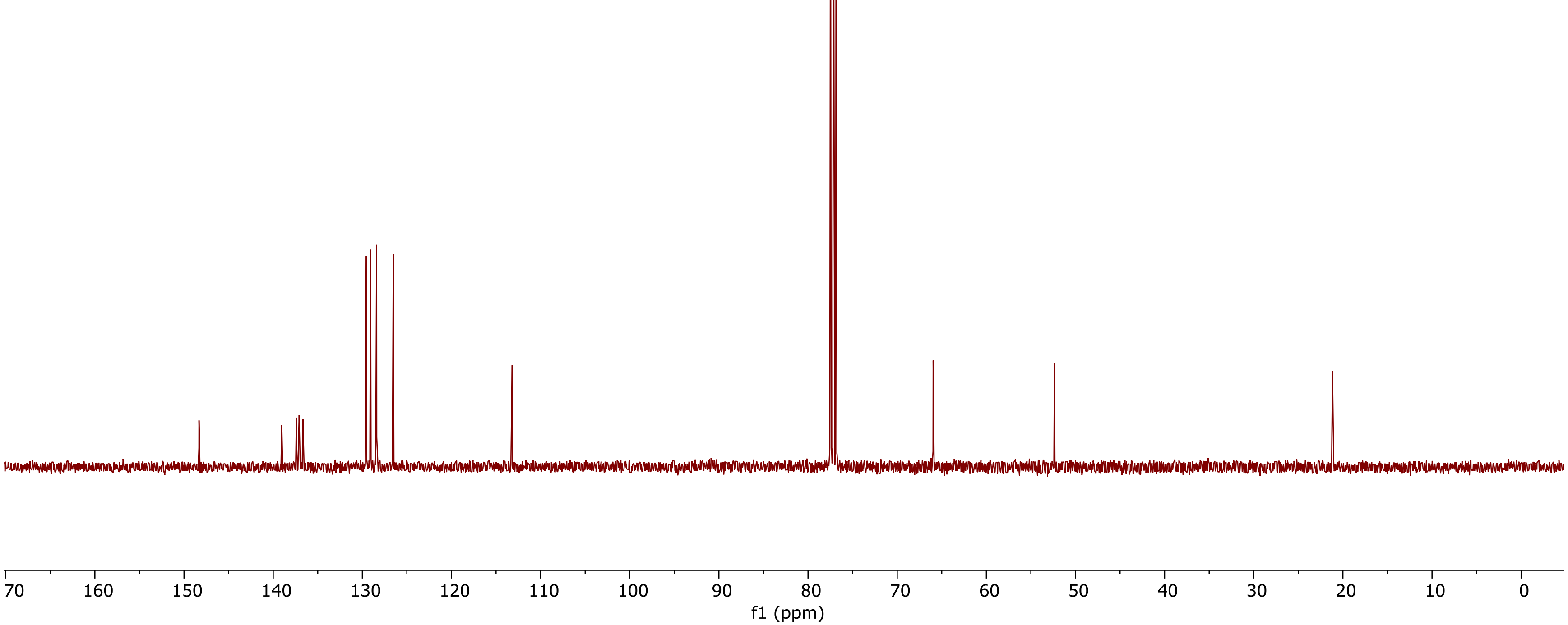

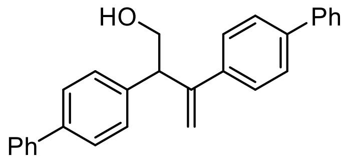

2'd

^{1}H NMR (400 MHz, $CDCl_3$)

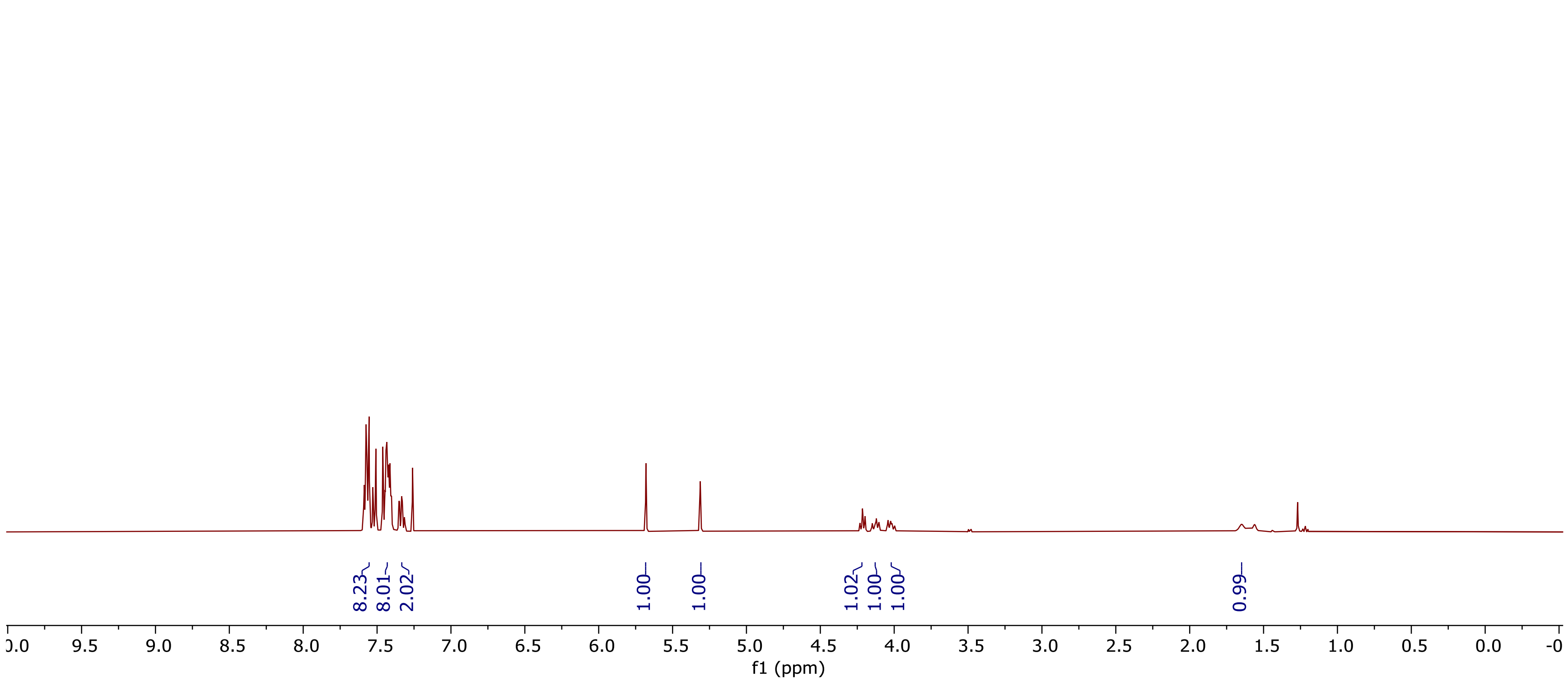

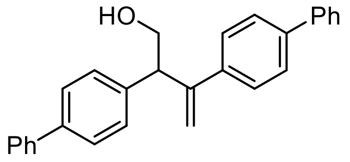

2'd

^{13}C{^{1}H} NMR (101 MHz, $CDCl_3$)

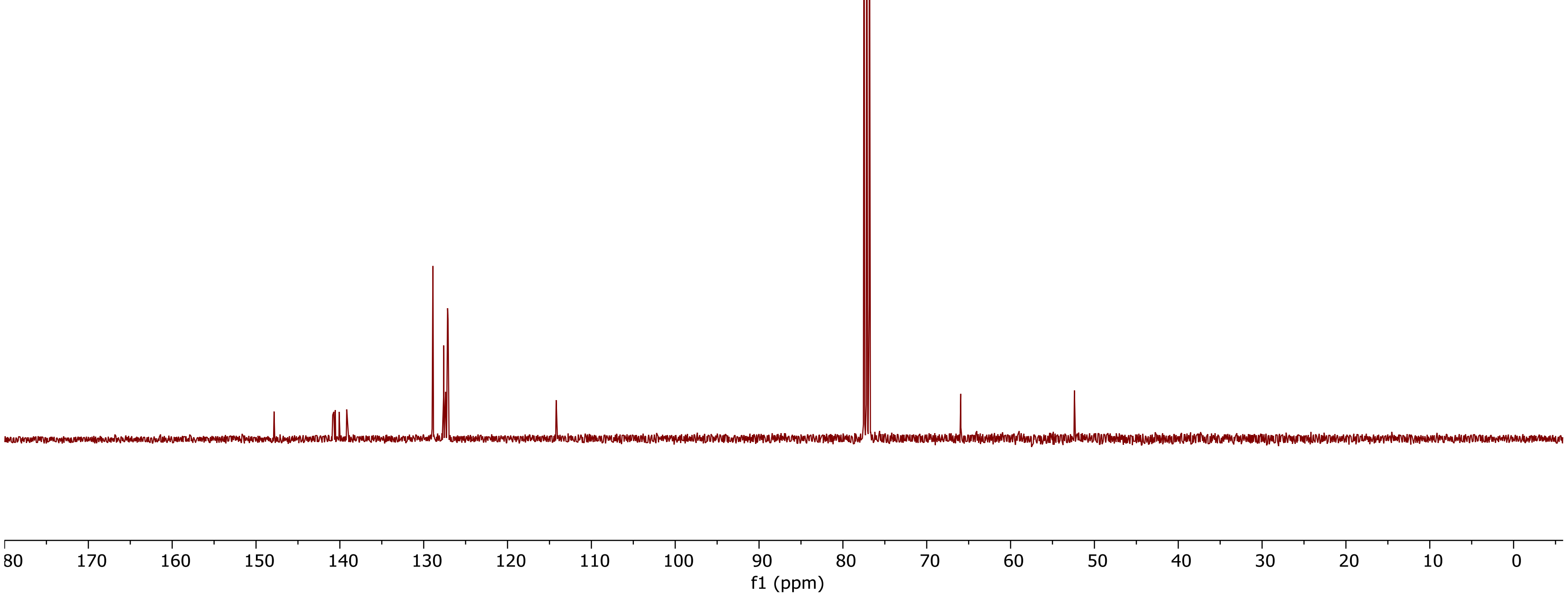

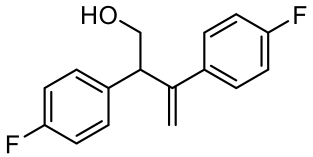

2'e

^{1}H NMR (400 MHz, $CDCl_3$)

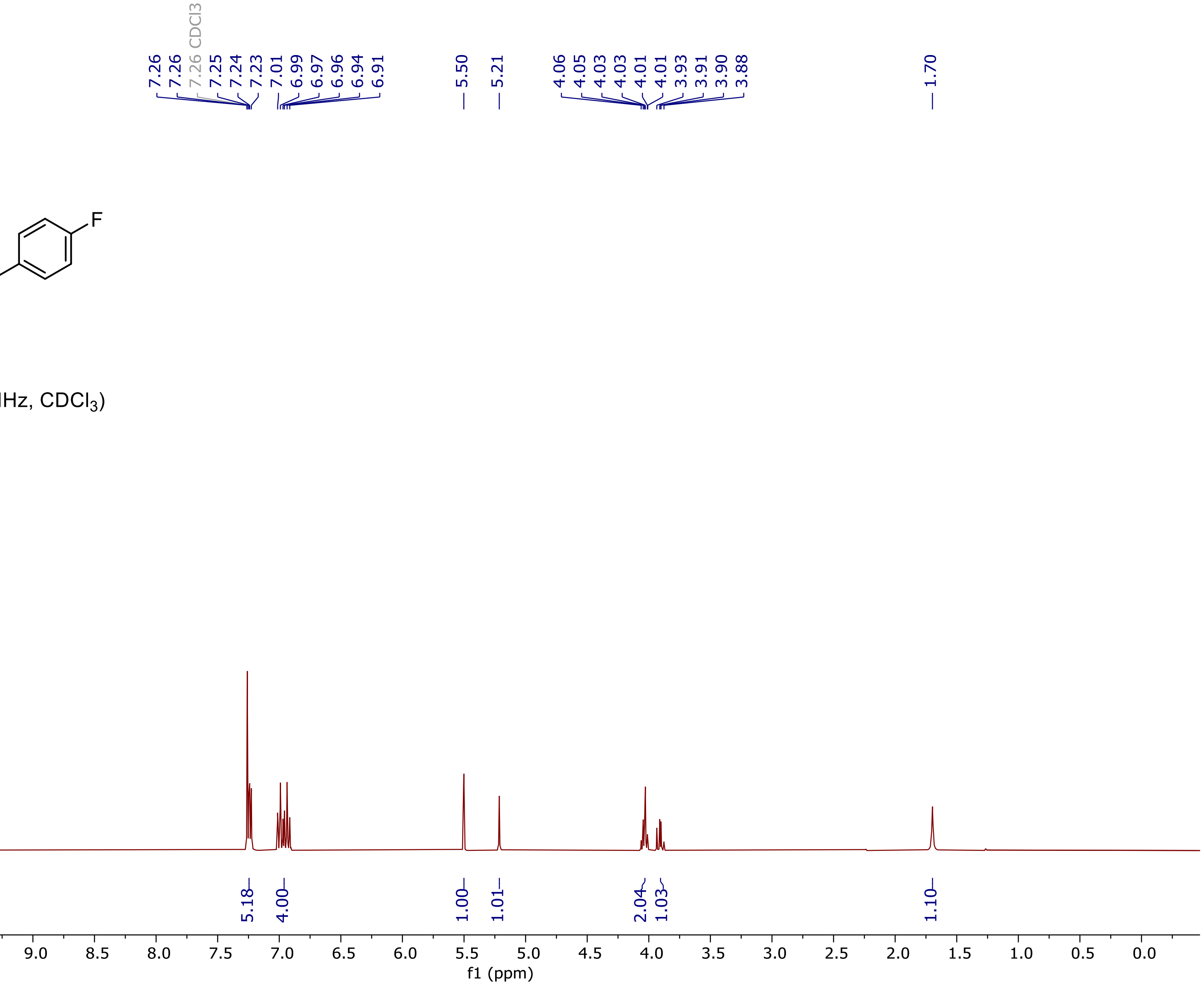

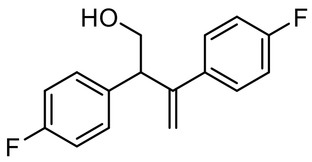

2'e

^{19}F{^{1}H} NMR (282 MHz, $CDCl_3$)

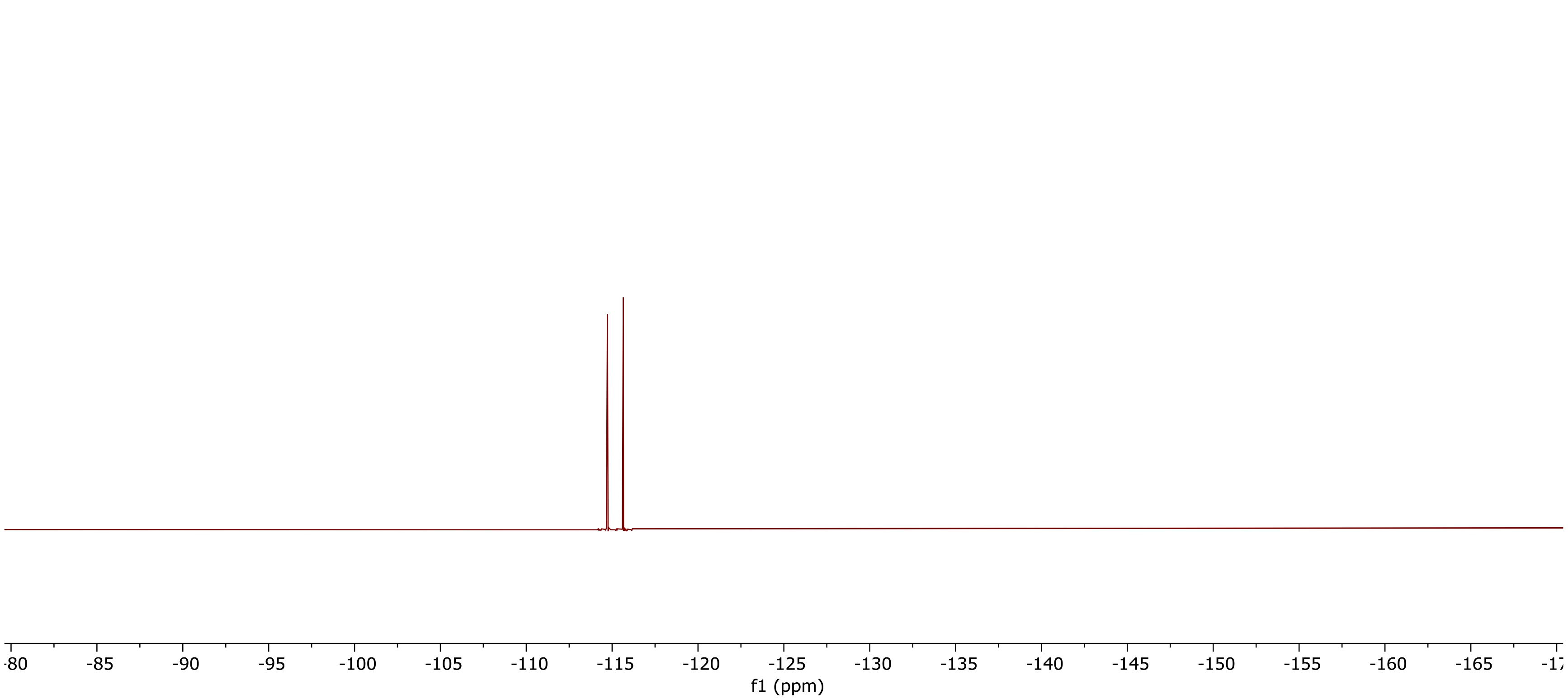

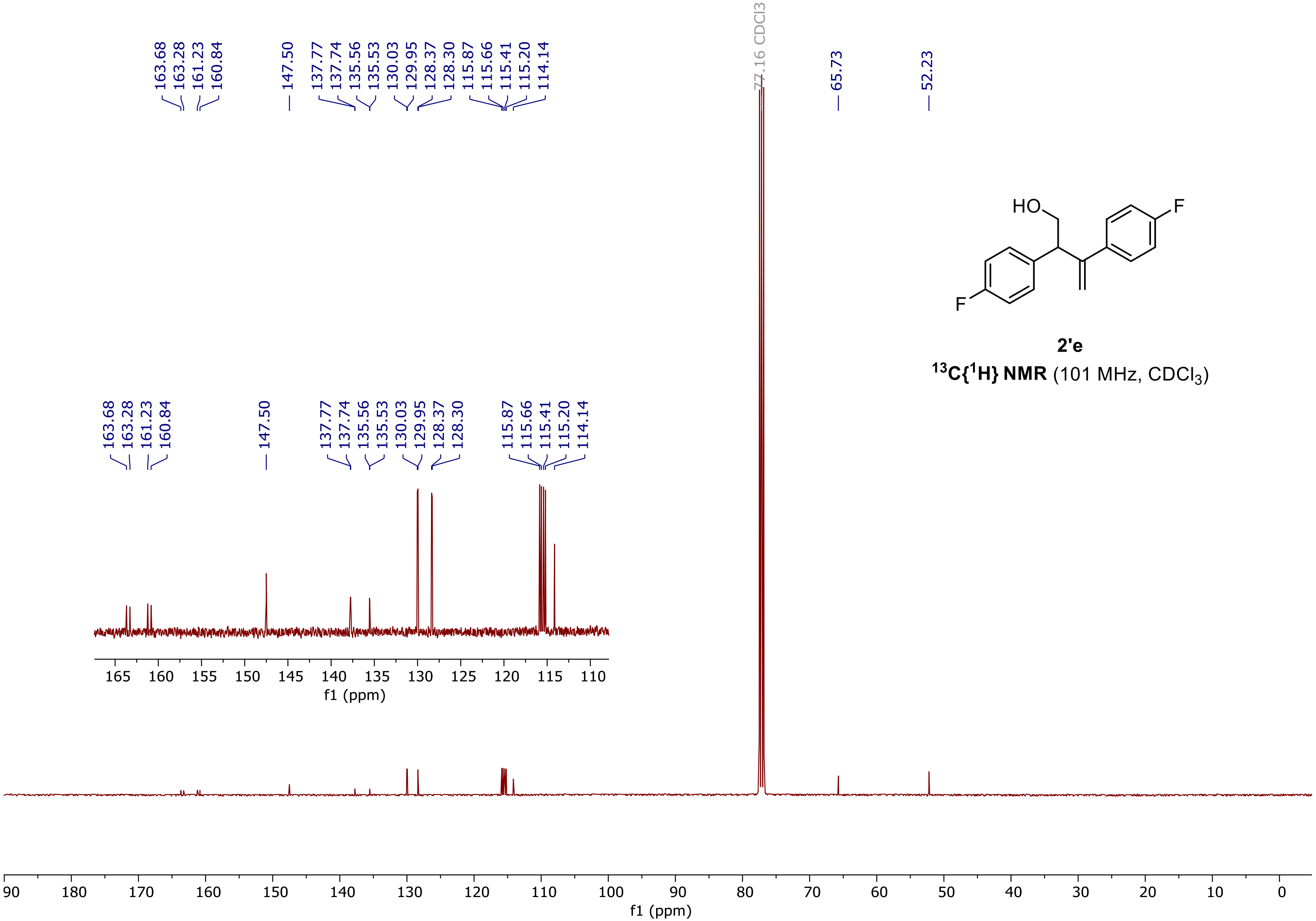

2'e

^{13}C{^{1}H} NMR (101 MHz, $CDCl_3$)

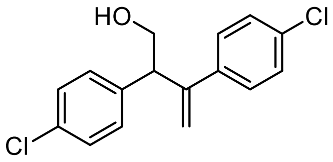

2'f

^{1}H NMR (400 MHz, $CDCl_3$)

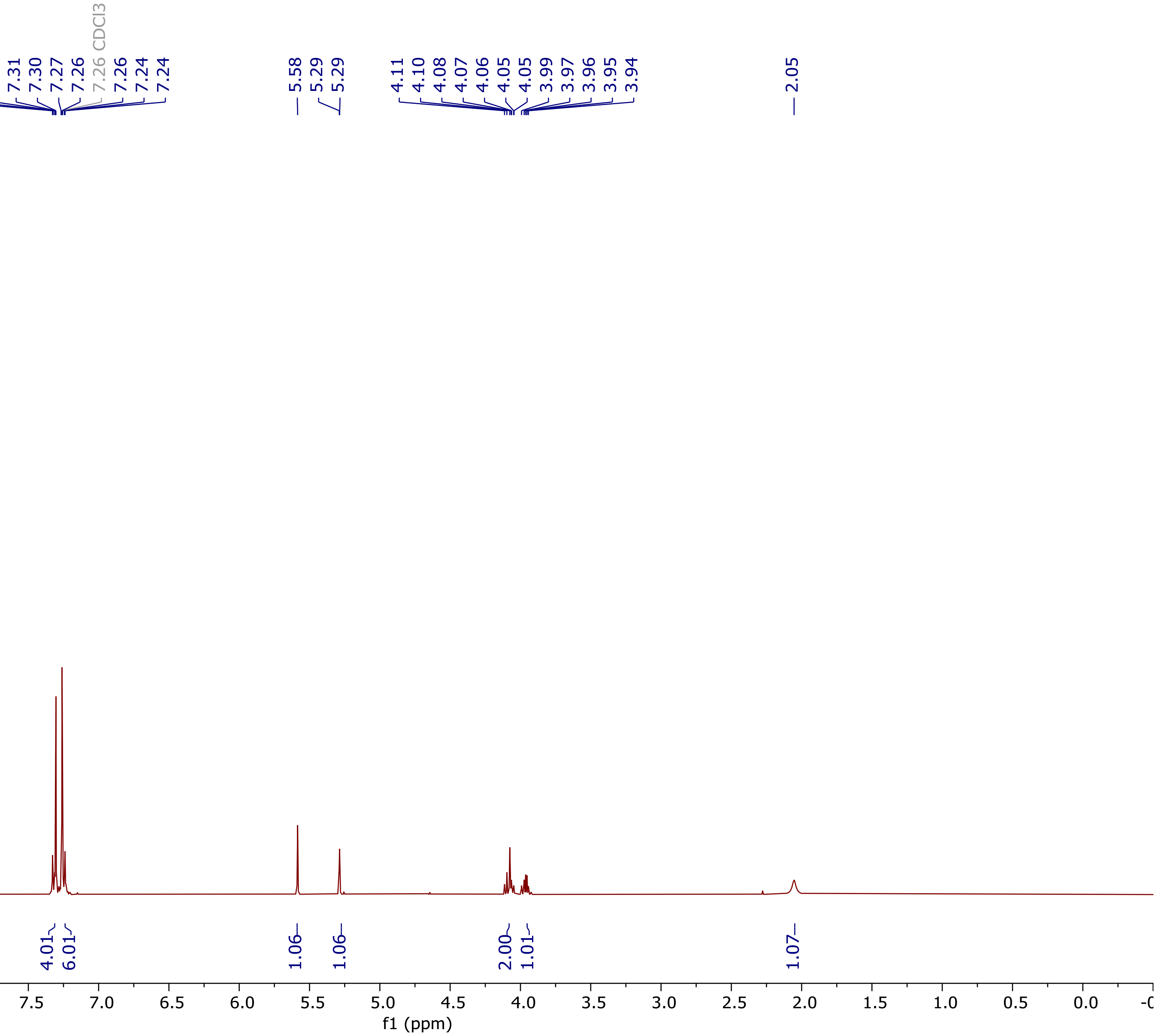

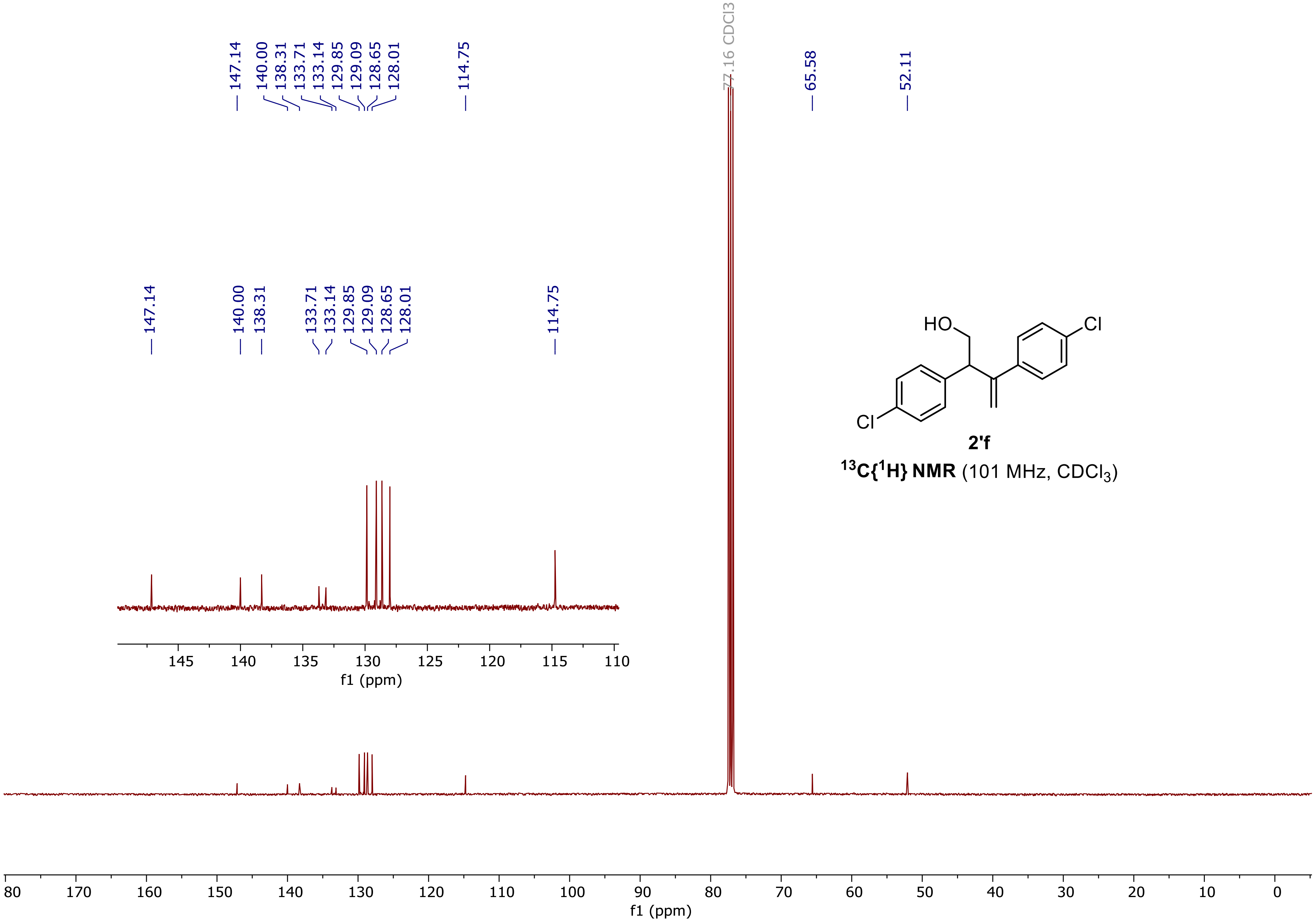

2'f

^{13}C{^{1}H} NMR (101 MHz, $CDCl_3$)

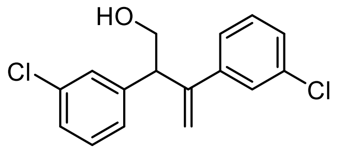

2'g

^{1}H NMR (400 MHz, $CDCl_3$)

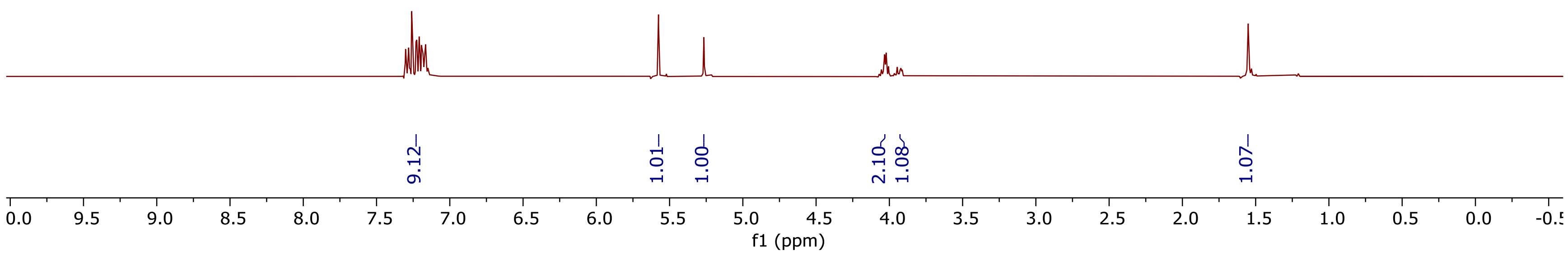

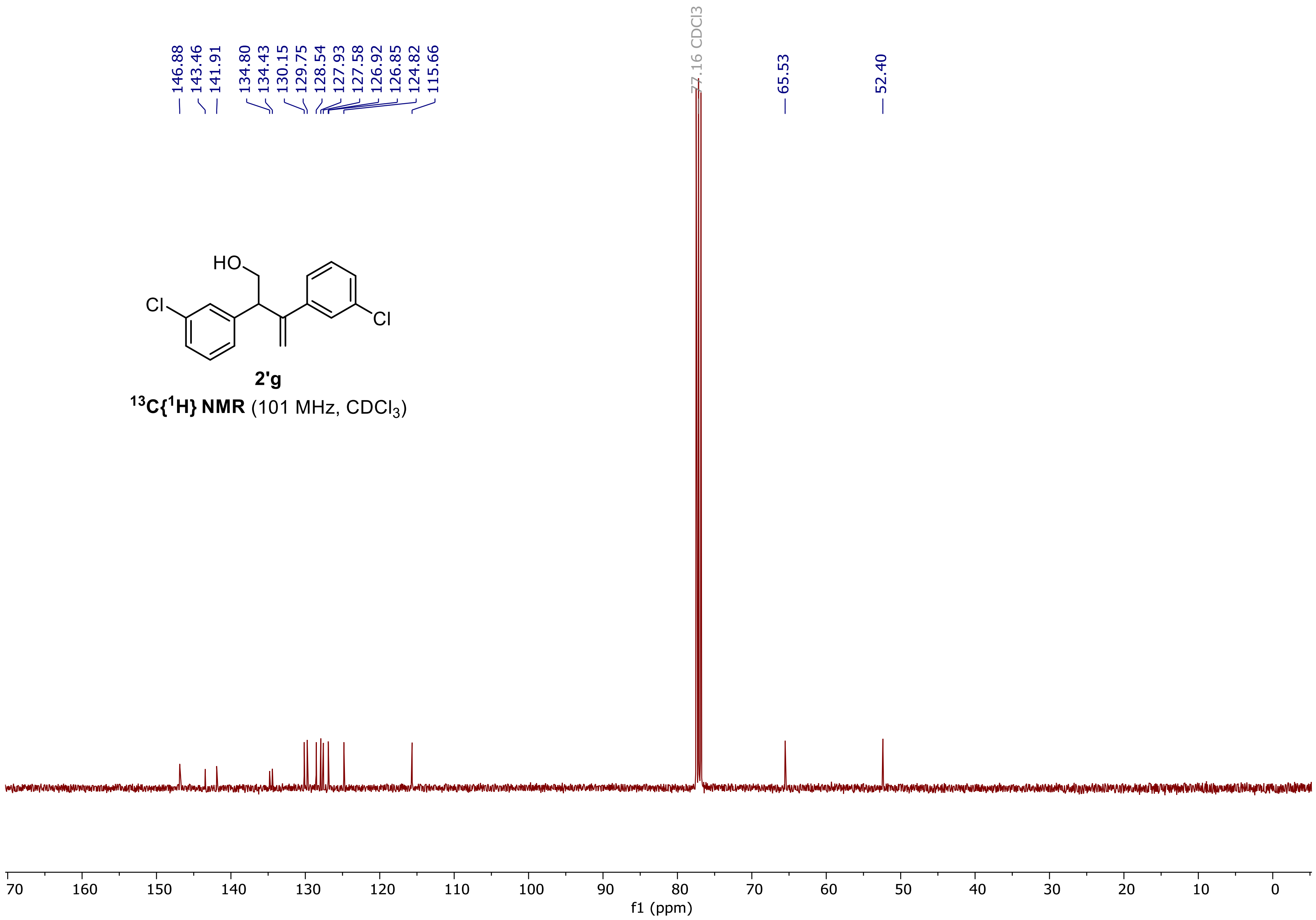

2'g

$^{13}C\{^{1}H\}$ **NMR** (101 MHz, $CDCl_3$)

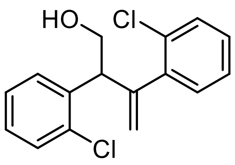

2'h

^{1}H NMR (400 MHz, $CDCl_3$)

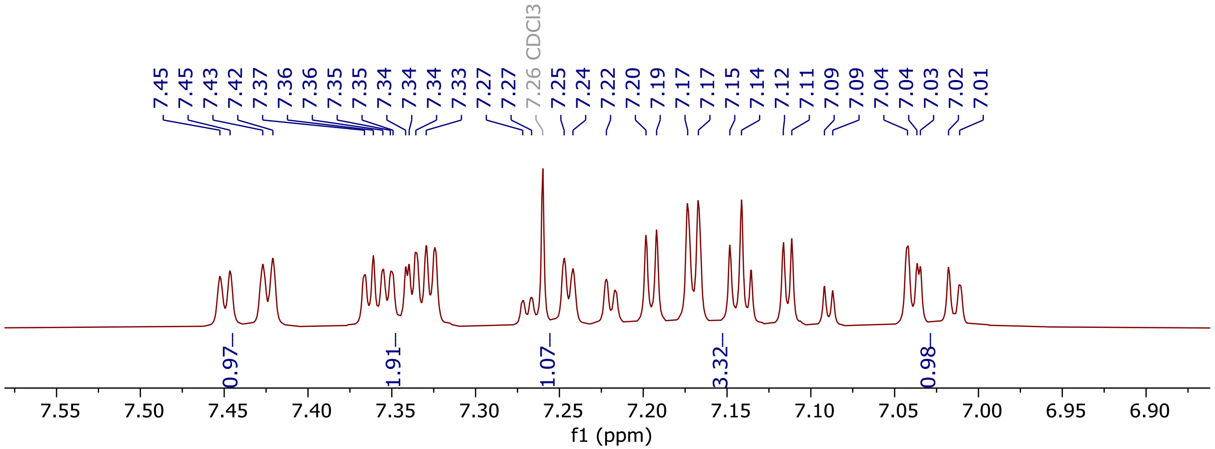

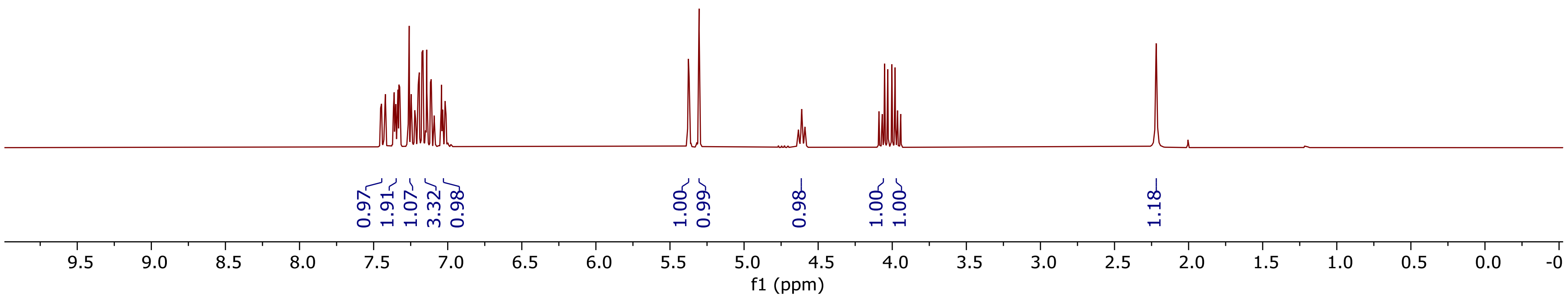

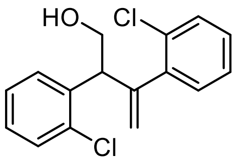

2'h

^{13}C{^{1}H} NMR (101 MHz, $CDCl_3$)

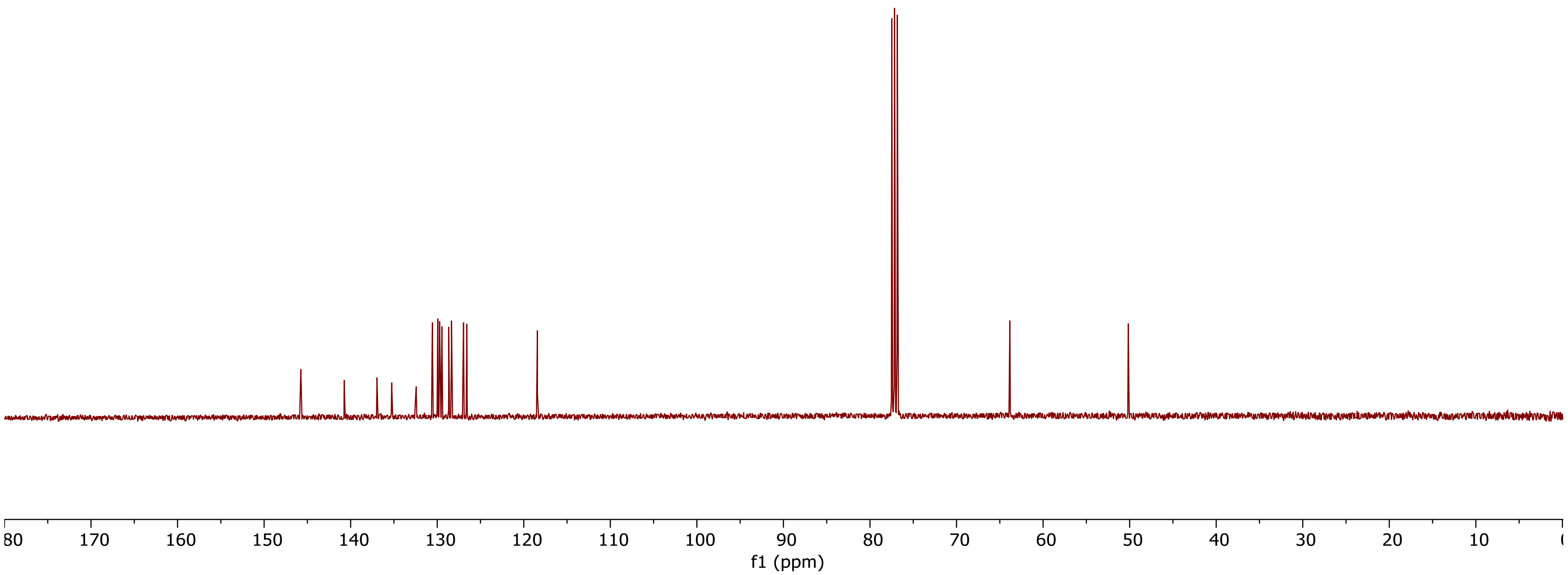

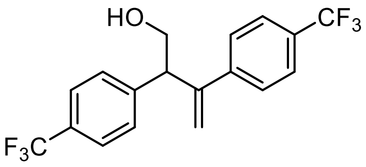

2'i

^{1}H NMR (400 MHz, $CDCl_3$)

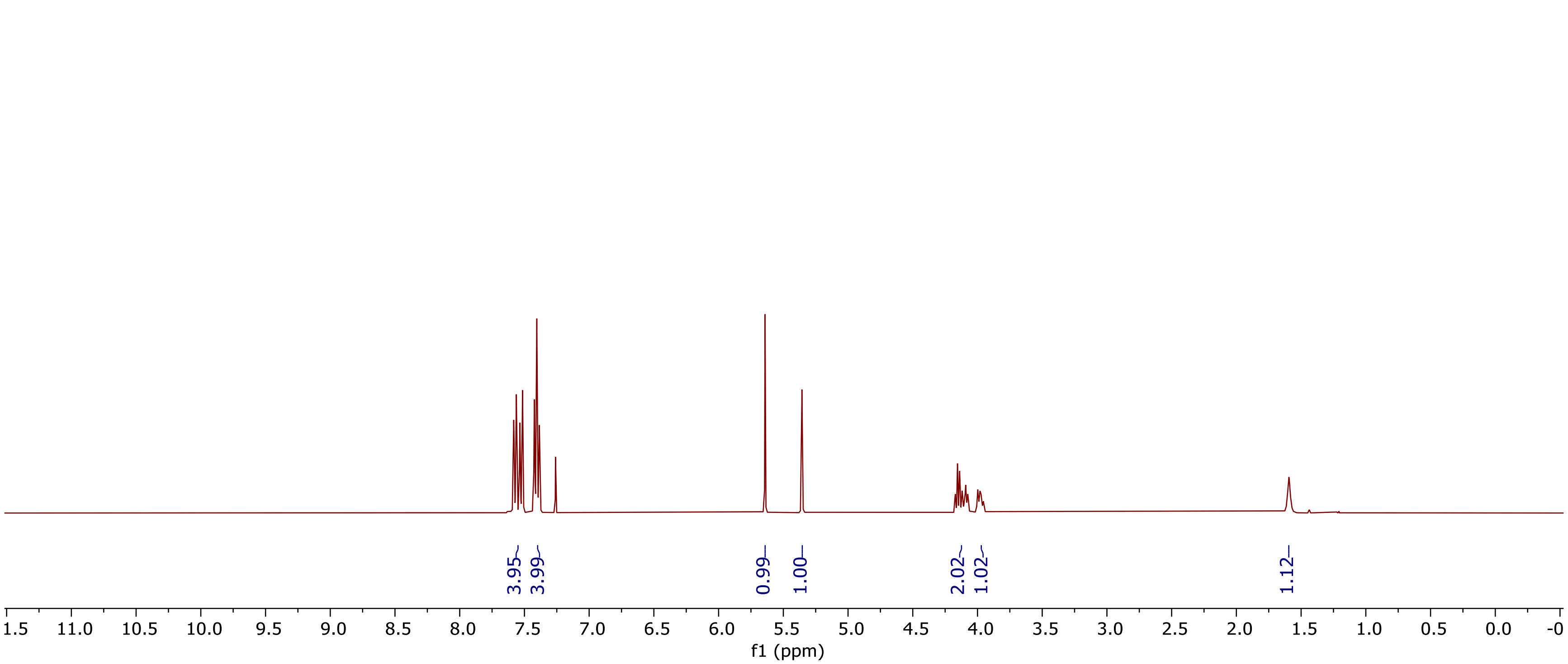

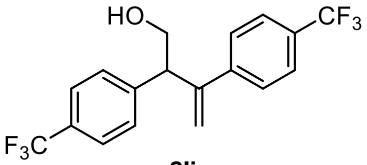

2'i

^{19}F{^{1}H} NMR (282 MHz, $CDCl_3$)

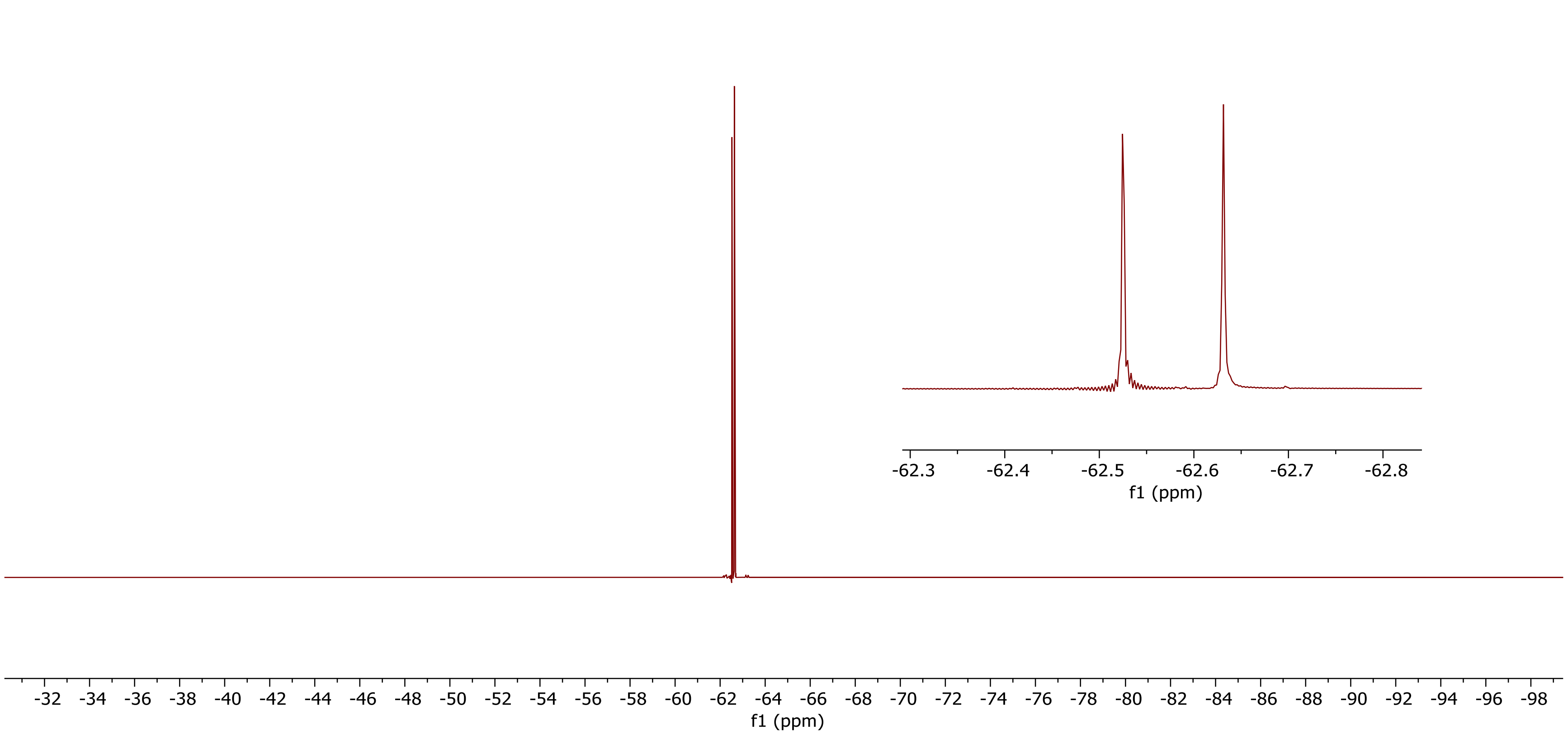

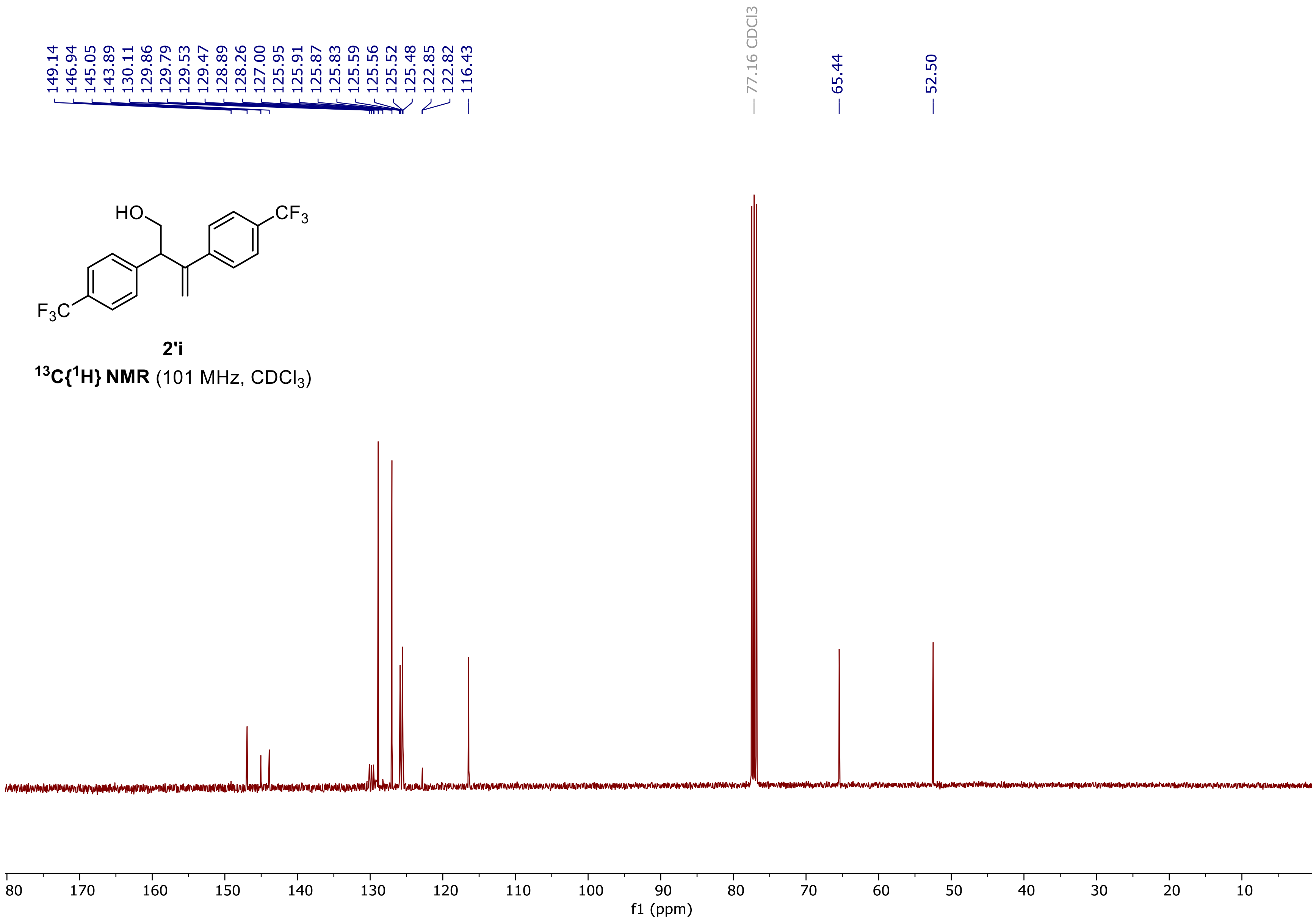

2'i

^{13}C{^{1}H} NMR (101 MHz, $CDCl_3$)

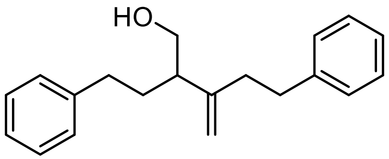

2'j

^{1}H NMR (400 MHz, $CDCl_3$)

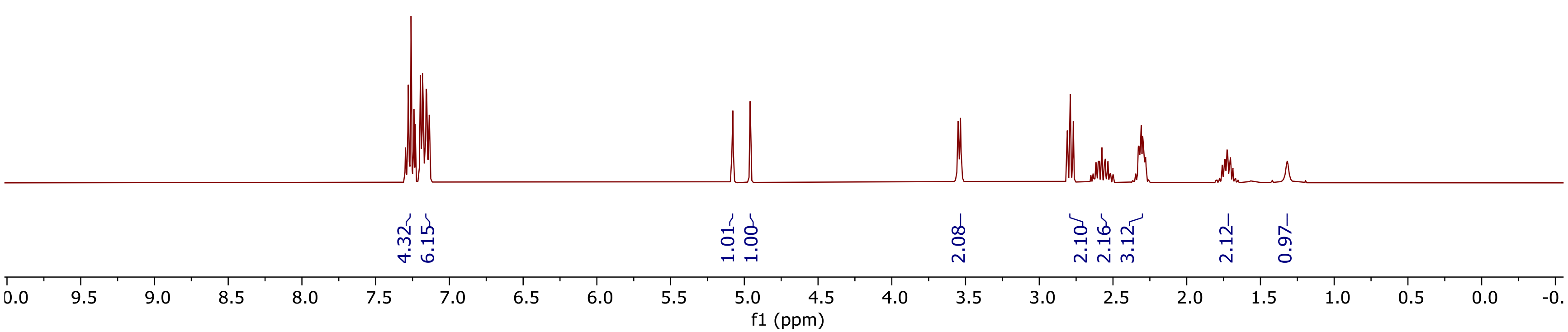

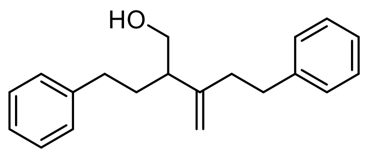

2'j

$^{13}C\{^{1}H\}$ NMR (101 MHz, $CDCl_3$)

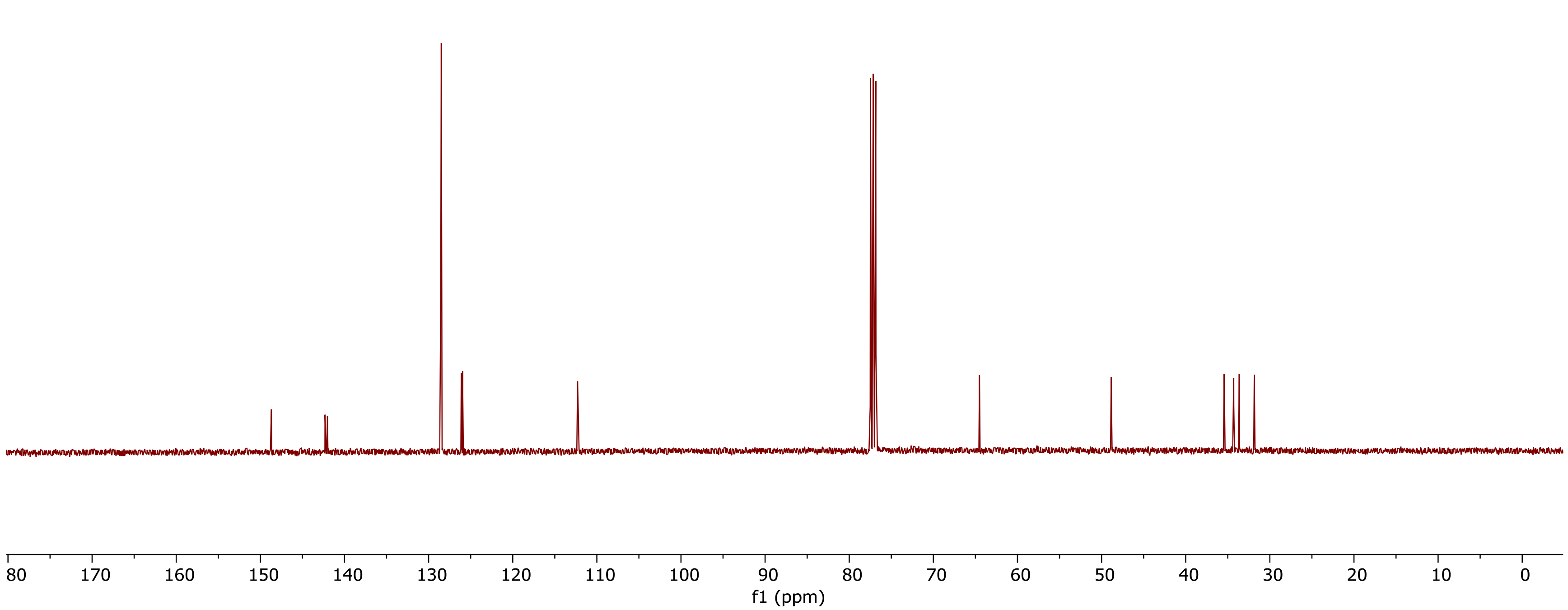

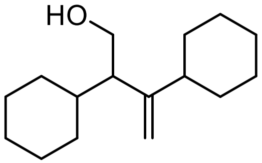

2'k

^{1}H NMR (400 MHz, $CDCl_3$)

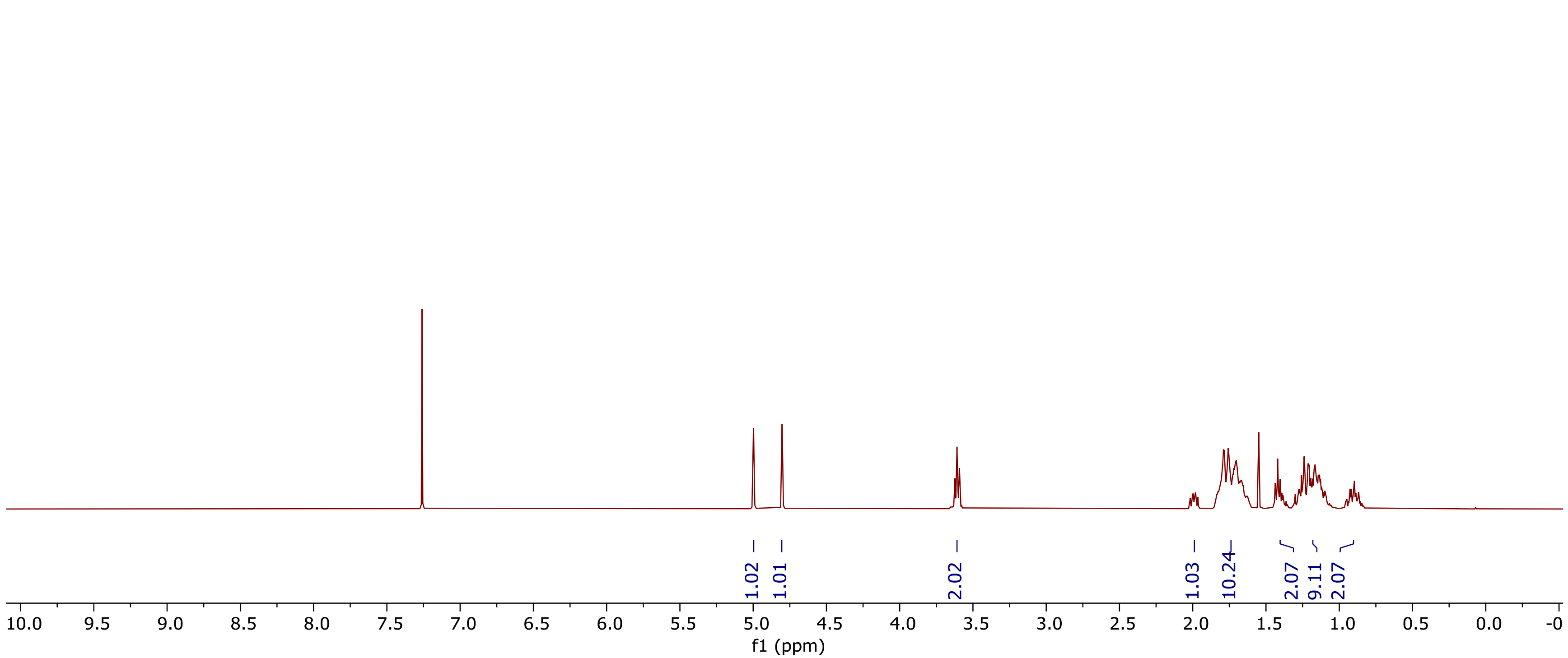

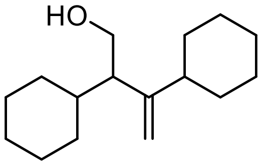

2'k

^{13}C{^{1}H} NMR (101 MHz, $CDCl_3$)

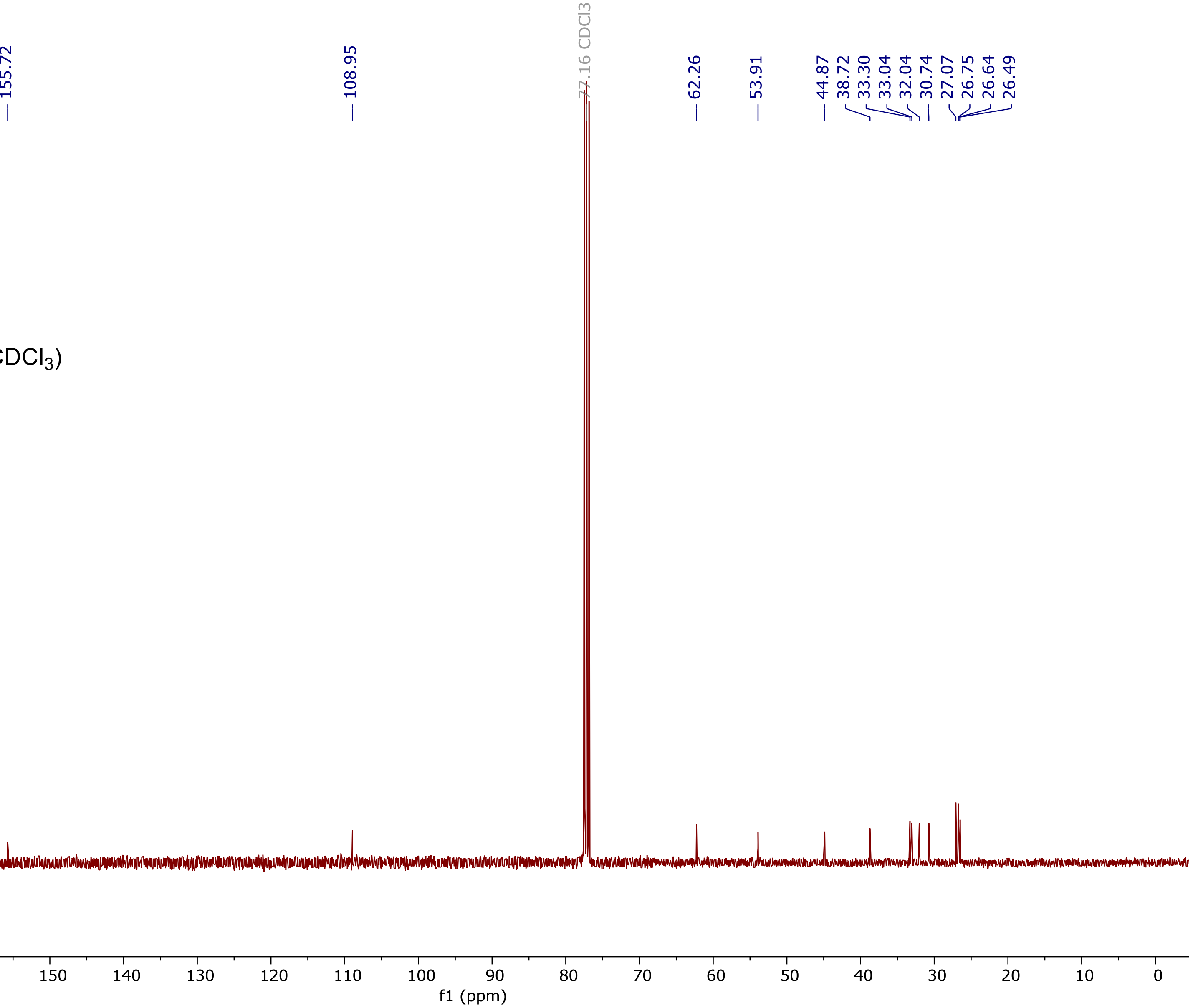

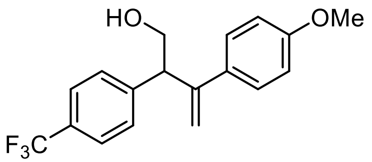

2'l

^{1}H NMR (400 MHz, $CDCl_3$)

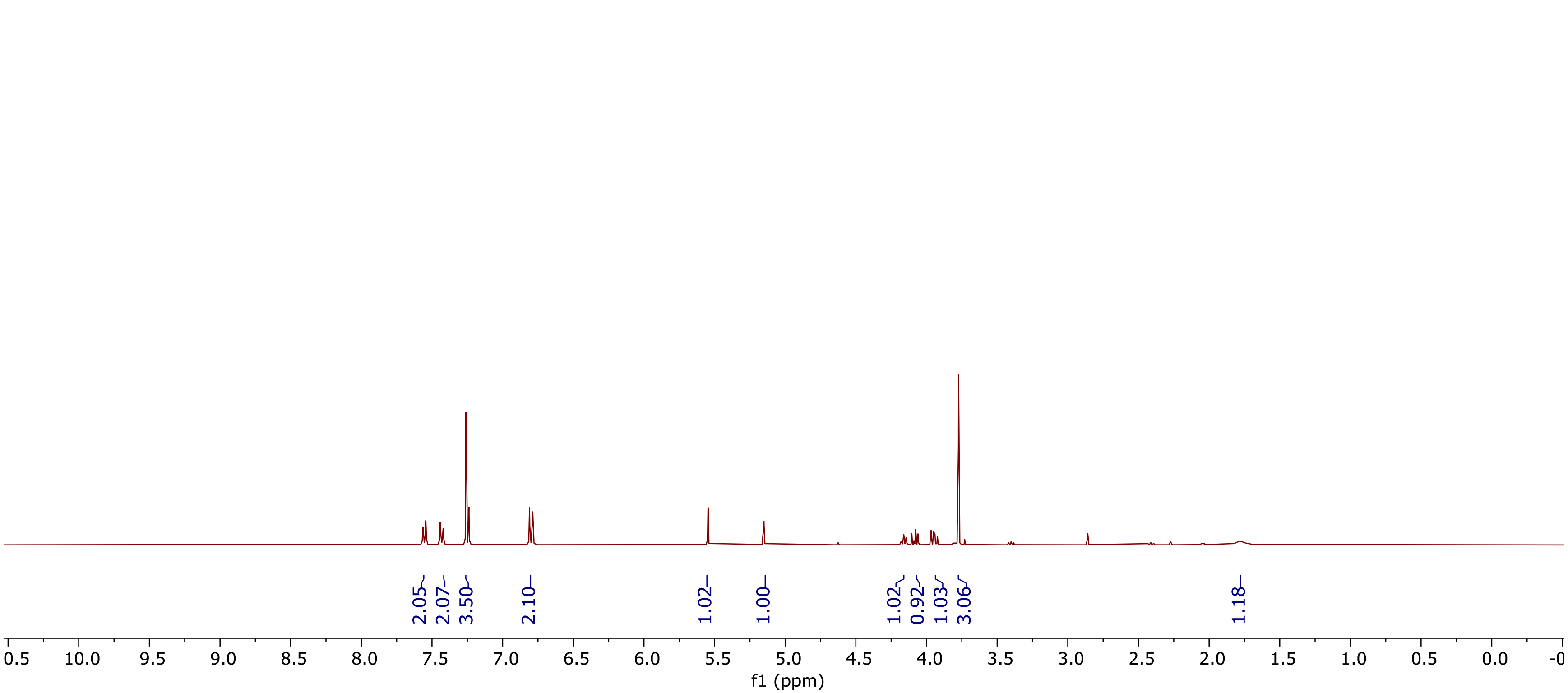

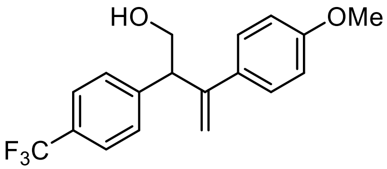

2'l

$^{19}F\{^{1}H\}$ NMR (282 MHz, $CDCl_3$)

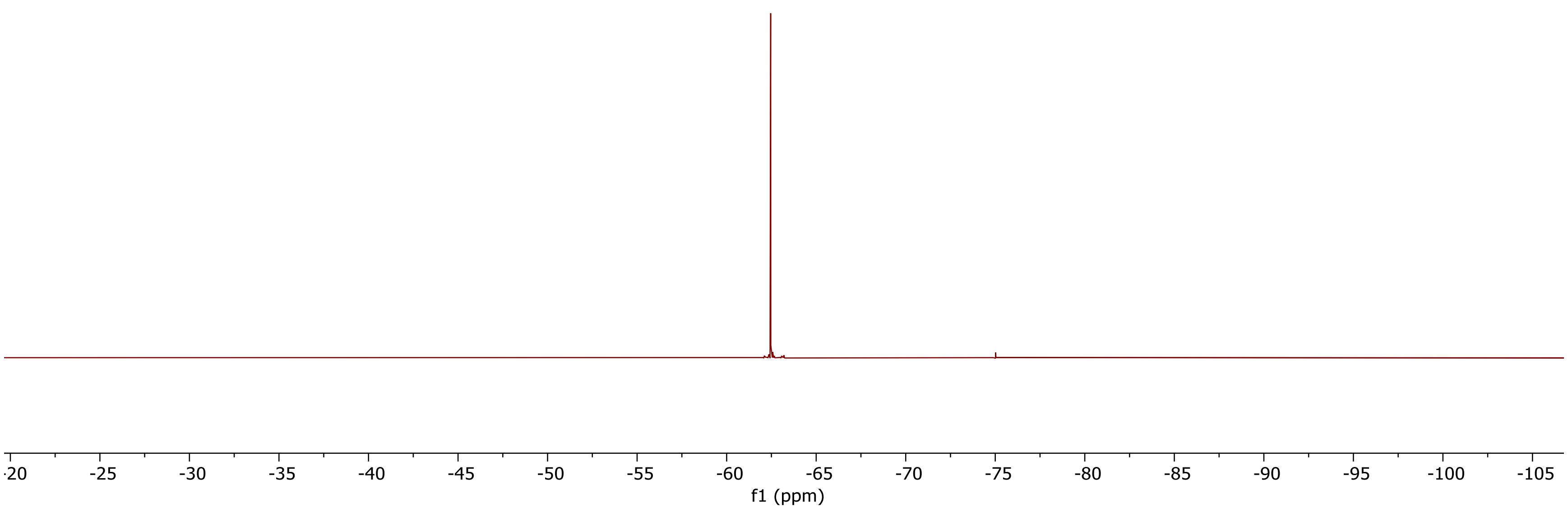

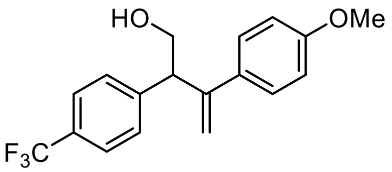

2'l

$^{13}C\{^{1}H\}$ NMR (101 MHz, $CDCl_3$)

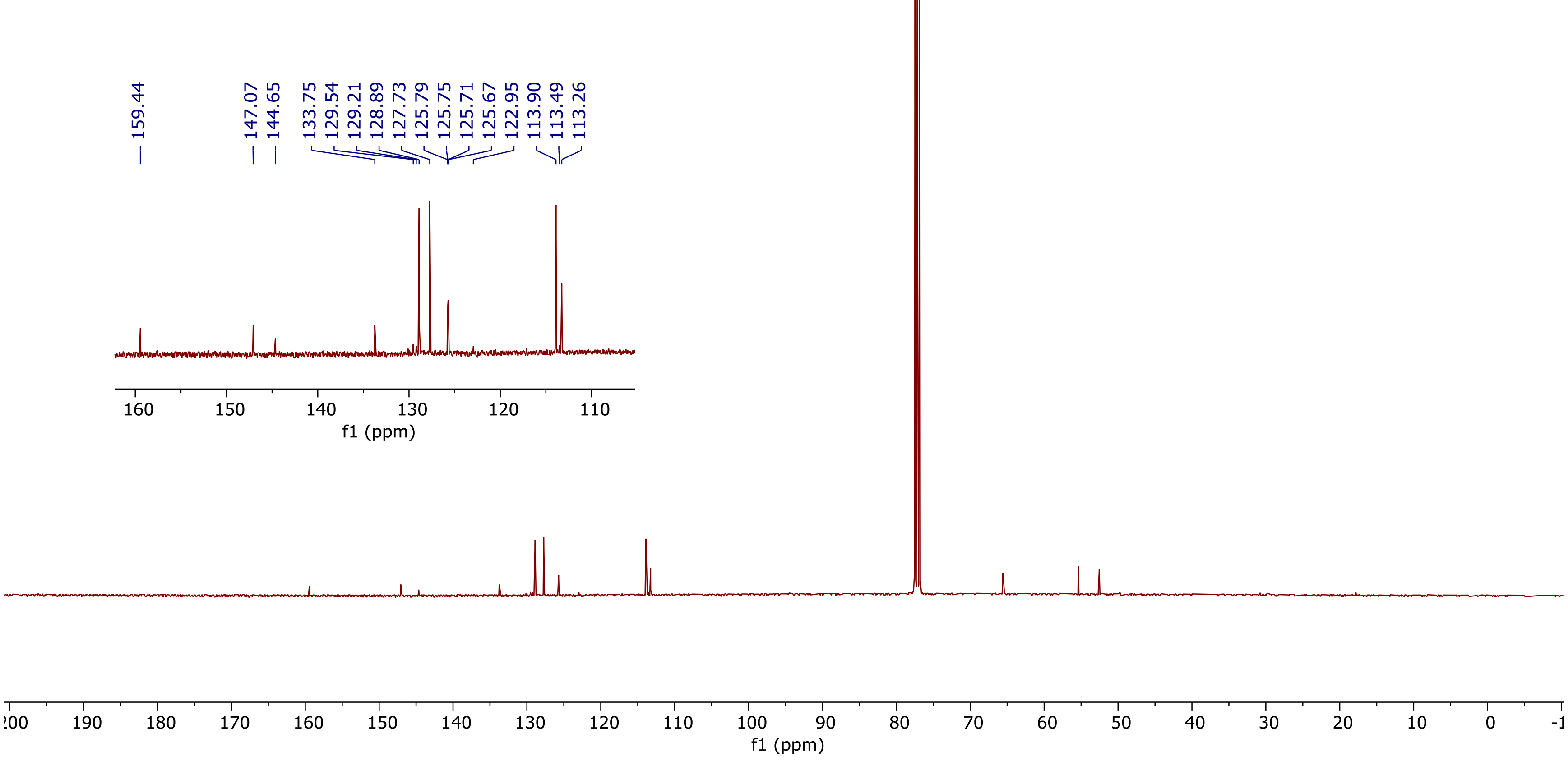

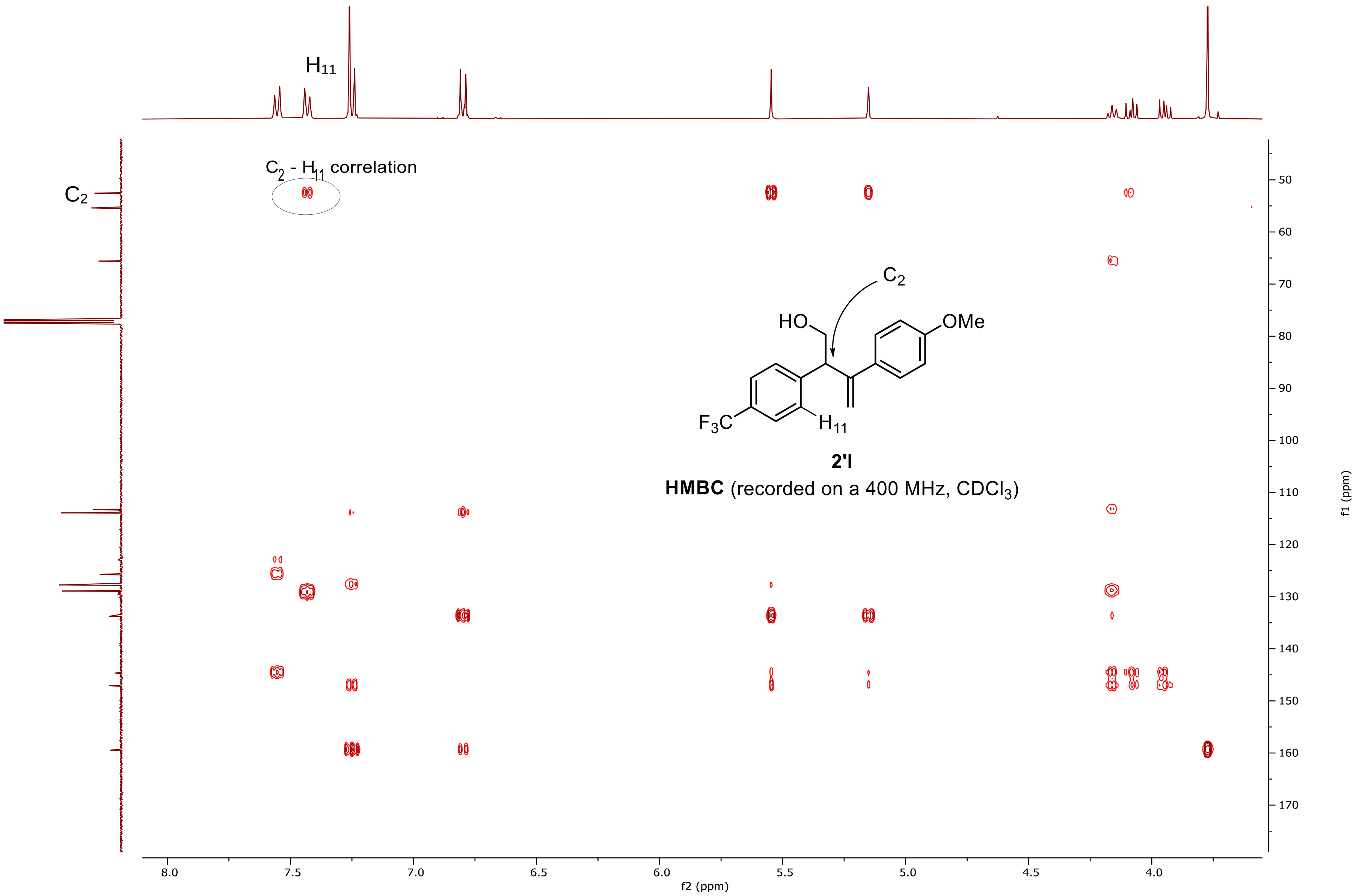

2'l

HMBC (recorded on a 400 MHz, $CDCl_3$)

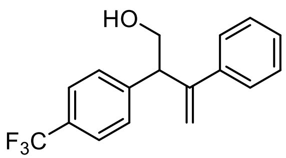

2'm

^{1}H NMR (400 MHz, $CDCl_3$)

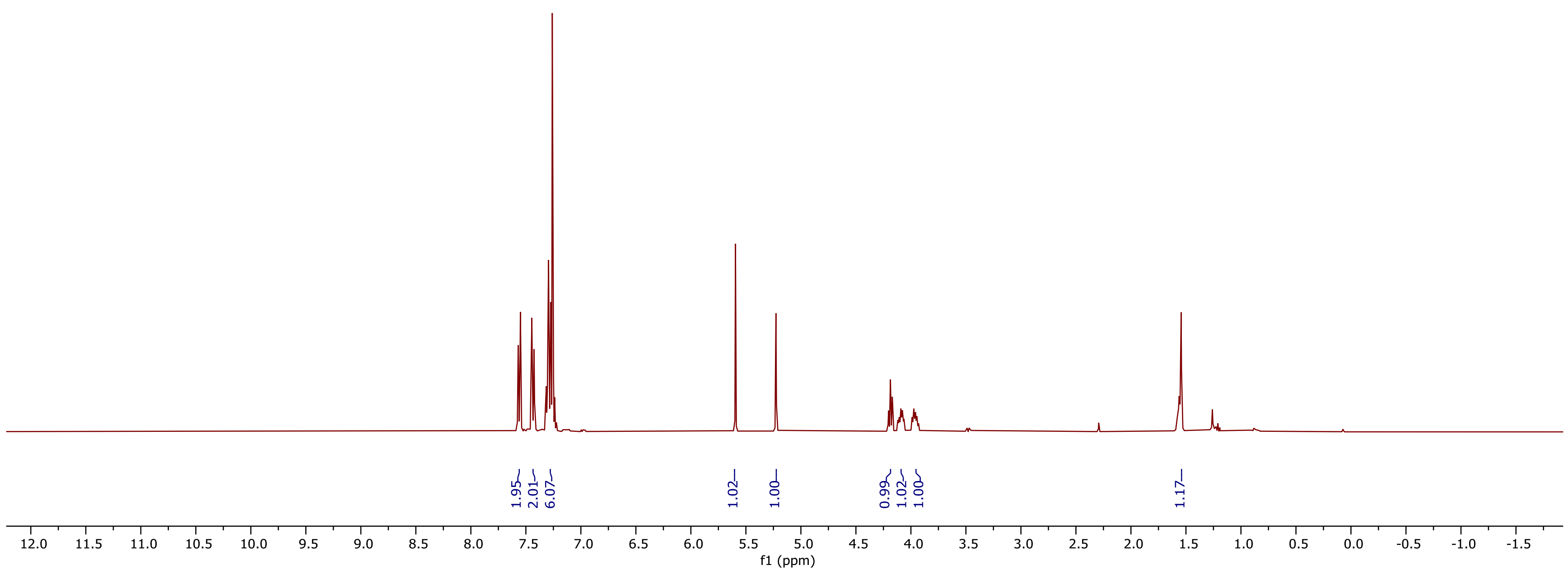

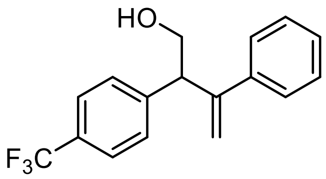

2'm

^{19}F{^{1}H} NMR (282 MHz, $CDCl_3$)

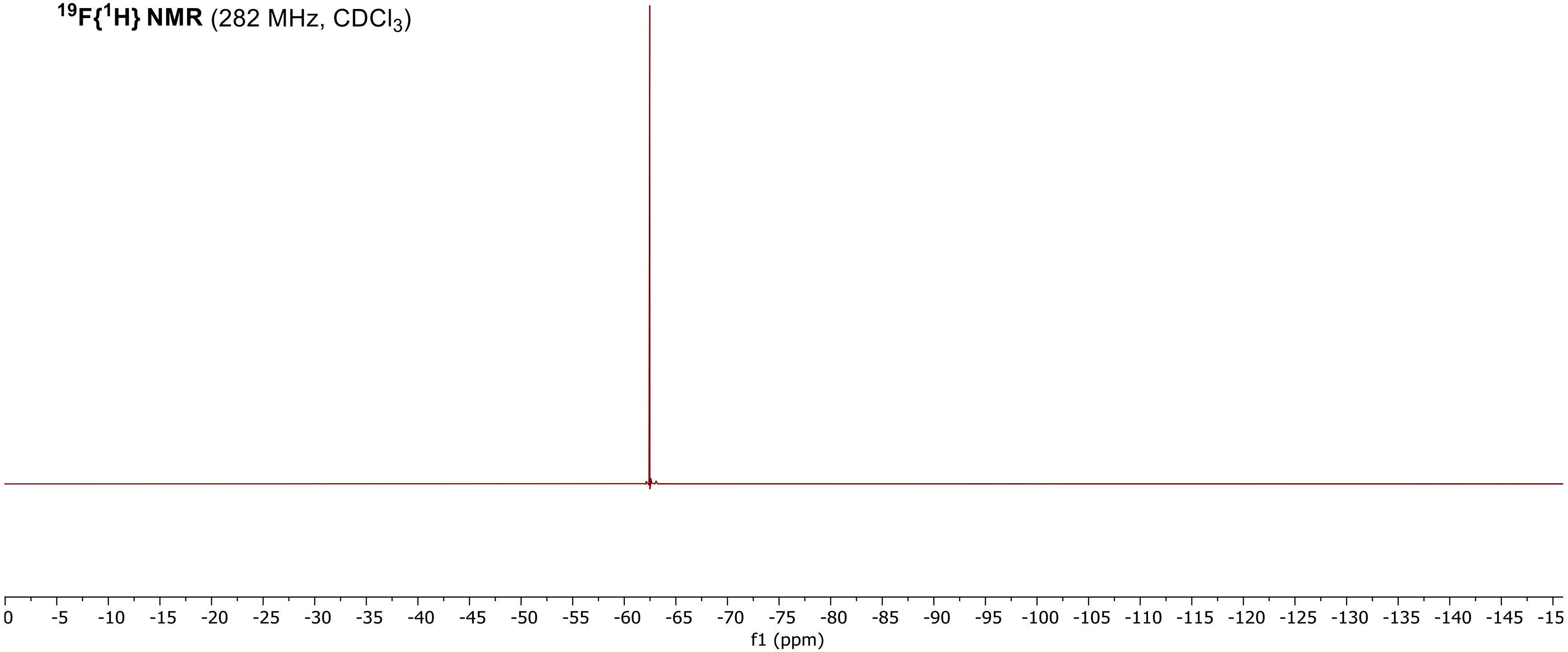

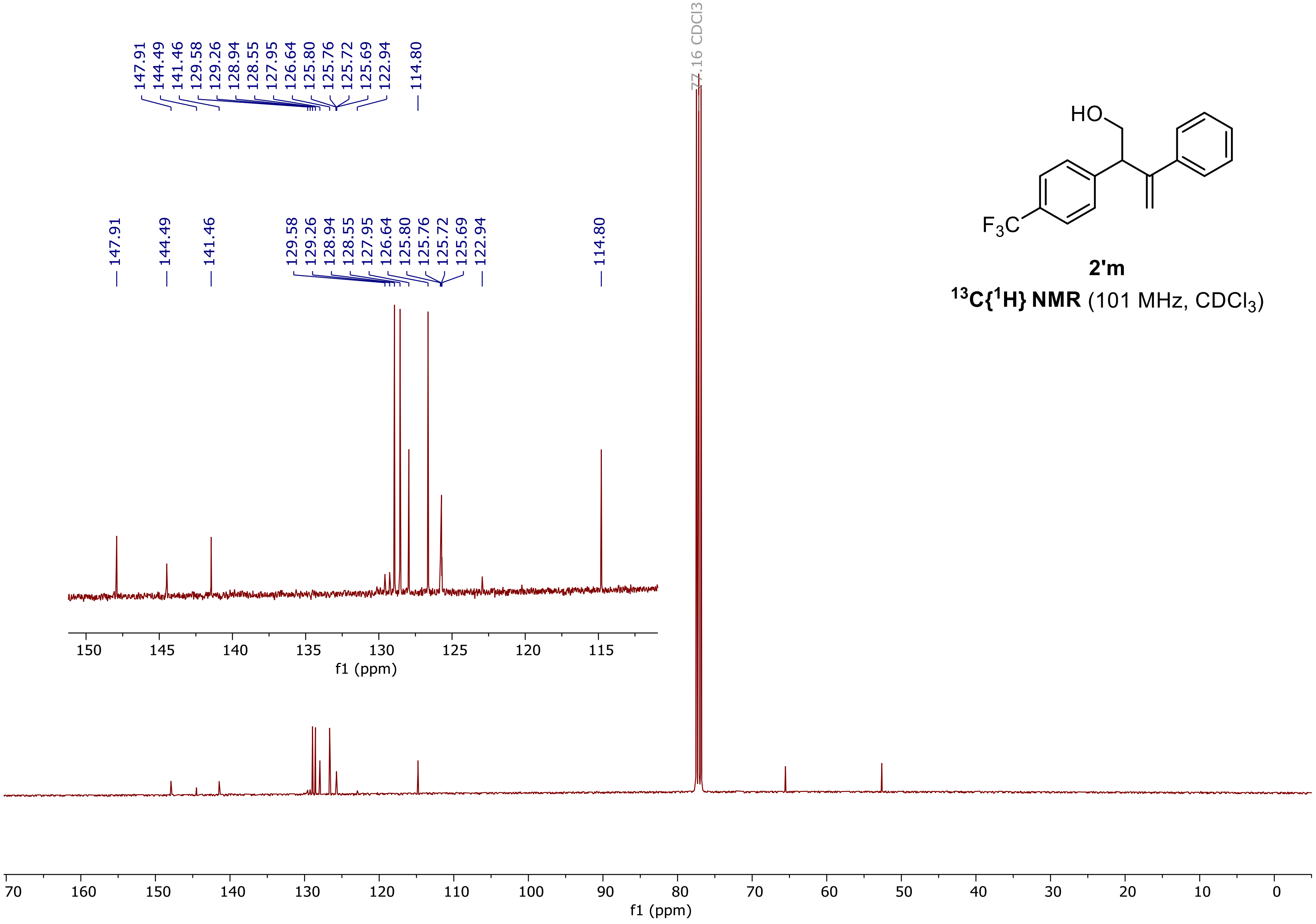

2'm

$^{13}C\{^{1}H\}$ NMR (101 MHz, $CDCl_3$)

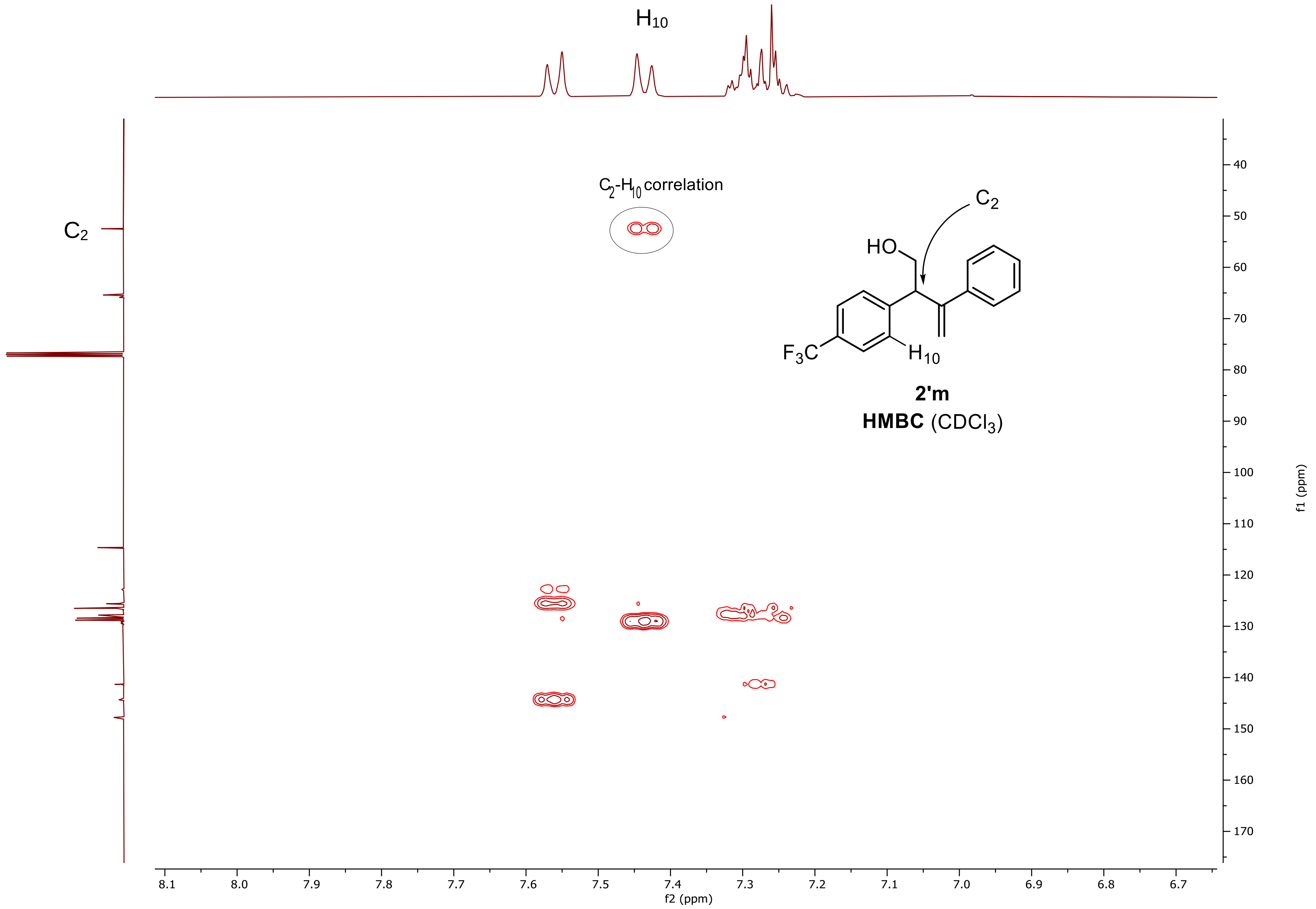
H10
C2-H10 correlation
C2
HO
F3C
H10
2'm
HMBC (CDCl3)
f1 (ppm)
f2 (ppm)

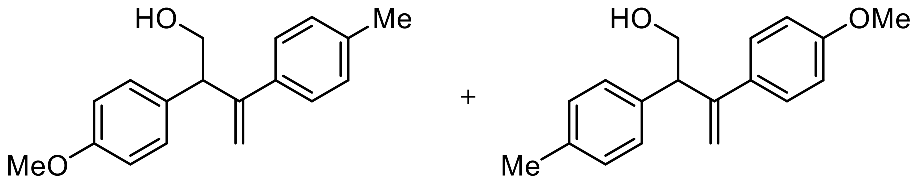

4'n **2'n**

^{1}H NMR (400 MHz, $CDCl_3$)

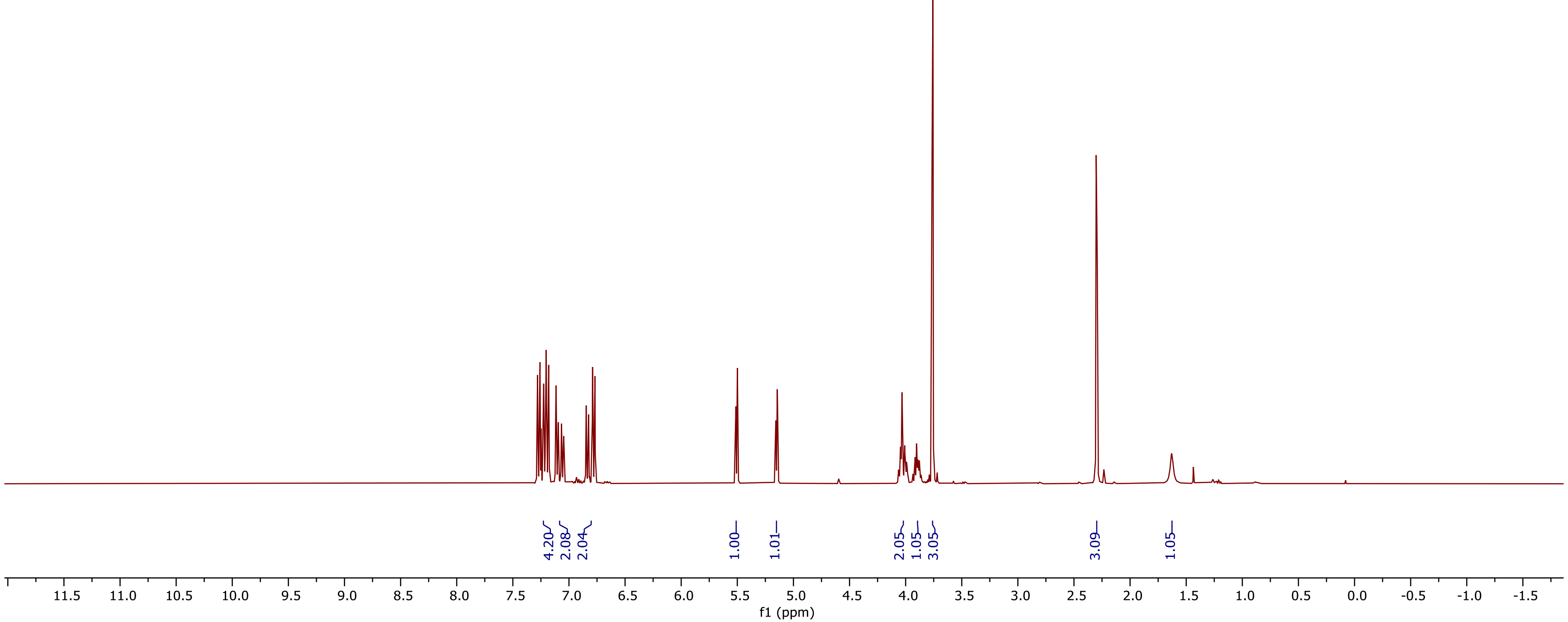

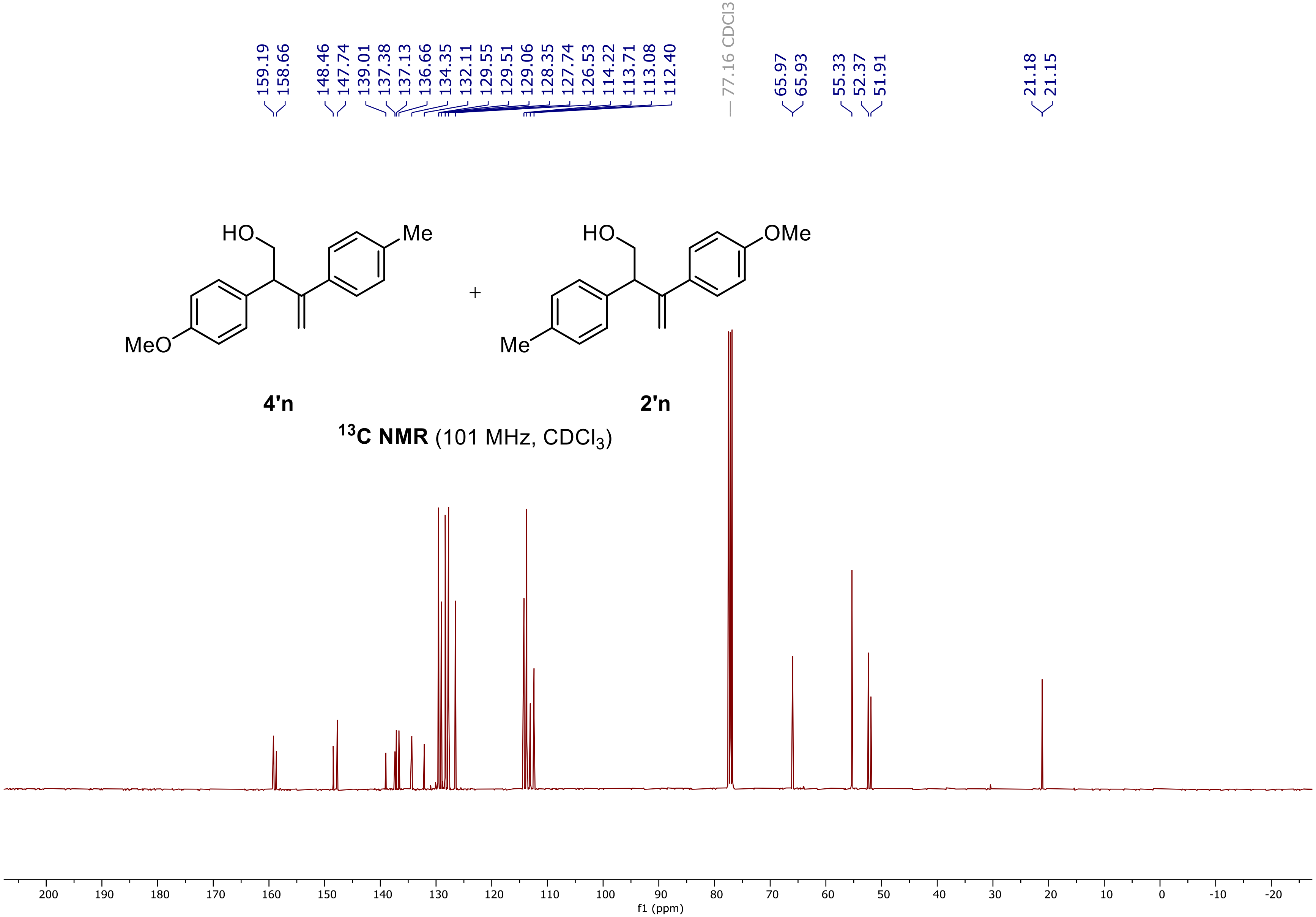

^{13}C NMR (101 MHz, $CDCl_3$)

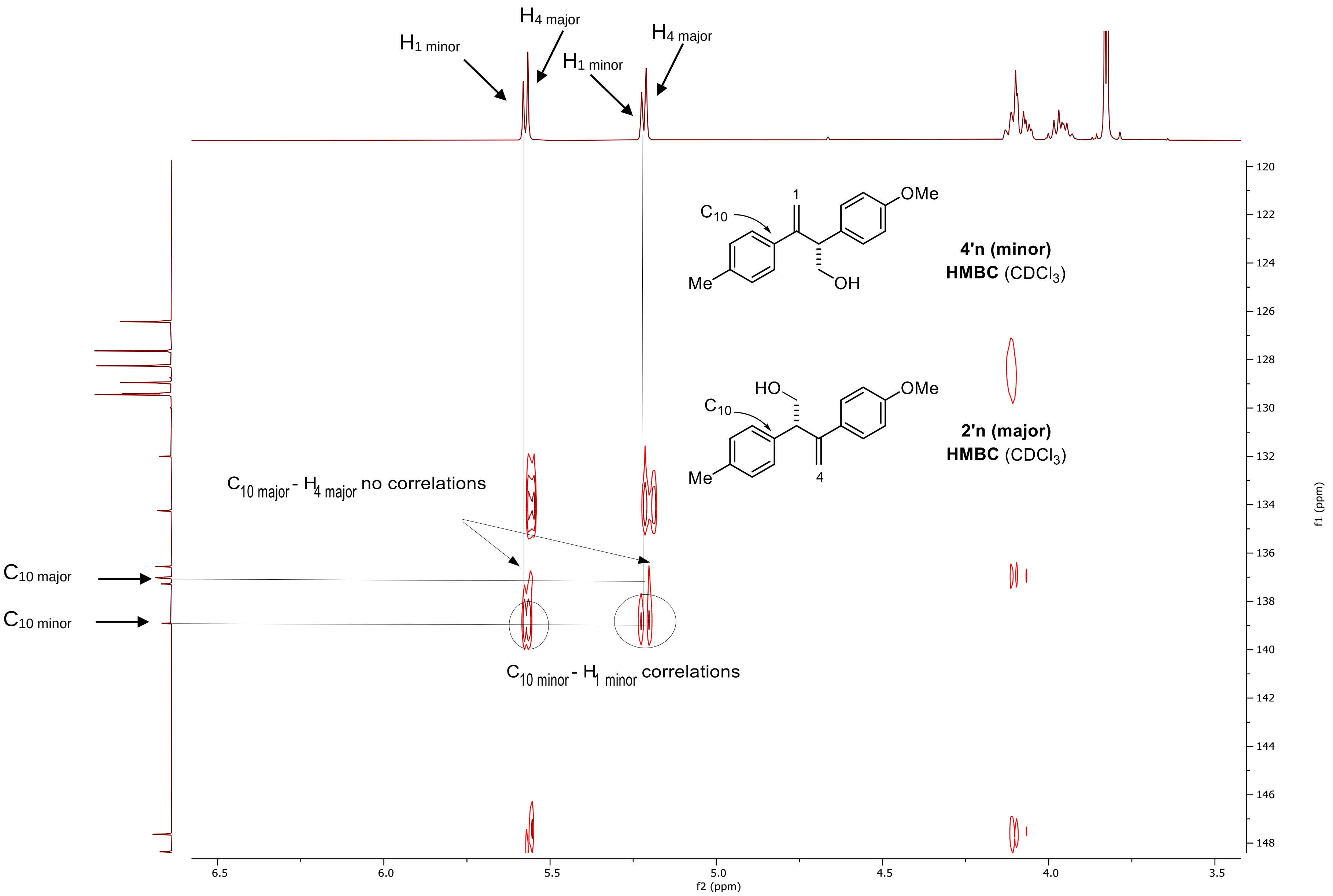
H1 minor
H4 major
H1 minor
H4 major
C10
1
OMe
Me
OH
4'n (minor)
HMBC (CDCl3)
HO
C10
OMe
Me
4
2'n (major)
HMBC (CDCl3)
C10 major - H4 major no correlations
C10 major
C10 minor
C10 minor - H1 minor correlations
f1 (ppm)
f2 (ppm)

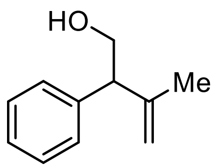

2'o

^{1}H NMR (300 MHz, $CDCl_3$)

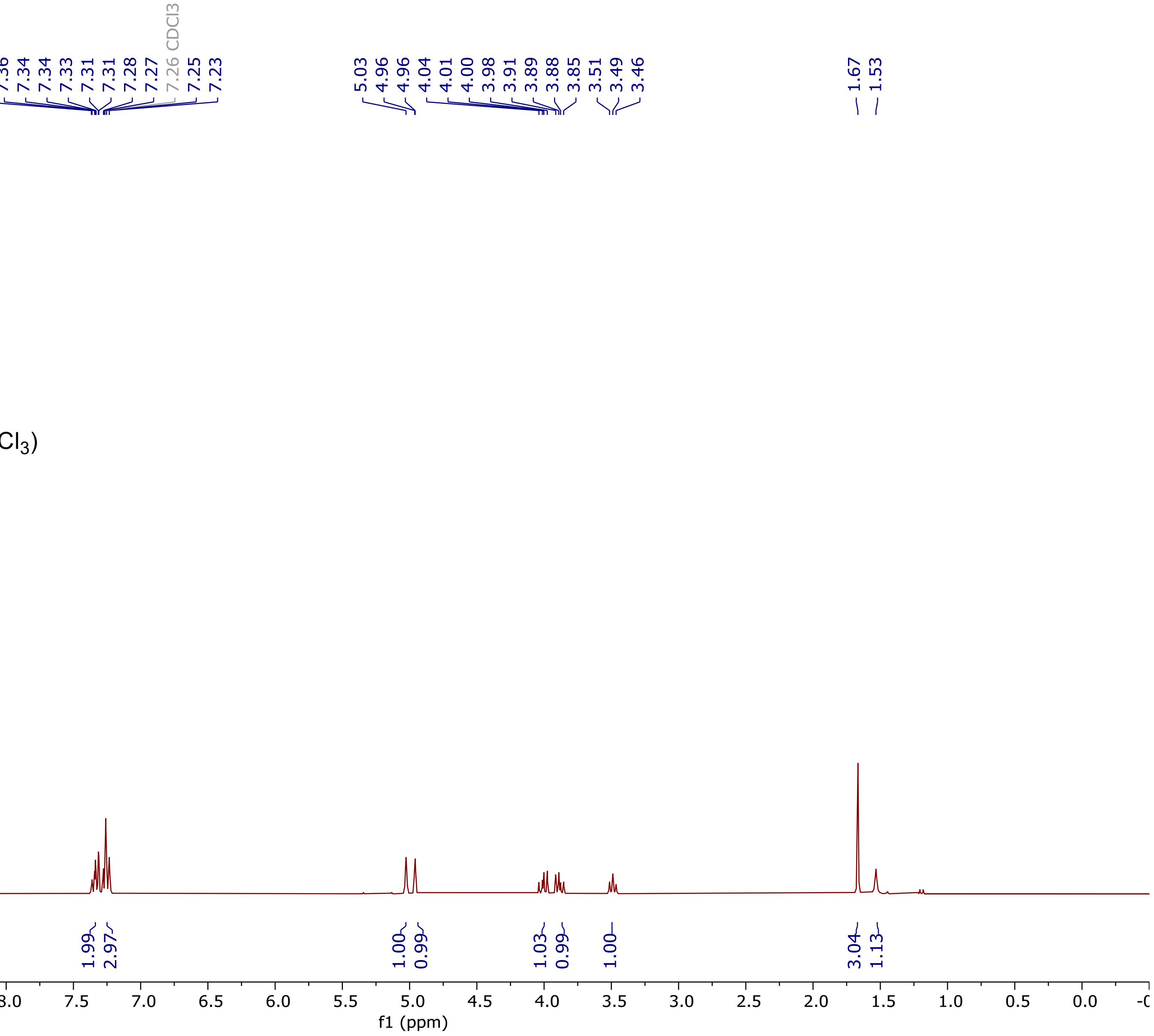

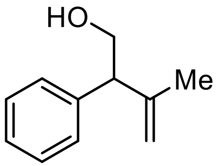

2'o

^{13}C{^{1}H} NMR (75 MHz, $CDCl_3$)

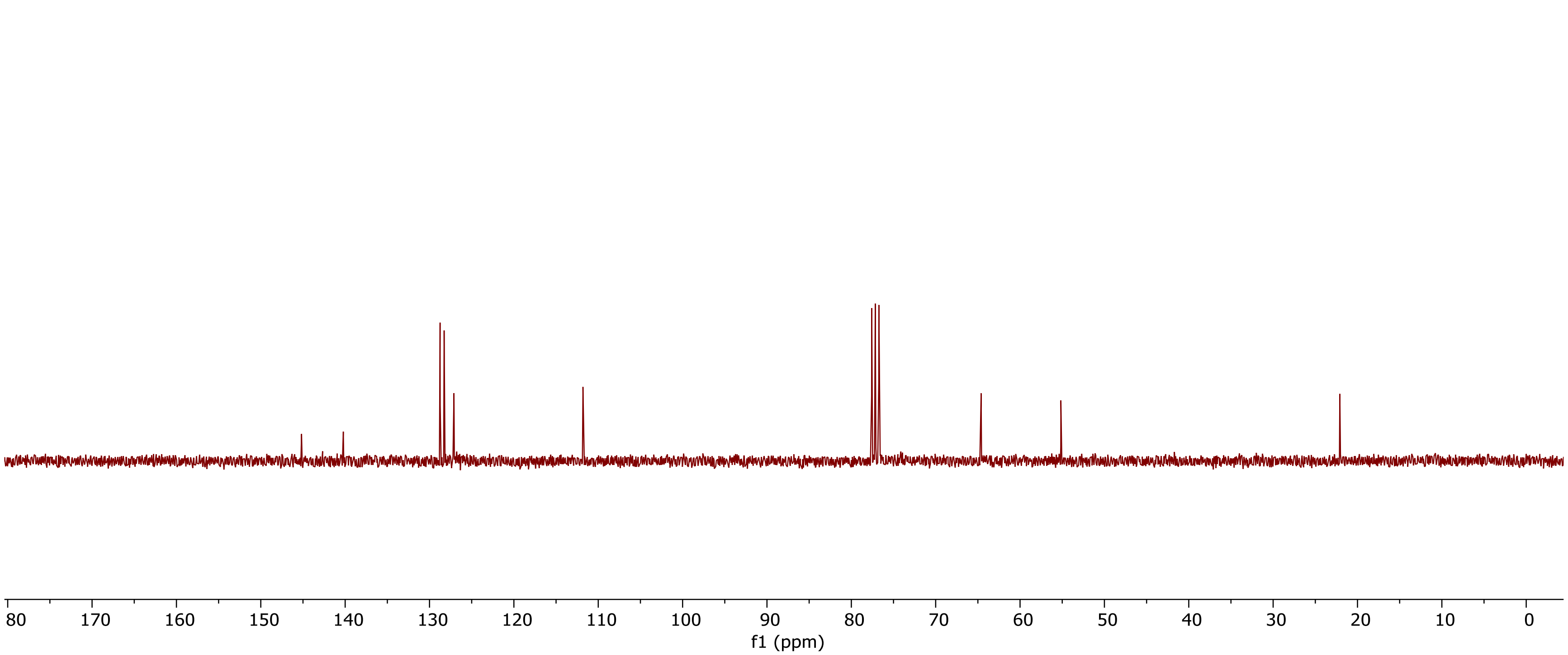

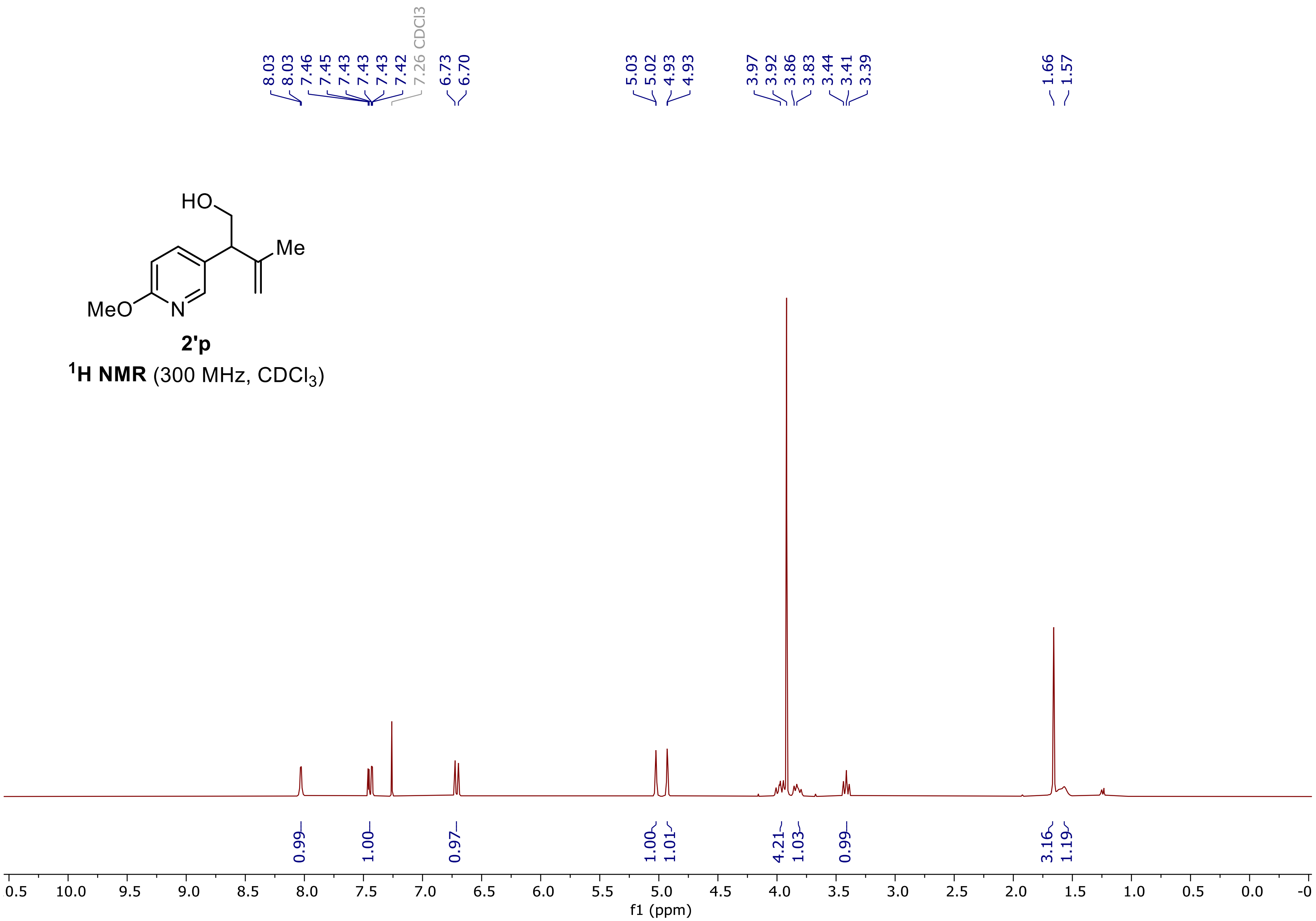
8.03
8.03
7.46
7.45
7.43
7.43
7.43
7.42
7.26 CDCl3
6.73
6.70
5.03
5.02
4.93
4.93
3.97
3.92
3.86
3.83
3.44
3.41
3.39
1.66
1.57
HO
Me
MeO
N
2'p
1H NMR (300 MHz, CDCl3)
0.99
1.00
0.97
1.00
1.01
4.21
1.03
0.99
3.16
1.19
0.5
10.0
9.5
9.0
8.5
8.0
7.5
7.0
6.5
6.0
5.5
5.0
4.5
4.0
3.5
3.0
2.5
2.0
1.5
1.0
0.5
0.0
-0
f1 (ppm)

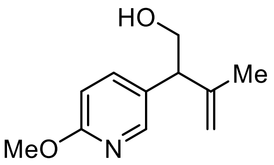

2'p

^{13}**C{^{1}H} NMR** (75 MHz, $CDCl_3$)

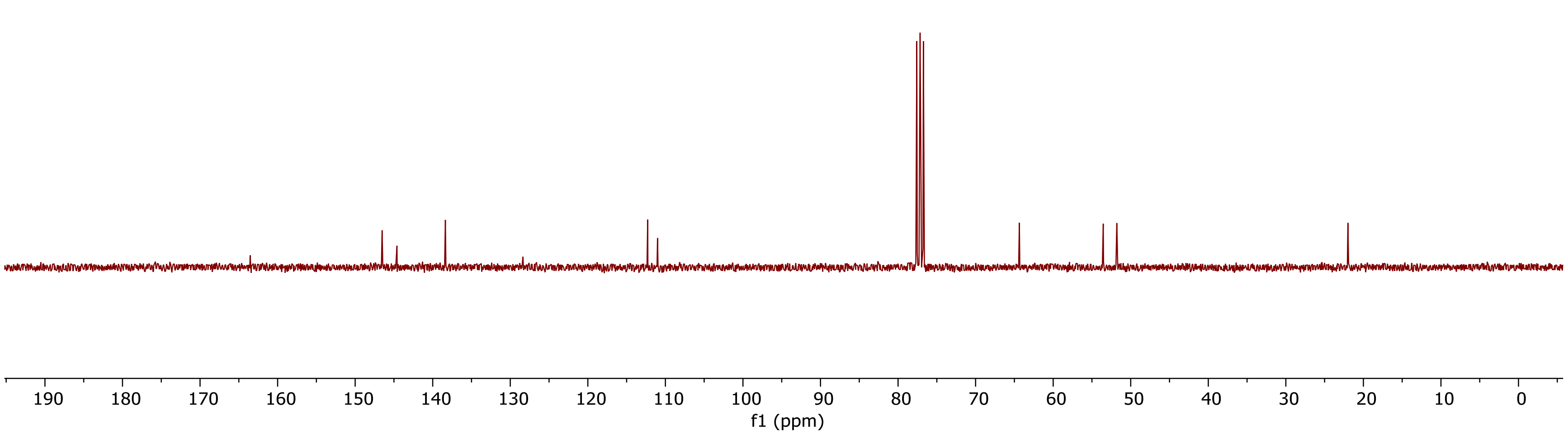

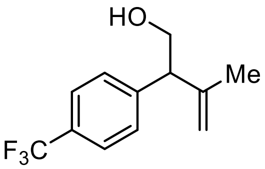

2'q

^{1}H NMR (400 MHz, $CDCl_3$)

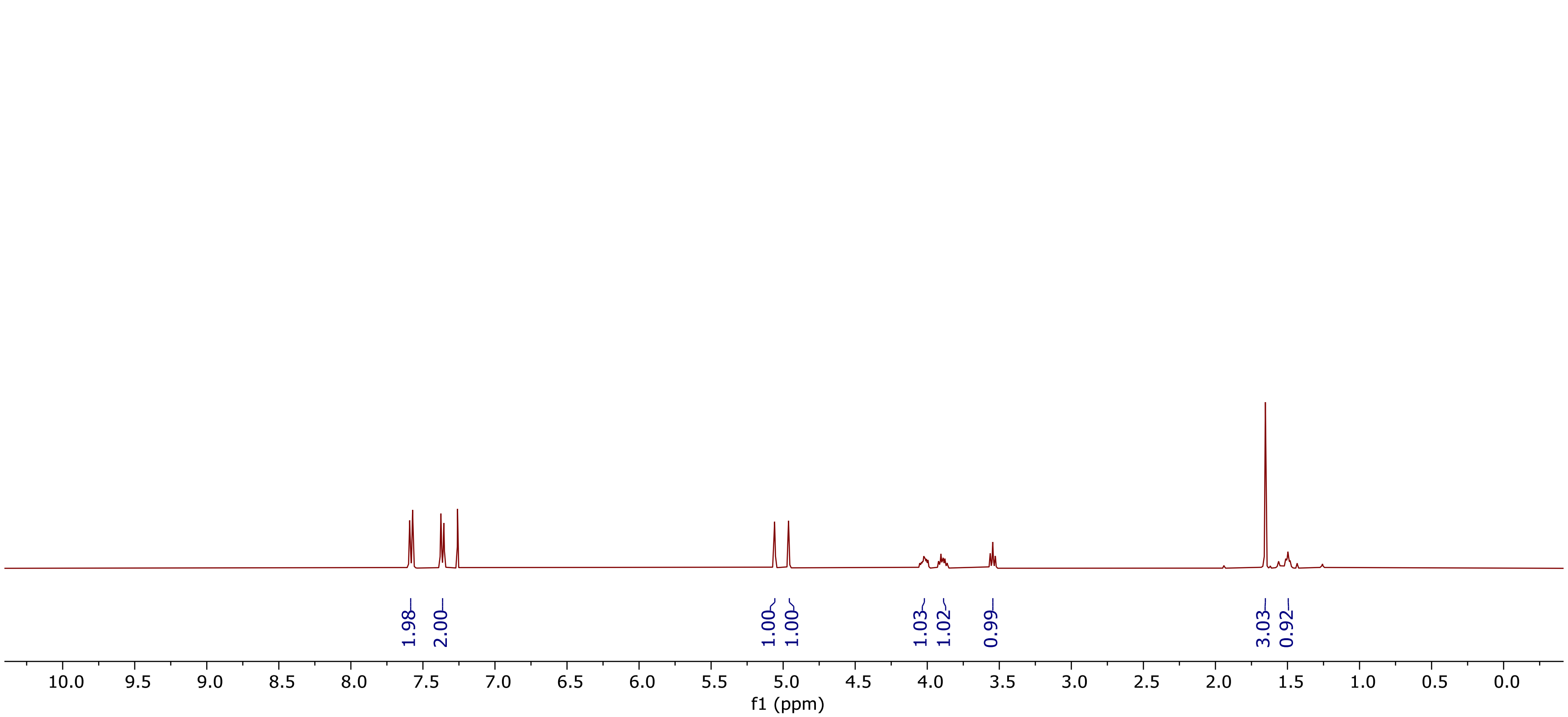

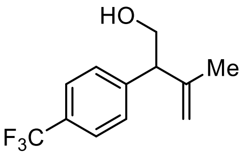

2'q

^{19}F{^{1}H} NMR (282 MHz, $CDCl_3$)

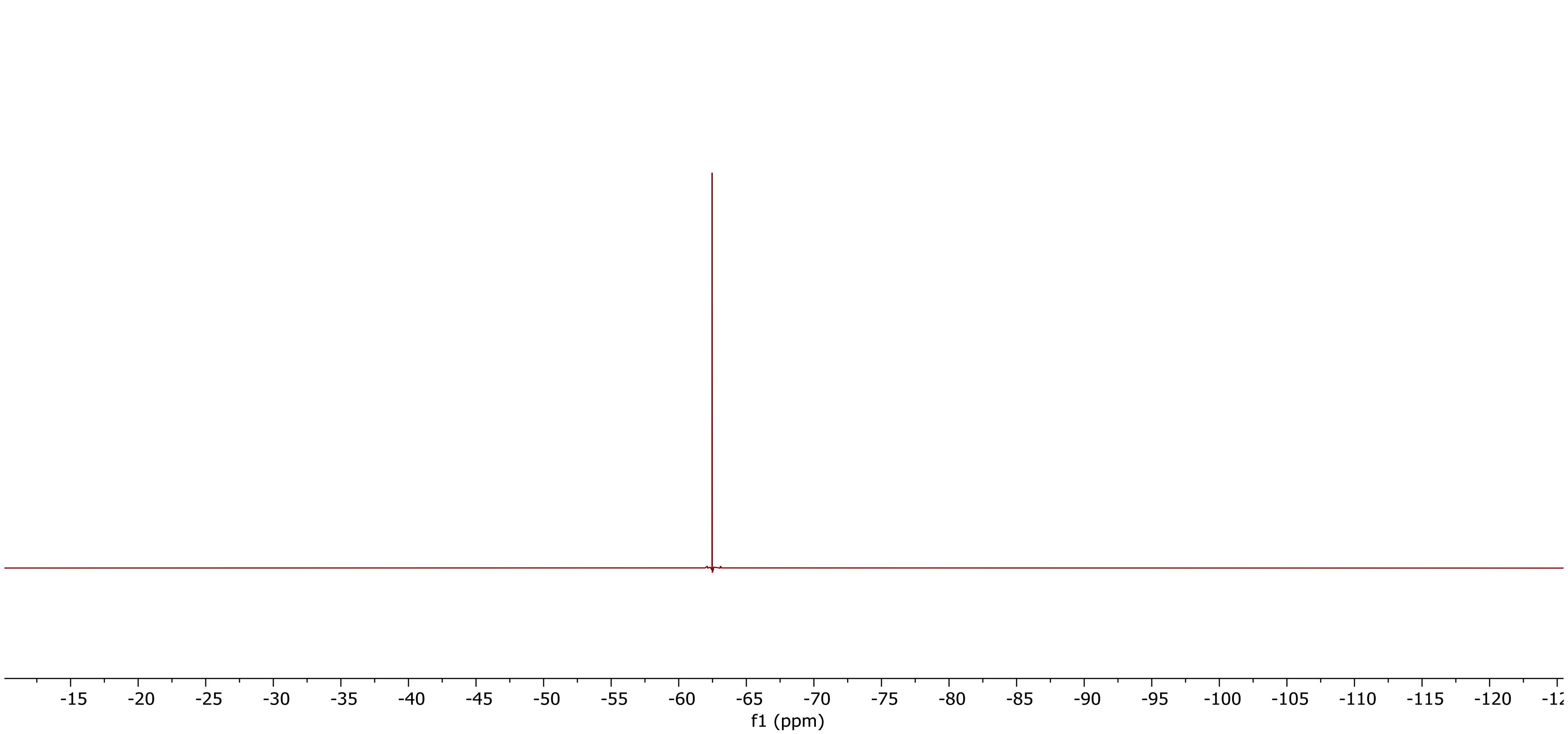

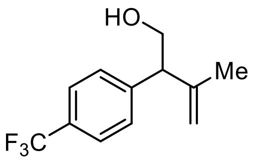

2'q

$^{13}C\{^{1}H\}$ NMR (101 MHz, $CDCl_3$)

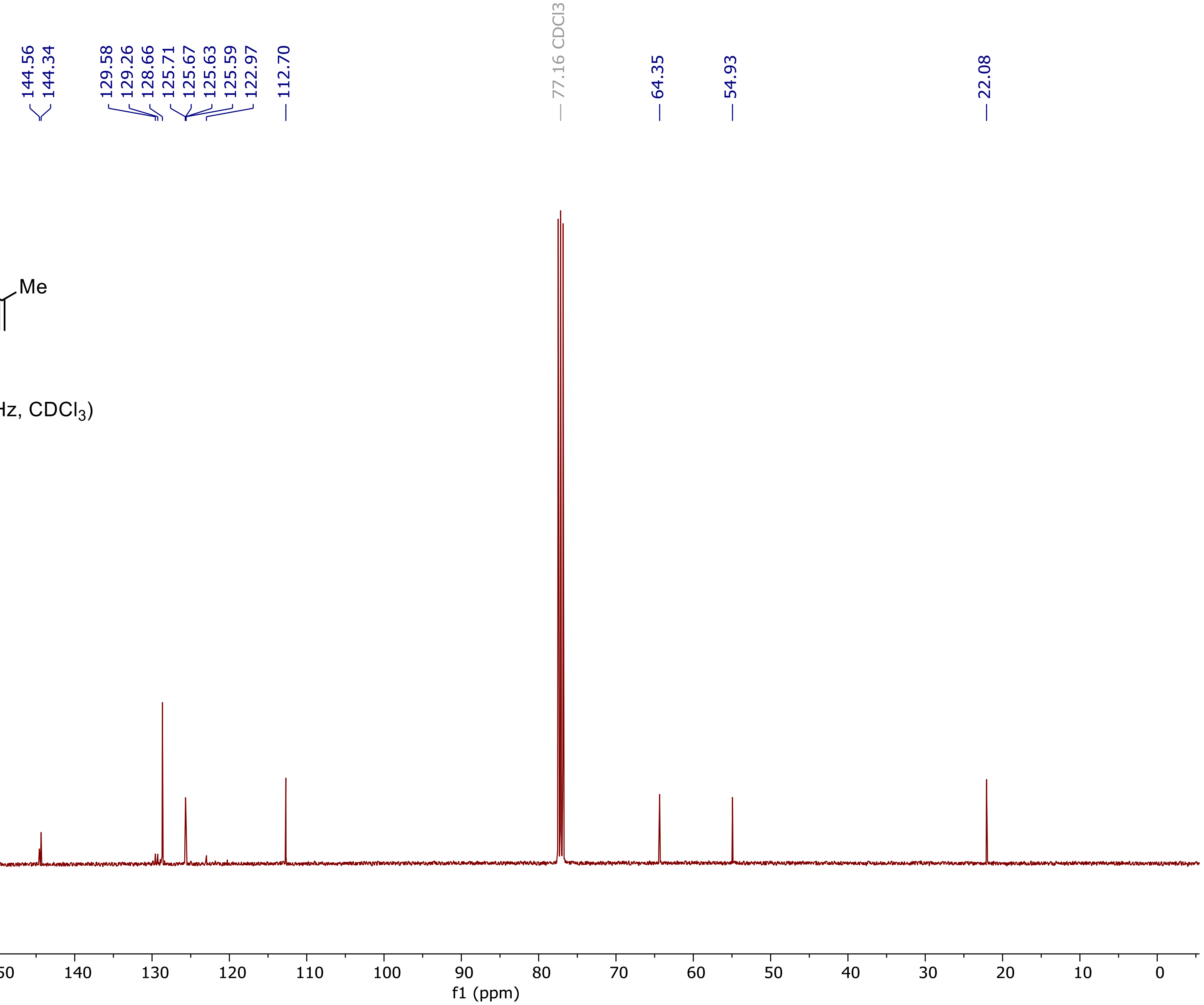

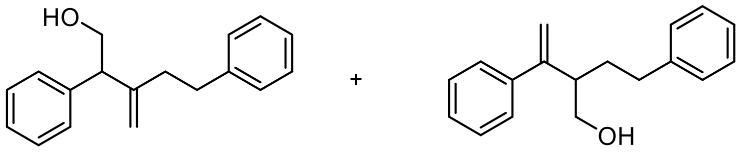

2'r ♣ 4'r ♥

^{1}H NMR (400 MHz, $CDCl_3$)

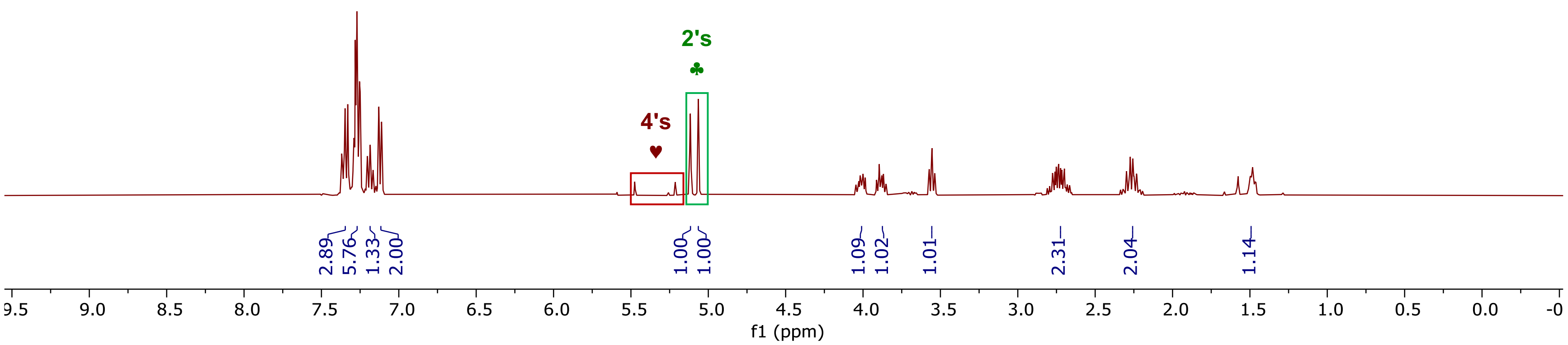

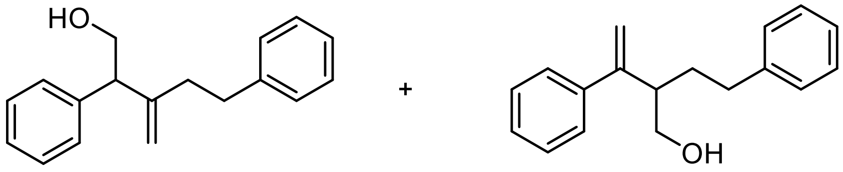

2'r **4'r**

$^{13}C\{^1H\}$ NMR (101 MHz, $CDCl_3$)

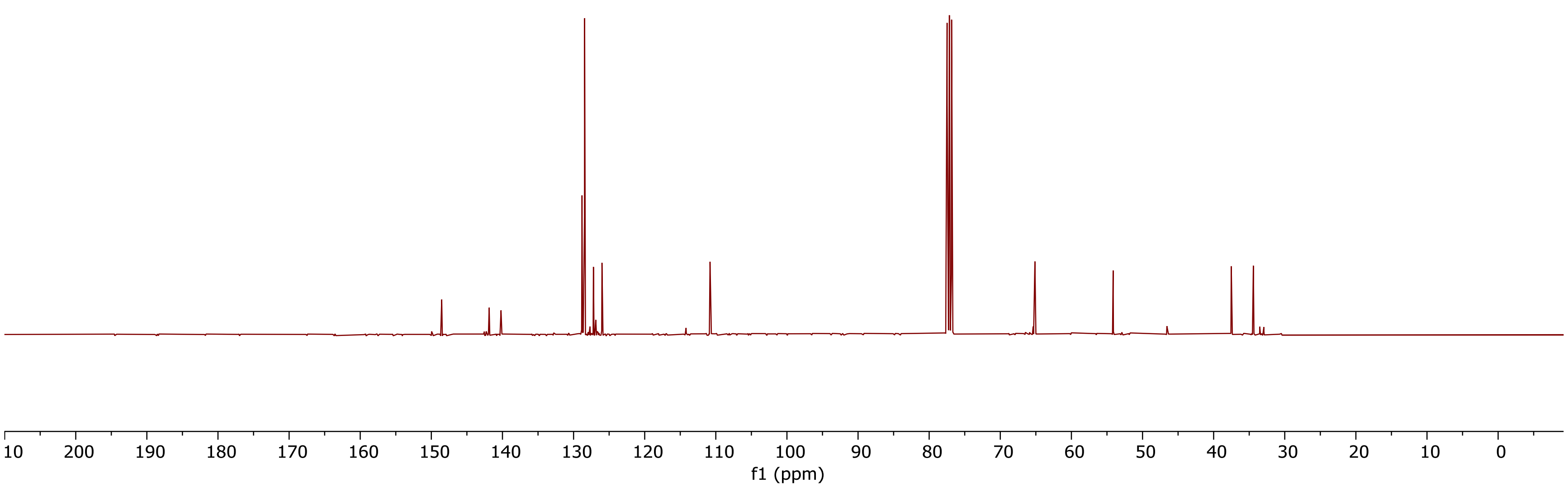

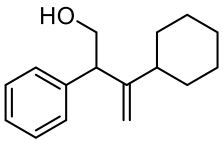

2's

^{1}H NMR (400 MHz, $CDCl_3$)

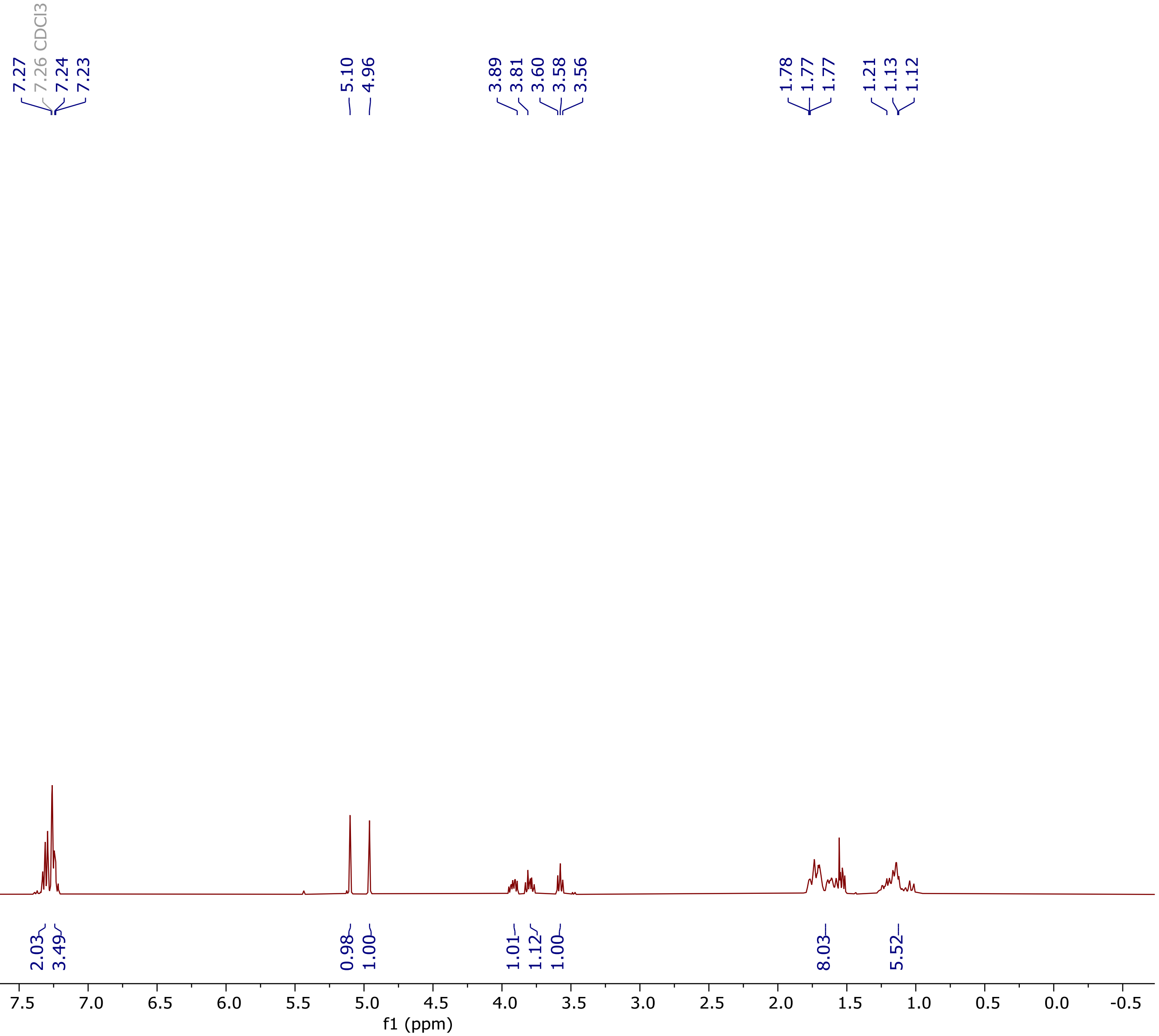

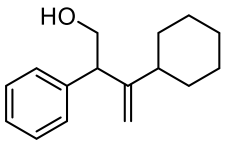

2's

^{13}C{^{1}H} NMR (101 MHz, $CDCl_3$)

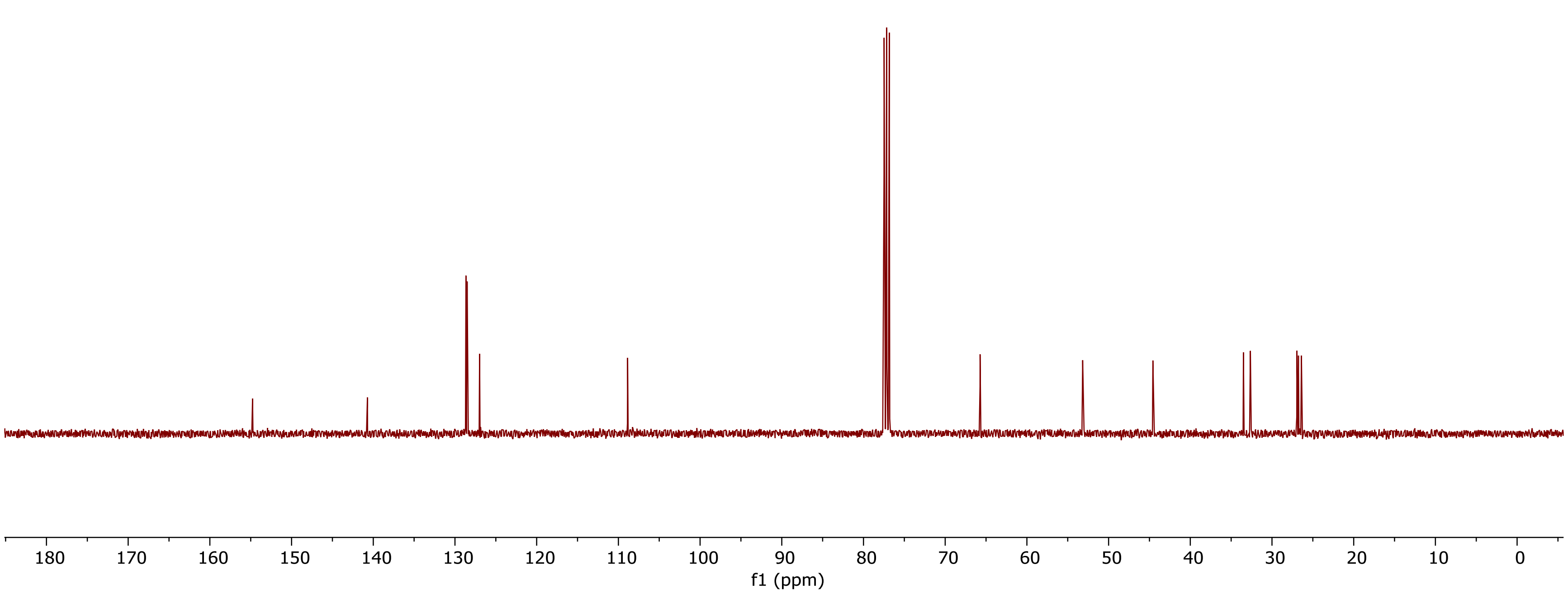

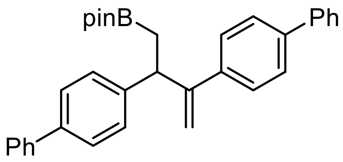

2d

^{1}H NMR (400 MHz, $CDCl_3$)

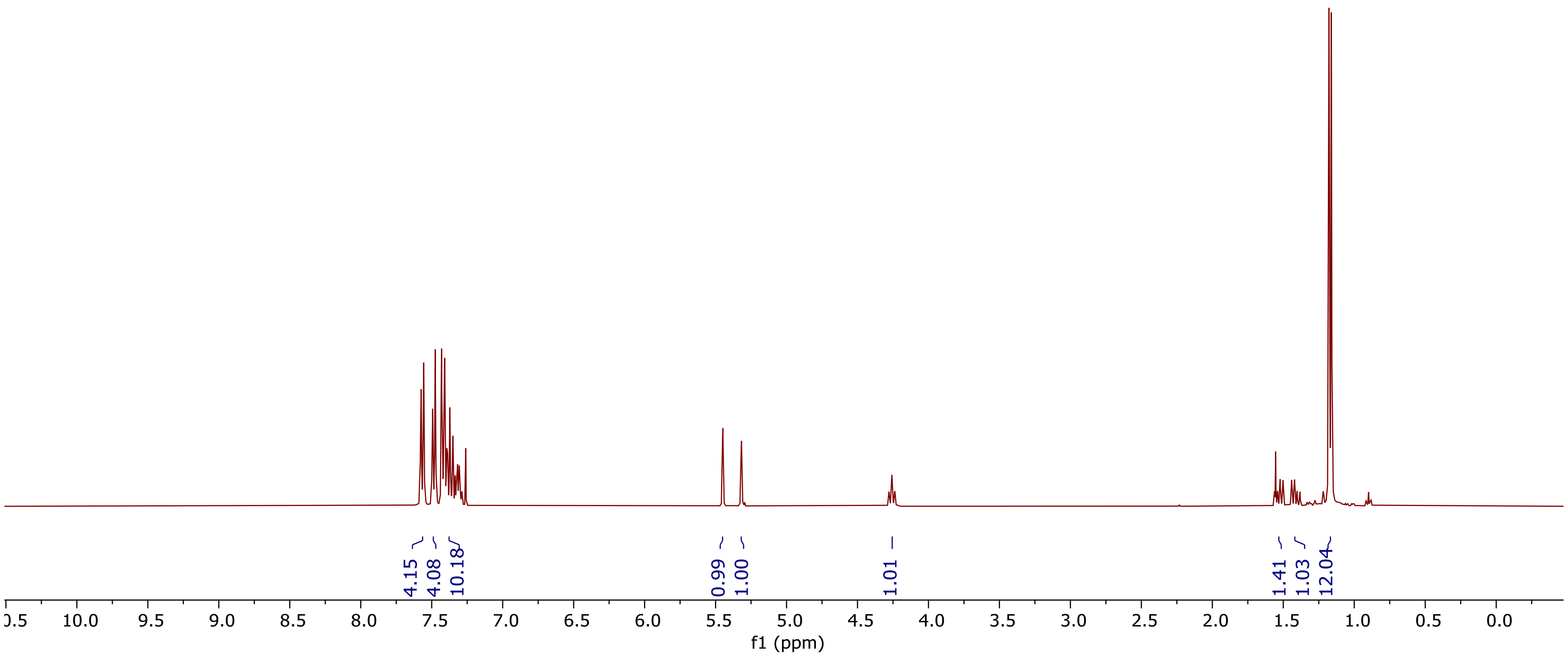

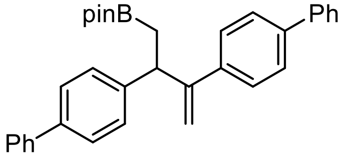

2d

^{13}C{^{1}H} NMR (101 MHz, $CDCl_3$)

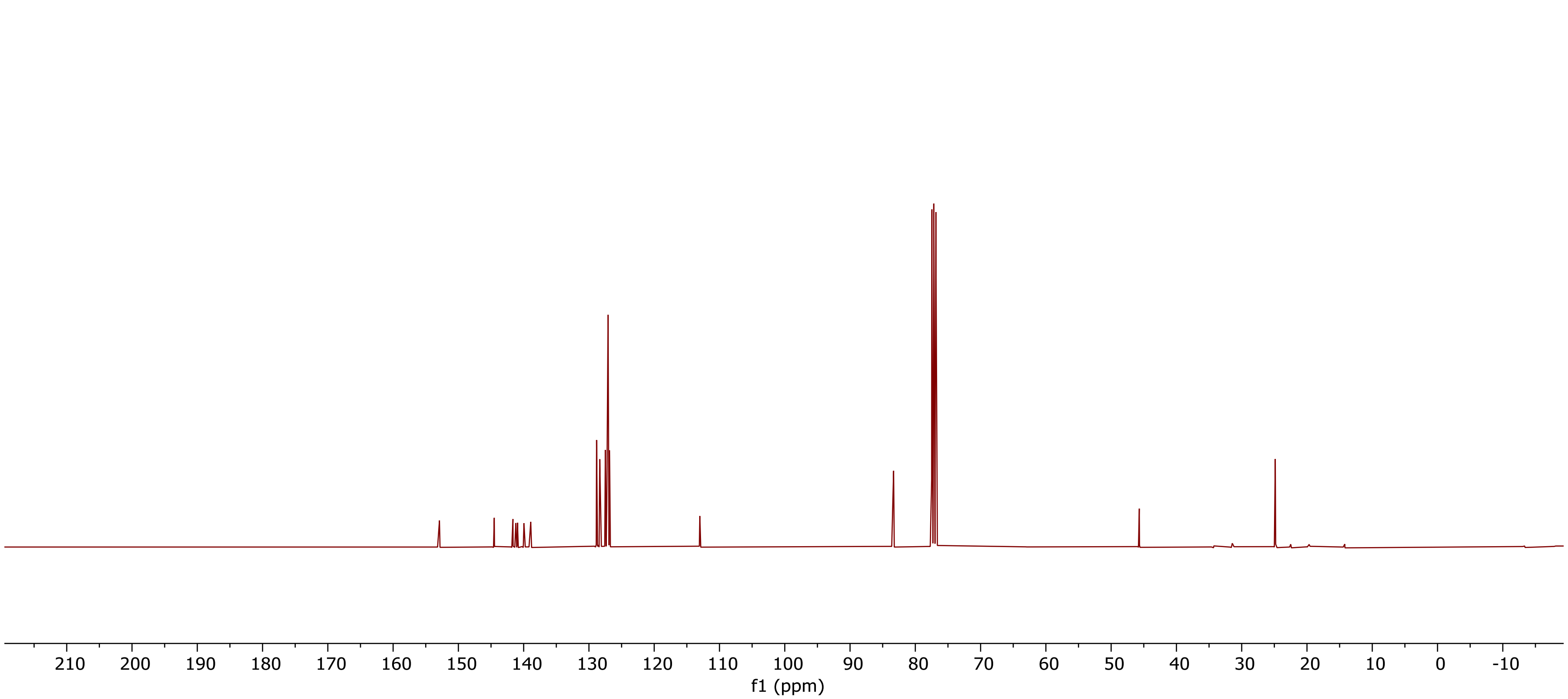

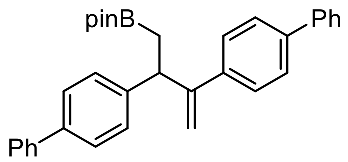

2d

^{11}B{^{1}H} NMR (128 MHz, $CDCl_3$)

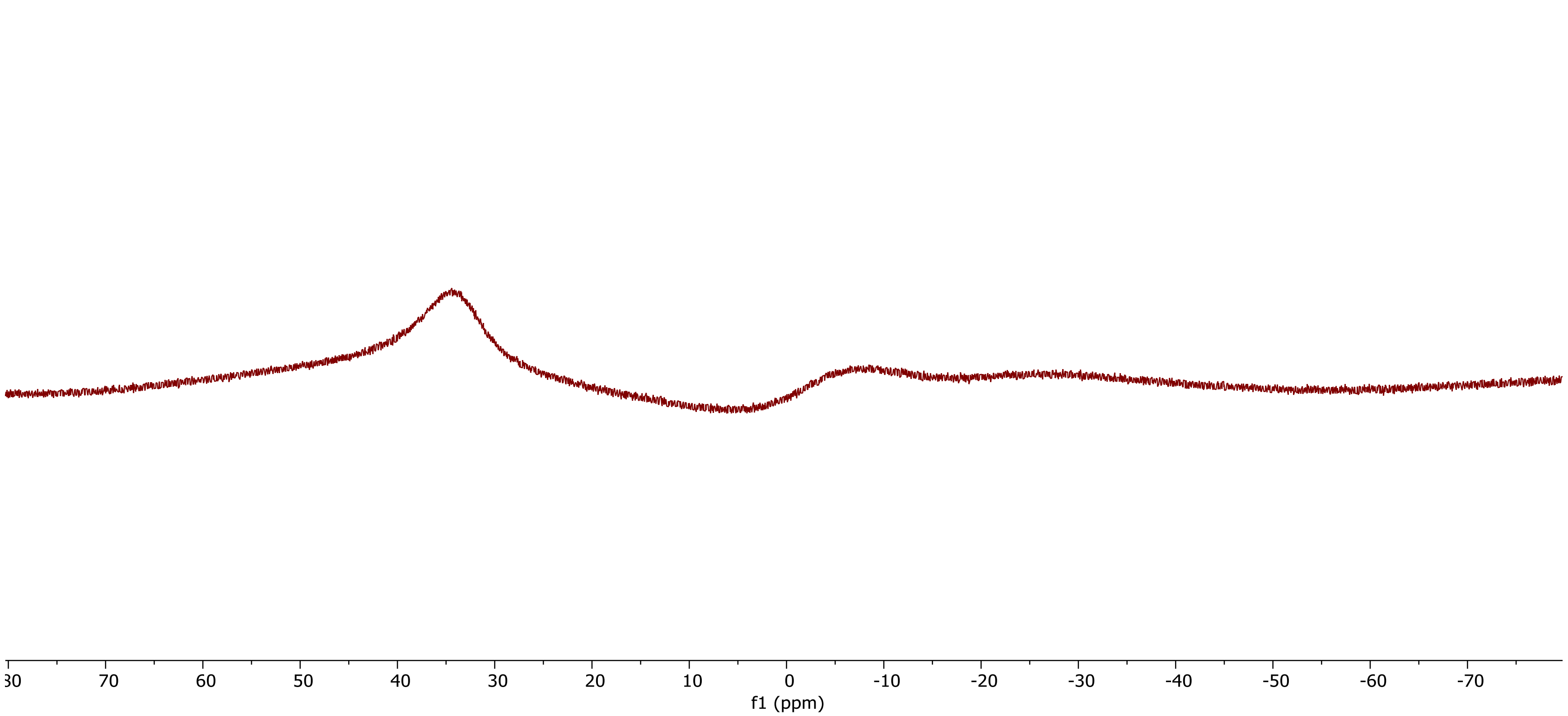

9. NMR spectra of known compounds

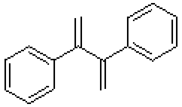

1a

^{1}H NMR ($CDCl_3$, 400 MHz)

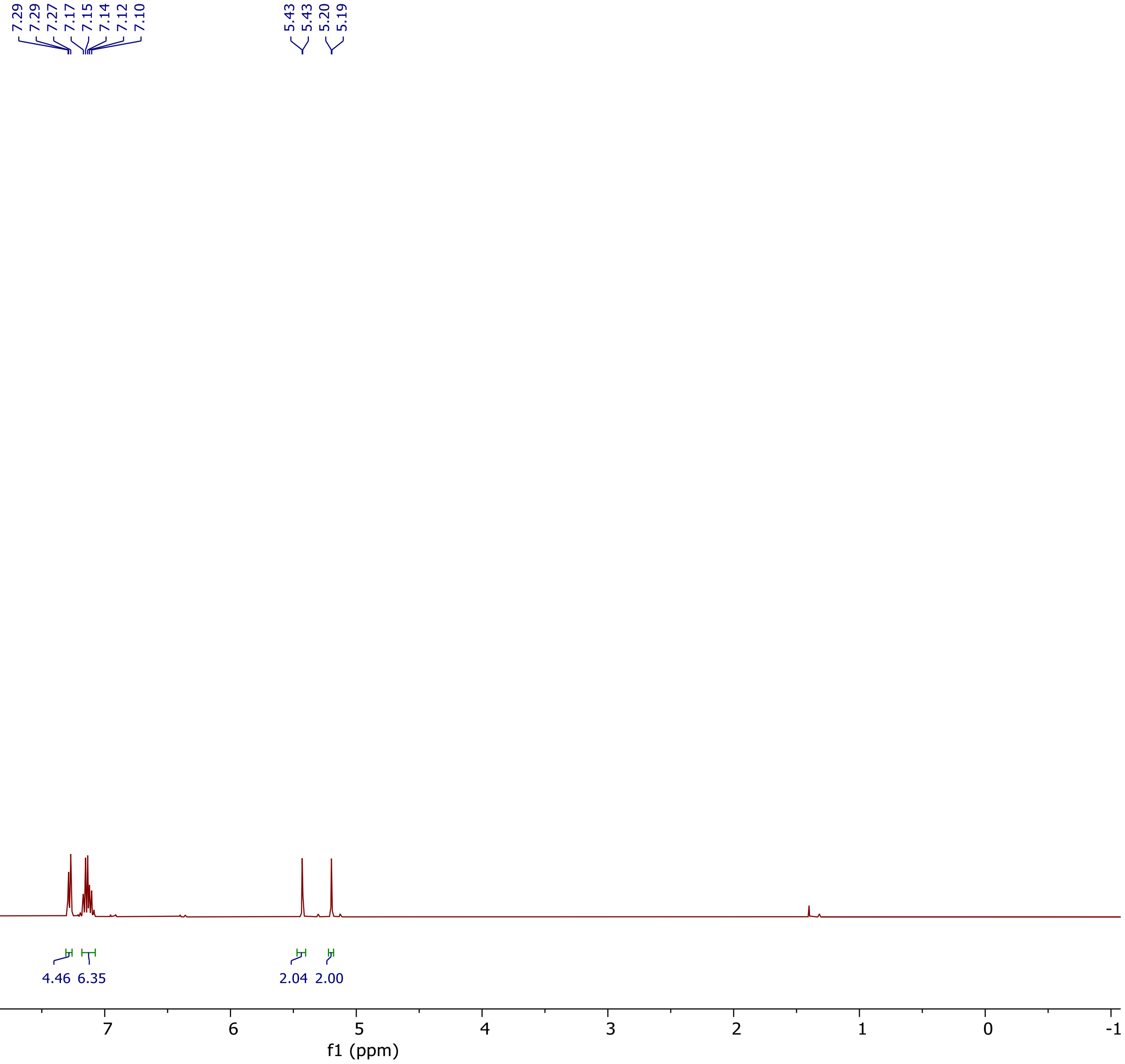

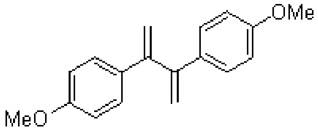

1b

^{1}H NMR ($CDCl_3$, 300 MHz)

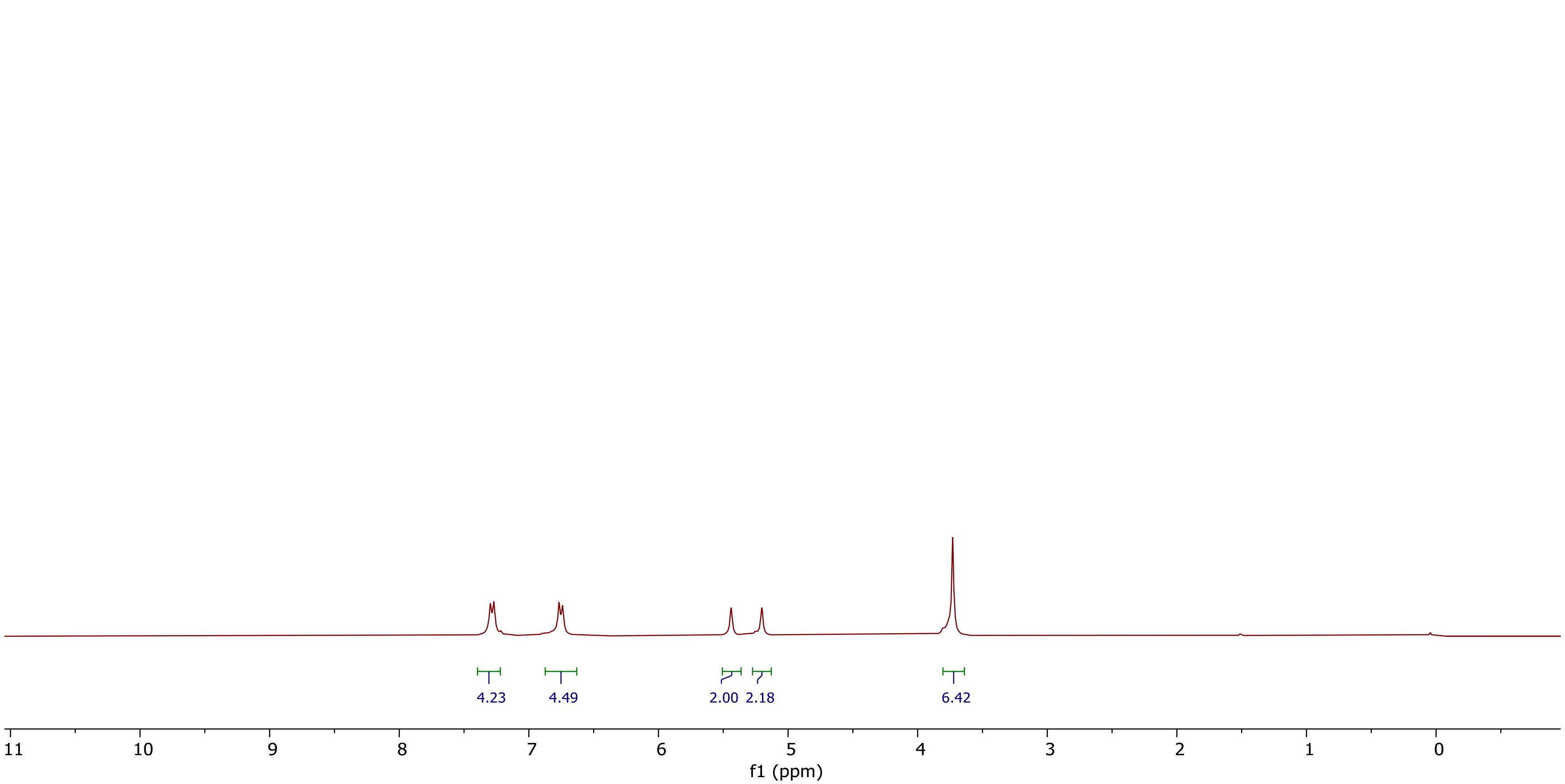

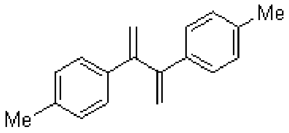

1c

^{1}H NMR ($CDCl_3$, 400 MHz)

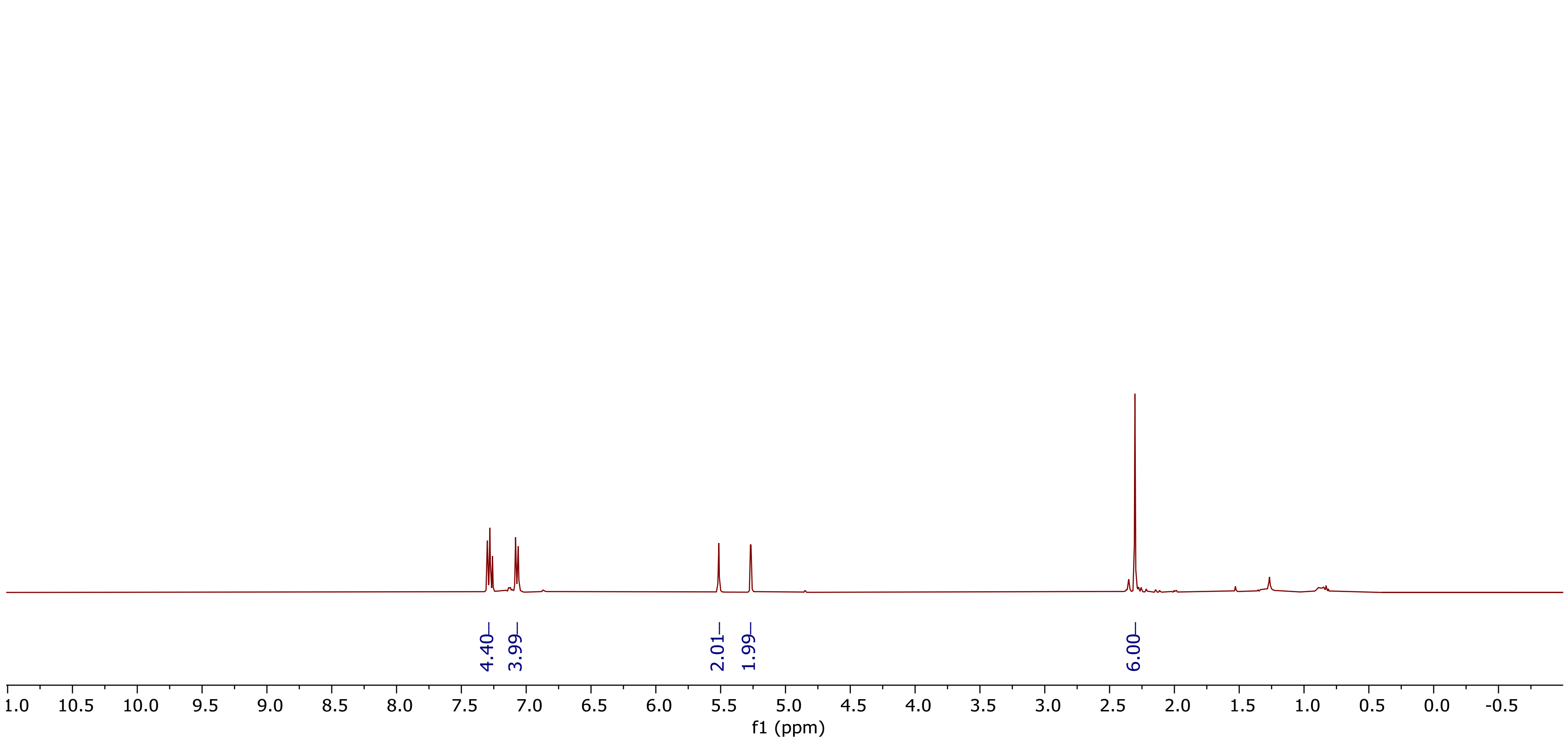

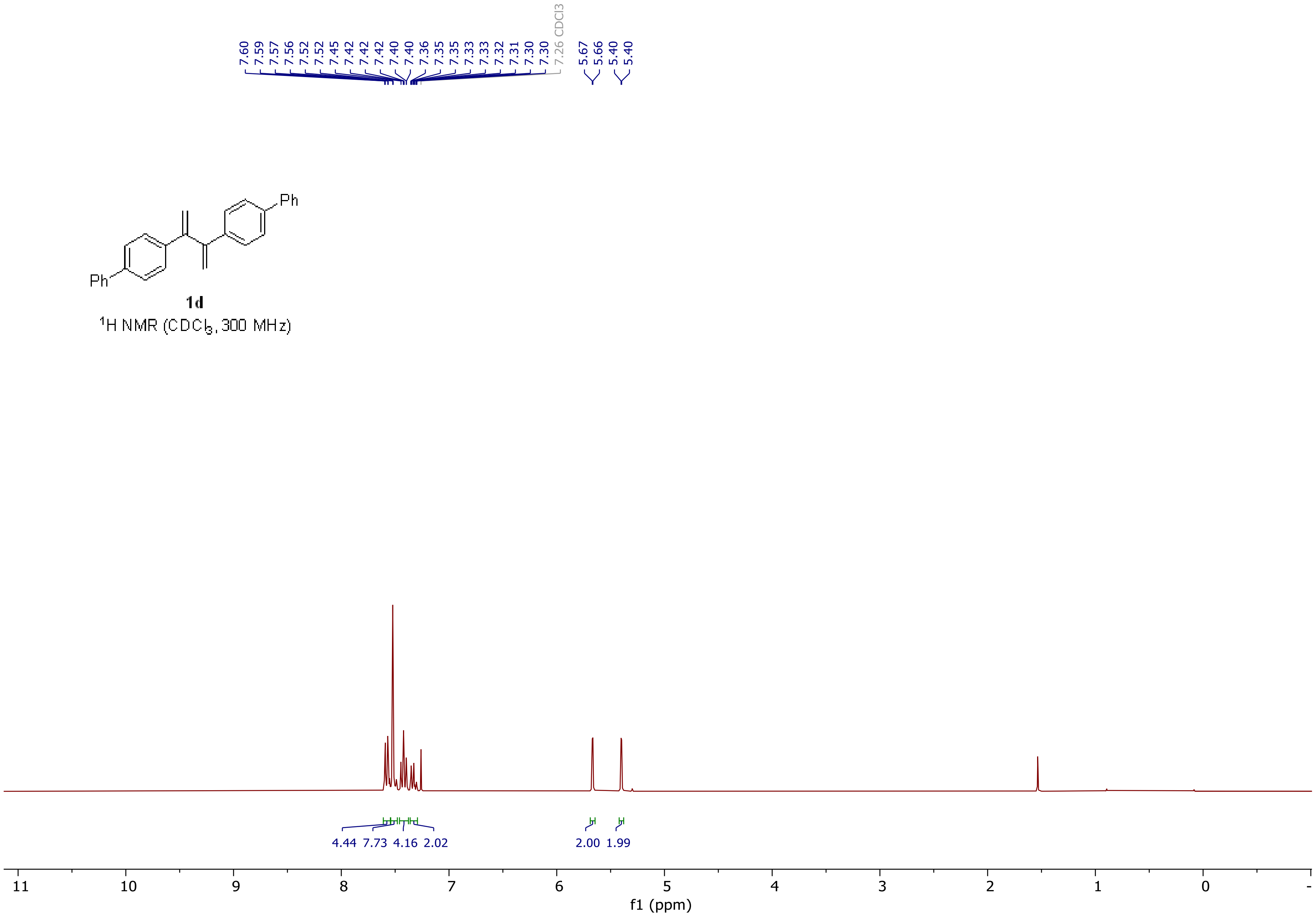
7.60 7.59 7.57 7.56 7.52 7.52 7.45 7.42 7.42 7.42 7.40 7.40 7.36 7.35 7.35 7.33 7.33 7.32 7.31 7.30 7.30
7.26 CDCl3
5.67 5.66 5.40 5.40
Ph
Ph
1d
^{1}H NMR (CDCl$_3$, 300 MHz)
4.44 7.73 4.16 2.02
2.00 1.99
11 10 9 8 7 6 5 4 3 2 1 0
f1 (ppm)

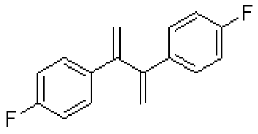

1e

^{1}H NMR (CDCl$_3$, 400 MHz)

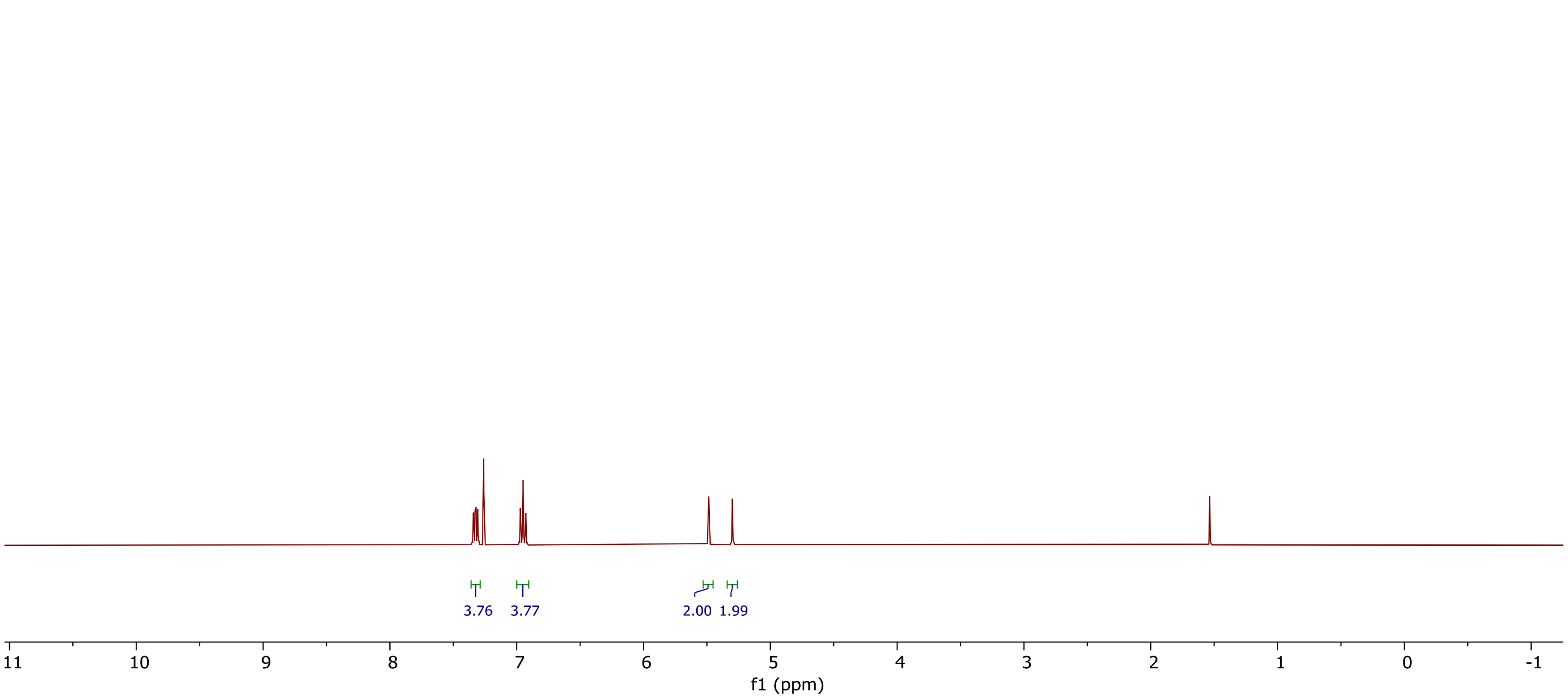

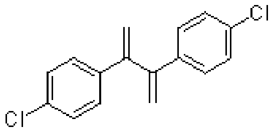

1f
^{1}H NMR ($CDCl_3$, 400 MHz)

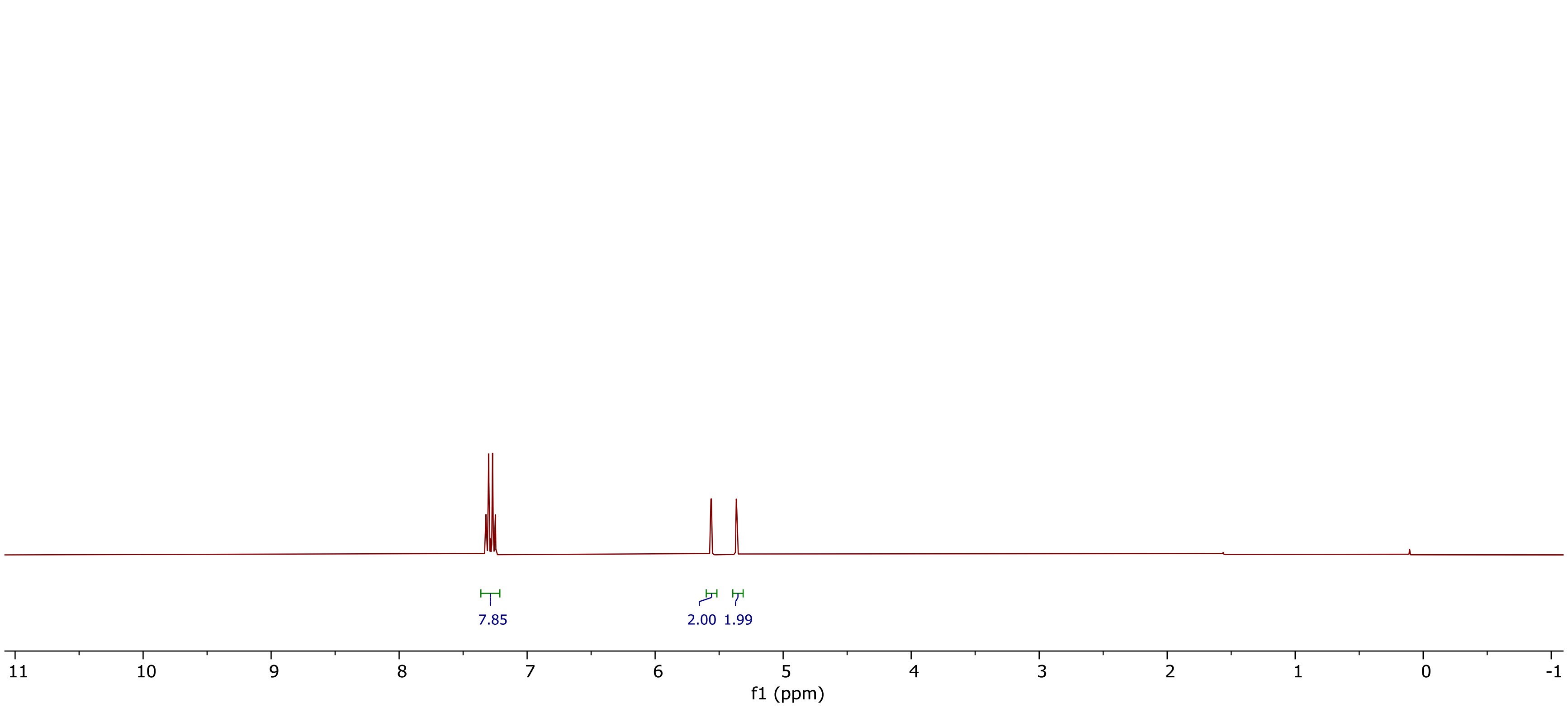

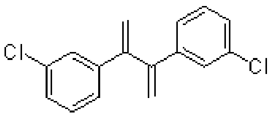

1g

^{1}H NMR ($CDCl_3$, 300 MHz)

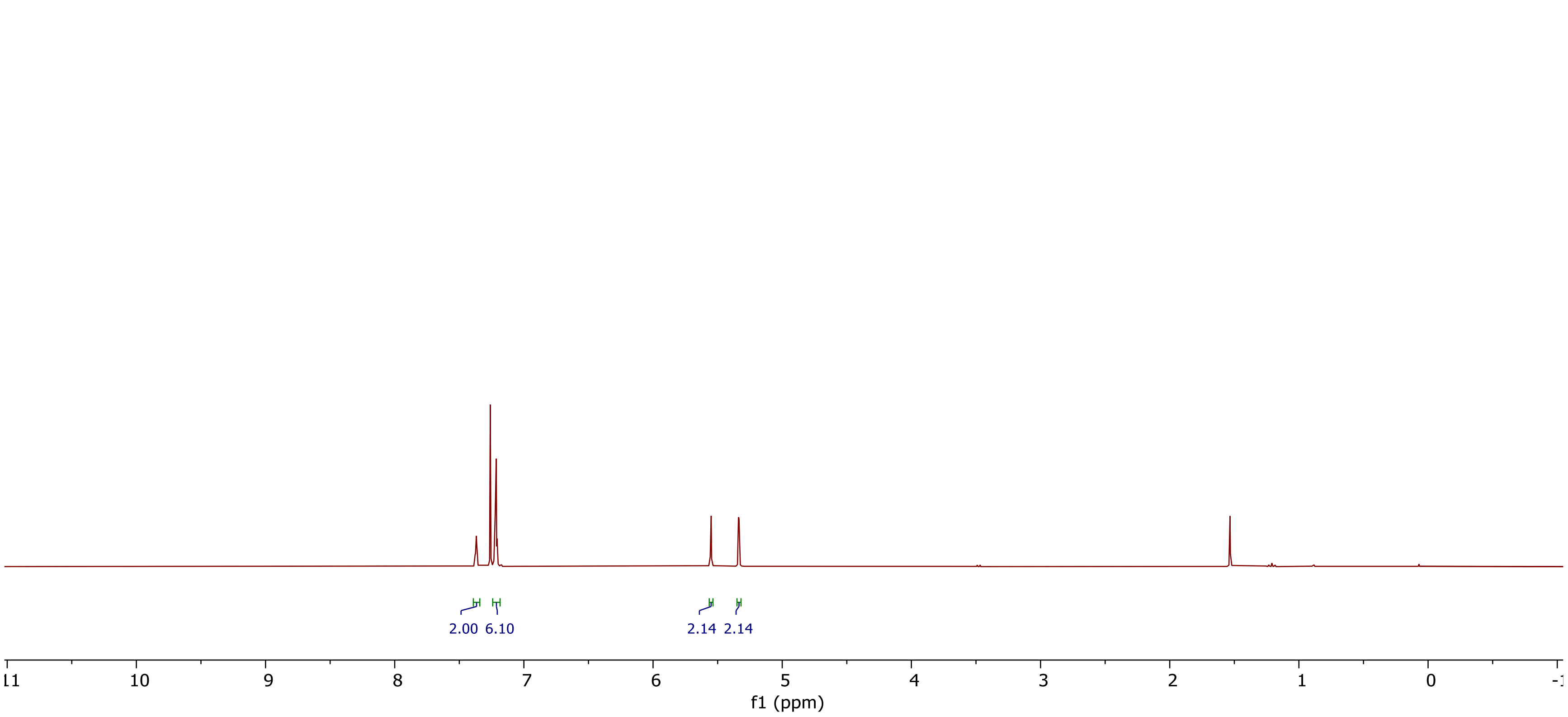

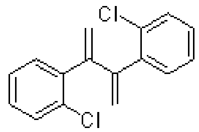

1h

^{1}H NMR ($CDCl_3$, 300 MHz)

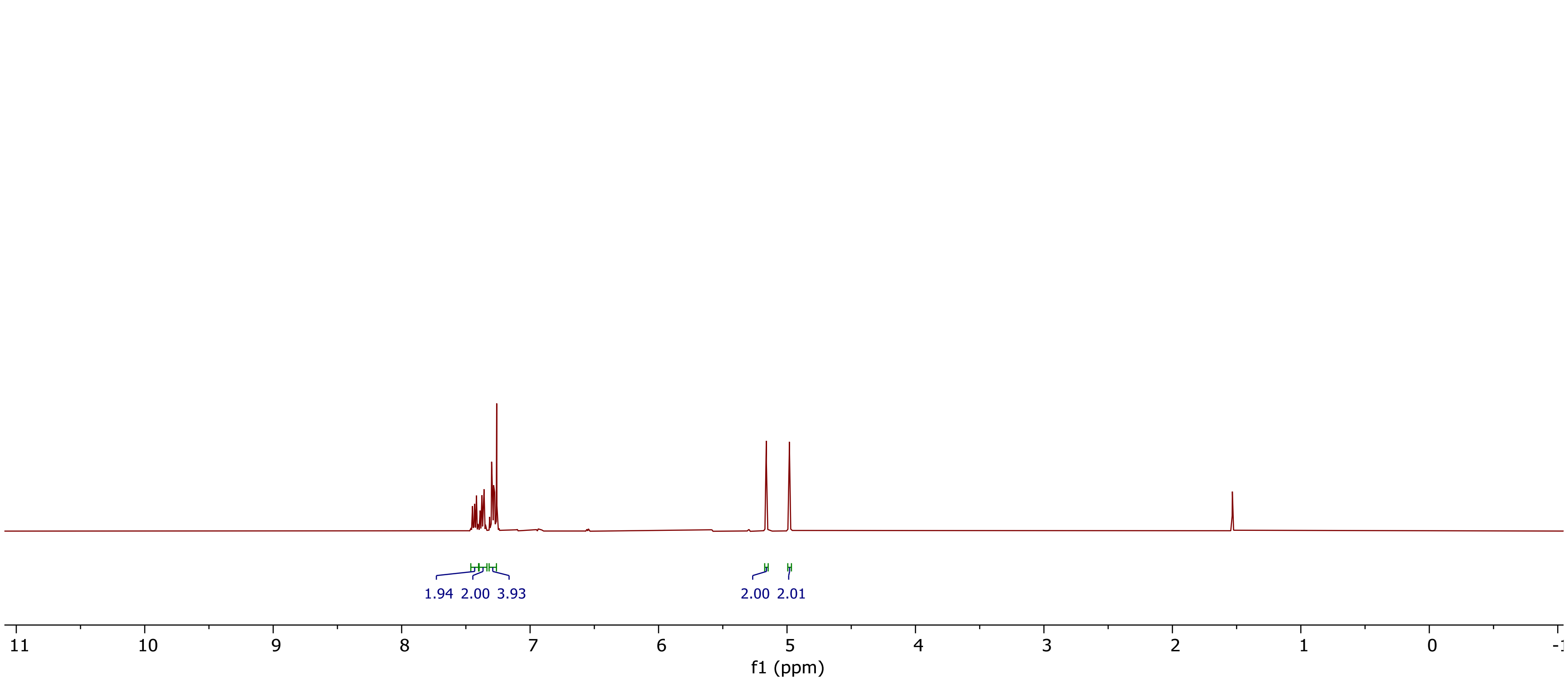

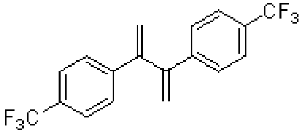

1i

^{1}H NMR ($CDCl_3$, 300 MHz)

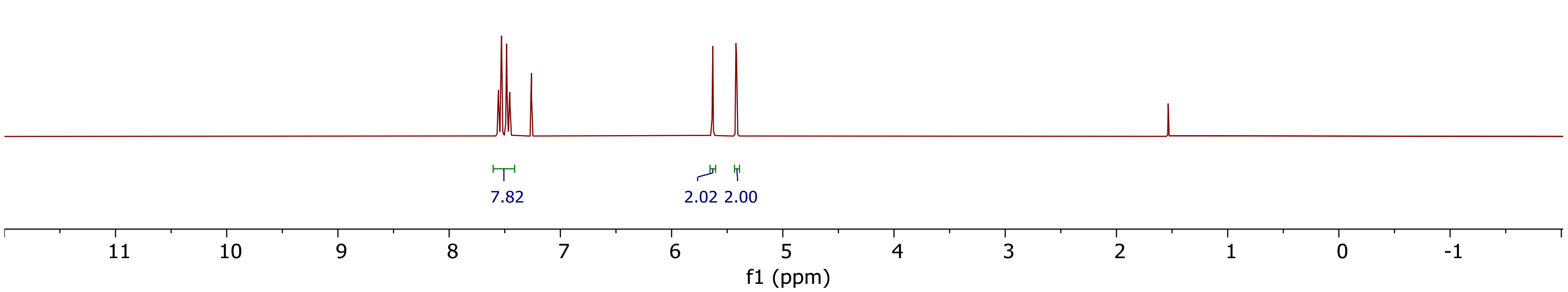

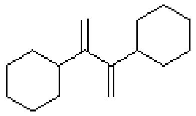

1k
^1H NMR ($CDCl_3$, 400 MHz)

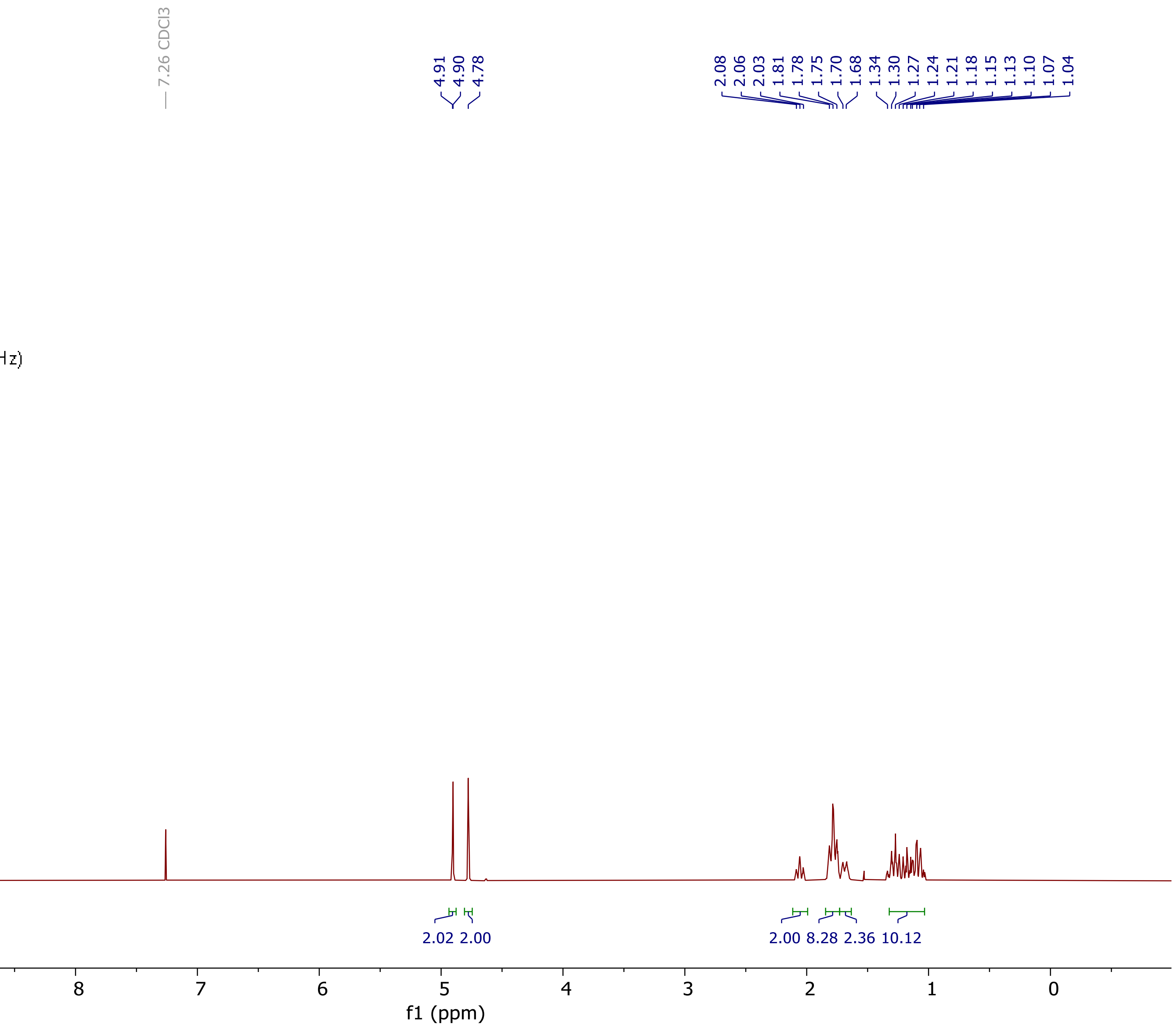

Supplement: Supplementary file 1 — ol3c02627_si_001.pdf [file ol3c02627_si_001.pdf]
